# Metatranscriptomic insights into the effect of *Lactiplantibacillus plantarum* inoculation on fermentation metabolism, bacterial community function and antibiotic resistome of corn silage

**DOI:** 10.3389/fmicb.2026.1876540

**Published:** 2026-09-09

**Authors:** Neha Sheoran, Xusheng Guo, Rina Su, Samaila Usman, Dongmei Xu

**Affiliations:** 1 School of Life Sciences, Lanzhou University, Lanzhou, China; 2 Probiotics and Life Health Institute, Lanzhou University, Lanzhou, China; 3 College of Life Science and Technology, Inner Mongolia Normal University, Hohhot, China

**Keywords:** antibiotic resistance genes (ARGs), fermentation quality, gene expression, *Lactiplantibacillus plantarum*, metatranscriptomics, nitrogen metabolism

## Abstract

Lactic acid bacteria inoculants are widely applied in silage production; however, it remains unclear how *Lactiplantibacillus plantarum* inoculation shapes temporal changes in microbial function during ensiling, and whether it accelerates normal successional process or is associated with changes in antibiotic resistome dynamics. In this study, we applied an integrated metatranscriptomic approach combining microbial community profiling, differential expression analysis, carbohydrate-active enzyme (CAZy) profiling, and resistome characterization to analyze whole-crop corn silage inoculated with *L. plantarum* at fresh material (day 0), early fermentation (day 3), and late fermentation (day 90). The results indicated that inoculation was associated with higher expression of central carbon genes, including >30-fold induction of *ackA* at day 3 and 44 differentially expressed genes at day 90, together with higher residual water-soluble carbohydrates and reduced late-stage acetic acid accumulation. Nitrogen-related gene expression underwent marked temporal restructuring, shifting from broad early enrichment of *glnA* and denitrification-linked genes to targeted late-stage enrichment of *nirA* involved in nitrite assimilation. In LP-treated silage, we did not detect statistically significant temporal differences in CAZyme family-level transcript abundance between day 3 and day 90, whereas 56.4% of families shifted significantly over time in control silage. An existing core resistome was dominated by *tet(C), APH (3’)-Ia, bacA*, and *tet(M)*, collectively accounting for 85%–94% of total ARG abundance across all treatments. Resistome configuration was determined mainly by fermentation time rather than LP inoculation. Notably, LP-driven gene expression changes did not increase the actively transcribed antibiotic resistome under the tested conditions, suggesting that the microbial community shifted without expanding the detected resistome profile. Overall, these results show that *L. plantarum* inoculation improved fermentation characteristics by reducing acetic acid accumulation and preserving water-soluble carbohydrates and was associated with differences in the late-stage dominant taxa and functional gene transcription but did not increase the active antibiotic resistome under controlled laboratory conditions. These results support the potential application of this inoculant in laboratory-scale silage production, although further validation across different forage types, strains, and field conditions is warranted.

## 1 Introduction

Silage fermentation is a classic resource-limited ecosystem: progressive depletion of fermentable substrates and organic acid accumulation jointly create energetic and chemical constraints that shape community composition, resource allocation, and function over time (Borreani et al., 2018; Zhao et al., 2021). *Lactiplantibacillus plantarum* is among the most broadly applied inoculants in silage production. There is consistent evidence across forage types for accelerated acidification, reduced proteolysis, and improved dry matter preservation with this inoculant (Guo et al., 2023; Ridwan et al., 2023; Akhtar et al., 2025). The above effects of fermentation end-products and community composition are well-characterized by culture-based and 16S amplicon approaches, but the temporal patterns of metabolic gene expression across the full ensiling period from the high resource phase through late substrate-limited stabilization have not been studied using metatranscriptomics (Sun et al., 2021; Xiao et al., 2023). Unlike 16S rRNA sequencing and metagenomic inference of functional potential, metatranscriptomics captures actively expressed genes and provides insights into microbial transcriptional responses (Aguiar-Pulido et al., 2016). This makes it more suitable for studying dynamic functional changes in microbial gene expression during fermentation (Chen et al., 2015; Jovel et al., 2022). Therefore, integrating microbial community succession with transcriptional responses provides an opportunity to understand the influence of inoculation-driven shifts in fermentation metabolism and resistome dynamics.

Despite similar taxonomic compositions, microbial communities that operate under progressive resource limitation can show different functional patterns (Lennon and Jones, 2011; Bakkeren et al., 2025). Even under normalized conditions, small differences in the initial community composition can generate distinctive functional outcomes, as recently demonstrated in replicated synthetic microbial community experiments (Pascual-García et al., 2025). Under progressive resource depletion, communities diverge not only in composition but in whether they maintain broad metabolic activity or shift toward narrower, stress-dominated functional states, a distinction that suggests differences in energy allocation, not taxonomy alone (Wood et al., 2023). In silage, *L. plantarum* inoculation has been shown to influence predicted carbohydrate and amino acid metabolic pathways, but mainly through static, DNA-based functional prediction at single ensiling time points (Xu et al., 2024; Meng et al., 2025).

Carbon and nitrogen metabolism are tightly coupled in resource-limited microbial systems: continuous carbon flux drives simultaneous upregulation of nitrogen transformation and gene expression and assimilation, with glutamine synthetase (glnA) and GS-GOGAT components serving as central markers of nitrogen status under limitation (van Heeswijk et al., 2013; Chuckran et al., 2021). This coupling is directly demonstrated in soil metatranscriptomic studies, where labile carbon additions change nitrogen cycling gene transcription including, nifH, nirA, and glnA, through temporally structured, carbon-stoichiometry-driven responses (Séneca et al., 2021; Rosado-Porto et al., 2022). Although soil and silage systems differ, both are resource-limited environments where carbon availability influences nitrogen-carbon metabolism. Whether *L. plantarum* inoculation also alters nitrogen-related gene transcription alongside central carbon pathways during ensiling, and whether carbon-driven nitrogen transcriptional coupling documented in soil systems operates in silage, remain unknown.

Silage functions as an agricultural-scale antibiotic resistance gene (ARGs) reservoir and can transfer ARGs to ruminant gut microbiomes before returning them to the environment through manure application (Nagy et al., 2022). Since microbial inoculants alter bacterial community succession, ecological interactions, and metabolic activity during fermentation, they may also indirectly influence the composition and transcription of ARGs by favoring bacterial taxa with different intrinsic resistance properties. Therefore, inoculation-driven changes in ARG expression may reflect shifts in the active microbial community rather than acquisition of new resistance determinants. Existing ARG studies are almost exclusively DNA-based and static, providing no insight into how inoculant-driven metabolic shifts relate to resistome dynamics over time. Whether *L. plantarum* inoculation introduces novel ARGs, changes the class-level collection of expressed resistance determinants, or primarily rearranges the activity of a pre-existing core resistome remains unknown.

In this study, we applied an integrated metatranscriptomic system to characterize how *L. plantarum* inoculation alters the temporal metabolic dynamics of whole-crop corn silage across fresh material, early (day 3), and late (day 90) fermentation. To obtain a system-level understanding of the effects of *L. plantarum* inoculation on silage fermentation, we evaluated four interconnected aspects of microbial responses across fresh material, early (day 3), and late (day 90) fermentation stages.

We particularly asked: (i) whether inoculation maintains central carbon gene expression beyond early acidification and changes substrate utilization course; (ii) whether LP inoculation temporally alters nitrogen-cycling gene expression over time; (iii) whether LP inoculation reduces temporal instability in carbohydrate-active enzyme profiles compared to control silage; and (iv) whether LP inoculation changes ARG composition, expression patterns, or genetic diversity of resistance determinants. Furthermore, we assessed if LP-driven community restructuring changed the actively expressed resistome. By incorporating these aspects across fermentation stages, this study aims to establish whether inoculation accelerates normal succession or redirects fermentation to a distinct metabolic state.

## 2 Materials and methods

### 2.1 Silage preparation

Whole-crop corn (*Zea mays L.*) at the half-milk line stage was harvested from a commercial planting field in Wuwei City, Gansu Province, China. The whole-plant corn was chopped immediately after harvest to a theoretical particle length of 1–2 cm before ensiling. The chopped forage was then divided into two treatment groups with three replicates each. The control (CK) group was treated with sterile distilled water and ensiled for 3 and 90 days. The inoculated group (LP) was treated with *Lactiplantibacillus plantarum* MTD1 at a dosage of 1 × 10^5^ colony-forming units (CFU) per gram of fresh matter (FM) and ensiled for 3 and 90 days. The inoculant was dissolved in distilled water and sprayed uniformly onto the forage, while the control received an equal volume of water. Approximately 300 g of forage was packed into separate polyethylene bags (30 × 23 cm) and vacuum-sealed to ensure proper anaerobic conditions. Three biological replicates were prepared for each treatment at two sampling time points: 3 and 90 days, resulting in a total of 12 silage samples and three fresh material samples. Bags were then stored at ambient temperature (20 °C–25 °C) until opening. At each time point, bags were opened, and samples were collected for fermentation quality and RNA extraction.

### 2.2 Chemical composition and fermentation quality analysis

At each sampling point, 20 g of fresh silage were homogenized with 180 mL distilled water using a high-speed blender for 30 s. The homogenate was filtered through four layers of sterile gauze, and the pH of the filtrate was measured immediately using a calibrated pH meter (PB-10, Sartorius, Germany). For organic acid analyses, an aliquot of the filtrate was acidified with 50% H_2_SO_4_ (w/w) to pH < 2 and passed through a 0.22 μm membrane filter prior to injection. Concentrations of lactic acid, acetic acid, propionic acid, and butyric acid were determined by high-performance liquid chromatography (HPLC; Agilent 1260 Infinity, Agilent Technologies, Santa Clara, CA, United States) equipped with a C-811 ion-exclusion column (Shodex, Tokyo, Japan) at a column temperature of 50 °C, using 2.5 mM H_2_SO_4_ as the mobile phase with a flow rate of 1.0 mL/min and UV detection at 210 nm.

For ammonia nitrogen (NH_3_-N) determination, a separate aliquot of non-acidified filtrate was mixed with 250 g/L trichloroacetic acid (1:4, v/v), incubated overnight at 4 °C, and centrifuged at 18,000 × *g* for 15 min. The concentration of NH_3_-N in the resulting supernatant was determined colorimetrically using the phenol-sodium hypochlorite method, and results were expressed as g/kg TN (total nitrogen) (Kamali et al., 2023).

The remaining silage was dried in a forced-air oven at 65 °C for 72 h to determine dry matter (DM) content. Dried samples were ground to pass through a 1 mm screen prior to chemical analyses. Crude protein (CP) content was determined by the Kjeldahl method using a nitrogen-to-protein conversion factor of 6.25, and expressed as g/kg DM (Al-Mentafji, 2016). Water-soluble carbohydrate (WSC) concentration was determined calorimetrically using the anthrone–sulfuric acid method (Gnoumou et al., 2022).

### 2.3 RNA extraction and metatranscriptome sequencing

Total RNA was extracted from the silage precipitate using the RNA PowerSoil^®^ Kit (MoBio, cat. no. 12866-25) according to the manufacturer’s instructions. RNA quality was assessed by 1.5% agarose gel electrophoresis, and RNA integrity was determined using an Agilent 2100 Bioanalyzer (Agilent Technologies, Santa Clara, CA, United States); only samples meeting the minimum integrity threshold (RIN > 7.0) were retained for library preparation. Following ribosomal RNA depletion with the Ribo-Zero Magnetic Kit (Illumina, San Diego, CA, United States), strand-specific cDNA libraries were constructed using the TruSeq Stranded mRNA LT Kit (Illumina). Libraries were sequenced on an Illumina NovaSeq 6000 platform (Illumina) in paired-end mode (2 × 150 bp) at Bioyi Biotechnology Co., Ltd. (Wuhan, China).

Raw reads were subjected to quality control and adapter trimming using fastp (v0.23.4). Residual rRNA sequences were removed using SortMeRNA (v4.3.6) (Kopylova et al., 2012), and the resulting high-quality non-rRNA reads were retained for downstream analyses as described in section “2.4 Bioinformatic analyses.” Sequencing generated approximately 38.6–83.1 million raw reads per sample. After quality filtering, clean reads showed high sequencing quality, with Q20 and Q30 values exceeding 97% and 95%, respectively. The efficiency of rRNA removal ranged from 93.9% to 99.9% across samples.

### 2.4 Bioinformatic analyses

Following quality control and rRNA depletion as described in section “2.3 RNA extraction and metatranscriptome sequencing,” high-quality non-rRNA reads were used for all downstream analyses. Taxonomic classification was performed at the species level using Kraken2 (v2.1.4) (Wood et al., 2019) against the NCBI NT database (release 202503), and species-level abundance was estimated using Bracken (v2.6.1) (Lu et al., 2017) with KrakenTools used for format conversion and statistics. Species diversity and community composition were visualized using Krona (Ondov et al., 2011). Alpha diversity indices (Chao1, Shannon, and Simpson) were calculated and rarefaction curves were generated. Beta diversity was assessed using Bray-Curtis dissimilarity metrics and visualized by principal coordinate analyses (PCoA), non-metric multidimensional scaling (NMDS), and UPGMA hierarchical clustering. Group differences in community composition were tested using PERMANOVA based on Bray-Curtis dissimilarity distances. Differentially abundant taxa between groups were identified using LEfSe (linear discriminant analysis effect size) with a default LDA score threshold of >5.0. Multivariate dispersion was assessed using PERMDISP to evaluate whether differences in within-group variability contributed to beta diversity patterns.

High-quality reads were assembled using MEGAHIT (v1.2.9), and non-microbial contigs were removed by classification against the NCBI NT database (release 202503) with Kraken2 (Li et al., 2015). Open reading frames (ORFs) were predicted using MetaGeneMark (Gemayel et al., 2022), and a non-redundant gene catalog and protein set were constructed using CD-HIT (Li and Godzik, 2006). Transcript abundance was quantified using Salmon (v1.9.0) after quality-controlled read processing, generating both raw read count matrices and TPM-normalized expression values. Raw read counts were used for differential expression analysis, whereas TPM values were used for visualization and expression pattern analyses. Functional annotation of the non-redundant protein set was performed by alignment against the KEGG (2025) and GO (202503) databases using DIAMOND (v2.0.3; coverage ≥ 30%) (Buchfink et al., 2015). The annotation rates for KEGG and GO databases were 28.61% and 39.78%, respectively. Carbohydrate-active enzymes (CAZymes) were annotated by alignment of predicted proteins against the CAZy database using BLASTp with a minimum alignment coverage of 30% (Drula et al., 2021) and with an annotation rate of 13.80%.

Antibiotic resistance genes (ARGs) were identified by aligning predicted proteins to the Comprehensive Antibiotic Resistance Database (CARD) (v4.0.1) using ABRicate (v1.0.1) (Seemann, 2020), retaining hits with alignment coverage ≥ 50% and nucleotide identity ≥ 75%. ARG profiles were summarized at both individual gene and drug-class levels. Beta diversity of resistome profiles was assessed using Bray–Curtis-based PCoA and tested with PERMANOVA. Differentially abundant ARGs between groups were identified using LEfSe (Segata et al., 2011).

### 2.5 Statistical analyses

Statistical analyses were performed in R (v4.2.2) and Python (v3.8.11). Data on fermentation parameters and chemical composition between treatments and ensiling time points were analyzed using two-way analysis of variance (ANOVA) with Tukey’s honestly significant difference (HSD) *post-hoc* test. Alpha diversity indices were compared using ANOVA, whereas beta diversity based on Bray-Curtis dissimilarity was assessed by principal coordinate analysis (PCoA) and statistically evaluated using permutational multivariate analysis of variance (PERMANOVA). Differentially abundant taxa and ARGs were identified using LEfSe. Differential gene expression analysis was performed using pyDESeq2 (v0.5.1) based on raw read count matrices generated by Salmon. Genes were considered significantly differentially expressed when |log_2_FC| ≥ 1.5 and adjusted *P*-value (FDR) < 0.05. TPM values of the identified differential genes were subsequently extracted for heatmap visualization. Differential expression was evaluated using treatment comparisons between LP-inoculated and control silage at each fermentation stage (day 3 and day 90). Genes and CAZyme families were considered significantly different in abundance at |log_2_FC| ≥ 1.5 and FDR < 0.05. PERMANOVA was used to test significant differences in beta diversity of bacterial communities and resistome profiles. LEfSe analysis was performed with a default LDA score threshold of >5.0 for taxa and >4.0 for ARGs. All multiple comparisons were adjusted using Tukey’s HSD test where appropriate.

## 3 Results

### 3.1 Fermentation quality and chemical composition

As shown in Table 1, pH was significantly influenced by ensiling time (*P* < 0.001), treatment (*P* < 0.001), and their interaction (*P* = 0.007). The pH decreased from day 3 to day 90 in both treatments, with LP-treated silage exhibiting lower values than the control at both day 3 (3.92 vs. 4.02) and day 90 (3.45 vs. 3.63). Dry matter was not affected by ensiling time, treatment, or their interaction. Lactic acid (LA) concentration increased significantly with ensiling time (*P* < 0.001), but was not affected by treatment or the interaction, with similar values in control and LP-treated silage at both day 3 (26.3 vs. 27.2 g/kg DM) and day 90 (36.8 vs. 37.4 g/kg DM). Acetic acid (AA) was affected by ensiling time (*P* = 0.019), treatment (*P* = 0.015), and their interaction (*P* = 0.028). Acetic acid increased from day 3 to day 90 in both treatments, with higher values in the control than in LP-treated silage at day 90 (6.96 vs. 2.83 g/kg DM). The LA/AA ratio showed a significant interaction effect (*P* < 0.001), decreasing from day 3 to day 90 in control silage (14.01–5.92) but increasing in LP-treated silage (6.28–13.16). The opposite trends in LA/AA ratio between treatments (increasing in LP but decreasing in control) show that *L. plantarum* inoculation shifted the fermentation toward a more homolactic pathway at the later ensiling stage. Propionic acid was not affected by ensiling time, treatment, or their interaction. Butyric acid increased significantly with ensiling time (*P* < 0.001) but was not affected by treatment or interaction. WSC was significantly affected by ensiling time, treatment, and their interaction (*P* < 0.001). LP-treated silage showed higher WSC than the control at both day 3 (225 vs. 151 g/kg DM) and day 90 (224 vs. 197 g/kg DM), whereas WSC increased from day 3 to day 90 in control silage. NH_3_-N was affected by ensiling time (*P* = 0.007) and treatment (*P* = 0.007), but not by their interaction. At day 3, LP-treated silage showed higher NH_3_-N than the control (269 vs. 211 g/kg TN), whereas no significant difference between treatments was observed at day 90 (211 vs. 190 g/kg TN). Crude protein was not affected by ensiling time, treatment, or their interaction.

**TABLE 1:** Fermentation characteristics and chemical composition of control and LP-treated silage during ensiling.

Parameter	Ensiling day	Control	LP	SEM	P-value		
					Day (D)	Treatment (T)	TxD
pH	3	4.02^a^	3.92^b^	0.012	<0.001	<0.001	0.007
	90	3.63^c^	3.45^d^				
DM	3	372^a^	351^a^	6.85	0.091	0.368	0.075
	90	371^a^	378^a^				
LA g/kg DM	3	26.3^b^	27.2^b^	1.26	<0.001	0.585	0.880
	90	36.8^a^	37.4^a^				
AA g/kg DM	3	2.96^b^	2.66^b^	0.715	0.019	0.015	0.028
	90	6.96^a^	2.83^b^				
LA/AA	3	14.01^a^	6.28^b^	1.13	0.608	0.837	<0.001
	90	5.92^b^	13.16^a^				
PA g/kg DM	3	2.93^a^	2.94^a^	0.404	0.368	0.990	0.997
	90	3.32^a^	3.33^a^				
BA g/kg DM	3	1.45^b^	1.01^b^	0.330	<0.001	0.638	0.429
	90	2.99^a^	3.10^a^				
WSC g/kg DM	3	151^c^	225^a^	3.44	<0.001	<0.001	<0.001
	90	197^b^	224^a^				
NH_3_-N g/kg TN	3	211^b^	269^a^	10.8	0.007	0.007	0.126
	90	190^b^	211^b^				
CP g/kg DM	3	69.8^a^	70.2^a^	2.47	0.448	0.793	0.662
	90	72.9^a^	71.1^a^				

Values are presented as mean ± SEM (*n* = 3). CK, control; LP, *L. plantarum*-inoculated treatment; 3 and 90 days represent ensiling duration. SEM represents the pooled standard error of the mean obtained from two-way ANOVA. Different lowercase letters (a–c) indicate significant differences among treatments across ensiling time based on Tukey’s multiple comparison test (*P* < 0.05). *P*-values for the effects of ensiling time (day), treatment, and their interaction (day × treatment) were determined using two-way ANOVA. DM, dry matter; LA, lactic acid; AA, acetic acid; PA, propionic acid; BA, butyric acid; WSC, water-soluble carbohydrates; NH_3_-N, ammonia nitrogen; CP, crude protein.

### 3.2 Microbial community diversity and composition

**FIGURE 1:**
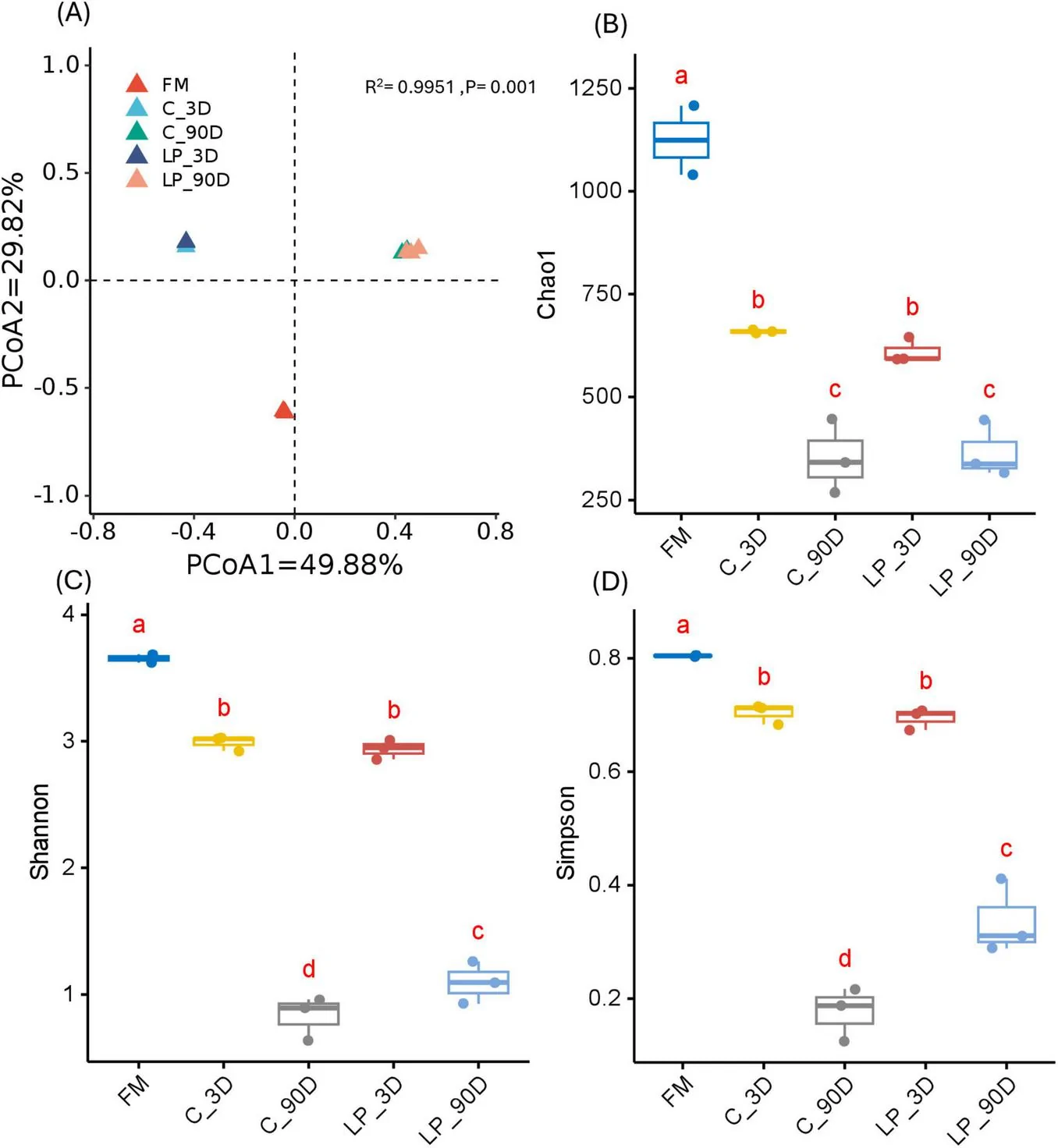
Alpha and beta diversity dynamics of bacterial communities during ensiling. **(A)** Principal coordinate analysis (PCoA) of bacterial community composition based on Bray–Curtis dissimilarity. Fresh material (FM), control (C), and *L. plantarum*-inoculated (LP) silage samples at day 3 and day 90 are shown. PERMANOVA test results: R^2^ = 0.9951, *P* = 0.001. **(B–D)** Alpha diversity indices including Chao1 richness **(B)**, Shannon diversity **(C)**, and Simpson diversity **(D)**. Different lowercase letters above boxes indicate significant differences among groups (Tukey’s HSD test, *P* < 0.05).

To determine the impact of inoculation on the overall microbial community structure, the alpha and beta diversity of the silage microbiota were analyzed. Beta diversity based on Bray-Curtis dissimilarity revealed that community composition was mainly driven by ensiling time, as shown by principal coordinates analysis (PCoA; PCoA1 = 49.88%; PCoA2 = 29.82%; Figure 1A). Fresh material samples were clearly separated from other treatments along PCoA2 (mean PCoA2 = −0.612). PERMANOVA analysis showed significant differences among groups (R^2^ = 0.9951, *P* = 0.001). Pairwise PERMANOVA comparisons between LP-treated and control silages at individual fermentation stages showed no significant differences after FDR correction (day 3: R^2^ = 0.2775, *P* = 0.30, FDR = 0.36; day 90: R^2^ = 0.9883, *P* = 0.10, FDR = 0.36). PERMDISP analysis showed no significant differences in within-group dispersion between treatments (*P* > 0.05). At day 3, LP-inoculated and control communities showed near-complete positional overlap on both axes (mean PCoA1: C_3D = −0.432, LP_3D = −0.432; mean PCoA2: C_3D = +0.163, LP_3D = +0.179). By day 90, these communities showed different ordination positions along PCoA1 (mean PCoA1: C_90D = +0.438, LP_90D = +0.467), with differences in replicate distributions between the two groups (C_90D range: +0.427 to +0.447; LP_90D range: +0.444 to +0.493; Figure 1A). At day 3, LP inoculation had no significant effect on any alpha diversity indices, with Chao1 (control: 659; LP: 610), Shannon (control: 2.990; LP: 2.937), and Simpson (control: 0.704; LP: 0.695) indices assigned to the same statistical group (*P* > 0.05; Figures 1B–D). By day 90, species richness (Chao1) remained comparable between control and LP-treated silage (control: 352; LP: 366; *P* > 0.05); however, Shannon (control: 0.830; LP: 1.095) and Simpson (control: 0.176; LP: 0.337) indices were significantly higher in LP-treated silage than in control (*P* < 0.05; Figures 1B–D).

**FIGURE 2:**
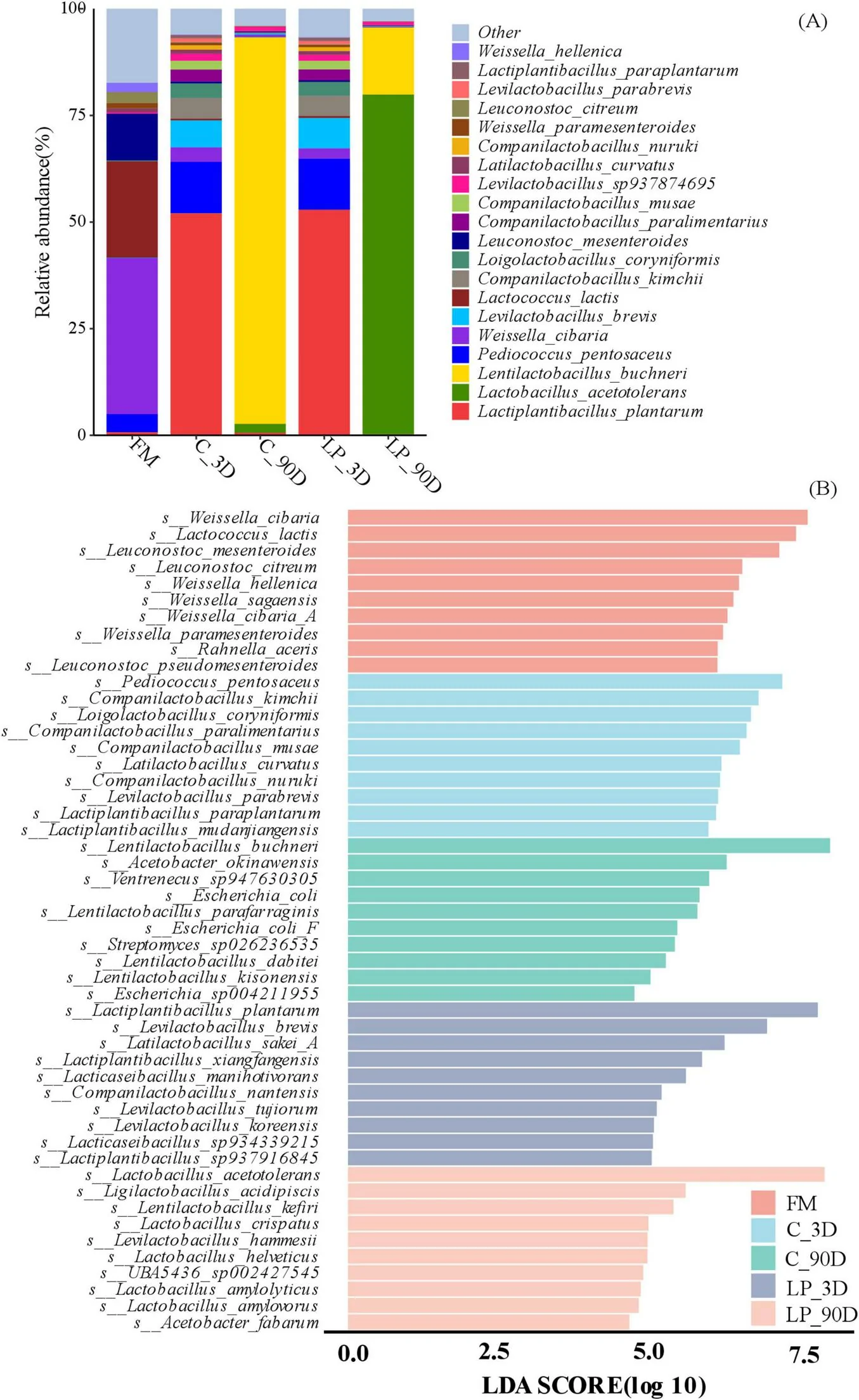
Bacterial community composition and LEfSe biomarker analysis at the species level. **(A)** Relative abundance of dominant bacterial species in fresh material (FM), control (C), and *L. plantarum*-inoculated (LP) silage at day 3 and day 90. **(B)** LEfSe analysis identifying treatment- and stage-specific biomarker taxa (LDA score > 5.0). Color bars indicate the fermentation stage or treatment with the highest abundance for each biomarker taxon.

To characterize the taxonomic basis of these community-level shifts, species-level composition was examined across treatments and ensiling time points (Figure 2A). Fresh material was dominated by *Weissella cibaria* (36.6%), *Lactococcus lactis* (22.6%), and *Leuconostoc mesenteroides* (11%), all of which declined to negligible abundance in both treatments by day 3. At day 3, both treatments converged on a similar community composition dominated by *L. plantarum* (control: 52%; LP: 52.9%), *Pediococcus pentosaceus* (control: 12.1%; LP: 12%), and *Levilactobacillus brevis* (control: 6.3%; LP: 7.1%). By day 90, differences in dominant taxa were observed between LP-inoculated silage and control silage. The control silage was dominated by *Lentilactobacillus buchneri* (90.7%), which corresponds to the marked accumulation of acetic acid in control silage at day 90 (6.96 g/kg DM) and the concurrent decline in the LA/AA ratio from 14.01 to 5.92 (Table 1), whereas LP-treated silage was dominated by *Lactobacillus acetotolerans* (79.7%), with *L. buchneri* remaining a minor community member in the LP treatment (15.8%; Figure 2A). LEfSe analysis identified stage- and treatment-specific biomarker taxa across fermentation stages (Figure 2B). Fresh material was characterized by *W. cibaria* and *L. lactis*, while *P. pentosaceus* was enriched in control silage at day 3 and *L. plantarum* in LP-treated silage at the same stage. By day 90, *L. buchneri* and *L. acetotolerans* emerged as the primary discriminating taxa for control and LP silages, respectively.

### 3.3 Functional pathway enrichment

To evaluate the effect of LP inoculation on the functional potential of the silage microbiota over the ensiling period, Gene Ontology (GO) and KEGG pathway analyses were performed on metatranscriptomic profiles from both treatments at day 3 and day 90. Global functional profiling (Supplementary Table 1) revealed a shared core of broad biological processes across all groups, including metabolic activity, catalytic functions, and transporter activity. At the GO level, functional categories related to microbial community organization and cellular interactions, including cell aggregation, supramolecular fiber assembly, and biofilm formation, were detected in both control and LP-treated silages at day 90 (Supplementary Table 1). In contrast, GO terms associated with cell killing were below the detection threshold in both treatments at day 90.

**FIGURE 3:**
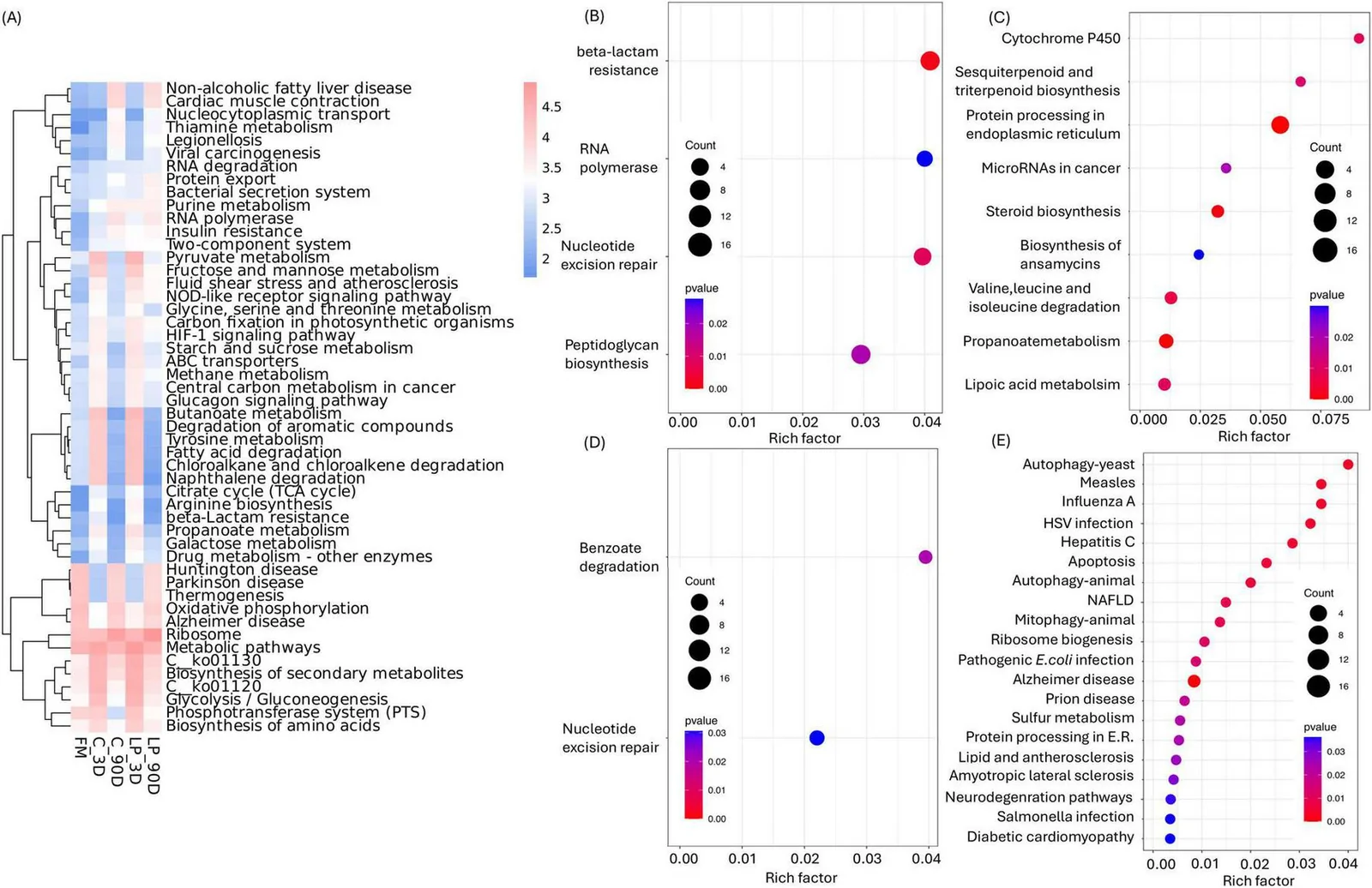
Differential KEGG pathway enrichment between *L. plantarum*-inoculated (LP) and control (C) silage. **(A)** Overview of KEGG pathway expression profiles across fresh material (FM), control, and LP-treated silage at day 3 and day 90. **(B–E)** Volcano plots showing significantly enriched or depleted KEGG pathways in LP versus control silage at **[(B)** upregulated; **(C)** downregulated] day 3 and [**(D)** upregulated; **(E)** downregulated] day 90. Pathways with adjusted *P* < 0.05 are highlighted. Rich factor indicates the proportion of differentially expressed genes within each pathway.

KEGG-based analyses (Figure 3A) revealed that LP-inoculated silage exhibited higher expression of carbohydrate metabolism pathways than the control at day 90, including glycolysis/gluconeogenesis, starch and sucrose metabolism, and the phosphotransferase system (PTS), consistent with the greater preservation of water-soluble carbohydrates observed in this treatment. In contrast, most other KEGG functional categories showed comparable expression patterns between treatments at this stage. Pathway enrichment analysis across time (Figures 3B–E) further resolved early and late responses to inoculation: at day 3, LP-inoculated silage exhibited significant upregulation of peptidoglycan biosynthesis and β-lactam resistance pathways, accompanied by significant suppression of propionate metabolism and complex biosynthetic pathways including protein processing in the endoplasmic reticulum. By day 90, the control silage was characterized by marked enrichment of stress-response and degradation-related pathways, including autophagy and apoptosis (*P* < 0.05), whereas these pathways remained comparatively less expressed in LP-inoculated silage.

### 3.4 Carbon metabolism and related functional gene expression

Genome-wide differential expression patterns revealed a strong bias toward upregulation in LP-treated silage, with 1,024 upregulated versus 82 downregulated genes at day 3, and 665 upregulated versus 72 downregulated genes at day 90 (Supplementary Tables 2, 3). There was a persistent asymmetry between upregulated and downregulated genes across both time points (12.5:1 at day 3; 9.2:1 at day 90). Subsequent analyses focused on functional gene categories contributing to this transcriptional divergence.

**FIGURE 4:**
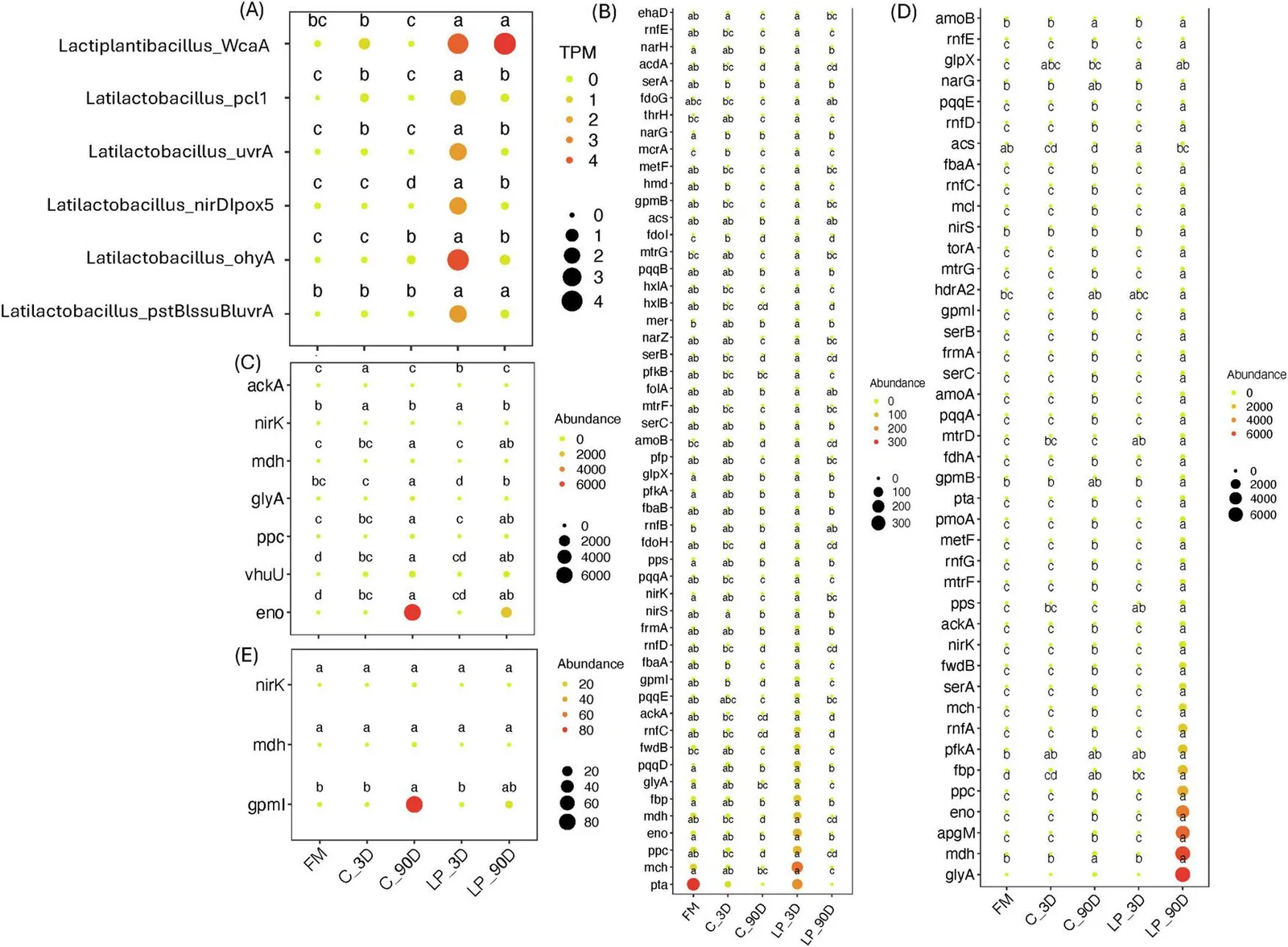
Differential expression of central carbon and associated functional genes in response to *L. plantarum* inoculation. Bubble plots showing TPM-normalized transcript abundance of selected genes involved in central carbon metabolism and related pathways. **(A)** Representative genes with higher expression in *L. plantarum*-inoculated (LP) silage across fermentation stages. **(B)** Genes significantly upregulated in LP versus control at day 3. **(C)** Genes significantly downregulated in LP versus control at day 3. **(D)** Genes significantly upregulated in LP versus control at day 90. **(E)** Genes significantly downregulated in LP versus control at day 90. Bubble size represents mean transcript abundance (TPM), and color intensity indicates higher expression levels. Different lowercase letters above bubbles denote significant differences among groups (Tukey’s HSD test, *P* < 0.05). FM, fresh material.

To assess the effect of LP inoculation on selected functional gene expression in silage, TPM-normalized transcript abundances were compared between treatments at day 3 and day 90 (Figure 4A). Genes related to stress tolerance and cell surface biosynthesis, including *pcl1, uvrA, ohyA*, and *nirD—pox5*, showed the highest transcript levels in LP-treated silage at day 3 and remained generally higher than in the control at day 90 (*P* < 0.05). Transcript abundance of these genes declined at day 90 in LP-inoculated silage, although levels generally remained above those detected in control silage.

Differential expression analyses were then performed separately at each ensiling time based on LP versus control contrasts, and genes were classified as upregulated (UP) or downregulated (DOWN) in LP-treated silage relative to the control. At day 3, 52 genes were UP and seven genes were DOWN in LP-treated silage (Figures 4B,C and Supplementary Table 4). Upregulated genes included those involved in central carbon metabolism (eno, ppc, mdh, fbp), as well as related pathways such as amino acid biosynthesis (serA, serB, serC, glyA), acetate formation (ackA, pta), and nitrogen and redox metabolism (nirK, nirS, narG, narH, narZ, rnfC, rnfD, rnfE). Several key central carbon metabolism genes, including ackA, fbp, eno, and pfkA, exhibited fold changes exceeding 30-fold at day 3 (Supplementary Table 4). By day 90, LP-treated silage still differed transcriptionally from the control, with 44 genes UP and three genes DOWN (Figures 4D,E and Supplementary Table 4). Elevated transcription of central carbon metabolism, amino acid biosynthesis, acetate formation, and nitrogen/redox-associated pathways persisted in LP-treated silage, whereas expression of these pathways was lower in the control. The mdh, nirK, and gpmI genes were upregulated in the LP group at day 3 but showed reduced expression at day 90 in LP-versus-control comparisons (Figures 4B,E and Supplementary Table 4). Despite these contrasting patterns, at day 90 mdh, nirK, and gpmI all had higher mean TPM values in the control than in LP-treated silage (Supplementary Table 4). Notably, several key genes in glycolysis and acetate formation, including ackA, fbp, eno, and pfkA, exhibited dramatic upregulation (>30-fold) in LP-treated silage at day 3, showing rapid induction of central carbon metabolism-related gene expression shortly after inoculation.

Consistent with the functional pathway patterns observed in LP-treated silage, no statistically significant temporal differences in CAZyme family abundance were detected between day 3 and day 90 in the LP treatment, whereas several CAZyme families showed significant temporal differences in the control group (Supplementary Table 5). In the control group, 56.4% of CAZyme families showed significant differential abundance between day 3 and day 90 (FDR < 0.05), whereas no CAZyme families differed significantly between LP-treated and control silages at either time point. In this context, CAZyme profiles reflect microbial carbohydrate utilization potential during fermentation rather than direct lignocellulose degradation, as silage fermentation is primarily driven by soluble carbohydrate metabolism.

### 3.5 Nitrogen metabolism gene expression

**FIGURE 5:**
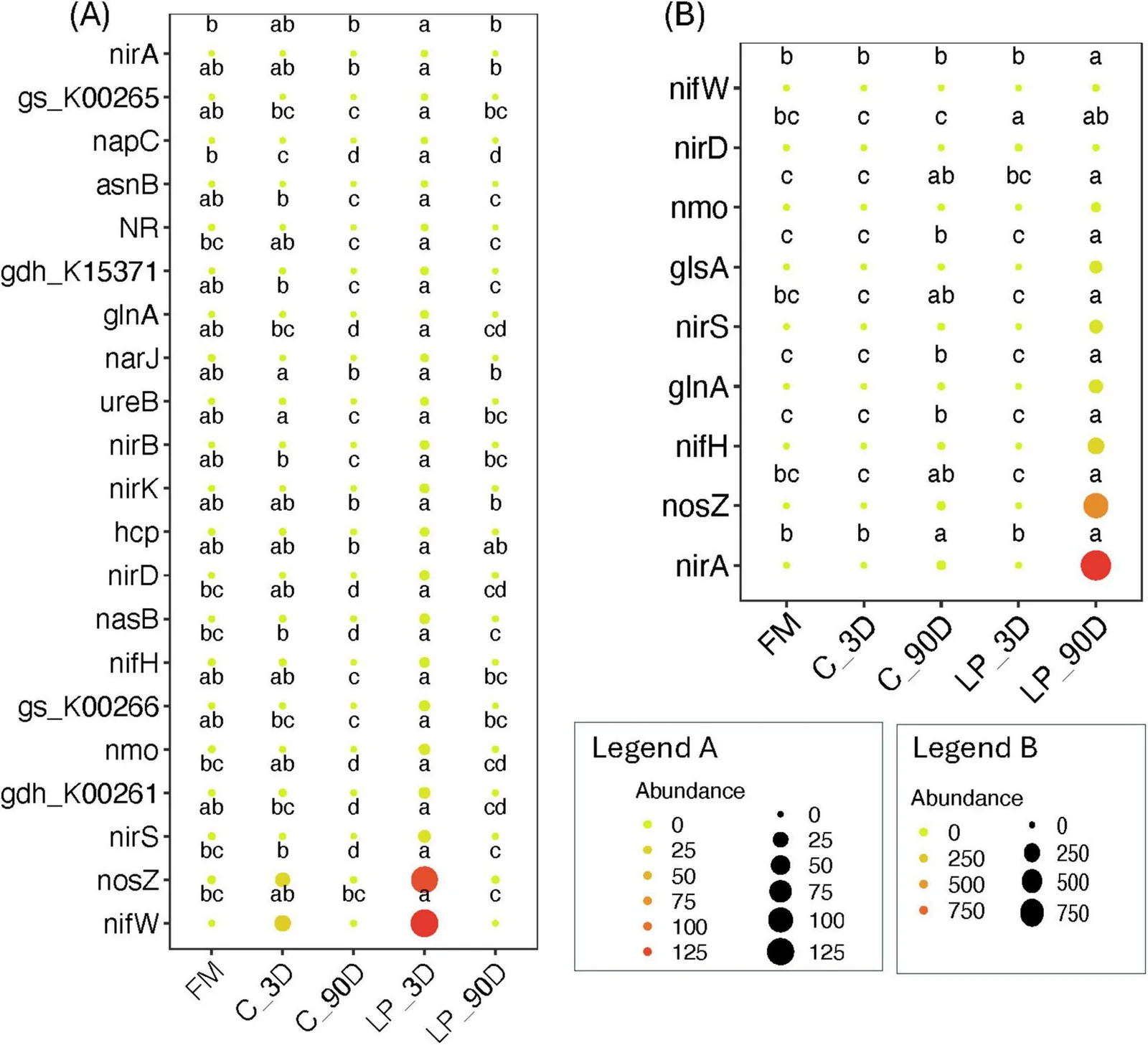
Transcriptional profiles of nitrogen metabolism-related genes. Bubble plots showing TPM-normalized transcript abundance of nitrogen-associated genes identified as significantly upregulated in LP-treated silage relative to control. **(A)** Genes significantly upregulated in LP versus control at day 3. **(B)** Genes significantly upregulated in LP versus control at day 90. Bubble size and color represent mean transcript abundance (TPM). Different lowercase letters above bubbles indicate significant differences among groups (Tukey’s HSD test, *P* < 0.05). FM, fresh material.

Transcript abundances of nitrogen-cycling genes differed between LP-treated and control silages at both time points (Figure 5 and Supplementary Table 6). At day 3, LP-treated silage showed broadly elevated expression across multiple nitrogen transformation pathways relative to the control (Figure 5A). Denitrification genes, including *nirK, nirS*, and *nosZ*, as well as DNRA- and nitrate reduction-associated genes (*nirD, nirB, narJ, napC*), showed substantially higher expression in LP-treated silage compared to the control at day 3, whereas these differences were less pronounced at day 90 (Supplementary Table 4). In parallel, genes involved in nitrogen acquisition and assimilation, including *nifH, nifW, glnA, gdh*, and the organic nitrogen processing gene *nmo*, showed higher expression in LP-treated silage at day 3 (Supplementary Table 4).

By day 90, treatment differences were concentrated in a smaller subset of nitrogen metabolism genes (Figure 5B). Ferredoxin-dependent nitrite reductase *nirA* and glutaminase *glsA* showed markedly higher expression in LP-treated silage compared to the control at day 90, whereas most denitrification and DNRA genes that differed at day 3 showed reduced treatment contrasts at day 90, although *nosZ* and *nirS* continued to exhibit substantially higher expression in LP-treated silage (Supplementary Table 4).

### 3.6 Antibiotic resistance gene (ARG) profiling

**FIGURE 6:**
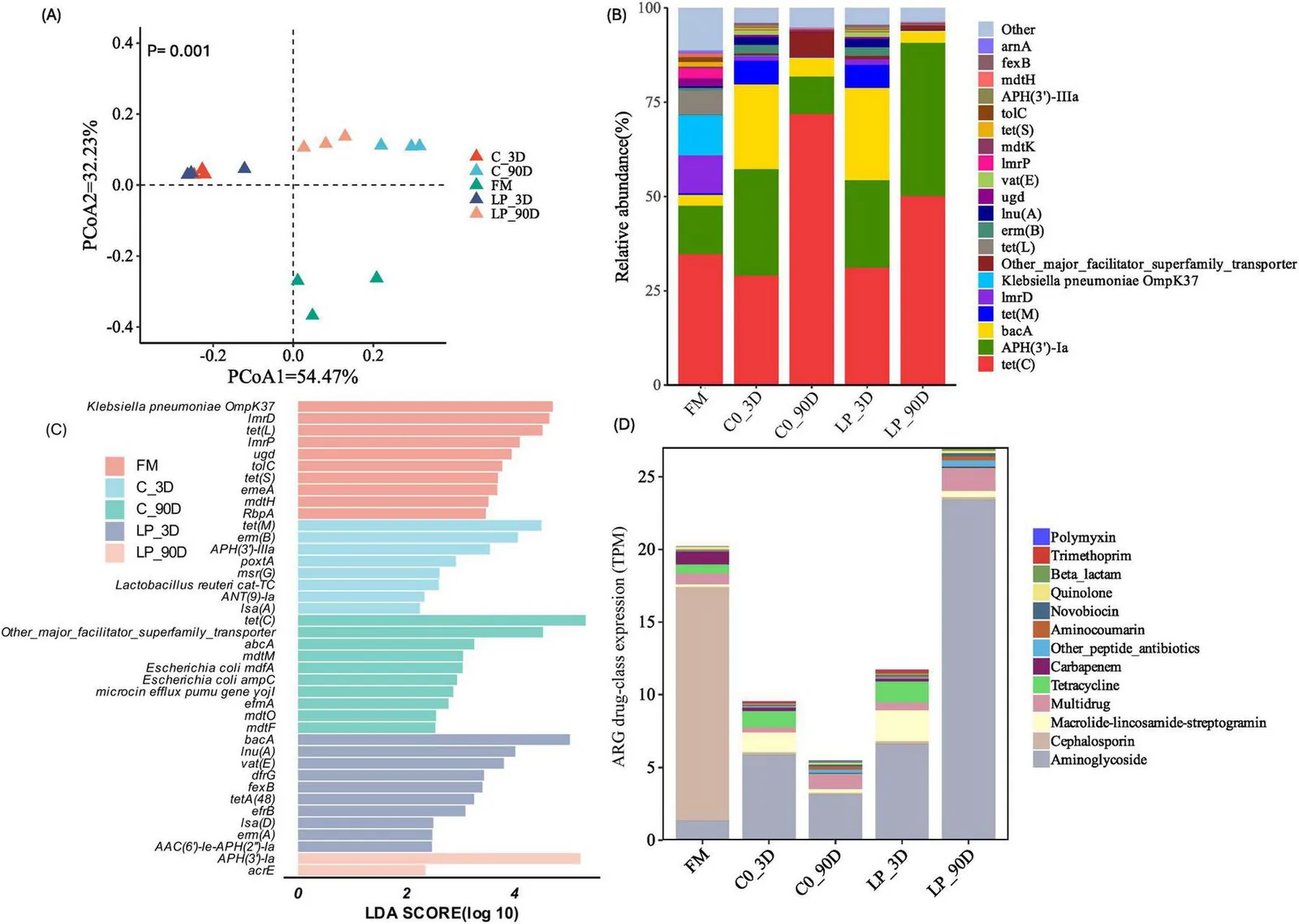
Antibiotic resistance gene (ARG) profiles during ensiling. **(A)** Principal coordinate analysis (PCoA) of resistome composition based on Bray–Curtis dissimilarity. PERMANOVA test: R^2^ = 0.9051, *P* = 0.001. **(B)** Relative abundance of dominant ARGs (top 20) across treatments and timepoints. **(C)** LEfSe analysis identifying stage- and treatment-specific ARG biomarkers (LDA score > 4.0). **(D)** ARG drug-class expression (TPM) across fresh material (FM), control (C), and LP-treated silage. Stacked bars represent the relative contribution of each antibiotic class.

Resistome composition patterns were mainly organized by ensiling time rather than LP inoculation. At day 3, principal coordinate analyses (PCoA) (R^2^ = 0.9051, *P* = 0.001) showed considerable overlap between LP-treated and control silages, whereas at day 90 both treatments shifted to a separate ordination space (Figure 6A). Relative abundance profiles of expressed ARGs revealed that the resistome was dominated by *tet(C), APH (3’)-Ia, bacA* and *tet(M*) in corn silage (Figure 6B). At day 3, *tet(C), APH (3’)-Ia*, and *bacA* contributed similar proportions in both LP-treated and control treatments: in control silage, 29.04%, 28.20%, 22.46%; and in LP-treated silage: 31.15%, 23.23%, 24.44%, respectively. *tet(M)* was present at lower but comparable levels (control: 6.31%; LP: 6.13%). By day 90, *tet(C)* and *APH (3’)-Ia* became strongly dominant in both treatments but with distinct distributions: in control silage, *tet(C)* increased to 71.87% and *APH (3’)-Ia* declined to 9.97%, whereas in LP-treated silage *tet(C)* reached 50.20% and *APH (3’)-Ia* reached 40.54%; *bacA* and *tet(M)* decreased to low relative abundances in both groups (control: 5.00% and 0.21%; LP: 3.25% and 0.11%, respectively). ARGs associated with macrolide–lincosamide–streptogramin resistance [*erm(B), lnu(A), vat(E)*] and multidrug efflux-related transporters (including *lmrD*, *tolC, mdtK*, and *mdtH*) remained at consistently low and comparable levels between treatments but showed a general decrease over the course of ensiling. Consistent with these abundance patterns, LEfSe analysis (Figure 6C) identified group-specific ARG biomarkers. Across all samples, the overall ARG repertoire was largely conserved, with no ARG subtypes uniquely detected in LP-treated silage or consistently absent from the control. At day 90, control silage was characterized by high LDA scores for *tet(C)* and several multidrug/β-lactam efflux transporters. In contrast to the control, LP-treated silage showed enrichment of subsets of core ARGs including *bacA, lnu(A)*, and *vat(E)* at day 3, and *APH (3’)-Ia* and *acrE* at day 90.

Metatranscriptomic profiling of ARGs at the CARD drug-class level further revealed treatment and time-dependent patterns in resistance gene expression (Figure 6D). Class-level transcriptional profiles did not directly mirror patterns observed at the individual ARG level (Figure 6B). While *tet(C)* dominated at day 90, the tetracycline antibiotic class as a whole showed relatively lower expression. In contrast, aminoglycoside resistance genes showed higher expression, dominating at the class level. This gene class displayed similar abundance profiles between the LP-treated and control silages at day 3, whereas by day 90 LP-treated silage not only demonstrated higher expression of aminoglycoside genes but also of multidrug efflux and peptide antibiotic classes. Conversely, control silage showed lower and a more evenly distributed ARG expression across the drug classes. Despite these statistical differences in abundance patterns, the collection of antibiotic drug classes that were detected remained consistent across treatments and ensiling times. No novel resistance classes were detected during fermentation. Collectively, these results demonstrate that the core resistome structure detected at the transcriptional level was primarily shaped by ensiling time rather than *L. plantarum* inoculation, with no newly detected ARG subtypes or overall expansion of the active resistome under the tested conditions.

## 4 Discussion

*L. plantarum* inoculation altered the fermentation process of corn silage, as evidenced by lower pH, reduced acetic acid accumulation, and a higher LA/AA ratio at day 90 of ensiling (Table 1). Even though the lactic acid concentrations increased similarly in both treatments, the difference in the LA/AA ratios suggests a change in balance between homolactic and heterofermentative fermentation pathways (Muck et al., 2018). The higher acetic acid concentrations observed in control silage suggest an increase in heterofermentative metabolism at day 90, whereas LP-treated silage maintained comparatively lower acetic acid levels, compatible with homolactic fermentation (Kleinschmit and Kung, 2006). These differences in the end products may result from changes in metabolic activity during ensiling, consistent with the observed changes in the microbial community composition. NH_3_-N concentrations at day 3 in LP-treated silage were higher than in control silage, although this difference was not maintained at day 90. The overall NH_3_-N levels observed in this study were comparably high, indicating significant proteolysis during ensiling (Kung et al., 2018). This may be linked to the active nitrogen transformation observed during early fermentation, potentially involving microbial nitrogen turnover in addition to proteolytic activity. Butyric acid concentration increased from day 3 to day 90 in both treatments, indicating that these changes were driven largely by ensiling time rather than the inoculation. Dry matter and crude protein levels remained stable in both treatments across ensiling times, consistent with the lack of notable treatment effects. An increase in WSC was observed in control silage over time, which is atypical. Due to microbial utilization, WSC is commonly expected to decrease during the course of fermentation (Guo et al., 2023). Differences in microbial utilization of soluble carbohydrates between control and LP-treated silage may contribute to this pattern. In addition, the release of soluble sugars from structural carbohydrates during prolonged fermentation may also influence WSC dynamics (Wilkinson and Rinne, 2018).

In accordance with these fermentation changes, the microbial community also underwent substantial dynamics. *W. cibaria*, *L. lactis* and *Leuconostoc* species initially dominated fresh material but declined rapidly by day 3. A strong selective pressure imposed by anaerobic and acidic conditions during early ensiling is the probable reason. Similar community compositions were observed between LP-treated and control silage at day 3. Both were dominated by *L. plantarum*, *P. pentosaceus*, and *L. brevis*, with no significant differences observed in alpha diversity (Figures 1B–D). Together with the corresponding fermentation values at day 3 (Table 1), this similarity suggests that an early community structure was predominantly shaped by rapid acidification along with the suppression of non-acid taxa. However, clear differences in dominant taxa emerged by day 90 (Guan et al., 2020; Sun et al., 2021). Although pairwise PERMANOVA comparisons showed no statistically significant differences between LP-treated and control silages at either day 3 or day 90 after FDR correction, control silage was dominated by *L. buchneri*, whereas LP-treated silage was dominated by *L. acetotolerans* (Figure 2A). The dominance of *L. buchneri* in control silage explains the increased acetic acid production and reduced LA/AA ratio observed at day 90 (Table 1), as this species is known to convert lactic acid into acetic acid via heterofermentative pathways (Kleinschmit and Kung, 2006). In contrast, the increased presence of *L. acetotolerans* in LP-treated silage suggests that the inoculation was associated with a specialized fermentation environment. Low pH, reduced acetic acid accumulation, and higher WSC availability all might have favored acid-tolerant *Lactobacillus* species at the later fermentation stage. Increased Shannon and Simpson indices in LP-treated silage at day 90 indicated a more complex late-stage community structure and higher microbial diversity. LEfSe analysis supported these observations by highlighting the dominance of *L. plantarum* in LP-treated silage and *P. pentosaceus* in control silage at day 3. This was accompanied by differences in dominant taxa, with *L. buchneri* and *L. acetotolerans* becoming dominant in control and LP-treated silage, respectively, at day 90. The reduced dominance of *L. plantarum* during the late stage can be attributed to ecological succession driven by substrate depletion and increased acidity, which favors acid-tolerant and metabolically adapted taxa over fast-growing early-stage colonizers. These findings are consistent with previous studies showing that fermentation conditions strongly influence microbial succession and the eventual dominance of specific taxa (Bai et al., 2021; Sun et al., 2021). Since pairwise PERMANOVA comparisons showed no statistically significant differences between LP-treated and control silages at either day 3 or day 90 after FDR correction, the observed differences in dominant taxa could be interpreted as taxonomic variation rather than statistically significant community-level divergence at individual time points.

Furthermore, functional gene expression patterns were consistent with these differences in dominant taxa. The higher proportion of upregulated genes in LP-treated silage at both evaluated time points (12.5:1 at day 3; 9.2:1 at day 90; Supplementary Tables 2, 3) suggests consistent transcription under LP inoculation. The enrichment of carbohydrate metabolism pathways, including glycolysis, the phosphotransferase system (Deutscher et al., 2006), and starch and sucrose metabolism, in LP-treated silage at day 90 (Figure 3A) is consistent with greater WSC preservation observed in this treatment (Table 1), indicating enhanced transcription of carbohydrate metabolism-related genes under acidic conditions. In addition, no statistically significant temporal differences in CAZyme family abundance were detected in LP-treated silage across ensiling times (Supplementary Table 5), whereas 56.4% of CAZyme families showed significant temporal differences in control silage, and no such differences were detected in LP-treated silage (Lombard et al., 2014; Li et al., 2024). However, this result should be interpreted cautiously considering the limited number of biological replicates. In contrast, the functional shifts in control silage likely reflect metabolic changes associated with the transition to *L. buchneri* dominance and the related changes in fermentation end products. The upregulation of pathways related to peptidoglycan biosynthesis and β-lactam resistance at day 3 in LP-inoculated silage likely reflects the active cell growth and adaptation to acidic conditions during early fermentation, rather than the induction of different resistance mechanisms. The suppression of propionate metabolism pathways in LP-inoculated silage at day 3 may reflect a shift toward homolactic fermentation, with reduced contribution from alternative fermentation pathways associated with secondary end products. Despite these differences, most functional categories remained somewhat similar between treatments at day 90. These results indicate that LP effects were pathway specific, rather than global. The increase in stress and degradation-related pathways, including autophagy and apoptosis (terms likely reflecting conserved stress-related functional annotations rather than specific bacterial pathway activity), in control silage could suggest a microbial community shaped by stress adaptation. Another possibility would be a turnover related to the dominance of *L. buchneri.* The appearance of GO terms related to cell aggregation and biofilm formation at day 90 in both treatments implies increased microbial interactions under stabilized fermentation. As the community becomes more stabilized, the disappearance of cell killing-related functions suggests reduced competitive or adverse interactions. These pathway differences were also supported by gene-level transcriptional patterns. LP inoculation caused a prominent imbalance, with considerably higher numbers of upregulated than downregulated genes at both time points. This suggests that increased metabolic gene expression is more probable than simple redistribution of existing expression. Additionally, the upregulation of stress tolerance and cell surface-related genes such as *pcl1*, *uvrA*, and *ohyA* in the LP-treated silage suggests a transcriptional response associated with adaptation to acidic and anaerobic conditions. This supports the interpretation of elevated microbial activity during fermentation. For the key carbon metabolism genes including *ackA*, *fbp*, *eno*, and *pfkA*, which exhibited fold changes exceeding 30-fold (Supplementary Table 4), this effect was particularly evident during day 3, reflecting rapid metabolic activation under LP inoculation. Even though these differences were reduced at day 90, the persistence of high gene expression under LP suggests that LP largely accelerates early metabolic activity while maintaining a higher transcriptional activity in the community. The higher expression of central carbon metabolism genes (e.g., *ackA, pfkA, eno*) in LP-treated silage was associated with changes in carbohydrate metabolism, together with reduced acetic acid accumulation and better preservation of water-soluble carbohydrates. These metabolic changes may have contributed to the observed differences in dominant late-stage community members, including the higher relative abundance of *L. acetotolerans*, in LP-treated silage. Furthermore, the coordinated changes in nitrogen metabolism genes (early activation of denitrification-related genes and later higher expression of *nirA*) suggest that *L. plantarum* inoculation influenced nitrogen-related transcriptional patterns during fermentation. Consistent with carbon metabolism patterns, nitrogen cycling genes were also strongly upregulated at day 3 under LP inoculation. LP-treated silage showed higher expression of multiple nitrogen transformation genes, including those involved in denitrification, DNRA, and nitrogen assimilation. These results indicate enhanced expression of genes involved in early nitrogen transformation pathways, which was consistent with the increase in NH_3_-N at day 3 and may reflect altered nitrogen metabolism during early fermentation rather than sustained protein degradation. Nitrate and nitrite serve as key intermediate metabolites linking nitrate reduction, nitrite assimilation, and denitrification pathways, thus representing central nodes in the observed nitrogen metabolic network. By day 90, these overall differences were reduced, and LP-treated silage showed higher expression of genes associated with ferredoxin-dependent nitrite reduction and nitrogen recycling, including *nirA* and *glsA*. While most nitrogen-related genes showed reduced treatment differences at day 90, genes *nosZ* and *nirS* remained more highly expressed under LP inoculation, indicating resilience of specific nitrogen functions. The following shifts indicate a transition from early nitrogen transformation patterns to different nitrogen-related transcriptional responses in LP-treated silage, whereas the control silage maintained lower and more stable nitrogen-related gene expression at both ensiling time points.

Higher gene expression under LP inoculation may also contribute to the treatment-specific patterns in ARG expression, which are linked to metabolic state and dominant taxa. The ARG profiles recorded in this study were driven mainly by ensiling time rather than inoculation (Zhang et al., 2022, 2025). Principal coordinate analyses showed significant overlap between LP-treated and control silages at day 3, whereas both treatments shifted to a distinct ordination space by day 90 (Figure 6A). This indicates that ensiling time was the main factor shaping resistome structure. This pattern clearly matched the microbial community changes observed across time points. Overall stability of ARG classes was observed across all treatments and time points, with no clear evidence of ARG subtypes unique to LP-treated silage. This indicates that LP inoculation was not associated with detectable enrichment of additional ARG subtypes within the detected resistome. This observation aligns with previous studies showing that silage systems usually maintain a stable core resistome composition (Nagy et al., 2022; Zhang et al., 2023). Differences in ARG expression profiles between treatments at day 90 were likely linked to differences in dominant taxa. LP-treated silage showed relatively higher expression of aminoglycoside resistance genes, whereas control silage was characterized by higher relative expression of *tet(C)* (Figures 6B–D). At day 90, LP-treated silage also exhibited higher expression of multidrug efflux and peptide antibiotic resistance classes, likely reflecting activity of the dominant community rather than resistome expansion. ARG biomarkers classified by LEfSe (Figure 6C) aligned closely with the dominant taxa in each treatment, suggesting that changes in ARG expression conformed to shifts in the dominant taxa (Liao et al., 2019). Since metatranscriptomic analysis depicts actively expressed genes, these results reflect active resistome dynamics rather than total genetic potential (Liu et al., 2019). The constant presence of major ARG classes across treatments suggests that LP inoculation changed the expression of resistance genes within an existing resistome rather than altering its overall composition. Several ARGs, including *bacA* and *tet(M)*, declined significantly over time in both treatments. Whereas the macrolide–lincosamide–streptogramin resistance genes and multidrug efflux transporters remained at low and comparable levels, indicating that LP inoculation did not increase the diversity of expressed ARGs under the tested conditions. The clear inconsistency between subtype-level dominance of *tet(C)* and the comparatively lower contribution of tetracycline resistance at the class level likely reflects the extensive diversity and higher total expression of aminoglycoside-associated genes in the metatranscriptome. These observations indicate that in conditions of low antibiotic selection pressure (such as in silage fermentation), resistome dynamics are mainly driven by microbial succession and that inoculant-driven community changes mainly affect transcription patterns without significantly changing the overall resistance profile. Although LP inoculation altered the dominant taxa and certain ARG expression patterns (e.g., higher aminoglycoside class expression at day 90), the overall actively transcribed resistome remained stable in our dataset. No LP-specific ARG subtypes or additional resistance classes were detected under the tested conditions. These findings, derived from metatranscriptomic profiles, should not be interpreted as evidence that ARG acquisition, horizontal gene transfer, or environmental dissemination did not occur; they only indicate that such processes did not manifest as detectable changes in ARG transcription under the conditions and experimental design used here.

In summary, LP inoculation improved corn silage fermentation by promoting a more homolactic profile, characterized by low pH, reduced acetic acid, and higher LA/AA ratios at day 90. These fermentation changes were associated with dominant community taxa, with LP-treated silage showing higher abundance of acid-tolerant taxa, maintaining higher expression of carbohydrate metabolism-related genes compared with the control silage. However, several limitations should be considered when interpreting these findings. This study was conducted using a single corn variety and one *L. plantarum* strain with three biological replicates per treatment at each sampling stage. In addition, the metatranscriptomic findings were not validated using complementary approaches such as RT-qPCR, metabolomics, or enzyme activity assays. Therefore, these findings should be interpreted within the scope of the experimental conditions used in this study. Future studies incorporating additional forage types, inoculant strains, and field-scale ensiling systems will further strengthen these findings and improve our understanding of ARG dynamics in silage ecosystems.

## Data Availability

The data used for this research have been deposited in CNGBdb (CNP0008680 for metatranscriptomics).
