# Supplementary material for: Metatranscriptomic insights into the effect of Lactiplantibacillus plantarum inoculation on fermentation metabolism, bacterial community function and antibiotic resistome of corn silage
Source: Front Microbiol. 2026 Sep 9;17:1876540. doi: 10.3389/fmicb.2026.1876540 (PMC13597671; doi:10.3389/fmicb.2026.1876540)
Supplement: Supplementary file 1 [file Table_1.docx]

| GO Term | Category | FM | C_3D | C_90D | LP_3D | LP_90D | Log₂ fold difference (LP_90D − C_90D) |
| --- | --- | --- | --- | --- | --- | --- | --- |
| Metabolic process | Biological Process | 8.532 | 8.627 | 8.916 | 8.773 | 9.102 | 0.186 |
| Catalytic activity | Molecular Function | 8.372 | 8.421 | 8.530 | 8.581 | 8.704 | 0.174 |
| Response to stimulus | Biological Process | 6.745 | 6.988 | 6.967 | 7.174 | 7.473 | 0.506 |
| Cell killing | Biological Process | 2.880 | 2.105 | -6.000 | 2.696 | -6.000 | -6.000 |
| Cell aggregation | Biological Process | 4.226 | 3.451 | 4.206 | 3.591 | 4.002 | -0.204 |
| Supramolecular fiber | Cellular Component | 2.089 | 2.563 | 4.640 | 2.951 | 4.462 | -0.178 |
| Transporter activity | Molecular Function | 8.469 | 7.806 | 7.874 | 7.986 | 8.059 | 0.185 |
| Signalling | Biological Process | 6.008 | 6.470 | 6.141 | 6.657 | 6.564 | 0.423 |

Table S1. Log₂-transformed TPM values of selected Gene Ontology (GO) terms across silage treatments and fermentation stages.

Note- Log₂-transformed transcript abundance (TPM) for selected Gene Ontology (GO) terms representing metabolic activity and community-associated processes in fresh material (FM), control silage (C_3D, C_90D), and LP-treated silage (LP_3D, LP_90D).

The final column indicates the log₂ fold difference between LP_90D and C_90D.

Values of −6.000 indicate expression below the detection threshold.
