# Supplementary material for: Metatranscriptomic insights into the effect of Lactiplantibacillus plantarum inoculation on fermentation metabolism, bacterial community function and antibiotic resistome of corn silage
Source: Front Microbiol. 2026 Sep 9;17:1876540. doi: 10.3389/fmicb.2026.1876540 (PMC13597671; doi:10.3389/fmicb.2026.1876540)
Supplement: Supplementary file 2 [file Table_2.docx]

Table S2. Differentially expressed genes between LP-inoculated and control silage at day 3

| **Gene** | **C0_3D1** | **C0_3D2** | **C0_3D3** | **LP_3D1** | **LP_3D2** | **LP_3D3** | **baseMean** | **log2FoldChange** | **lfcSE** | **stat** | **pvalue** | **padj** | **-log10padj** | **type** |
| --- | --- | --- | --- | --- | --- | --- | --- | --- | --- | --- | --- | --- | --- | --- |
| **C0_90D1.tig00013.39** | 41 | 127 | 91 | 21 | 25 | 5 | 50.56212011977550 | -2.4128312809404600 | 0.4940075690866330 | -4.884199012176130 | 1.03850145026913E-06 | 0.00010143232807654500 | 3.9938236064657300 | down |
| **LP_3D1.tig00341.3** | 1 | 11 | 18 | 627 | 308 | 119 | 167.68515126267800 | 5.062978420164020 | 1.0106005524976000 | 5.009871019416490 | 5.44665265828671E-07 | 5.58871787653985E-05 | 4.252687813403330 | up |
| **LP_3D1.tig01877.2** | 1 | 2 | 3 | 37 | 22 | 8 | 11.369866866378500 | 3.3866953575422000 | 0.853826137085637 | 3.966492954996670 | 7.29379305108788E-05 | 0.004591535614497130 | 2.338042042705020 | up |
| **LP_3D1.tig06200.1** | 4 | 12 | 7 | 52 | 51 | 40 | 26.68776037698460 | 2.5819647823797300 | 0.511928900780113 | 5.04360034849595 | 4.56853000351901E-07 | 4.73901620529267E-05 | 4.324311806207300 | up |
| **C0_3D2.tig22139.1** | 15 | 28 | 23 | 83 | 262 | 125 | 86.74510984337810 | 2.7873248198088300 | 0.44219408256792000 | 6.303396923862510 | 2.91191892658373E-10 | 4.94113526512394E-08 | 7.306173257000520 | up |
| **LP_3D1.tig02458.2** | 0 | 3 | 5 | 62 | 40 | 24 | 21.076953411748400 | 3.905905120296870 | 0.7100671153268100 | 5.500754838504490 | 3.78168733916636E-08 | 4.63297449989034E-06 | 5.334140090167530 | up |
| **FM1.tig04901.1** | 0 | 0 | 3 | 17 | 16 | 12 | 7.66588763301606 | 3.854740789974120 | 1.079351781474270 | 3.5713479665628500 | 0.000355148682874682 | 0.018122465779184300 | 1.7417827116571400 | up |
| **LP_3D2.tig16979.1** | 0 | 0 | 2 | 71 | 39 | 13 | 19.320068468678000 | 5.840543007491690 | 1.1700862320313800 | 4.991549210310740 | 5.98969176456446E-07 | 6.10186430746714E-05 | 4.2145374543752700 | up |
| **LP_3D1.tig00497.2** | 0 | 4 | 5 | 97 | 80 | 51 | 37.55089000848090 | 4.597746995173700 | 0.6250943997235030 | 7.355284253398220 | 1.90521245235327E-13 | 4.87845954297394E-11 | 10.311717292259800 | up |
| **LP_3D1.tig01972.2** | 9 | 7 | 9 | 159 | 117 | 55 | 56.04489326593430 | 3.6302143094587 | 0.4793079286618460 | 7.573866594683090 | 3.62276443430945E-14 | 1.03485449381066E-11 | 10.985120710088700 | up |
| **LP_3D1.tig00025.2** | 3 | 0 | 2 | 38 | 34 | 7 | 13.097180317643400 | 3.8627929032814600 | 0.9005719893712800 | 4.289266098513910 | 1.7926449209255E-05 | 0.0013285906196872300 | 2.876608818154030 | up |
| **LP_3D1.tig35241.1** | 1 | 7 | 11 | 127 | 85 | 42 | 42.81199457007460 | 3.6671119078335100 | 0.5545576337359820 | 6.612679521024100 | 3.77424974476105E-11 | 7.29752471907966E-09 | 8.136824425241390 | up |
| **C0_90D1.tig00034.7** | 27 | 59 | 22 | 10 | 15 | 5 | 22.733022126907500 | -1.9177275925262400 | 0.5613431358747590 | -3.416319662549700 | 0.000634736894792637 | 0.029770312528455000 | 1.526216606110040 | down |
| **LP_3D1.tig00062.2** | 0 | 1 | 0 | 32 | 18 | 6 | 8.81729525120877 | 5.594280239029850 | 1.5562301901728300 | 3.594763984374690 | 0.0003246857809528410 | 0.01677887565296750 | 1.775237144465120 | up |
| **LP_3D2.tig07083.1** | 2 | 4 | 0 | 76 | 53 | 35 | 26.892192636338900 | 4.688204428512240 | 0.7358479265599850 | 6.371159392171050 | 1.87604542083461E-10 | 3.25133542522389E-08 | 7.487938224041450 | up |
| **C0_3D2.tig13128.1** | 9 | 30 | 37 | 322 | 226 | 80 | 110.06338134349600 | 2.9676684581511900 | 0.4658814479588300 | 6.3700078016702800 | 1.8901858514388E-10 | 3.25888120786568E-08 | 7.486931470068370 | up |
| **LP_3D1.tig06757.2** | 1 | 0 | 0 | 29 | 20 | 11 | 9.59932889173812 | 5.71343579475143 | 1.5246946347969600 | 3.7472656257574400 | 0.00017877274504214500 | 0.01001215437627660 | 1.9994724626746600 | up |
| **LP_3D1.tig00142.4** | 11 | 43 | 40 | 1219 | 737 | 339 | 372.9068958691010 | 4.531930414870870 | 0.4306340907576600 | 10.523854270100500 | 6.7072448178721E-26 | 7.3321794552296E-23 | 22.134766914214200 | up |
| **LP_3D1.tig13680.1** | 19 | 24 | 52 | 833 | 530 | 254 | 268.1154550206840 | 4.0059732686973900 | 0.40896403481801700 | 9.79541702360206 | 1.17810262468726E-22 | 8.5858004744035E-20 | 19.066219208317800 | up |
| **LP_3D2.tig15089.1** | 0 | 0 | 1 | 47 | 16 | 8 | 11.090805665849600 | 5.930543413858250 | 1.5459720127827900 | 3.8361259872894600 | 0.0001249903064845250 | 0.007370408684764350 | 2.1325084301033600 | up |
| **LP_3D2.tig03572.1** | 2 | 0 | 3 | 40 | 44 | 23 | 17.807594226544600 | 4.342316645409310 | 0.8100247270123930 | 5.360721099743500 | 8.28904065299385E-08 | 9.6357978715631E-06 | 5.016112318697090 | up |
| **LP_3D2.tig08826.1** | 14 | 23 | 36 | 196 | 132 | 71 | 74.59160137431270 | 2.3738453410805900 | 0.4159834753043290 | 5.706585674692760 | 1.15264901014487E-08 | 1.57885139714904E-06 | 5.80165874423821 | up |
| **LP_3D1.tig00019.3** | 2 | 1 | 0 | 26 | 12 | 6 | 7.347337688416200 | 3.7561069637101000 | 1.1044524346122400 | 3.400877073559820 | 0.0006717002575652240 | 0.03119509897164640 | 1.5059136321598700 | up |
| **C0_3D3.tig28942.1** | 10 | 11 | 25 | 299 | 181 | 58 | 90.82353005754970 | 3.4489937181640700 | 0.4957199431272240 | 6.957544811302650 | 3.4625383250596E-12 | 7.71874474854953E-10 | 9.11245332053253 | up |
| **LP_3D1.tig02961.1** | 0 | 1 | 1 | 97 | 75 | 54 | 36.17762938875150 | 6.748057755292080 | 1.0919368837328600 | 6.179897259467390 | 6.41433313474223E-10 | 1.03000781650967E-07 | 6.987159479514110 | up |
| **LP_3D1.tig00019.6** | 0 | 1 | 6 | 389 | 167 | 58 | 95.53777090474210 | 6.373823844213400 | 1.2619287272252400 | 5.0508588216613000 | 4.39828062725298E-07 | 4.60442471926695E-05 | 4.336824623198930 | up |
| **FM1.tig04015.1** | 18 | 60 | 53 | 248 | 166 | 66 | 96.21298266643830 | 1.7953373773313900 | 0.4460522548859280 | 4.024948551802590 | 5.69877730749906E-05 | 0.0036896013216945800 | 2.4330205588117400 | up |
| **LP_3D1.tig00918.2** | 0 | 0 | 1 | 27 | 37 | 13 | 12.302706207935100 | 6.078640990484450 | 1.5069506287243200 | 4.033735992817620 | 5.48969936717442E-05 | 0.003566422406023200 | 2.447767220403300 | up |
| **LP_3D1.tig03082.1** | 0 | 0 | 1 | 44 | 23 | 19 | 13.712720705921300 | 6.237015815439560 | 1.4951633608651400 | 4.171461111667880 | 3.02652713666525E-05 | 0.002108369302496770 | 2.6760533156658200 | up |
| **LP_3D2.tig14056.1** | 2 | 1 | 1 | 59 | 58 | 10 | 20.33968848216970 | 4.873829405440040 | 0.9166090334091930 | 5.31723911481927 | 1.05353580525912E-07 | 1.20986349191828E-05 | 4.9172636280728300 | up |
| **LP_3D1.tig00871.3** | 2 | 5 | 2 | 43 | 22 | 8 | 12.770835368169700 | 2.9169506959883300 | 0.7795079516784430 | 3.74204097560202 | 0.0001825317429432020 | 0.01016260227973190 | 1.9929950704950800 | up |
| **LP_3D1.tig13121.1** | 5 | 16 | 33 | 84 | 57 | 74 | 43.47188630033420 | 1.9676720195937900 | 0.4844895607546180 | 4.061330065665470 | 4.87939288333593E-05 | 0.003206579057262780 | 2.4939580482121300 | up |
| **LP_3D1.tig12289.1** | 0 | 1 | 0 | 29 | 19 | 6 | 8.527800584032360 | 5.544984800083380 | 1.555781493445160 | 3.5641154130227100 | 0.00036508530309860000 | 0.018496679193083700 | 1.7329062357902400 | up |
| **LP_3D3.tig04251.1** | 549 | 220 | 189 | 76 | 107 | 99 | 211.30488644692900 | -1.8532786069106300 | 0.41154352159347200 | -4.503238441793090 | 6.6925722801001E-06 | 0.0005505633448079460 | 3.2591927062691700 | down |
| **C0_3D1.tig06494.1** | 21 | 30 | 65 | 144 | 72 | 122 | 73.64300706282310 | 1.510027698359970 | 0.4211187788258360 | 3.5857524629279900 | 0.0003361076633083280 | 0.017274900651683400 | 1.762584441583270 | up |
| **LP_3D2.tig02063.1** | 0 | 2 | 1 | 130 | 79 | 31 | 37.75299857862500 | 6.223725139071860 | 0.9559013129539310 | 6.510844848449130 | 7.47292995335366E-11 | 1.38370913093938E-08 | 7.858955193191540 | up |
| **LP_3D1.tig03276.2** | 0 | 1 | 0 | 60 | 37 | 14 | 17.392329083269700 | 6.585281620361590 | 1.4966673931845000 | 4.399963312055520 | 1.0826918139139E-05 | 0.0008454068154112000 | 3.0729342553435900 | up |
| **C0_3D1.tig24455.1** | 61 | 66 | 42 | 182 | 209 | 145 | 114.30052956034600 | 1.5928292828861700 | 0.32053097375347100 | 4.969345908240560 | 6.71791328684517E-07 | 6.75118447615695E-05 | 4.170620024749680 | up |
| **LP_3D1.tig01886.2** | 2 | 2 | 1 | 107 | 48 | 30 | 29.658352039171100 | 5.10613740455018 | 0.7994616279217110 | 6.386969963554280 | 1.69204856541259E-10 | 2.95044480677542E-08 | 7.530112505186000 | up |
| **FM1.tig39129.1** | 5 | 24 | 27 | 244 | 173 | 123 | 94.5643897104262 | 3.213896623270280 | 0.425330435366724 | 7.556234767209220 | 4.14904683188792E-14 | 1.16758696613202E-11 | 10.932710761721500 | up |
| **C0_3D1.tig00394.3** | 18 | 24 | 3 | 1 | 1 | 2 | 8.305505477437780 | -3.554193137033050 | 1.0353609932911900 | -3.4328057171007000 | 0.0005973699888767280 | 0.02827435222123030 | 1.5486073361803300 | down |
| **LP_3D1.tig00993.2** | 9 | 25 | 31 | 224 | 141 | 71 | 78.78463717823980 | 2.6702344213630000 | 0.44423761845196900 | 6.010824636301500 | 1.84581986612726E-09 | 2.82058145940871E-07 | 6.549661353176370 | up |
| **C0_3D1.tig00075.1** | 210 | 601 | 737 | 3098 | 1764 | 916 | 1154.9402982500100 | 1.8289049787303900 | 0.40639131983822800 | 4.500354435371360 | 6.78402487423297E-06 | 0.0005556741988123530 | 3.255179768004190 | up |
| **LP_3D3.tig08968.1** | 3 | 4 | 7 | 30 | 15 | 46 | 17.369443541505800 | 2.6856412614490800 | 0.6641496655641100 | 4.043728997692060 | 5.26077239111872E-05 | 0.0034324119036524800 | 2.4644006005583800 | up |
| **C0_3D3.tig03491.2** | 70 | 120 | 129 | 2560 | 1718 | 893 | 862.7844943808600 | 3.937051715213450 | 0.3387994256798720 | 11.620597370591600 | 3.23863769100798E-31 | 5.93872127565641E-28 | 27.226307057158600 | up |
| **C0_3D2.tig32963.1** | 26 | 32 | 47 | 753 | 455 | 268 | 248.7432201406640 | 3.72770406424189 | 0.36468366713110900 | 10.22174668135530 | 1.58459496013707E-24 | 1.45284355659664E-21 | 20.837781148375600 | up |
| **LP_3D1.tig02383.2** | 1 | 2 | 2 | 100 | 38 | 12 | 23.80341824806900 | 4.789720202142420 | 0.868335730344956 | 5.515977328538170 | 3.46846973757285E-08 | 4.29553730353657E-06 | 5.366982505088530 | up |
| **LP_3D2.tig00941.1** | 2 | 3 | 2 | 65 | 75 | 44 | 30.4737392683955 | 4.646420962128050 | 0.6754402136856570 | 6.879100278578390 | 6.02317521184831E-12 | 1.31182909930083E-09 | 8.882122739705050 | up |
| **LP_3D1.tig02050.1** | 6 | 3 | 12 | 165 | 144 | 72 | 63.50076795554700 | 4.098988752957760 | 0.49240692064254800 | 8.32439306013192 | 8.47619117990541E-17 | 3.27774889538587E-14 | 13.484424320297400 | up |
| **LP_3D1.tig02435.2** | 2 | 7 | 3 | 179 | 79 | 34 | 47.0730759726803 | 4.500017698605430 | 0.6385539420794420 | 7.047200560615410 | 1.82553109897024E-12 | 4.16756286429572E-10 | 9.380117840471570 | up |
| **LP_3D1.tig01443.3** | 0 | 2 | 0 | 25 | 19 | 19 | 10.425069388872200 | 4.920458845553850 | 1.1783481777684200 | 4.175725764580300 | 2.97037491436222E-05 | 0.0020743361425911600 | 2.683120865573050 | up |
| **LP_3D1.tig17571.1** | 5 | 2 | 6 | 37 | 19 | 62 | 21.78218866322510 | 3.1601061494226600 | 0.6607122851407640 | 4.782877843340550 | 1.72803149359759E-06 | 0.00015946420495707000 | 3.797336788056690 | up |
| **LP_3D1.tig27086.1** | 0 | 1 | 5 | 25 | 9 | 15 | 8.779033529071490 | 2.984268426160040 | 0.9113064496645390 | 3.274714479699530 | 0.001057688151885630 | 0.046072247504934000 | 1.3365606013964900 | up |
| **LP_3D3.tig12206.1** | 4 | 19 | 18 | 431 | 236 | 121 | 129.4552628710130 | 4.184302931140230 | 0.4702309631860240 | 8.898399422253500 | 5.66577991863571E-19 | 2.77647637478316E-16 | 15.556506017615500 | up |
| **LP_3D1.tig00497.1** | 1 | 1 | 1 | 75 | 49 | 27 | 24.174444632371400 | 5.562627829371850 | 0.9522402909282710 | 5.8416219964282800 | 5.16950004558257E-09 | 7.45838147439445E-07 | 6.127355407514320 | up |
| **C0_3D1.tig23744.1** | 19 | 28 | 51 | 200 | 147 | 72 | 81.83489438555700 | 2.0207203828978300 | 0.41255455148282500 | 4.898068329715070 | 9.67833817324639E-07 | 9.46927940547661E-05 | 4.023683068740380 | up |
| **LP_3D2.tig16612.1** | 2 | 0 | 1 | 15 | 17 | 7 | 6.671543460919480 | 3.6052351052498400 | 1.0972322226726300 | 3.28575394593156 | 0.0010170981437507500 | 0.044406254978119400 | 1.3525558517062800 | up |
| **C0_3D1.tig00004.26** | 51 | 129 | 170 | 576 | 344 | 150 | 223.85244596393200 | 1.5348795605935400 | 0.4233829802415760 | 3.6252745911462000 | 0.0002886545778333620 | 0.01516503648515480 | 1.8191565404946100 | up |
| **LP_3D1.tig02262.1** | 8 | 3 | 7 | 132 | 80 | 38 | 42.03424017034300 | 3.688046337823240 | 0.551487990687421 | 6.687446327210400 | 2.27098459241303E-11 | 4.52961821599013E-09 | 8.343938401447690 | up |
| **C0_3D1.tig20905.1** | 6 | 13 | 7 | 33 | 40 | 64 | 26.964405714242400 | 2.379244307684910 | 0.5354196119367220 | 4.443700332676840 | 8.84247438774321E-06 | 0.0007039922360942060 | 3.1524321304034000 | up |
| **LP_3D1.tig02921.2** | 4 | 1 | 4 | 38 | 40 | 13 | 15.776303307173600 | 3.240043840216140 | 0.7237851398431980 | 4.47652716511708 | 7.58670187486886E-06 | 0.0006169757769340780 | 3.209731886453590 | up |
| **C0_3D1.tig12378.2** | 53 | 37 | 56 | 28 | 17 | 9 | 33.01963682129090 | -1.5274670778476500 | 0.4596727500713330 | -3.322944589625160 | 0.0008907260576179510 | 0.03968128741794080 | 1.4014142460582500 | down |
| **LP_3D1.tig12538.1** | 1 | 0 | 0 | 23 | 18 | 9 | 8.035181094143650 | 5.451530149152620 | 1.5426671626621700 | 3.5338343105358800 | 0.0004095777492974390 | 0.02038743183783970 | 1.6906374779442900 | up |
| **LP_3D1.tig23455.1** | 0 | 1 | 1 | 30 | 21 | 14 | 10.583591312111900 | 4.948735941530060 | 1.1781436017400800 | 4.200452248962620 | 2.66382347525016E-05 | 0.0018810564652247900 | 2.725598167677970 | up |
| **LP_3D1.tig08962.1** | 2 | 2 | 1 | 40 | 25 | 7 | 11.968105554976500 | 3.7349045110514600 | 0.897056896288862 | 4.163509055560260 | 3.13393590301162E-05 | 0.0021725437366670200 | 2.6630314717713800 | up |
| **LP_3D2.tig14104.1** | 3 | 1 | 2 | 33 | 33 | 37 | 17.735590439695500 | 4.046851231520100 | 0.7540647761979670 | 5.366715644675150 | 8.01833191947584E-08 | 9.35938558444772E-06 | 5.0287526604544500 | up |
| **LP_3D3.tig12057.1** | 14 | 20 | 19 | 62 | 41 | 68 | 36.658518656514300 | 1.6507548842304800 | 0.43284108524713000 | 3.813766623581910 | 0.00013686471932459900 | 0.007979564071801870 | 2.098020833759830 | up |
| **LP_3D1.tig05440.2** | 16 | 16 | 23 | 104 | 49 | 88 | 48.095464172811600 | 2.0811716927237900 | 0.4267104976765860 | 4.877245120651250 | 1.07577718891212E-06 | 0.0001048928890286610 | 3.9792539528006400 | up |
| **C0_3D2.tig01584.2** | 45 | 111 | 126 | 588 | 163 | 204 | 195.90169715309800 | 1.6886589208066300 | 0.43399250293997300 | 3.8909863865556000 | 9.98375348581204E-05 | 0.006056845964791740 | 2.2177534709943800 | up |
| **LP_3D1.tig00106.1** | 1 | 5 | 7 | 39 | 22 | 10 | 13.153200106809200 | 2.370789352872600 | 0.7191151985056830 | 3.2968144155471700 | 0.0009778809321764730 | 0.04298094497242100 | 1.3667240404081700 | up |
| **LP_3D1.tig00051.3** | 1 | 2 | 6 | 232 | 158 | 75 | 74.16779713352450 | 5.604226374488950 | 0.6232695768790300 | 8.991657193588110 | 2.43530274360488E-19 | 1.24716022036234E-16 | 15.90407774993010 | up |
| **LP_3D1.tig00078.2** | 4 | 1 | 1 | 51 | 45 | 13 | 18.013108930625700 | 4.066671938431410 | 0.8022913260894880 | 5.068822017873610 | 4.00285310511894E-07 | 4.25312494879414E-05 | 4.371291858345300 | up |
| **LP_3D1.tig00715.6** | 30 | 24 | 41 | 96 | 130 | 111 | 70.43660095263230 | 1.777150294082890 | 0.36017181595373200 | 4.934173678684460 | 8.04907935518638E-07 | 7.94357492961059E-05 | 4.099984003518680 | up |
| **C0_90D1.tig15340.2** | 1098 | 1158 | 1609 | 295 | 538 | 436 | 850.9273035805890 | -1.6443699587172500 | 0.2989167084055120 | -5.50109750468176 | 3.77434397837011E-08 | 4.63297449989034E-06 | 5.334140090167530 | down |
| **LP_3D1.tig11340.1** | 2 | 2 | 1 | 34 | 27 | 12 | 12.285161228364700 | 3.7740547364771000 | 0.8568339189316480 | 4.404651418541900 | 1.05954112261781E-05 | 0.0008284678832903600 | 3.081724322947880 | up |
| **C0_3D3.tig07594.2** | 3 | 7 | 14 | 119 | 98 | 45 | 45.03929368412600 | 3.375137307209360 | 0.5129411655989870 | 6.579969660395730 | 4.70544275134034E-11 | 9.00609068013271E-09 | 8.04546368454575 | up |
| **LP_3D1.tig07322.2** | 1 | 1 | 1 | 14 | 15 | 12 | 7.086693459588490 | 3.711509711355800 | 1.0647856645413800 | 3.4856871527796200 | 0.0004908747160823820 | 0.023890216811389500 | 1.6217799088703500 | up |
| **LP_3D1.tig01715.2** | 0 | 2 | 0 | 47 | 44 | 20 | 17.793391335888200 | 5.713634864663740 | 1.1398013301441600 | 5.012834003221480 | 5.36341641032065E-07 | 5.52325010588184E-05 | 4.257805290445510 | up |
| **LP_3D3.tig06764.1** | 18 | 19 | 21 | 516 | 444 | 180 | 188.27851290354400 | 4.202160857631390 | 0.38872018538223500 | 10.81024607327590 | 3.07843179453346E-27 | 3.8887434524501E-24 | 23.410190707112300 | up |
| **C0_3D1.tig07320.4** | 39 | 49 | 75 | 17 | 16 | 7 | 33.320866196844000 | -2.0927395982639800 | 0.4648091528284850 | -4.502363143085970 | 6.72020283608187E-06 | 0.000552037471411956 | 3.2580314420622600 | down |
| **LP_3D1.tig00025.3** | 4 | 18 | 15 | 162 | 158 | 77 | 68.69865018933800 | 3.358479963376710 | 0.4484659047992500 | 7.488818943504940 | 6.94961306771552E-14 | 1.91607318550361E-11 | 10.717587906816300 | up |
| **C0_3D1.tig01690.2** | 169 | 248 | 246 | 5503 | 4451 | 2541 | 2081.50529884283 | 4.159159985390370 | 0.2866889626743870 | 14.507569271560100 | 1.08497644569793E-47 | 6.85283178396654E-44 | 43.16412992865130 | up |
| **C0_3D1.tig20330.1** | 9 | 27 | 24 | 248 | 162 | 83 | 86.99973395142340 | 2.9612289504071 | 0.43116403145639400 | 6.867986971001750 | 6.51141664496338E-12 | 1.41156672150059E-09 | 8.850298588934220 | up |
| **LP_3D1.tig06390.1** | 0 | 4 | 1 | 48 | 46 | 32 | 20.890934392154400 | 4.594548924979050 | 0.7936281513087170 | 5.789296810354450 | 7.06817022965763E-09 | 9.89630878583468E-07 | 6.004526762258090 | up |
| **LP_3D1.tig15968.1** | 0 | 1 | 2 | 49 | 28 | 18 | 15.383050302456500 | 4.904740027367990 | 0.991048806854052 | 4.949039838852550 | 7.45804818065508E-07 | 7.42474166075898E-05 | 4.129318652752660 | up |
| **LP_3D2.tig11654.1** | 1 | 3 | 2 | 30 | 21 | 11 | 10.712158866936900 | 3.288233525802420 | 0.8340530756227060 | 3.9424751516531600 | 8.06450076867082E-05 | 0.0050156077264233400 | 2.2996764372713700 | up |
| **LP_3D1.tig00156.7** | 0 | 1 | 3 | 45 | 21 | 9 | 12.23848899853540 | 4.136719786405620 | 0.9629659327486550 | 4.295811145258190 | 1.74055716830383E-05 | 0.0012933591141468100 | 2.888280872151150 | up |
| **C0_3D3.tig05677.1** | 9 | 32 | 43 | 135 | 116 | 55 | 61.82096823083350 | 1.8048673149933100 | 0.4547485507605150 | 3.9689347266195200 | 7.21946524932115E-05 | 0.004559894467751780 | 2.3410452083450900 | up |
| **LP_3D2.tig00750.2** | 0 | 12 | 7 | 156 | 135 | 64 | 58.89286647686440 | 4.173884080125400 | 0.9852331024726260 | 4.236443202781410 | 2.2708832922235E-05 | 0.0016340298828663900 | 2.7867400054258200 | up |
| **LP_3D1.tig02677.3** | 0 | 7 | 2 | 150 | 90 | 41 | 45.21546943745970 | 4.877311972149990 | 0.6680982520880060 | 7.3002914719608 | 2.87144999600025E-13 | 7.25455888989486E-11 | 10.139388989499000 | up |
| **C0_3D3.tig18758.2** | 13 | 11 | 22 | 309 | 263 | 143 | 120.45544392595800 | 3.8777051869623500 | 0.39182130706731300 | 9.896616434634520 | 4.30597149150701E-23 | 3.23193497095538E-20 | 19.490537386152200 | up |
| **LP_3D2.tig01895.1** | 944 | 848 | 883 | 38595 | 43779 | 30575 | 18529.10561299550 | 5.327910350078970 | 0.24057307532167800 | 22.14674415644750 | 1.12135894422368E-108 | 6.37436491843953E-104 | 103.19556307774200 | up |
| **FM1.tig15994.1** | 0 | 5 | 5 | 92 | 50 | 24 | 27.430518706232600 | 3.97044291712092 | 0.6794205189296870 | 5.843866657686010 | 5.1002858882218E-09 | 7.37724558055899E-07 | 6.13210575910548 | up |
| **C0_3D3.tig16629.1** | 0 | 1 | 9 | 240 | 148 | 78 | 74.46473690810220 | 5.489965669514880 | 1.1990606097075300 | 4.57855560016599 | 4.68197720157272E-06 | 0.00040264295616248300 | 3.395079894054540 | up |
| **C0_3D2.tig23324.1** | 41 | 52 | 64 | 254 | 201 | 125 | 117.79212382384100 | 1.8109675109093600 | 0.3279828681464840 | 5.521530807824220 | 3.36058875121472E-08 | 4.17101894241923E-06 | 5.379757837828720 | up |
| **C0_3D1.tig06262.2** | 28 | 12 | 9 | 54 | 64 | 79 | 40.68146720628800 | 1.9400986507178300 | 0.4794694868910060 | 4.046344353001260 | 5.20236878713559E-05 | 0.0033991799276404900 | 2.468625846445700 | up |
| **LP_3D1.tig01946.1** | 4 | 18 | 15 | 432 | 316 | 109 | 139.34576681376300 | 4.448974032379660 | 0.48009389609701400 | 9.26688314212734 | 1.91664835307984E-20 | 1.15906250671089E-17 | 16.93589314247680 | up |
| **LP_3D1.tig01585.2** | 1 | 1 | 2 | 35 | 27 | 6 | 11.174941197774500 | 3.984077558201050 | 0.9660465580173870 | 4.124105122197780 | 3.7217889771315E-05 | 0.00251863207625048 | 2.5988352699471200 | up |
| **LP_3D1.tig02808.3** | 0 | 3 | 1 | 37 | 15 | 12 | 10.651065099287800 | 3.9190819236377300 | 0.9615277121869250 | 4.075890766293220 | 4.58385343958966E-05 | 0.0030404801490487100 | 2.5170578277016500 | up |
| **LP_3D1.tig00547.1** | 1 | 3 | 1 | 170 | 121 | 65 | 56.71965773254750 | 6.0658535462363600 | 0.7506945102808030 | 8.080322239158750 | 6.4595853791045E-16 | 2.30940333883771E-13 | 12.636500210636700 | up |
| **FM1.tig11130.3** | 0 | 2 | 3 | 40 | 16 | 10 | 11.05309761554440 | 3.637974673238520 | 0.9104234076766200 | 3.995915134170970 | 6.44448189036586E-05 | 0.0041139238521000200 | 2.3857437512700800 | up |
| **C0_3D2.tig17016.2** | 2 | 2 | 3 | 72 | 41 | 17 | 21.346452204409400 | 4.113634901264730 | 0.7479533312829300 | 5.499855043373890 | 3.8010360343002E-08 | 4.63669299076813E-06 | 5.333791659081420 | up |
| **LP_3D1.tig03857.2** | 42 | 79 | 81 | 350 | 253 | 152 | 152.34414472753700 | 1.8303252763182800 | 0.34251119258875 | 5.343840773448630 | 9.09975098488056E-08 | 1.0492400501735E-05 | 4.9791251404631300 | up |
| **LP_3D2.tig01953.2** | 3 | 6 | 15 | 67 | 53 | 31 | 27.74331832468340 | 2.5898251672432100 | 0.5439018721607540 | 4.7615669292599300 | 1.9209556777452E-06 | 0.0001752756428594320 | 3.756278431348910 | up |
| **LP_3D1.tig06017.1** | 0 | 2 | 2 | 126 | 55 | 31 | 33.557671333020300 | 5.632398278621280 | 0.8690323841477130 | 6.481229447099540 | 9.09781924636334E-11 | 1.65228605450327E-08 | 7.781914762622320 | up |
| **LP_3D1.tig00149.6** | 1 | 3 | 0 | 41 | 40 | 15 | 15.721960810498800 | 4.496004176980520 | 0.9019322005693110 | 4.984858256687790 | 6.20072529058781E-07 | 6.29428980613328E-05 | 4.20105326486942 | up |
| **C0_3D3.tig14904.1** | 42 | 32 | 56 | 600 | 420 | 199 | 212.47894967347800 | 3.1320811472790800 | 0.37021778304426600 | 8.460104540425560 | 2.67137940763162E-17 | 1.10842746296949E-14 | 13.955292722452800 | up |
| **LP_3D1.tig00027.12** | 2 | 0 | 5 | 30 | 22 | 25 | 13.551717541233900 | 3.4053094747294000 | 0.7722697877747990 | 4.409481671607780 | 1.03618327262502E-05 | 0.000812439146653371 | 3.0902091586720300 | up |
| **LP_3D1.tig01155.2** | 0 | 5 | 2 | 54 | 28 | 12 | 15.701261907509600 | 3.659211697654930 | 0.8064271480011710 | 4.537560158688530 | 5.69087991017828E-06 | 0.00047784057384650600 | 3.3207169767093700 | up |
| **LP_3D1.tig11586.1** | 1 | 1 | 5 | 80 | 45 | 26 | 24.752215707234200 | 4.346165755049200 | 0.7301893611955420 | 5.952107749054580 | 2.64710996542802E-09 | 3.94363802568741E-07 | 6.404102954446070 | up |
| **C0_3D1.tig09274.1** | 41 | 44 | 40 | 9 | 17 | 18 | 28.208493258944800 | -1.5492524052011600 | 0.46626968393394800 | -3.3226530880798700 | 0.0008916572940566260 | 0.03969166709526150 | 1.4013006598464800 | down |
| **LP_3D1.tig01758.1** | 0 | 1 | 0 | 29 | 29 | 6 | 10.09465173439550 | 5.791753354770480 | 1.543885674756840 | 3.751413365295110 | 0.00017584051436675900 | 0.009867378123571960 | 2.005798229065920 | up |
| **LP_3D1.tig03934.1** | 12 | 25 | 26 | 70 | 98 | 86 | 51.575076348005700 | 1.9764620491338900 | 0.4018464913706110 | 4.918450432135450 | 8.72319995423126E-07 | 8.56425391016021E-05 | 4.06731046536793 | up |
| **C0_90D1.tig10915.1** | 4 | 0 | 0 | 11 | 30 | 16 | 9.928915394342030 | 3.7458884550583800 | 0.986928578661432 | 3.7955010484537000 | 0.00014734557238101600 | 0.008486179394122430 | 2.071287791676880 | up |
| **LP_3D1.tig00427.4** | 0 | 2 | 5 | 51 | 26 | 20 | 16.368075099270300 | 3.723277959118760 | 0.7694758042231930 | 4.8387199944220600 | 1.30677996070849E-06 | 0.0001244286547177120 | 3.905079594301640 | up |
| **LP_3D1.tig05507.2** | 1 | 0 | 1 | 34 | 34 | 15 | 13.41341137034420 | 5.28864132685618 | 1.1564164287285100 | 4.573301792911300 | 4.80097838341257E-06 | 0.00041194082884270400 | 3.3851651615217700 | up |
| **LP_3D1.tig00182.2** | 3 | 2 | 8 | 100 | 70 | 40 | 35.15783801373390 | 3.932606102764620 | 0.5832869127638490 | 6.742146989258410 | 1.5606307559216E-11 | 3.17971524445746E-09 | 8.497611770989820 | up |
| **LP_3D2.tig26805.1** | 0 | 1 | 3 | 45 | 25 | 7 | 12.5068114555842 | 4.170537304591300 | 0.9724002824350540 | 4.288910009515390 | 1.79552098136041E-05 | 0.0013289894555394800 | 2.8764784648203900 | up |
| **LP_3D2.tig06087.2** | 25 | 26 | 19 | 104 | 62 | 54 | 46.655065772740800 | 1.5690589475732000 | 0.4014898724479940 | 3.908090976258550 | 9.30282781630117E-05 | 0.005680120807923090 | 2.2456424273776400 | up |
| **LP_3D1.tig12310.1** | 0 | 1 | 2 | 44 | 22 | 14 | 12.982470635691300 | 4.653431391739830 | 1.015795137613220 | 4.581072717746850 | 4.6259694533104E-06 | 0.00039842914177792300 | 3.3996489040573500 | up |
| **LP_3D1.tig00026.6** | 50 | 82 | 71 | 1606 | 1292 | 731 | 606.039799634622 | 4.082292313167040 | 0.3092603450192280 | 13.200180297649300 | 8.7525315756651E-40 | 3.10961035886677E-36 | 35.50729402563410 | up |
| **C0_3D1.tig24044.1** | 40 | 47 | 64 | 1033 | 761 | 375 | 365.20250826155300 | 3.757351827914980 | 0.34387539088862100 | 10.926492350050000 | 8.61130520146319E-28 | 1.11252191858449E-24 | 23.953691423918700 | up |
| **LP_3D1.tig25630.1** | 3 | 0 | 1 | 25 | 15 | 10 | 8.564322522125900 | 3.5390421991717500 | 0.9760712937430010 | 3.625802973469660 | 0.0002880649341336120 | 0.015148058446646800 | 1.8196430278839800 | up |
| **LP_3D1.tig00271.3** | 2 | 8 | 4 | 58 | 51 | 24 | 23.22036490614910 | 3.172159535372240 | 0.6053724474100110 | 5.240013067895340 | 1.60565226489198E-07 | 1.80382021734752E-05 | 4.743806749778970 | up |
| **LP_3D2.tig15892.1** | 0 | 2 | 1 | 73 | 46 | 40 | 25.717854934318800 | 5.658416821042790 | 0.9395420449442890 | 6.022526454766920 | 1.71715291965408E-09 | 2.63103929158319E-07 | 6.579872666171100 | up |
| **FM1.tig01889.5** | 0 | 7 | 4 | 86 | 62 | 29 | 29.479319960427300 | 3.932471121135570 | 0.6441189632391390 | 6.10519383152453 | 1.02675998737009E-09 | 1.60266615563334E-07 | 6.795156934206050 | up |
| **LP_3D1.tig00393.5** | 1 | 1 | 1 | 46 | 32 | 12 | 14.509591425474800 | 4.8075485053424200 | 1.0037331689185900 | 4.789667866134200 | 1.67057593989575E-06 | 0.00015603406413902200 | 3.8067805795131700 | up |
| **LP_3D2.tig20265.1** | 0 | 2 | 1 | 32 | 34 | 7 | 11.825946145885300 | 4.5141286601153900 | 1.0491152317739900 | 4.302795844916180 | 1.68656244231957E-05 | 0.0012565221760636400 | 2.900829842242850 | up |
| **LP_3D1.tig05993.1** | 0 | 0 | 3 | 61 | 24 | 21 | 17.076219706360300 | 5.061630748422480 | 1.0024203786828700 | 5.049409265874270 | 4.4317836836148E-07 | 4.62247235770795E-05 | 4.3351256772333200 | up |
| **LP_3D1.tig12892.2** | 11 | 23 | 6 | 101 | 27 | 32 | 31.677061014095300 | 1.9042683031237900 | 0.5696876480583070 | 3.3426533111858600 | 0.0008298150236309550 | 0.03754400896254960 | 1.4254593551202600 | up |
| **LP_3D2.tig00750.1** | 3 | 1 | 2 | 17 | 25 | 7 | 8.726213967824500 | 2.9336510913183400 | 0.8820254703570540 | 3.3260389749638100 | 0.0008808961019938310 | 0.03930497560269960 | 1.4055524689625200 | up |
| **C0_3D1.tig02919.1** | 54 | 55 | 57 | 10 | 13 | 23 | 35.49026882496460 | -1.8832767222413900 | 0.4524410039506230 | -4.1624802036000400 | 3.14809441884184E-05 | 0.002179700697187140 | 2.6616031368926600 | down |
| **C0_3D2.tig08140.2** | 3 | 15 | 8 | 83 | 56 | 33 | 31.29338738614270 | 2.654565160044940 | 0.5320967388922320 | 4.98887695792208 | 6.07313109663434E-07 | 6.17579851857208E-05 | 4.209306881017480 | up |
| **LP_3D1.tig04805.2** | 18 | 33 | 35 | 803 | 483 | 213 | 247.50238267506400 | 4.031882437686530 | 0.40497732108383900 | 9.955822777670660 | 2.3784721029969E-23 | 1.90428516471632E-20 | 19.720268015936100 | up |
| **LP_3D1.tig00528.1** | 0 | 5 | 3 | 100 | 43 | 39 | 29.89162689366780 | 4.433490625586510 | 0.6922187385053510 | 6.404753842924510 | 1.50612176497095E-10 | 2.65063441887845E-08 | 7.576650166952770 | up |
| **LP_3D1.tig00341.2** | 1 | 6 | 12 | 482 | 169 | 82 | 115.93740892077800 | 5.187366041808700 | 0.9926488674513120 | 5.225781453947140 | 1.73421100095157E-07 | 1.94057528246244E-05 | 4.71206950462516 | up |
| **C0_3D1.tig01022.1** | 67 | 71 | 88 | 395 | 193 | 101 | 144.84797208060300 | 1.5055874313757100 | 0.3901435647907470 | 3.859060015979580 | 0.00011382398494375400 | 0.00678231071711496 | 2.168622317825800 | up |
| **LP_3D1.tig02142.1** | 5 | 17 | 12 | 281 | 190 | 96 | 94.35474210463090 | 3.978702979492420 | 0.4533044616291280 | 8.777109682956530 | 1.67729405533002E-18 | 7.81522791600287E-16 | 15.10705835210170 | up |
| **LP_3D1.tig11140.1** | 0 | 1 | 0 | 46 | 26 | 3 | 11.615321483847400 | 5.997914304749090 | 1.570909719229640 | 3.8181152177799300 | 0.00013447512245800400 | 0.007848293979594700 | 2.105224737620390 | up |
| **LP_3D1.tig07244.1** | 0 | 0 | 2 | 118 | 53 | 23 | 30.295900016001000 | 6.495797748685150 | 1.1460240517607200 | 5.6681164227794100 | 1.4437585561212E-08 | 1.92202471013371E-06 | 5.716241033209760 | up |
| **LP_3D1.tig00271.4** | 4 | 0 | 3 | 50 | 43 | 28 | 20.396732751809100 | 4.026347750046880 | 0.7183676284503600 | 5.6048568874580100 | 2.08427107115453E-08 | 2.69272188795484E-06 | 5.569808499452850 | up |
| **LP_3D1.tig13450.2** | 3 | 1 | 1 | 31 | 22 | 27 | 13.762489079121800 | 3.9332751992411300 | 0.8283798766213860 | 4.748153969267470 | 2.05281737664421E-06 | 0.0001864095906954320 | 3.729531747137870 | up |
| **LP_3D1.tig01430.1** | 2 | 6 | 4 | 122 | 112 | 52 | 46.98959466525800 | 4.495136678939060 | 0.5696734771225370 | 7.890724879178740 | 3.00436316638735E-15 | 9.70358092007322E-13 | 12.013067968121500 | up |
| **LP_3D1.tig00371.5** | 1 | 12 | 9 | 930 | 131 | 118 | 183.5590960402300 | 5.649541863751810 | 1.1803528298540700 | 4.786316193650580 | 1.69870364672273E-06 | 0.00015752497356925500 | 3.802650584582080 | up |
| **C0_3D3.tig18162.1** | 13 | 12 | 15 | 3 | 0 | 2 | 7.510756587558340 | -3.0616816775210900 | 0.9328043272564720 | -3.2822335703844600 | 0.001029882588149220 | 0.044929912297269600 | 1.3474644291445500 | down |
| **LP_3D2.tig18928.1** | 0 | 0 | 1 | 29 | 27 | 7 | 9.95805423642455 | 5.771996175719870 | 1.5380091687007200 | 3.752901018526400 | 0.0001747998875898540 | 0.009828387349204020 | 2.0075177357586200 | up |
| **LP_3D1.tig00027.14** | 2 | 5 | 4 | 36 | 14 | 27 | 14.201283101899500 | 2.757510375718050 | 0.6951356960908640 | 3.966866312901310 | 7.28238130995643E-05 | 0.0045894342080318600 | 2.338240851603350 | up |
| **LP_3D1.tig01804.1** | 1 | 0 | 4 | 83 | 21 | 17 | 19.502591662094300 | 4.493524693436740 | 0.8848678093118730 | 5.078187550896640 | 3.810524466591E-07 | 4.05635324538137E-05 | 4.391864231722110 | up |
| **LP_3D1.tig27590.1** | 2 | 2 | 3 | 45 | 44 | 25 | 19.234561521989400 | 3.951044270740570 | 0.7139818718157590 | 5.533815950666220 | 3.1333776876266E-08 | 3.91465614622273E-06 | 5.4073063792909500 | up |
| **LP_3D1.tig03306.3** | 4 | 12 | 12 | 637 | 199 | 95 | 147.5388194767430 | 4.954108848112010 | 0.8499724993967440 | 5.828551925654200 | 5.59104006682675E-09 | 8.00560888158102E-07 | 6.096605631408080 | up |
| **LP_3D1.tig00081.2** | 1 | 4 | 7 | 243 | 156 | 69 | 74.9001036424869 | 5.197766639040110 | 0.5854591789562850 | 8.878102566102590 | 6.80105136375333E-19 | 3.27632004044541E-16 | 15.484613681694700 | up |
| **LP_3D1.tig01715.1** | 1 | 4 | 3 | 124 | 95 | 43 | 42.344325324379000 | 4.947712436742480 | 0.6572177566488580 | 7.52826956771646 | 5.14171581474863E-14 | 1.42576017311895E-11 | 10.845953520943200 | up |
| **LP_3D1.tig00981.1** | 2 | 7 | 5 | 406 | 173 | 78 | 103.7676534914110 | 5.448436577116990 | 0.5904487391811780 | 9.227619970317440 | 2.76711820486154E-20 | 1.55739439955796E-17 | 16.807601391374500 | up |
| **LP_3D1.tig02677.2** | 0 | 1 | 0 | 112 | 80 | 38 | 36.16458795867510 | 7.64603437100397 | 1.4560314227360700 | 5.251283901988950 | 1.51042665012242E-07 | 1.70357545488509E-05 | 4.768638625868040 | up |
| **C0_3D2.tig15893.1** | 53 | 84 | 29 | 15 | 12 | 23 | 36.12663403985650 | -1.7712187325435500 | 0.4932355601383260 | -3.5910199419661000 | 0.00032938642926589200 | 0.016990899792758300 | 1.7697836214439400 | down |
| **LP_3D1.tig14835.1** | 1 | 2 | 1 | 31 | 11 | 6 | 8.075509250356400 | 3.479623418034550 | 1.0134150627831400 | 3.433561968655230 | 0.0005957060172020580 | 0.028219090456542500 | 1.5494569883162700 | up |
| **LP_3D2.tig07077.2** | 1 | 2 | 4 | 31 | 22 | 8 | 10.637529193503500 | 3.03720347418247 | 0.8271869814021100 | 3.6717254290369800 | 0.00024091843850976900 | 0.012956488776809700 | 1.8875126768190600 | up |
| **LP_90D2.tig22768.1** | 136 | 53 | 48 | 9 | 22 | 31 | 51.283287298539200 | -1.994117744678100 | 0.526153567431841 | -3.7899918733068900 | 0.00015065221349755800 | 0.008659074900170590 | 2.0625285037346 | down |
| **C0_3D1.tig14211.2** | 12 | 26 | 41 | 8 | 2 | 4 | 15.179904879424300 | -2.5413636531687400 | 0.6795962900404360 | -3.739519609528080 | 0.00018437226566090300 | 0.010245006296670600 | 1.989487770341510 | down |
| **LP_3D1.tig00918.4** | 0 | 1 | 0 | 23 | 11 | 10 | 7.098796105509220 | 5.271253607043310 | 1.559275759739160 | 3.380578178118470 | 0.000723334945389084 | 0.03326697004097290 | 1.4779867529156700 | up |
| **LP_3D1.tig00028.6** | 0 | 5 | 1 | 42 | 32 | 16 | 15.100097353446300 | 3.8318108901628600 | 0.8121840055619440 | 4.717909813443890 | 2.3828007395867E-06 | 0.0002133075717193790 | 3.670993728246860 | up |
| **LP_3D3.tig25138.1** | 1 | 0 | 0 | 74 | 32 | 21 | 19.96433702084670 | 6.783713998029000 | 1.4870975668625700 | 4.5617141398066 | 5.07376938985359E-06 | 0.00043241142573647200 | 3.3641028390740900 | up |
| **LP_3D1.tig35929.1** | 116 | 87 | 56 | 48 | 16 | 27 | 58.605303458685100 | -1.6021191868895500 | 0.44027403522944600 | -3.6389136280875900 | 0.0002737906004839080 | 0.01446433706738640 | 1.8397014662381600 | down |
| **LP_3D1.tig00028.3** | 1 | 0 | 0 | 37 | 26 | 20 | 13.342133348457000 | 6.1935632276149000 | 1.4901269152626700 | 4.156399810094790 | 3.23301853044713E-05 | 0.002230351193728970 | 2.6516267470460900 | up |
| **LP_3D3.tig15080.2** | 2 | 4 | 1 | 1183 | 806 | 452 | 384.4065994323260 | 8.353755426798040 | 0.6365261182405180 | 13.123979028369600 | 2.39995394839797E-39 | 8.02502248215781E-36 | 35.095553742242300 | up |
| **LP_3D3.tig02991.2** | 30 | 51 | 68 | 250 | 102 | 101 | 95.86818462183150 | 1.5326759332050300 | 0.39842841463472800 | 3.8468037843389300 | 0.00011966873001692700 | 0.00710822252644955 | 2.148238984848670 | up |
| **FM1.tig38859.1** | 0 | 3 | 1 | 62 | 16 | 7 | 13.629870058355800 | 4.298649604477940 | 1.0023976486749300 | 4.288367605570850 | 1.79991032035372E-05 | 0.0013305058798505500 | 2.875983201848400 | up |
| **FM1.tig14547.2** | 4 | 5 | 12 | 197 | 170 | 64 | 70.86256357451770 | 4.273805897983770 | 0.510482925753518 | 8.372083927538330 | 5.66081027232072E-17 | 2.26611802767656E-14 | 13.644717474245900 | up |
| **LP_3D2.tig18123.1** | 0 | 1 | 1 | 20 | 18 | 11 | 8.088643021649300 | 4.547240426137810 | 1.2083643729718500 | 3.7631367887438900 | 0.0001677953606793770 | 0.009472023116007140 | 2.023557250745510 | up |
| **LP_3D1.tig09617.1** | 0 | 1 | 5 | 36 | 13 | 13 | 10.683348520900300 | 3.301230333217810 | 0.8844144510257610 | 3.7326734421729300 | 0.00018945815339437600 | 0.010507071931417900 | 1.978518294552130 | up |
| **LP_3D1.tig02677.1** | 1 | 15 | 6 | 692 | 432 | 182 | 206.8010711847090 | 5.818701335216960 | 0.9106707839822200 | 6.389467453619960 | 1.66464461871898E-10 | 2.91159148772555E-08 | 7.535869558985420 | up |
| **LP_3D1.tig14450.1** | 0 | 1 | 0 | 36 | 24 | 11 | 11.248357325451300 | 5.949394451512000 | 1.516706311335420 | 3.92257512680467 | 8.76075632123853E-05 | 0.005389666591783590 | 2.2684380997162600 | up |
| **LP_3D1.tig08575.1** | 0 | 1 | 2 | 28 | 17 | 4 | 8.027329539893260 | 3.9332088747426300 | 1.1142227316510900 | 3.530002362197630 | 0.00041555595670055500 | 0.020630810793574700 | 1.6854837038612600 | up |
| **C0_3D1.tig03010.3** | 22 | 50 | 50 | 12 | 11 | 6 | 24.695151641264800 | -2.1268070137373100 | 0.5192745619137170 | -4.095727327561060 | 4.20845034034902E-05 | 0.002821100938645520 | 2.5495813745633500 | down |
| **LP_3D1.tig04891.3** | 0 | 4 | 7 | 169 | 114 | 57 | 54.981796485210900 | 4.8722605983118700 | 0.6041702990352050 | 8.06438285048495 | 7.36067505305658E-16 | 2.58282452710495E-13 | 12.587905098084800 | up |
| **LP_3D1.tig02564.3** | 1 | 1 | 1 | 39 | 30 | 8 | 12.438299030713100 | 4.5774389530370500 | 1.0311346566473800 | 4.439225200634860 | 9.0283308998562E-06 | 0.0007147847771620130 | 3.145824705277940 | up |
| **LP_3D1.tig16711.1** | 3 | 1 | 3 | 56 | 37 | 32 | 21.047019447281000 | 4.081384470755550 | 0.7074306398384400 | 5.769306898677210 | 7.95982287294176E-09 | 1.10629860932121E-06 | 5.9561276335405200 | up |
| **FM2.tig19772.1** | 2 | 0 | 3 | 96 | 57 | 23 | 28.173189989987500 | 5.032041365719030 | 0.8126759895187990 | 6.1919404911896 | 5.94279903265192E-10 | 9.62445615416234E-08 | 7.016623801655230 | up |
| **LP_3D1.tig08364.1** | 0 | 2 | 0 | 56 | 59 | 21 | 21.66141640961930 | 6.002123524597380 | 1.1349034287312600 | 5.28866454417828 | 1.23212620545373E-07 | 1.40643000299232E-05 | 4.8518818774843100 | up |
| **C0_3D1.tig02954.1** | 16 | 13 | 16 | 109 | 171 | 126 | 73.15709551258400 | 3.117448310721150 | 0.39235251472092900 | 7.945529068262810 | 1.93363703389007E-15 | 6.3536183347677E-13 | 12.196978877025800 | up |
| **LP_3D2.tig28331.1** | 1 | 0 | 2 | 73 | 78 | 32 | 29.314468518577200 | 5.8461178719980200 | 0.9421496765998030 | 6.205083987394150 | 5.46676993554991E-10 | 8.9298430168487E-08 | 7.049156175771660 | up |
| **LP_3D1.tig00019.9** | 4 | 5 | 8 | 135 | 126 | 87 | 58.229089149930600 | 4.28882730386451 | 0.4839884036341970 | 8.861425752477430 | 7.89975533118682E-19 | 3.74217993167762E-16 | 15.426875334565600 | up |
| **C0_3D3.tig16707.1** | 0 | 0 | 5 | 20 | 17 | 33 | 12.352185345878100 | 3.8000176508431200 | 0.9017998684059310 | 4.2138148207542300 | 2.5109317926799E-05 | 0.0017819465387626600 | 2.749105329630600 | up |
| **C0_3D1.tig07272.2** | 63 | 44 | 42 | 20 | 17 | 11 | 32.89770617634640 | -1.7221694535785700 | 0.44824870201754800 | -3.8419954052898700 | 0.00012203812137143900 | 0.007211285872515010 | 2.1419872876321300 | down |
| **LP_3D1.tig00578.5** | 6 | 2 | 15 | 51 | 40 | 51 | 26.804902748829300 | 2.5839406300969100 | 0.5552438403975330 | 4.653704268465030 | 3.26024309371738E-06 | 0.00028634686970910500 | 3.5431075601514700 | up |
| **C0_3D2.tig03028.2** | 42 | 99 | 151 | 581 | 353 | 124 | 211.80134304666600 | 1.7751983919020000 | 0.44955251756857000 | 3.948812035361870 | 7.85399902793859E-05 | 0.004911557477922650 | 2.308780769219050 | up |
| **LP_3D1.tig09638.1** | 14 | 12 | 12 | 91 | 23 | 41 | 30.892796243287300 | 1.9404154102120400 | 0.5259440050315930 | 3.68939543306607 | 0.00022478759614974300 | 0.012169572288697300 | 1.9147246851982700 | up |
| **LP_3D1.tig02271.2** | 1 | 3 | 2 | 105 | 42 | 21 | 26.94913085349950 | 4.70299297941732 | 0.7858990100811220 | 5.984220515727420 | 2.17428579782188E-09 | 3.27844233891736E-07 | 6.484332450280290 | up |
| **LP_3D1.tig01757.2** | 0 | 4 | 1 | 111 | 77 | 40 | 36.55162039713280 | 5.427491768200230 | 0.7777752431984350 | 6.978226442231340 | 2.98929583345926E-12 | 6.69002053751936E-10 | 9.17457254900084 | up |
| **LP_3D3.tig01380.3** | 20 | 42 | 32 | 772 | 550 | 222 | 256.34721244640400 | 3.947839906165220 | 0.3956685293033120 | 9.977644451825680 | 1.90945919310924E-23 | 1.57308996858398E-20 | 19.803246438379800 | up |
| **LP_3D1.tig00949.4** | 0 | 2 | 1 | 29 | 32 | 17 | 12.85848614908200 | 4.633651099540390 | 0.9962607463629650 | 4.651042527226320 | 3.30261208227998E-06 | 0.00028927116150570900 | 3.5386948605905800 | up |
| **LP_3D1.tig06196.1** | 8 | 15 | 19 | 642 | 281 | 107 | 165.61369300619800 | 4.5116120656883800 | 0.4982535548061740 | 9.054851735966930 | 1.36753442696123E-19 | 7.19791615746401E-17 | 16.142793216388700 | up |
| **FM3.tig01224.1** | 28 | 33 | 26 | 11 | 10 | 7 | 19.054172875100300 | -1.7049120581597800 | 0.5239158124201810 | -3.2541717919985900 | 0.0011372348516028500 | 0.04904864578100440 | 1.3093729788641800 | down |
| **LP_3D2.tig03518.1** | 11 | 5 | 15 | 133 | 81 | 43 | 45.384557379364500 | 2.953800706642090 | 0.49667313702851600 | 5.947172267689000 | 2.72814256126028E-09 | 4.04911916174518E-07 | 6.392639442167900 | up |
| **LP_3D1.tig01630.1** | 3 | 6 | 17 | 70 | 27 | 57 | 29.09516293930610 | 2.5347876670548500 | 0.5714465395380440 | 4.435738939121000 | 9.17569922603809E-06 | 0.0007244341979224100 | 3.1400010563999000 | up |
| **C0_3D3.tig07840.1** | 25 | 53 | 26 | 7 | 13 | 12 | 22.530761033829000 | -1.7380023990634700 | 0.5336300148759080 | -3.2569427330050600 | 0.0011261915127387300 | 0.048716362831148900 | 1.3123251436372300 | down |
| **LP_3D2.tig31234.2** | 0 | 1 | 2 | 63 | 50 | 23 | 21.808340157989100 | 5.4200943889291400 | 0.9628108117914820 | 5.629449028354890 | 1.80786187131813E-08 | 2.36248064540412E-06 | 5.626631740756740 | up |
| **LP_3D1.tig05180.2** | 30 | 34 | 34 | 151 | 199 | 182 | 102.65610404307600 | 2.3934257321821200 | 0.3300831317361730 | 7.250978623455150 | 4.13770990954845E-13 | 1.01821696886702E-10 | 9.992159669600880 | up |
| **LP_3D1.tig05678.2** | 1 | 6 | 5 | 45 | 26 | 11 | 14.693923510824100 | 2.689238442600190 | 0.7116486636646750 | 3.7788849749984800 | 0.00015753216562080100 | 0.008981861539332420 | 2.0466336441260500 | up |
| **LP_3D2.tig13774.1** | 0 | 2 | 0 | 24 | 34 | 15 | 11.90978351415320 | 5.12084416756394 | 1.1739239148582500 | 4.362160190068410 | 1.28784517398343E-05 | 0.0009866247832222080 | 3.005847979608250 | up |
| **LP_3D1.tig00027.6** | 5 | 5 | 11 | 38 | 19 | 27 | 16.946084143851900 | 1.9458035872494200 | 0.5898720389325730 | 3.2986876115886600 | 0.0009713794473772780 | 0.04277154507061300 | 1.3688450610941000 | up |
| **LP_3D3.tig25261.1** | 2 | 5 | 3 | 206 | 187 | 92 | 78.07989220431820 | 5.518154169721890 | 0.5763598740382060 | 9.574147018701200 | 1.02701534472998E-21 | 6.9500818179971E-19 | 18.15801008276310 | up |
| **C0_3D3.tig17306.1** | 3 | 2 | 11 | 21 | 50 | 40 | 20.754800456057200 | 2.7688236183561000 | 0.639994672697136 | 4.326322915607130 | 1.51619104913478E-05 | 0.0011491717358408800 | 2.9396150641274400 | up |
| **LP_3D1.tig00062.6** | 3 | 4 | 3 | 118 | 108 | 38 | 42.9553399395354 | 4.627748744592080 | 0.6202151956159900 | 7.461521061243680 | 8.552928737499E-14 | 2.32627384728771E-11 | 10.633339161763400 | up |
| **LP_3D2.tig03450.2** | 0 | 8 | 4 | 378 | 122 | 58 | 87.66811153720570 | 5.4545024705579000 | 1.2000006251268500 | 4.545416357580090 | 5.48267666096069E-06 | 0.00046378386129808100 | 3.333684367968700 | up |
| **LP_3D1.tig00406.2** | 2 | 8 | 8 | 533 | 229 | 110 | 137.80750926564000 | 5.500307381533970 | 0.5525684275399410 | 9.954074658267360 | 2.42064243210832E-23 | 1.9111308201833E-20 | 19.718709583725100 | up |
| **LP_3D1.tig00249.6** | 0 | 1 | 6 | 80 | 75 | 29 | 29.96916170859320 | 4.640829186401830 | 0.7250583102185300 | 6.400628916318610 | 1.54738235085409E-10 | 2.71484412760187E-08 | 7.566255100316640 | up |
| **LP_3D1.tig02829.2** | 0 | 5 | 1 | 102 | 91 | 31 | 35.956249808066200 | 5.135964539013990 | 0.7523971544703970 | 6.826134985357740 | 8.72328548947604E-12 | 1.85028046137786E-09 | 8.732762437220220 | up |
| **FM1.tig06806.2** | 2 | 4 | 4 | 44 | 42 | 21 | 18.540566905197300 | 3.3448108913836000 | 0.658640735833096 | 5.078354115393190 | 3.80718599090406E-07 | 4.05635324538137E-05 | 4.391864231722110 | up |
| **LP_3D1.tig00460.3** | 13 | 57 | 28 | 291 | 62 | 70 | 81.63560675252550 | 2.0234035392275700 | 0.5361165617580800 | 3.7741858460634900 | 0.00016053105925965000 | 0.009125388063614830 | 2.039748657797190 | up |
| **LP_3D1.tig04274.1** | 2 | 6 | 12 | 347 | 117 | 68 | 85.3840230635682 | 4.638450523051830 | 0.5779892202808040 | 8.025150574258700 | 1.01401489050435E-15 | 3.47239014763372E-13 | 12.459371484692200 | up |
| **LP_3D1.tig00981.5** | 1 | 6 | 10 | 274 | 110 | 62 | 71.85363175282240 | 4.62460807880431 | 0.5818438892309220 | 7.948193947550250 | 1.89250207746724E-15 | 6.3059616248577E-13 | 12.2002486764938 | up |
| **LP_3D1.tig00028.5** | 0 | 1 | 0 | 58 | 36 | 16 | 17.29660878318800 | 6.576921079269300 | 1.4911775094379300 | 4.410555442020000 | 1.03105802230261E-05 | 0.0008106568918090190 | 3.0911629207871400 | up |
| **LP_3D1.tig00981.4** | 4 | 10 | 14 | 355 | 220 | 98 | 109.42106183026100 | 4.500018298184400 | 0.48272540198824100 | 9.32210793061605 | 1.14051508766565E-20 | 7.04701958242976E-18 | 17.151994521673000 | up |
| **LP_3D1.tig12943.1** | 2 | 0 | 3 | 38 | 9 | 11 | 9.875653993553480 | 3.4352504106549600 | 0.9434238159578870 | 3.6412589469845500 | 0.000271308080848543 | 0.01435987696074060 | 1.8428492812288400 | up |
| **LP_3D1.tig02340.3** | 28 | 48 | 70 | 1123 | 751 | 386 | 377.9398629537960 | 3.873900194106450 | 0.371092087597067 | 10.439188340530600 | 1.64208670471692E-25 | 1.6971712496297E-22 | 21.770274333865700 | up |
| **LP_3D1.tig00778.5** | 15 | 6 | 12 | 112 | 96 | 84 | 52.36656256294350 | 3.0707924800558000 | 0.4300421330482030 | 7.140678189575440 | 9.28715513060757E-13 | 2.17254458189048E-10 | 9.66303130281008 | up |
| **LP_3D1.tig00460.4** | 81 | 217 | 168 | 900 | 298 | 335 | 317.3999772000420 | 1.6462842973114500 | 0.40180779457013800 | 4.097193532725470 | 4.18189265852371E-05 | 0.00280660788871051 | 2.5518182584252300 | up |
| **LP_3D1.tig00339.1** | 6 | 10 | 13 | 257 | 237 | 93 | 96.81594835948860 | 4.255153886195740 | 0.45392787381811700 | 9.374074895212810 | 6.97850384221998E-21 | 4.40770056567772E-18 | 17.355787916672400 | up |
| **LP_3D3.tig20415.2** | 1 | 4 | 5 | 66 | 66 | 30 | 27.16464129518760 | 3.9454418956075800 | 0.6347366955454380 | 6.215871751698880 | 5.10404796497057E-10 | 8.36137194722628E-08 | 7.077722456983850 | up |
| **LP_3D2.tig34025.1** | 3 | 12 | 7 | 86 | 81 | 33 | 35.00929866972200 | 3.108213907835460 | 0.533749944600491 | 5.823352188189810 | 5.76788283926186E-09 | 8.196882499946E-07 | 6.086351290360600 | up |
| **LP_3D1.tig00205.3** | 2 | 5 | 1 | 241 | 140 | 76 | 72.73373478222130 | 5.739764022466440 | 0.6466515791126640 | 8.876130837479060 | 6.92264056750578E-19 | 3.30686977361232E-16 | 15.480582907510000 | up |
| **LP_3D1.tig00026.3** | 5 | 5 | 8 | 126 | 71 | 52 | 42.18180976803370 | 3.706636895429710 | 0.5196856623373990 | 7.132459415482630 | 9.85911527211958E-13 | 2.28751594956587E-10 | 9.64063586915909 | up |
| **C0_90D1.tig24947.1** | 4 | 9 | 10 | 168 | 161 | 89 | 69.94934021072640 | 4.113566314846570 | 0.45553784409539000 | 9.03013079630149 | 1.71466729283334E-19 | 8.86093293282828E-17 | 16.052520550545300 | up |
| **FM1.tig03230.2** | 3 | 13 | 11 | 310 | 217 | 86 | 99.93385419579680 | 4.419906062712860 | 0.4925083493597670 | 8.974276412691260 | 2.85220302946148E-19 | 1.44762036794409E-16 | 15.839345315019800 | up |
| **C0_3D1.tig17081.2** | 48 | 143 | 180 | 892 | 658 | 316 | 353.1287342004270 | 2.2655021035327200 | 0.40128971218087800 | 5.645552414539750 | 1.64651696698941E-08 | 2.16157637386866E-06 | 5.665229415155200 | up |
| **LP_3D1.tig06053.2** | 0 | 1 | 3 | 56 | 31 | 13 | 16.15816868987130 | 4.553470216022010 | 0.9214426156758630 | 4.941675301920040 | 7.74541305006614E-07 | 7.68390933387452E-05 | 4.1144177682286500 | up |
| **LP_3D1.tig02378.1** | 3 | 9 | 3 | 295 | 185 | 99 | 93.0887408473068 | 5.179762379621520 | 0.5332538757646080 | 9.71350160783079 | 2.64104765100255E-22 | 1.9003842243195E-19 | 18.721158583451400 | up |
| **C0_3D1.tig00004.24** | 11 | 29 | 45 | 156 | 89 | 41 | 58.399984570628200 | 1.674343547551660 | 0.48251733357675400 | 3.470017408784590 | 0.0005204246324595570 | 0.02511336012917110 | 1.6000951754619900 | up |
| **LP_3D1.tig33389.1** | 17 | 20 | 28 | 601 | 446 | 235 | 212.1914937707190 | 4.216737876527700 | 0.3695199922921730 | 11.411393062580500 | 3.66807201686038E-30 | 6.51598605620088E-27 | 26.18601985372530 | up |
| **LP_3D1.tig06187.2** | 0 | 0 | 4 | 28 | 13 | 13 | 9.171055715785720 | 3.6928443487027600 | 0.9906570772366280 | 3.72767169745933 | 0.0001932568973797660 | 0.010655371805579800 | 1.972431391534540 | up |
| **LP_90D2.tig10797.1** | 10 | 19 | 1 | 415 | 313 | 241 | 159.01263732865400 | 4.929355706843340 | 0.8460094250972390 | 5.826596679199840 | 5.65691018249003E-09 | 8.07957435486547E-07 | 6.092611517963330 | up |
| **C0_3D1.tig01042.1** | 226 | 199 | 196 | 789 | 757 | 662 | 459.2496727907460 | 1.7614907829666800 | 0.25228948230007000 | 6.982022266277400 | 2.90960999060108E-12 | 6.53742213105605E-10 | 9.184593471088960 | up |
| **LP_3D1.tig04792.1** | 9 | 45 | 62 | 426 | 234 | 108 | 138.23969979422500 | 2.6598573012314000 | 0.8051165085878370 | 3.3036924132840800 | 0.0009542046284033260 | 0.04214589129882450 | 1.3752447572960200 | up |
| **LP_3D1.tig00078.4** | 0 | 2 | 4 | 105 | 68 | 45 | 35.30028915777900 | 5.108816697128110 | 0.723122471843144 | 7.0649397523304900 | 1.6068569430738E-12 | 3.69804789186357E-10 | 9.432027468775170 | up |
| **LP_3D2.tig11532.3** | 82 | 128 | 185 | 610 | 369 | 218 | 252.86930707947400 | 1.5228530470041500 | 0.35765683513947800 | 4.257860880556830 | 2.06392316497348E-05 | 0.001506081030974550 | 2.822151661363470 | up |
| **LP_3D1.tig24338.1** | 4 | 3 | 1 | 104 | 56 | 30 | 30.9906240300965 | 4.461615671418460 | 0.687971753211732 | 6.485172756860760 | 8.86301705939578E-11 | 1.61999422746416E-08 | 7.790486532978930 | up |
| **LP_3D1.tig01364.2** | 0 | 5 | 10 | 177 | 118 | 48 | 55.827992519637800 | 4.4549365234003600 | 1.030745568886620 | 4.322052558724510 | 1.54584351150547E-05 | 0.0011700862105396600 | 2.9317821387817600 | up |
| **LP_3D2.tig30746.1** | 0 | 1 | 0 | 46 | 25 | 17 | 13.96756239048590 | 6.264534804431370 | 1.496682342900820 | 4.185614157971340 | 2.84396091026403E-05 | 0.001990947757930520 | 2.7009401356231 | up |
| **LP_3D1.tig08437.1** | 0 | 1 | 1 | 35 | 20 | 13 | 10.991330165881900 | 5.006370049747620 | 1.1821076379707300 | 4.235121987995800 | 2.28427666281274E-05 | 0.0016415892148873600 | 2.784735509839660 | up |
| **LP_3D1.tig01045.3** | 15 | 15 | 45 | 111 | 61 | 113 | 58.67376385763550 | 1.8989581181721600 | 0.45421363787175900 | 4.180759800762090 | 2.90536646844868E-05 | 0.0020314336641939100 | 2.6921973548511900 | up |
| **LP_3D1.tig06393.3** | 0 | 2 | 1 | 41 | 31 | 3 | 11.977594141221700 | 4.536261389874840 | 1.0984390336489600 | 4.1297343329156900 | 3.63182662255306E-05 | 0.0024636179517783900 | 2.6084266399731300 | up |
| **LP_3D1.tig23648.1** | 0 | 2 | 3 | 36 | 27 | 14 | 12.898563510853000 | 3.8719484866482400 | 0.8576704488745140 | 4.514494456150660 | 6.34679823443544E-06 | 0.0005251582905916780 | 3.2797097739862100 | up |
| **LP_3D1.tig07163.1** | 2 | 10 | 7 | 163 | 129 | 59 | 58.1353933072588 | 4.128724369377240 | 0.5185307148020750 | 7.962352569515930 | 1.68798679519334E-15 | 5.67772836525239E-13 | 12.245825388896100 | up |
| **LP_3D1.tig01935.3** | 1 | 3 | 0 | 59 | 52 | 26 | 22.250559901241100 | 5.014405589864050 | 0.8599903509508870 | 5.830769594472370 | 5.51723218563833E-09 | 7.91987534324774E-07 | 6.1012816540374900 | up |
| **LP_3D1.tig03233.2** | 2 | 3 | 3 | 41 | 29 | 23 | 16.093418578061600 | 3.4683245583002200 | 0.704115995754686 | 4.925785778496350 | 8.40220105886923E-07 | 8.26337576455746E-05 | 4.082842497907570 | up |
| **LP_3D1.tig13921.1** | 1 | 0 | 1 | 44 | 25 | 12 | 12.952884271081900 | 5.23847336825818 | 1.1744525293566700 | 4.4603534304853300 | 8.18246025693129E-06 | 0.0006588271293275620 | 3.1812285256609500 | up |
| **C0_3D2.tig24035.1** | 13 | 22 | 16 | 75 | 100 | 129 | 58.43172956243580 | 2.5497956636344700 | 0.4176682109279410 | 6.104835361948050 | 1.02906701874601E-09 | 1.60266615563334E-07 | 6.795156934206050 | up |
| **C0_90D2.tig00002.34** | 66 | 191 | 97 | 59 | 29 | 25 | 76.31351348681920 | -1.713001026854200 | 0.42593705546165400 | -4.021723409336990 | 5.77738751935294E-05 | 0.0037319953811093000 | 2.4280589024282100 | down |
| **LP_3D1.tig00328.5** | 2 | 2 | 5 | 88 | 32 | 12 | 21.739910449576900 | 3.762455834384410 | 0.7549099525390770 | 4.983979641187280 | 6.22896364193049E-07 | 6.31168339082957E-05 | 4.199854794504640 | up |
| **LP_3D1.tig13064.1** | 3 | 3 | 4 | 326 | 370 | 232 | 149.70608167670200 | 6.467447412598660 | 0.5421957891692370 | 11.928250904545400 | 8.43267006790559E-33 | 1.74104344670601E-29 | 28.759190391186400 | up |
| **LP_3D2.tig21215.1** | 6 | 3 | 3 | 21 | 41 | 33 | 17.516223476262700 | 2.9283426410483900 | 0.6494410036038360 | 4.5090202571112900 | 6.51276907632192E-06 | 0.0005365483451355360 | 3.270391140278370 | up |
| **C0_3D1.tig18618.1** | 22 | 37 | 18 | 5 | 8 | 4 | 15.592935511989800 | -2.242261133526210 | 0.6198379754087060 | -3.6174955754327800 | 0.00029746741410226600 | 0.015541852164194200 | 1.8084972264130400 | down |
| **LP_3D1.tig00318.5** | 2 | 1 | 1 | 52 | 43 | 31 | 20.711714630640900 | 4.8999982251983000 | 0.8482778976636310 | 5.776406810426300 | 7.63127367175248E-09 | 1.06584705619354E-06 | 5.972305109959190 | up |
| **FM3.tig10090.2** | 81 | 176 | 261 | 782 | 547 | 242 | 330.41964691304900 | 1.5280405648713000 | 0.3976904783346520 | 3.8422860191927300 | 0.00012189367086392400 | 0.007210245286430550 | 2.1420499606992600 | up |
| **LP_3D1.tig04275.1** | 1 | 4 | 0 | 178 | 117 | 56 | 55.67228528048220 | 6.038210620961640 | 0.7676460952641790 | 7.86587811520573 | 3.66517269961824E-15 | 1.16394827994301E-12 | 11.934066317138400 | up |
| **C0_3D1.tig07124.1** | 20 | 31 | 38 | 258 | 133 | 51 | 83.09240243740980 | 2.21264894857972 | 0.4575036898848460 | 4.836352137698930 | 1.32243561351128E-06 | 0.00012570878336128600 | 3.900634376792380 | up |
| **LP_3D1.tig00900.2** | 14 | 23 | 13 | 321 | 247 | 142 | 120.24454946661500 | 3.7436438176700700 | 0.3870086889786550 | 9.673281051001280 | 3.9161718153E-22 | 2.71481447366742E-19 | 18.56625984409190 | up |
| **LP_3D1.tig00088.3** | 6 | 5 | 8 | 81 | 47 | 37 | 29.221791596673700 | 3.0370898467553800 | 0.5320404592499230 | 5.708381372042830 | 1.14055629773206E-08 | 1.5660609358594E-06 | 5.805191343432220 | up |
| **LP_3D1.tig01163.3** | 5 | 13 | 10 | 210 | 156 | 85 | 75.5266845702432 | 3.930779632125540 | 0.45183695058879000 | 8.699553294619510 | 3.3319314715878E-18 | 1.51522915601927E-15 | 14.819521681571800 | up |
| **LP_3D1.tig18492.1** | 1 | 0 | 5 | 46 | 24 | 14 | 14.092157913485500 | 3.7225156075220800 | 0.836744002479353 | 4.4488106236697300 | 8.63471053980367E-06 | 0.000690351787109901 | 3.160929546555980 | up |
| **LP_3D1.tig00979.2** | 0 | 1 | 1 | 143 | 39 | 33 | 33.614999542193800 | 6.645248449133000 | 1.1456180630928400 | 5.800579323263050 | 6.60862025943341E-09 | 9.29121714077603E-07 | 6.031927390107720 | up |
| **LP_3D1.tig00078.5** | 3 | 3 | 1 | 33 | 16 | 13 | 10.935821877364800 | 3.0532904543148700 | 0.8042205265415200 | 3.7965835906294000 | 0.00014670390930914200 | 0.008466379415916910 | 2.072302272768840 | up |
| **C0_3D2.tig02719.1** | 4 | 14 | 12 | 378 | 255 | 137 | 125.64469244142900 | 4.602576741787340 | 0.4479968803924310 | 10.273680338478300 | 9.2603057037316E-25 | 8.92206911404446E-22 | 21.049534416856400 | up |
| **LP_3D1.tig07276.2** | 2 | 1 | 0 | 102 | 117 | 85 | 49.260007038925700 | 6.595964688734140 | 0.9024845303485480 | 7.308673408713960 | 2.6979261105633E-13 | 6.84658972120405E-11 | 10.164525696253600 | up |
| **LP_3D1.tig23260.1** | 15 | 9 | 10 | 36 | 40 | 35 | 23.67506459554920 | 1.638814596314780 | 0.4902925446123160 | 3.3425239978116 | 0.0008302017638477410 | 0.03754400896254960 | 1.4254593551202600 | up |
| **LP_3D1.tig00254.5** | 0 | 6 | 0 | 24 | 36 | 9 | 11.797732378924300 | 3.4499524763012200 | 0.9047882411472710 | 3.8129943774763100 | 0.00013729323706175800 | 0.007996346373745520 | 2.097108402029120 | up |
| **LP_3D1.tig09553.1** | 1 | 1 | 0 | 38 | 12 | 7 | 9.130009472889920 | 4.7156366329767600 | 1.2384000526420000 | 3.8078459564955700 | 0.0001401825478579040 | 0.00813130299283935 | 2.0898398557272100 | up |
| **C0_3D3.tig07326.1** | 40 | 62 | 68 | 25 | 25 | 11 | 37.81074018896330 | -1.546042775974150 | 0.42696100680940500 | -3.6210397467614800 | 0.000293421427480043 | 0.015372848889495900 | 1.813245641782200 | down |
| **LP_3D1.tig00618.7** | 171 | 306 | 351 | 7166 | 6444 | 3768 | 2887.587888592230 | 4.327886831563570 | 0.2982147857669380 | 14.512650070093800 | 1.0075165362878E-47 | 6.85283178396654E-44 | 43.16412992865130 | up |
| **LP_3D1.tig02671.1** | 3 | 3 | 4 | 105 | 65 | 47 | 35.89487901453950 | 4.357338581053150 | 0.6055270461406710 | 7.195943779596080 | 6.20301859688359E-13 | 1.49384729528579E-10 | 9.825693794892950 | up |
| **LP_3D1.tig04764.1** | 0 | 3 | 11 | 174 | 96 | 43 | 50.87380076844180 | 4.422836946329450 | 1.1562532981052100 | 3.8251453670033300 | 0.00013069494547713900 | 0.007635513027387410 | 2.1171617775364200 | up |
| **LP_3D1.tig06058.1** | 0 | 1 | 1 | 64 | 39 | 19 | 19.356672588917400 | 5.839921064246540 | 1.1430085931075400 | 5.109253858161580 | 3.23433544990261E-07 | 3.48211739866882E-05 | 4.45815659084805 | up |
| **LP_3D1.tig02671.2** | 1 | 1 | 2 | 47 | 48 | 25 | 19.655018771803700 | 4.831206393227820 | 0.8592534886919130 | 5.6225624414776800 | 1.88145526664216E-08 | 2.44739873300397E-06 | 5.6112952691295 | up |
| **LP_3D1.tig00123.3** | 2 | 4 | 9 | 55 | 33 | 15 | 18.49124885311340 | 2.697599124681050 | 0.652301272240671 | 4.135511058279450 | 3.54165558604742E-05 | 0.002416871690142440 | 2.6167464053400800 | up |
| **LP_3D1.tig11844.1** | 1 | 0 | 3 | 28 | 32 | 9 | 11.452471349657800 | 4.021006042142720 | 0.9517359515049200 | 4.2249176736305500 | 2.39028143646784E-05 | 0.0017048374938019300 | 2.7683170119258200 | up |
| **LP_3D1.tig01572.1** | 10 | 0 | 2 | 279 | 240 | 237 | 123.70428796377600 | 5.854733092003560 | 1.081831404531100 | 5.411872004715180 | 6.23692647684467E-08 | 7.4326642678456E-06 | 5.128855483760350 | up |
| **LP_3D1.tig00182.5** | 4 | 5 | 13 | 183 | 87 | 54 | 54.14345858557950 | 3.793433575380760 | 0.5318465814725510 | 7.132571135227920 | 9.85111347634886E-13 | 2.28751594956587E-10 | 9.64063586915909 | up |
| **LP_3D1.tig00234.1** | 0 | 6 | 5 | 155 | 93 | 43 | 47.10518326967390 | 4.641887682053750 | 0.6257533614172230 | 7.418078700433470 | 1.18831487195459E-13 | 3.15653078954479E-11 | 10.5007899701053 | up |
| **LP_3D1.tig05927.1** | 0 | 0 | 2 | 90 | 42 | 22 | 24.228810115068000 | 6.169835677065610 | 1.145392687348940 | 5.386655376110320 | 7.17808782119693E-08 | 8.50080004574874E-06 | 5.070540199097100 | up |
| **LP_3D1.tig00142.1** | 6 | 26 | 34 | 612 | 268 | 136 | 168.13690518586800 | 3.8679827339432400 | 0.7969617262580630 | 4.8534109060724900 | 1.21355875157521E-06 | 0.00011652828924542700 | 3.933568629386780 | up |
| **C0_3D3.tig05284.1** | 25 | 48 | 41 | 17 | 11 | 6 | 24.217440303447900 | -1.8139803821913200 | 0.5075844920223660 | -3.573750598573020 | 0.0003519041305736890 | 0.017989199912285400 | 1.744988151907730 | down |
| **LP_3D1.tig00673.2** | 1 | 5 | 4 | 339 | 103 | 50 | 77.15096703329940 | 5.518047671574450 | 0.9709014952277120 | 5.683426896237570 | 1.32022171603569E-08 | 1.77418447867728E-06 | 5.751001224467030 | up |
| **C0_3D1.tig02386.1** | 140 | 244 | 365 | 60 | 95 | 86 | 162.64432583455300 | -1.6574978311570500 | 0.35928031609164300 | -4.613383358119370 | 3.96166628760896E-06 | 0.0003448712406112270 | 3.4623430206648400 | down |
| **LP_3D1.tig01155.1** | 0 | 1 | 2 | 118 | 85 | 39 | 38.33962588293110 | 6.24590725489928 | 0.9382264501234150 | 6.6571425843704200 | 2.79202302879273E-11 | 5.5108523983237E-09 | 8.2587812208838 | up |
| **LP_3D1.tig16496.1** | 0 | 2 | 3 | 34 | 23 | 16 | 12.332787865662300 | 3.8015372591670600 | 0.8564517347600350 | 4.438705772756940 | 9.05014343501161E-06 | 0.0007155151649001880 | 3.1453811572044500 | up |
| **LP_3D1.tig31463.1** | 1 | 3 | 8 | 347 | 214 | 115 | 107.696583899738 | 5.732134597881290 | 0.56706387141108 | 10.108446132562600 | 5.06817091984942E-24 | 4.36515418089152E-21 | 20.360000412043700 | up |
| **LP_3D1.tig02424.1** | 27 | 51 | 21 | 145 | 87 | 179 | 83.81511969895540 | 2.021519166612800 | 0.414895664176095 | 4.872355488764040 | 1.10275513980163E-06 | 0.00010733923959250600 | 3.9692414856462300 | up |
| **LP_3D1.tig00427.5** | 3 | 5 | 5 | 69 | 51 | 26 | 25.068679198510500 | 3.4056708355845500 | 0.5987306735776100 | 5.688151594496670 | 1.28421798557539E-08 | 1.72989031729936E-06 | 5.7619814321901300 | up |
| **LP_3D1.tig02618.1** | 1 | 8 | 4 | 96 | 64 | 37 | 33.05734265051690 | 3.845734790573110 | 0.5949675470994280 | 6.463772367621980 | 1.02124462881156E-10 | 1.8371092064808E-08 | 7.735865026403880 | up |
| **LP_3D1.tig00954.1** | 0 | 3 | 2 | 10 | 21 | 17 | 8.63162464888262 | 3.2364380767751400 | 0.9234083249849750 | 3.5048829312079300 | 0.00045680810512959300 | 0.02238556615180320 | 1.6500319174299800 | up |
| **LP_3D2.tig00092.3** | 1 | 5 | 2 | 110 | 100 | 58 | 43.736149875237400 | 4.993073834159220 | 0.635749480415029 | 7.853838639238290 | 4.03493343662058E-15 | 1.27425439558165E-12 | 11.894743859623800 | up |
| **FM1.tig14222.1** | 3 | 6 | 11 | 124 | 103 | 39 | 44.84857438472470 | 3.6510339774247800 | 0.5371439424842470 | 6.797123989780190 | 1.06728240843804E-11 | 2.21422147838176E-09 | 8.65477894080674 | up |
| **LP_3D1.tig00516.2** | 0 | 1 | 2 | 68 | 60 | 16 | 22.864361267869400 | 5.491676073292810 | 0.9848371276717880 | 5.576227702011450 | 2.45790161023511E-08 | 3.12571402760212E-06 | 5.505050758215610 | up |
| **C0_3D2.tig02659.3** | 18 | 25 | 19 | 191 | 57 | 56 | 57.73801930235000 | 2.1949969304776400 | 0.4716918748021330 | 4.653455036507490 | 3.26418808288328E-06 | 0.00028634686970910500 | 3.5431075601514700 | up |
| **C0_3D1.tig12384.2** | 24 | 86 | 84 | 293 | 234 | 83 | 126.87894914795300 | 1.5803755462757300 | 0.4430186225746020 | 3.5672891967642000 | 0.0003606933375296290 | 0.01830679711774260 | 1.737387631436410 | up |
| **C0_3D1.tig02106.1** | 313 | 450 | 483 | 3486 | 2945 | 1705 | 1492.5799006949100 | 2.633390080143510 | 0.2803066773523130 | 9.39467480767018 | 5.73948422916784E-21 | 3.66585371918029E-18 | 17.435824868948400 | up |
| **C0_3D1.tig03610.1** | 239 | 100 | 69 | 41 | 42 | 48 | 91.88819055558160 | -1.7303468210605700 | 0.4486875481630060 | -3.8564627615472500 | 0.00011503961348564700 | 0.006847567359781790 | 2.1644636868815700 | down |
| **FM2.tig14715.2** | 1 | 1 | 1 | 17 | 16 | 14 | 8.047976359933920 | 3.908265227957190 | 1.0436095946024800 | 3.744949498519960 | 0.00018043006597478100 | 0.010075193615261700 | 1.9967465992581400 | up |
| **LP_3D1.tig20206.1** | 1 | 0 | 4 | 23 | 22 | 7 | 8.943591117394170 | 3.294470562269120 | 0.9401546697823480 | 3.5041793315049000 | 0.00045801680977541800 | 0.022425465591458800 | 1.6492585314855500 | up |
| **C0_3D1.tig28765.1** | 26 | 43 | 20 | 8 | 9 | 2 | 17.857200538926300 | -2.3100633238794200 | 0.6099043282758780 | -3.7875830958761600 | 0.0001521198175605200 | 0.008699447715520860 | 2.060508317693470 | down |
| **LP_3D1.tig04907.2** | 0 | 1 | 2 | 49 | 26 | 13 | 14.173635064642900 | 4.78520737223279 | 1.0137797779651500 | 4.720164552737120 | 2.35653928926795E-06 | 0.00021162318467367500 | 3.6744367542915700 | up |
| **LP_3D2.tig07878.2** | 0 | 0 | 2 | 176 | 61 | 50 | 45.01416643420550 | 7.069330423962890 | 1.1227239260986300 | 6.29658837727652 | 3.04267836424767E-10 | 5.13237541886228E-08 | 7.289681583712330 | up |
| **LP_3D1.tig06055.2** | 8 | 7 | 11 | 185 | 170 | 66 | 70.32618226290810 | 3.9254645981174400 | 0.4710671676661180 | 8.333131382443790 | 7.87317086563943E-17 | 3.06541368395393E-14 | 13.513510908246700 | up |
| **FM2.tig06674.1** | 4 | 11 | 12 | 422 | 169 | 107 | 112.85009815799000 | 4.6003443486655 | 0.5025131698378970 | 9.154674195204670 | 5.45220806544168E-20 | 3.00903657747604E-17 | 16.521572532997900 | up |
| **LP_3D1.tig03306.2** | 0 | 2 | 1 | 20 | 19 | 7 | 7.6909502914897300 | 3.860734369670050 | 1.0819103142504200 | 3.568442151644420 | 0.0003591101187937050 | 0.018259047140275700 | 1.7385218901378600 | up |
| **LP_3D2.tig30234.1** | 2 | 4 | 3 | 15 | 17 | 19 | 9.791927906586400 | 2.4626852507030200 | 0.7571172015950920 | 3.252713378476470 | 0.0011430873559566200 | 0.049226364204056000 | 1.3078022394745700 | up |
| **C0_3D2.tig01729.2** | 2421 | 3468 | 3219 | 619 | 1087 | 841 | 1930.4302367998600 | -1.8773284456443400 | 0.2838670301117200 | -6.613407851223490 | 3.7557182546263E-11 | 7.2864779585062E-09 | 8.137482344494910 | down |
| **LP_3D1.tig09577.2** | 1 | 0 | 2 | 29 | 16 | 8 | 8.755005210049110 | 4.04698363495019 | 1.0680711592130400 | 3.7890580604498700 | 0.0001512195716267050 | 0.008665399747096830 | 2.062211397871720 | up |
| **LP_3D2.tig07081.2** | 4 | 3 | 11 | 183 | 133 | 69 | 63.39414879537820 | 4.33558713222268 | 0.5180739130524930 | 8.368665209713790 | 5.82745584316958E-17 | 2.30042866253455E-14 | 13.638191229883600 | up |
| **LP_3D1.tig02418.1** | 14 | 21 | 16 | 286 | 167 | 112 | 97.28318877743930 | 3.382920034839900 | 0.39950987609530600 | 8.467675612687180 | 2.50338326464187E-17 | 1.04635898293064E-14 | 13.980319292945700 | up |
| **LP_3D1.tig07231.1** | 0 | 0 | 2 | 92 | 43 | 20 | 24.324530415149700 | 6.176025444246370 | 1.1504606944089900 | 5.368306343937350 | 7.94794700948693E-08 | 9.3154855207069E-06 | 5.030794504861270 | up |
| **LP_3D2.tig16734.1** | 0 | 2 | 4 | 48 | 28 | 12 | 14.641571643194200 | 3.7910300715480300 | 0.8299434541194150 | 4.56781730457815 | 4.92829291406085E-06 | 0.00042191085948763400 | 3.374779296246800 | up |
| **LP_3D1.tig12849.2** | 0 | 3 | 4 | 470 | 393 | 324 | 190.6699279734060 | 7.34418255136307 | 0.6157688681212880 | 11.926849393621000 | 8.57581432100772E-33 | 1.74104344670601E-29 | 28.759190391186400 | up |
| **LP_3D2.tig11496.1** | 0 | 0 | 1 | 55 | 16 | 15 | 13.535081429254300 | 6.21964265826738 | 1.520220776936530 | 4.091275920331040 | 4.29006342714364E-05 | 0.00287242232645442 | 2.5417517062944500 | up |
| **LP_3D1.tig30761.1** | 0 | 1 | 0 | 27 | 15 | 12 | 8.678860945034490 | 5.567136830792400 | 1.5331162554756900 | 3.631255497362820 | 0.0002820457881131540 | 0.014845271134529800 | 1.8284118660265200 | up |
| **LP_3D1.tig30739.1** | 7 | 14 | 22 | 294 | 271 | 119 | 114.57684630546700 | 3.917359062270950 | 0.42347910669457400 | 9.250418734580600 | 2.23614908829027E-20 | 1.33804099919853E-17 | 16.87353057905480 | up |
| **LP_3D1.tig02942.2** | 0 | 3 | 3 | 380 | 162 | 82 | 97.5616726674217 | 6.600480442363570 | 0.7367702352805840 | 8.95866869520036 | 3.28621913190155E-19 | 1.65314271285791E-16 | 15.781689652943200 | up |
| **LP_3D1.tig00028.2** | 5 | 17 | 20 | 296 | 186 | 89 | 95.98461267711530 | 3.6860818643595300 | 0.46133770396932700 | 7.98998614820479 | 1.34953916157071E-15 | 4.56634247854089E-13 | 12.340431519266600 | up |
| **C0_3D1.tig24653.1** | 269 | 218 | 255 | 37 | 76 | 56 | 152.42607530669400 | -2.189716771360810 | 0.3354121525233270 | -6.5284359999702800 | 6.64599986321662E-11 | 1.24683782912392E-08 | 7.904190029678370 | down |
| **C0_3D2.tig06751.1** | 32 | 35 | 23 | 59 | 80 | 170 | 66.94195072644630 | 1.7690802680164500 | 0.4556690801614740 | 3.882379439459770 | 0.00010343930385520700 | 0.006228821215730150 | 2.2055941343909300 | up |
| **FM1.tig00358.2** | 1 | 1 | 3 | 62 | 37 | 13 | 18.17189529043990 | 4.385579540978750 | 0.849231959340893 | 5.1641715702533 | 2.4150610696487E-07 | 2.66054547488722E-05 | 4.57502931357677 | up |
| **LP_3D1.tig00026.5** | 1 | 0 | 0 | 156 | 110 | 54 | 50.295253758584 | 8.122482398505710 | 1.4480728904401900 | 5.6091668120633 | 2.03302994682273E-08 | 2.63251907351112E-06 | 5.579628473517360 | up |
| **C0_3D1.tig03107.1** | 66 | 133 | 182 | 716 | 403 | 169 | 262.6125024818910 | 1.6724078024884900 | 0.4159861592042950 | 4.02034482514395 | 5.81130174808523E-05 | 0.0037496418600443200 | 2.4260102111080800 | up |
| **LP_3D1.tig08947.1** | 0 | 2 | 2 | 144 | 159 | 83 | 61.84887007057950 | 6.5210843810128800 | 0.8044673060326390 | 8.106089995344460 | 5.22747319360313E-16 | 1.89271155216796E-13 | 12.722915567149200 | up |
| **LP_3D1.tig01923.2** | 0 | 7 | 5 | 114 | 70 | 41 | 37.207675424832 | 4.154758792271250 | 0.6099166023725300 | 6.81201130795513 | 9.62435396493137E-12 | 2.01880590825286E-09 | 8.69490543292693 | up |
| **LP_3D2.tig10659.1** | 1 | 1 | 0 | 41 | 28 | 13 | 13.158405104972800 | 5.259763542160640 | 1.165933747641710 | 4.511202761562880 | 6.44610653611578E-06 | 0.000532600183205671 | 3.2735986885069200 | up |
| **LP_3D1.tig08058.2** | 0 | 6 | 1 | 78 | 51 | 18 | 23.952148089782200 | 4.30212843089097 | 0.770802509215185 | 5.5813627738074 | 2.38641299860967E-08 | 3.06056234683302E-06 | 5.514198769045120 | up |
| **LP_3D1.tig00772.3** | 1 | 18 | 13 | 493 | 345 | 129 | 155.6823245982280 | 4.852334610196080 | 0.940965752654131 | 5.156760059023790 | 2.51259434551165E-07 | 2.75730551294613E-05 | 4.559515110849000 | up |
| **LP_3D1.tig11334.1** | 4 | 3 | 10 | 81 | 14 | 15 | 19.741199535671300 | 2.5886365068773400 | 0.71527019609207 | 3.619102992156730 | 0.00029562597249756100 | 0.015474086930592900 | 1.8103949676845200 | up |
| **LP_3D1.tig29027.2** | 0 | 3 | 3 | 221 | 66 | 37 | 50.80796809699250 | 5.6625516052413900 | 1.0983517643150900 | 5.155499166309820 | 2.52956167457426E-07 | 2.77057675127502E-05 | 4.557429814392900 | up |
| **LP_3D3.tig16536.3** | 1 | 1 | 2 | 823 | 539 | 262 | 254.47178062058000 | 8.568420684249220 | 0.8044309753477120 | 10.651530021634900 | 1.71522424193434E-26 | 1.98983514352566E-23 | 22.701182903099300 | up |
| **LP_3D1.tig12334.1** | 28 | 36 | 37 | 295 | 43 | 53 | 76.95436374869880 | 1.8371520083803400 | 0.5384764799530940 | 3.4117590587064800 | 0.0006454513718111510 | 0.030173259235694800 | 1.5203777759494000 | up |
| **LP_3D1.tig15882.1** | 2 | 0 | 1 | 39 | 22 | 9 | 11.382825296899200 | 4.430504307383360 | 1.039452777896970 | 4.262343034329250 | 2.02294588334436E-05 | 0.0014780765904718500 | 2.8303030612338300 | up |
| **LP_3D1.tig01316.3** | 0 | 0 | 1 | 28 | 20 | 7 | 8.712531837099460 | 5.576290707834420 | 1.5470802987055800 | 3.6043964314586800 | 0.0003128791818779970 | 0.01622775282286020 | 1.7897416159840700 | up |
| **LP_3D2.tig00485.1** | 1 | 0 | 1 | 7 | 22 | 11 | 6.800735943801200 | 4.263812537023110 | 1.256536216409550 | 3.393306521006300 | 0.000690543207110653 | 0.03191376309610170 | 1.4960219831610900 | up |
| **C0_3D1.tig01790.2** | 45 | 80 | 37 | 17 | 27 | 4 | 34.54968464513990 | -1.8432644390940700 | 0.5231332512338560 | -3.523508465092920 | 0.00042587349277548300 | 0.02102878637682630 | 1.677185790797100 | down |
| **C0_3D1.tig05353.2** | 32 | 58 | 29 | 17 | 9 | 7 | 25.056392407765300 | -1.9242640147791400 | 0.5167007723611000 | -3.724136130058570 | 0.0001959851584744820 | 0.010774445196791000 | 1.967605083503710 | down |
| **LP_3D1.tig01443.1** | 4 | 11 | 13 | 234 | 142 | 58 | 72.03778118236730 | 3.8650609473711800 | 0.5045009720276520 | 7.6611565917049100 | 1.84266016803754E-14 | 5.54211731492562E-12 | 11.256324285359000 | up |
| **LP_3D1.tig00254.4** | 3 | 2 | 2 | 29 | 24 | 9 | 10.875124145732400 | 3.047479749077690 | 0.8061094095869740 | 3.780479067524000 | 0.0001565268798331110 | 0.008933504502121650 | 2.0489781393907200 | up |
| **LP_3D2.tig01675.1** | 99 | 179 | 133 | 1081 | 255 | 287 | 320.46843661051100 | 1.8843425930690900 | 0.4315283559792480 | 4.3666715453565800 | 1.26154214508286E-05 | 0.0009703973374456760 | 3.0130504037472500 | up |
| **LP_3D1.tig01758.2** | 1 | 9 | 14 | 169 | 88 | 34 | 48.8998768504211 | 3.517124662326070 | 0.5865364964602430 | 5.996429350180210 | 2.01703302170694E-09 | 3.05755312317149E-07 | 6.5146259886171700 | up |
| **LP_3D1.tig00156.3** | 4 | 5 | 10 | 76 | 32 | 21 | 23.218068967253400 | 2.6740902778909400 | 0.6021736085731160 | 4.440729782607620 | 8.96543153864479E-06 | 0.0007107949174536440 | 3.1482556862827900 | up |
| **LP_3D1.tig00027.1** | 2 | 2 | 2 | 10 | 19 | 19 | 8.87672695276022 | 2.962528703357950 | 0.8601836869527240 | 3.444065201762850 | 0.0005730374927693990 | 0.027263634658107500 | 1.5644162465668200 | up |
| **LP_3D2.tig10856.1** | 0 | 1 | 0 | 40 | 40 | 9 | 13.991807539219500 | 6.268320673121800 | 1.5155314774102200 | 4.136054424836690 | 3.53328424794728E-05 | 0.002414057008107730 | 2.617252478226130 | up |
| **LP_3D1.tig02262.2** | 0 | 2 | 5 | 84 | 33 | 18 | 22.014430505768000 | 4.179958895083860 | 0.7817102358167550 | 5.347197341885270 | 8.93266066707623E-08 | 1.03206726752022E-05 | 4.986291995576270 | up |
| **LP_3D1.tig00327.1** | 0 | 1 | 1 | 122 | 18 | 14 | 23.796382621526400 | 6.145061880980710 | 1.2137726841195800 | 5.062778196757730 | 4.13190478161691E-07 | 4.35337911315487E-05 | 4.3611735107470700 | up |
| **LP_3D2.tig07137.2** | 1 | 0 | 2 | 60 | 40 | 15 | 18.38043539795610 | 5.160313172114520 | 0.9912396335611640 | 5.205918929588590 | 1.93038914942765E-07 | 2.15162688625911E-05 | 4.6672330375074000 | up |
| **LP_3D1.tig03816.2** | 0 | 4 | 3 | 57 | 30 | 10 | 16.099957647252000 | 3.7003312829402500 | 0.812887268895366 | 4.552084187477360 | 5.31170708606404E-06 | 0.0004506626706079260 | 3.346148414017790 | up |
| **FM2.tig25003.1** | 103 | 102 | 149 | 797 | 483 | 264 | 300.60858033878800 | 2.0318845860604900 | 0.34457094203062600 | 5.896854140068180 | 3.70497090603844E-09 | 5.42806884416895E-07 | 6.265354652822710 | up |
| **LP_3D3.tig03299.1** | 4 | 5 | 1 | 37 | 19 | 43 | 17.883226313122600 | 3.2619221943989800 | 0.6911803851902980 | 4.719350063009820 | 2.36599360952901E-06 | 0.00021213707686699700 | 3.673383419783770 | up |
| **C0_3D2.tig08946.1** | 60 | 72 | 59 | 24 | 19 | 16 | 41.42745941693850 | -1.7620087670314900 | 0.39614333817070000 | -4.447907101424570 | 8.67110137676914E-06 | 0.0006922875811270250 | 3.15971345908101 | down |
| **LP_3D3.tig06137.1** | 5 | 8 | 27 | 78 | 49 | 63 | 37.09495016131020 | 2.213996874678410 | 0.510143804362002 | 4.339946610637150 | 1.42517337582817E-05 | 0.0010830746062694200 | 2.965341626500130 | up |
| **LP_3D1.tig01006.3** | 6 | 4 | 2 | 174 | 66 | 36 | 44.72858351911040 | 4.399077095940490 | 0.6362627319381540 | 6.913931737821930 | 4.71402078630931E-12 | 1.03863764185176E-09 | 8.983535941944820 | up |
| **LP_3D1.tig06901.1** | 0 | 2 | 1 | 183 | 132 | 71 | 61.108179223235600 | 6.922217877936280 | 0.9160041932969110 | 7.5569718223904800 | 4.12561297095307E-14 | 1.16676850414839E-11 | 10.933015302775500 | up |
| **LP_3D1.tig00626.1** | 5 | 5 | 7 | 128 | 75 | 38 | 40.43705550309550 | 3.729312869011500 | 0.549145352216845 | 6.791121610984480 | 1.11265081655617E-11 | 2.29995038789583E-09 | 8.638281532023910 | up |
| **LP_3D1.tig00188.5** | 1 | 1 | 3 | 35 | 21 | 17 | 12.366151416137200 | 3.795050671173740 | 0.8502473079603990 | 4.463466847401650 | 8.06440809131028E-06 | 0.0006502429474475640 | 3.186924349476140 | up |
| **FM2.tig04335.1** | 3 | 6 | 16 | 330 | 124 | 92 | 89.0748267189008 | 4.365304046274890 | 0.5308884550305450 | 8.222638870577320 | 1.99073583317465E-16 | 7.34827132706579E-14 | 13.133814816210500 | up |
| **LP_3D1.tig00578.9** | 5 | 3 | 10 | 40 | 21 | 26 | 16.892760346973800 | 2.211382868091610 | 0.6085516585376410 | 3.633845766529670 | 0.0002792278172197090 | 0.014737887901443200 | 1.8315647511087800 | up |
| **C0_3D1.tig11323.2** | 136 | 269 | 165 | 89 | 64 | 51 | 127.15989377343500 | -1.5460706496844600 | 0.33626691723649100 | -4.597748307774010 | 4.27081485078016E-06 | 0.00037008303383018000 | 3.4317008243424100 | down |
| **LP_3D1.tig05518.2** | 4 | 2 | 0 | 43 | 28 | 18 | 15.058130499628700 | 3.7884981214115800 | 0.7969360361855330 | 4.753829604123440 | 1.9959927893849E-06 | 0.0001818304649240140 | 3.740333350817290 | up |
| **LP_3D1.tig05537.1** | 8 | 10 | 23 | 108 | 54 | 17 | 34.32515728097010 | 2.027731079391700 | 0.569910246185248 | 3.557983196414740 | 0.00037371321431091800 | 0.018849802721831500 | 1.7246931906713600 | up |
| **LP_3D1.tig00618.5** | 2 | 2 | 2 | 179 | 139 | 49 | 58.19000520154700 | 5.833321962484800 | 0.7218869223887890 | 8.08065886992635 | 6.44177755275172E-16 | 2.30940333883771E-13 | 12.636500210636700 | up |
| **LP_3D2.tig08070.1** | 0 | 1 | 5 | 111 | 67 | 40 | 35.1374823299806 | 5.104367100038610 | 0.7394251747606380 | 6.903155686700770 | 5.08598361061164E-12 | 1.11626539901629E-09 | 8.95223253691713 | up |
| **LP_3D1.tig01966.2** | 0 | 0 | 3 | 62 | 54 | 20 | 21.746290749972300 | 5.418019317211710 | 0.9769817841131030 | 5.54567076409735 | 2.92829382505013E-08 | 3.68271819657024E-06 | 5.43383151230363 | up |
| **LP_3D1.tig00689.2** | 2 | 3 | 0 | 357 | 319 | 150 | 130.80918318696900 | 7.277262719986970 | 0.7307926725353580 | 9.958040075497430 | 2.32602875858934E-23 | 1.88890149688587E-20 | 19.72379068923240 | up |
| **LP_3D2.tig28312.1** | 1 | 0 | 1 | 31 | 13 | 8 | 8.42238116153157 | 4.596436063048120 | 1.2282793891707500 | 3.742174706807800 | 0.00018243460711916900 | 0.01016260227973190 | 1.9929950704950800 | up |
| **FM1.tig45965.1** | 2 | 6 | 6 | 112 | 75 | 32 | 36.440843220342200 | 3.879700647294580 | 0.5882117199231610 | 6.595755432757100 | 4.23095896539873E-11 | 8.12529940500307E-09 | 8.090160626958940 | up |
| **LP_3D1.tig00945.4** | 1 | 11 | 4 | 235 | 182 | 84 | 81.12938035641400 | 4.889158073033630 | 0.5398408364229030 | 9.056665859941610 | 1.34498847554648E-19 | 7.14540840116257E-17 | 16.145972943737600 | up |
| **LP_3D2.tig10200.2** | 0 | 2 | 4 | 25 | 26 | 10 | 10.549071746394300 | 3.2782368586068800 | 0.8561936848405570 | 3.828849612710430 | 0.0001287436441747670 | 0.007544775724860440 | 2.122353665484570 | up |
| **LP_3D1.tig00547.2** | 0 | 0 | 1 | 208 | 119 | 66 | 61.56847624980140 | 8.415381470531550 | 1.4480424215007100 | 5.811557275932610 | 6.18943492761839E-09 | 8.75219971294695E-07 | 6.057882780905380 | up |
| **LP_3D1.tig03817.1** | 0 | 3 | 0 | 42 | 15 | 17 | 12.130721185832900 | 4.547278082483890 | 1.0369696102842700 | 4.385160411053230 | 1.15900337593296E-05 | 0.0008988205580478710 | 3.046327002847650 | up |
| **FM2.tig02532.1** | 9 | 4 | 5 | 140 | 52 | 30 | 37.42686841332940 | 3.4991231081299700 | 0.5960105145939060 | 5.870908352202660 | 4.33413827847993E-09 | 6.30112763274147E-07 | 6.200581723426620 | up |
| **LP_3D1.tig00429.1** | 0 | 1 | 4 | 685 | 506 | 241 | 225.15230024726 | 8.074612433737820 | 0.7362975943803790 | 10.966506607335700 | 5.5370563891751E-28 | 7.49414215339663E-25 | 24.125278073205100 | up |
| **LP_3D1.tig00123.2** | 3 | 3 | 0 | 65 | 26 | 14 | 17.281111147994800 | 4.010506154514610 | 0.825040752037995 | 4.860979466296610 | 1.16806370543692E-06 | 0.00011292275737340400 | 3.947218525694850 | up |
| **LP_3D2.tig03134.1** | 1 | 9 | 6 | 221 | 134 | 35 | 62.74020890292350 | 4.5103983478448 | 0.6142680697239610 | 7.342719848472150 | 2.09296328688386E-13 | 5.33517928443557E-11 | 10.27285098187540 | up |
| **LP_3D1.tig10528.2** | 1 | 2 | 1 | 268 | 128 | 72 | 73.48366460659110 | 6.768238095466770 | 0.832837797780984 | 8.12671820791526 | 4.41069136706683E-16 | 1.6072163510315E-13 | 12.79392565793810 | up |
| **C0_3D2.tig01996.3** | 90 | 151 | 101 | 326 | 674 | 411 | 284.75200132831100 | 1.9973590995694000 | 0.33484863568987200 | 5.964961139693280 | 2.44692269803438E-09 | 3.67006123403073E-07 | 6.435326689595380 | up |
| **LP_3D1.tig25138.2** | 3 | 4 | 9 | 34 | 50 | 18 | 18.75052068381160 | 2.6041290403400700 | 0.6210348652858190 | 4.193209086806390 | 2.75035722201985E-05 | 0.0019301735343915800 | 2.7144036435100000 | up |
| **LP_3D3.tig27114.1** | 0 | 1 | 0 | 32 | 11 | 11 | 8.616544453695580 | 5.557773825033750 | 1.5487821306796100 | 3.588480080535920 | 0.0003326114006403710 | 0.01711067427095190 | 1.7667328761173500 | up |
| **LP_3D1.tig00062.3** | 2 | 6 | 14 | 158 | 90 | 46 | 49.42164442722670 | 3.661572952814940 | 0.5475026762100140 | 6.687771789832170 | 2.2659411960018E-11 | 4.52961821599013E-09 | 8.343938401447690 | up |
| **LP_3D1.tig00328.3** | 5 | 9 | 10 | 109 | 80 | 66 | 44.542426152918400 | 3.344735544529750 | 0.46236589177208100 | 7.233958222373620 | 4.69116139722748E-13 | 1.14943564493705E-10 | 9.939515339167320 | up |
| **LP_3D1.tig01177.1** | 2 | 2 | 6 | 124 | 58 | 38 | 35.987336758877300 | 4.369473714781150 | 0.6427145484115540 | 6.798467104222480 | 1.05738149472721E-11 | 2.2098107010209E-09 | 8.65564492768285 | up |
| **LP_3D1.tig09841.1** | 2 | 4 | 4 | 39 | 19 | 34 | 16.522893309134200 | 3.1557006805717800 | 0.6792332197409610 | 4.645975180329470 | 3.3847369904822E-06 | 0.0002960082680368620 | 3.5286961581551300 | up |
| **C0_3D3.tig30029.1** | 41 | 37 | 18 | 147 | 169 | 125 | 87.06657515081910 | 2.1222633742815100 | 0.36892223027088800 | 5.75260366588154 | 8.78793456568002E-09 | 1.21841497655142E-06 | 5.914204771364670 | up |
| **LP_3D1.tig01952.1** | 0 | 3 | 6 | 179 | 122 | 58 | 57.579272295211300 | 5.236646131241240 | 0.6364442996478680 | 8.227972399373480 | 1.90408940216086E-16 | 7.07437660561006E-14 | 13.150311824167200 | up |
| **LP_3D1.tig00516.4** | 0 | 2 | 3 | 69 | 33 | 13 | 18.567442803862600 | 4.428211298356000 | 0.8631074478046980 | 5.130544649590380 | 2.88905018706878E-07 | 3.12815348350333E-05 | 4.504711946340050 | up |
| **LP_3D2.tig02109.1** | 1 | 2 | 1 | 32 | 38 | 16 | 14.246823965889700 | 4.347606140024060 | 0.8953363426740330 | 4.855835659523650 | 1.19880096311783E-06 | 0.00011530599111410000 | 3.9381481268753100 | up |
| **LP_3D1.tig00081.3** | 1 | 6 | 4 | 165 | 97 | 40 | 48.70279712025850 | 4.6859788953032200 | 0.6258830834553050 | 7.486987616654200 | 7.04723484903886E-14 | 1.92596185093084E-11 | 10.71535231954500 | up |
| **LP_3D1.tig00026.8** | 2 | 1 | 4 | 50 | 41 | 19 | 18.43044886849930 | 3.888401640903650 | 0.7384953341797480 | 5.265302921950800 | 1.39958388238113E-07 | 1.58484752577601E-05 | 4.800012513824390 | up |
| **FM2.tig27339.1** | 3 | 1 | 9 | 89 | 66 | 51 | 34.863967766392300 | 3.918591083695290 | 0.5764769137031020 | 6.797481374446590 | 1.06463907587824E-11 | 2.21422147838176E-09 | 8.65477894080674 | up |
| **LP_3D1.tig20925.1** | 0 | 1 | 1 | 22 | 13 | 12 | 7.7818796361577500 | 4.4888602617297800 | 1.2162008816352600 | 3.6908871959492500 | 0.0002234732330685490 | 0.012121503753608500 | 1.9164434996925300 | up |
| **LP_3D1.tig14390.2** | 0 | 3 | 4 | 177 | 142 | 61 | 60.63310462933950 | 5.679654063275330 | 0.6769996495569800 | 8.38944904475508 | 4.88425720511901E-17 | 1.96911773634745E-14 | 13.705728316001400 | up |
| **FM1.tig19524.1** | 13 | 5 | 11 | 139 | 119 | 74 | 57.508855488813100 | 3.429183686049950 | 0.44721581555163200 | 7.667849764705020 | 1.74903574301529E-14 | 5.31678806479701E-12 | 11.274350650974800 | up |
| **LP_3D1.tig00263.5** | 7 | 7 | 4 | 128 | 112 | 63 | 50.9219931373374 | 3.9869857460970300 | 0.49662726947893700 | 8.028124895920820 | 9.89736959298057E-16 | 3.40979378492715E-13 | 12.46727188516480 | up |
| **C0_3D1.tig02151.2** | 2684 | 3616 | 3093 | 16009 | 6638 | 8718 | 6552.272366783420 | 1.6673876418598800 | 0.30627514589881700 | 5.444084066849920 | 5.20726249561895E-08 | 6.2317228750202E-06 | 5.2053918680000600 | up |
| **LP_3D1.tig15363.1** | 1 | 0 | 0 | 41 | 35 | 23 | 15.88483276471210 | 6.448261462635800 | 1.4778269015550300 | 4.363340155637100 | 1.2809154091218E-05 | 0.0009826401677669220 | 3.007605487012740 | up |
| **LP_3D1.tig00070.8** | 19 | 13 | 17 | 60 | 103 | 82 | 48.033499441846100 | 2.2677095676219400 | 0.42149803471054800 | 5.380118958749660 | 7.44366346640355E-08 | 8.77873547194419E-06 | 5.056568037295260 | up |
| **LP_3D1.tig32235.1** | 24 | 24 | 18 | 68 | 108 | 67 | 50.171946138515400 | 1.8143688706296100 | 0.39723652637888100 | 4.567477435091360 | 4.9362879788974E-06 | 0.0004219598348277030 | 3.3747288863437000 | up |
| **LP_3D1.tig00871.4** | 0 | 2 | 3 | 102 | 60 | 22 | 29.318799528116800 | 5.106995355324240 | 0.8186615961258930 | 6.238225146375240 | 4.42563382523273E-10 | 7.29203347232911E-08 | 7.137151346487930 | up |
| **LP_3D1.tig05507.1** | 1 | 5 | 2 | 58 | 40 | 17 | 19.253691017895300 | 3.7579531671958900 | 0.727038841718583 | 5.168847868310310 | 2.35541530443596E-07 | 2.59987539768276E-05 | 4.585047465642850 | up |
| **C0_3D1.tig01042.3** | 261 | 278 | 231 | 992 | 692 | 716 | 513.7128580237930 | 1.5704743165083800 | 0.2604267369266700 | 6.030388181496840 | 1.63566312437995E-09 | 2.51976342290997E-07 | 6.598640232610460 | up |
| **C0_3D2.tig12901.2** | 83 | 91 | 133 | 253 | 441 | 314 | 214.10847145581900 | 1.6716923379595900 | 0.3172922656739960 | 5.268619877665680 | 1.37453265628346E-07 | 1.55958700292282E-05 | 4.806990392734130 | up |
| **LP_3D1.tig14757.1** | 9 | 18 | 27 | 113 | 64 | 89 | 51.659698713260500 | 2.2588479346355400 | 0.42799415307735500 | 5.277754189850520 | 1.30776727949582E-07 | 1.4868006200588E-05 | 4.827747266457090 | up |
| **LP_3D1.tig00618.4** | 0 | 1 | 1 | 68 | 64 | 25 | 24.943960850398000 | 6.209263042670010 | 1.1230865322749200 | 5.528748555191500 | 3.22523223762474E-08 | 4.01177957434963E-06 | 5.39666293713554 | up |
| **LP_3D1.tig13831.1** | 2 | 7 | 3 | 47 | 48 | 22 | 20.433418970616500 | 3.2110577433118500 | 0.6339887838279220 | 5.064849450370400 | 4.0872323076378E-07 | 4.31856357858124E-05 | 4.364660682266600 | up |
| **LP_3D1.tig08908.2** | 0 | 0 | 1 | 29 | 13 | 6 | 7.585253624367980 | 5.373384440187200 | 1.5717726258085000 | 3.4186779639473700 | 0.0006292615297663550 | 0.02953787915736450 | 1.5296206905853300 | up |
| **LP_3D1.tig00371.1** | 1 | 8 | 3 | 325 | 74 | 48 | 70.49386096859760 | 5.117991396242530 | 1.066229212590940 | 4.800085512387890 | 1.58597896268326E-06 | 0.0001495107365401830 | 3.8253276190269700 | up |
| **LP_3D1.tig12568.1** | 2 | 7 | 3 | 196 | 125 | 49 | 59.49707838677910 | 4.84939825551978 | 0.6005927138971050 | 8.074354122701850 | 6.78347436101068E-16 | 2.41004125032283E-13 | 12.61797552396690 | up |
| **LP_3D1.tig02312.1** | 18 | 48 | 20 | 133 | 90 | 69 | 60.50876225704190 | 1.6954773821980900 | 0.4164176130319580 | 4.071579417242320 | 4.66954362117045E-05 | 0.003079352751107130 | 2.5115405582275900 | up |
| **LP_3D2.tig19409.1** | 0 | 1 | 0 | 23 | 21 | 17 | 9.920110266709730 | 5.759912342109790 | 1.5108658689204600 | 3.8123254092868900 | 0.00013766546743927400 | 0.008005378821495510 | 2.096618112065430 | up |
| **LP_3D2.tig02661.3** | 0 | 2 | 1 | 17 | 23 | 12 | 8.767555977163260 | 4.055839757064620 | 1.0472592339460800 | 3.8728135552285400 | 0.00010758612970195400 | 0.006460346107890730 | 2.1897442144011100 | up |
| **LP_3D1.tig00019.5** | 1 | 9 | 5 | 306 | 135 | 73 | 82.1885746812681 | 5.006604232853930 | 0.5804014299474350 | 8.626105957918420 | 6.34762158236745E-18 | 2.77561960653598E-15 | 14.55664005338370 | up |
| **C0_3D1.tig03972.2** | 30 | 27 | 32 | 11 | 10 | 7 | 19.402067950272000 | -1.7385529358159600 | 0.5204505666140850 | -3.340476593438120 | 0.0008363473026504830 | 0.03773187493584660 | 1.4232916137213300 | down |
| **LP_3D1.tig00266.7** | 0 | 1 | 1 | 47 | 30 | 14 | 14.522109446644100 | 5.4186317573641300 | 1.1630818774891100 | 4.6588566654155300 | 3.17970500523773E-06 | 0.00027979927402900800 | 3.553153416669080 | up |
| **LP_3D1.tig03088.1** | 0 | 1 | 2 | 60 | 6 | 2 | 10.704626281666500 | 4.374344185620770 | 1.2074034123082500 | 3.62293508617647 | 0.0002912789405930370 | 0.015288782435836700 | 1.815627099443950 | up |
| **LP_3D3.tig30346.1** | 2 | 6 | 3 | 58 | 30 | 15 | 17.832158262908500 | 3.1345386103758800 | 0.6898050403985430 | 4.544093514545590 | 5.51721621490495E-06 | 0.0004660121184788580 | 3.3316027894901200 | up |
| **LP_3D1.tig03514.1** | 0 | 2 | 4 | 136 | 79 | 33 | 39.48384182079810 | 5.277719808275610 | 0.7533301187163520 | 7.005852649657310 | 2.45486170981799E-12 | 5.53756404343666E-10 | 9.25668123800584 | up |
| **LP_3D1.tig01003.1** | 2 | 2 | 0 | 47 | 38 | 15 | 16.319748329780100 | 4.5430554928617600 | 0.8969217787084480 | 5.065163541244010 | 4.08049886822601E-07 | 4.31856357858124E-05 | 4.364660682266600 | up |
| **LP_3D1.tig01316.4** | 4 | 5 | 7 | 92 | 42 | 23 | 27.0428979511962 | 3.19713992786529 | 0.6026407674487390 | 5.305216806689470 | 1.12539157151405E-07 | 1.28977588473218E-05 | 4.889485747437170 | up |
| **C0_3D1.tig16205.1** | 34 | 56 | 76 | 270 | 190 | 75 | 110.78906390728100 | 1.6035717241209400 | 0.40589577798911700 | 3.950698211411140 | 7.7923532320259E-05 | 0.0048783735624946300 | 2.311724946556470 | up |
| **C0_3D1.tig10572.1** | 106 | 324 | 428 | 1226 | 946 | 418 | 545.8112722171030 | 1.530044320403020 | 0.4101119731124190 | 3.7307965158666800 | 0.0001908753466601590 | 0.010565052659101000 | 1.9761283339642500 | up |
| **LP_3D1.tig05145.1** | 0 | 2 | 2 | 139 | 34 | 26 | 31.304684632438600 | 5.532381458769820 | 0.9096235638648680 | 6.082056004863680 | 1.18651105808759E-09 | 1.84282024855161E-07 | 6.734517024449570 | up |
| **LP_3D1.tig06490.2** | 8 | 13 | 17 | 111 | 75 | 29 | 39.73947563121200 | 2.4106353074504900 | 0.49720590238529700 | 4.848364220709570 | 1.24483689946013E-06 | 0.00011933010716662900 | 3.9232499693495600 | up |
| **LP_3D1.tig00968.2** | 2 | 4 | 6 | 315 | 263 | 161 | 118.88218832128700 | 5.869754792528030 | 0.5242402669611790 | 11.196688164670700 | 4.23262287700045E-29 | 6.50279587684028E-26 | 25.186899878308500 | up |
| **LP_3D1.tig01698.1** | 0 | 9 | 4 | 72 | 33 | 17 | 21.027687610797000 | 3.1492271069043600 | 0.7017220966123920 | 4.487855123997740 | 7.19438241693001E-06 | 0.0005875929144976810 | 3.2309234497999400 | up |
| **LP_3D1.tig02942.1** | 0 | 1 | 0 | 73 | 48 | 23 | 22.66219208552470 | 6.969010064752660 | 1.4738848068881300 | 4.728327500346930 | 2.26376834743147E-06 | 0.00020393646863667500 | 3.6905051052102300 | up |
| **LP_3D1.tig01572.3** | 3 | 0 | 1 | 88 | 38 | 38 | 26.555707637777800 | 5.262086903682780 | 0.8572790547981320 | 6.138126056190510 | 8.350059457868E-10 | 1.31121306597377E-07 | 6.882326731759070 | up |
| **C0_3D3.tig00832.5** | 74 | 70 | 37 | 28 | 16 | 13 | 39.706874350320800 | -1.76010190866143 | 0.4525034684352340 | -3.8896981602105700 | 0.00010036898268271200 | 0.0060761180198070000 | 2.216373799184970 | down |
| **LP_3D1.tig00019.7** | 1 | 7 | 9 | 143 | 89 | 38 | 44.78148074606070 | 3.9071224275271600 | 0.5756355533920860 | 6.787493240303520 | 1.14098603349907E-11 | 2.34997648819763E-09 | 8.62893648287597 | up |
| **LP_90D1.tig01331.1** | 180 | 269 | 285 | 5771 | 4817 | 2896 | 2253.9762686633200 | 4.127357398817780 | 0.2802598007355790 | 14.726897642776400 | 4.33138211407272E-49 | 4.1036236045744E-45 | 44.386832480730800 | up |
| **LP_3D2.tig03169.2** | 1 | 9 | 8 | 129 | 92 | 42 | 44.05109421081320 | 3.7924239124795200 | 0.5541181109408850 | 6.844071394887330 | 7.69735280317251E-12 | 1.6574091670316E-09 | 8.780570263428710 | up |
| **LP_3D1.tig00471.4** | 2 | 0 | 2 | 49 | 40 | 17 | 17.28411721219790 | 4.63199391755876 | 0.8876795843655140 | 5.2180922025705500 | 1.80775474012708E-07 | 2.01889623187669E-05 | 4.694886002542980 | up |
| **C0_3D2.tig03705.2** | 42 | 51 | 51 | 545 | 822 | 602 | 341.7942264692890 | 3.7221684216333200 | 0.2980979875230800 | 12.486392318717500 | 8.8580067425379E-36 | 2.39777806323603E-32 | 31.620191017389200 | up |
| **LP_3D1.tig02618.2** | 3 | 7 | 6 | 286 | 118 | 54 | 73.34304307622420 | 4.733512634660270 | 0.5818656056304950 | 8.135061754562990 | 4.11728415966764E-16 | 1.5099807616536E-13 | 12.821028585926000 | up |
| **LP_3D1.tig07408.1** | 4 | 13 | 14 | 342 | 242 | 98 | 111.42206312112900 | 4.37508153276898 | 0.47322424443076900 | 9.245260749545220 | 2.34667834585053E-20 | 1.36119316908034E-17 | 16.86608023900480 | up |
| **LP_3D2.tig00155.5** | 0 | 2 | 1 | 35 | 33 | 9 | 12.473858816158100 | 4.593245983542620 | 1.0309864158936600 | 4.455195444608450 | 8.38168027382532E-06 | 0.0006739131756231970 | 3.171396052688230 | up |
| **LP_3D1.tig00088.2** | 2 | 2 | 3 | 67 | 48 | 17 | 21.699615039309000 | 4.138403860815920 | 0.741587600156881 | 5.580465288174250 | 2.39876022588868E-08 | 3.06421404585712E-06 | 5.513680901021220 | up |
| **LP_3D1.tig27855.1** | 2 | 0 | 1 | 25 | 21 | 6 | 8.606340860225600 | 4.003330051339020 | 1.0758029597623200 | 3.7212484080016700 | 0.00019824031076401700 | 0.010877384619093200 | 1.9634755147515800 | up |
| **LP_3D1.tig09902.1** | 0 | 1 | 1 | 119 | 92 | 44 | 40.321171398446800 | 6.907505882028150 | 1.1014490217568500 | 6.271289678945310 | 3.5806973270637E-10 | 6.00426960344944E-08 | 7.221539815319240 | up |
| **LP_3D3.tig11685.1** | 0 | 2 | 4 | 38 | 39 | 26 | 17.386767989559300 | 4.045980746635940 | 0.7659914050565610 | 5.2820184664306800 | 1.27768316147607E-07 | 1.45550900429072E-05 | 4.836985103518420 | up |
| **LP_3D1.tig01935.2** | 1 | 0 | 0 | 44 | 26 | 18 | 14.024801503857000 | 6.268080844759110 | 1.4931281051851900 | 4.197952488464930 | 2.69339119161383E-05 | 0.001897222085344340 | 2.721881828418570 | up |
| **LP_3D1.tig00197.4** | 5 | 7 | 12 | 109 | 69 | 40 | 38.15458330837270 | 3.1021630385616700 | 0.507669189320298 | 6.110599389959150 | 9.92576389071028E-10 | 1.55435275032349E-07 | 6.808450414020260 | up |
| **C0_3D2.tig18073.1** | 6 | 27 | 6 | 2 | 0 | 1 | 6.910701961461710 | -3.7482076322052400 | 1.1512121778501900 | -3.2558790675796800 | 0.0011304188795456700 | 0.04882876991472180 | 1.31132421624378 | down |
| **LP_3D2.tig26313.1** | 0 | 0 | 1 | 59 | 25 | 14 | 15.36094483931650 | 6.404738566619100 | 1.507061124953790 | 4.24982003753531 | 2.13942332464542E-05 | 0.001549525691493050 | 2.809801218662410 | up |
| **C0_3D1.tig06100.1** | 105 | 151 | 214 | 1559 | 1267 | 715 | 636.3270204390040 | 2.8425158061147800 | 0.31431027306358300 | 9.043661788107590 | 1.51509507842078E-19 | 7.9014293332871E-17 | 16.102294339670300 | up |
| **LP_3D3.tig01737.1** | 29 | 12 | 38 | 128 | 58 | 143 | 67.03839177728960 | 2.01619804883106 | 0.4594201110400810 | 4.388571593582600 | 1.14097575080279E-05 | 0.0008872608283773510 | 3.051948691697660 | up |
| **LP_3D3.tig15987.1** | 2 | 5 | 15 | 338 | 285 | 182 | 131.1160167331800 | 5.130610144155900 | 0.4627357688458710 | 11.08755901224660 | 1.44168108398369E-28 | 2.04880903047632E-25 | 24.688498520308200 | up |
| **C0_3D2.tig06045.2** | 14 | 32 | 36 | 9 | 2 | 0 | 15.148947667382600 | -2.980357547626550 | 0.741444985255759 | -4.01966107653757 | 5.82819235361935E-05 | 0.0037562765798355100 | 2.425242437525720 | down |
| **LP_3D1.tig09882.2** | 1 | 0 | 0 | 33 | 21 | 10 | 10.17171138150990 | 5.799521166373930 | 1.5257710141006300 | 3.8010429564966300 | 0.00014408831723900200 | 0.008332350349390700 | 2.079232477609520 | up |
| **LP_3D1.tig03753.1** | 6 | 11 | 14 | 82 | 67 | 27 | 32.64701079127290 | 2.4238389135049800 | 0.507158441332083 | 4.779253810975950 | 1.75946967944998E-06 | 0.0001621021943733130 | 3.790211106078250 | up |
| **LP_3D1.tig01873.1** | 21 | 12 | 9 | 58 | 61 | 32 | 31.114700743219700 | 1.7484306646883600 | 0.47915054316374300 | 3.6490215645876100 | 0.0002632410085272480 | 0.013998068409477500 | 1.8539318883918300 | up |
| **LP_3D1.tig00051.7** | 2 | 5 | 0 | 218 | 135 | 64 | 66.21906763328000 | 5.796202023370480 | 0.6862880633587190 | 8.445727578305320 | 3.0215757673836E-17 | 1.24464836591972E-14 | 13.904953326729400 | up |
| **LP_3D2.tig09457.1** | 8 | 11 | 14 | 286 | 275 | 143 | 116.72849678073300 | 4.338586852966590 | 0.40623582880204500 | 10.679970956182500 | 1.26311171341322E-26 | 1.49586636143696E-23 | 22.825107203986900 | up |
| **LP_3D1.tig13517.1** | 4 | 7 | 12 | 208 | 91 | 54 | 58.653258327940900 | 3.8486227011846600 | 0.5304474639218970 | 7.255426716021270 | 4.00397516959712E-13 | 9.89591167459776E-11 | 10.004544189632600 | up |
| **LP_3D2.tig10806.1** | 3 | 5 | 9 | 51 | 41 | 20 | 20.389582911783600 | 2.6445775827671900 | 0.5938180513265520 | 4.453514972910260 | 8.44758141684604E-06 | 0.0006772958612702580 | 3.1692215782146600 | up |
| **LP_3D2.tig06926.1** | 23 | 11 | 23 | 66 | 58 | 58 | 38.93425320349090 | 1.6088552247503300 | 0.42383845244271800 | 3.795916145592690 | 0.00014709921710425400 | 0.008480583160538850 | 2.0715742828027000 | up |
| **LP_3D2.tig08662.1** | 2 | 2 | 4 | 39 | 36 | 17 | 15.815070916438300 | 3.444080191520170 | 0.7196821864168980 | 4.78555709245398 | 1.70513705704251E-06 | 0.00015773449167671300 | 3.8020733294550800 | up |
| **LP_3D1.tig06762.1** | 3 | 2 | 9 | 31 | 15 | 22 | 13.212281559526900 | 2.2286239439266700 | 0.685559853759561 | 3.2508087101440600 | 0.0011507725706905500 | 0.04948235006119850 | 1.3055496826408500 | up |
| **LP_3D3.tig23930.1** | 21 | 48 | 26 | 363 | 297 | 206 | 153.20523130504900 | 3.1207380285490100 | 0.34899758544774200 | 8.942004640362470 | 3.82173542554198E-19 | 1.88910043708638E-16 | 15.72374495149440 | up |
| **LP_3D1.tig03907.3** | 3 | 8 | 1 | 283 | 192 | 110 | 93.88961459664250 | 5.5180535523598700 | 0.5575893439756060 | 9.896267946973660 | 4.32099670670436E-23 | 3.23193497095538E-20 | 19.490537386152200 | up |
| **LP_3D1.tig00106.4** | 2 | 0 | 1 | 62 | 29 | 13 | 16.61716877197710 | 4.998424551417030 | 1.0097145242143500 | 4.950334407941960 | 7.40860705802606E-07 | 7.38846084585073E-05 | 4.131446023820860 | up |
| **C0_3D3.tig02520.2** | 300 | 518 | 641 | 4108 | 2812 | 1360 | 1536.395897688780 | 2.4252555312918900 | 0.3458218813677500 | 7.013019308378730 | 2.33229248981621E-12 | 5.28203850930686E-10 | 9.27719843684388 | up |
| **C0_90D1.tig00035.10** | 17 | 29 | 27 | 4 | 4 | 4 | 14.051718464125100 | -2.6516789879787500 | 0.6557836514297310 | -4.043527133068340 | 5.26530597235459E-05 | 0.0034324119036524800 | 2.4644006005583800 | down |
| **C0_3D3.tig11881.1** | 11 | 25 | 30 | 158 | 61 | 53 | 53.4106015390877 | 1.9669624299945400 | 0.46527102571650900 | 4.227562692014730 | 2.36236364976887E-05 | 0.001689164297749830 | 2.772328106417210 | up |
| **LP_3D1.tig00624.2** | 4 | 22 | 16 | 143 | 23 | 28 | 36.74896783176500 | 2.118629139818950 | 0.6297393288981040 | 3.3642954197033500 | 0.0007673935344224440 | 0.035122774125800200 | 1.4544111892367000 | up |
| **LP_3D1.tig11991.1** | 1 | 0 | 0 | 24 | 16 | 9 | 7.8705374581419400 | 5.421162550598920 | 1.5460705784933100 | 3.506413372073860 | 0.0004541892522540190 | 0.022296485244506600 | 1.6517635925223100 | up |
| **LP_3D2.tig21691.1** | 0 | 2 | 0 | 33 | 25 | 6 | 10.225275811530800 | 4.900649762222670 | 1.2260502410155500 | 3.9971035429701600 | 6.41222580188438E-05 | 0.004100145958471510 | 2.387200682833810 | up |
| **LP_3D1.tig03239.2** | 0 | 1 | 0 | 51 | 36 | 27 | 18.22682164172180 | 6.6503450953944200 | 1.4712329658671200 | 4.520252910099000 | 6.17657979519387E-06 | 0.0005125659539529870 | 3.290250244233320 | up |
| **LP_3D1.tig07322.1** | 1 | 2 | 0 | 238 | 194 | 132 | 89.95560257820200 | 7.477168751103650 | 0.8933333512437430 | 8.369964852083010 | 5.76354224323048E-17 | 2.29110880291214E-14 | 13.63995428602560 | up |
| **LP_3D1.tig00772.1** | 3 | 6 | 5 | 364 | 268 | 144 | 124.25281475599200 | 5.70683803032342 | 0.514185269119007 | 11.098797210004400 | 1.27143385471554E-28 | 1.85319634541807E-25 | 24.73207856491520 | up |
| **LP_3D1.tig00463.2** | 1 | 10 | 8 | 144 | 80 | 44 | 44.92264790553430 | 3.7401830672446600 | 0.5579109008648330 | 6.703907490330280 | 2.02918754681359E-11 | 4.07594226496885E-09 | 8.38977197632975 | up |
| **LP_3D1.tig21610.1** | 1 | 0 | 2 | 49 | 17 | 8 | 11.886222206503600 | 4.511413109724170 | 1.0640797817142500 | 4.2397320081171000 | 2.23786793381023E-05 | 0.0016164117242368800 | 2.7914480081719100 | up |
| **C0_3D2.tig11683.2** | 36 | 31 | 18 | 110 | 139 | 131 | 76.05736103278690 | 2.098400877912300 | 0.37460951194593800 | 5.601568596088200 | 2.12420693088389E-08 | 2.73191273724196E-06 | 5.563533177003520 | up |
| **C0_3D2.tig02160.3** | 41 | 48 | 50 | 243 | 74 | 104 | 89.60359352124390 | 1.5158401104631000 | 0.40502820902010300 | 3.742554411532030 | 0.00018215907294453700 | 0.010161759079030600 | 1.9930311058130400 | up |
| **LP_3D2.tig07936.2** | 410 | 379 | 483 | 6010 | 4214 | 2977 | 2300.8004212042500 | 3.291915036804950 | 0.27211488310921200 | 12.097519250660600 | 1.0885105872177E-33 | 2.47505537321561E-30 | 29.606415080361500 | up |
| **LP_3D1.tig00249.5** | 0 | 0 | 1 | 143 | 100 | 34 | 43.18954239996010 | 7.9032231451383200 | 1.464141309622860 | 5.397855448238160 | 6.74421687224402E-08 | 8.0036536138353E-06 | 5.0967117152451200 | up |
| **C0_3D2.tig05871.2** | 18 | 15 | 22 | 163 | 139 | 93 | 71.90807122330760 | 2.7680421688705200 | 0.3744966324376040 | 7.3913673157841200 | 1.45326538192604E-13 | 3.77218587377105E-11 | 10.423406915626100 | up |
| **LP_3D1.tig05042.1** | 0 | 1 | 0 | 31 | 12 | 4 | 7.370039963823590 | 5.331605285429830 | 1.5944270094344100 | 3.343900507130220 | 0.0008260935782828190 | 0.03747971938210430 | 1.4262036694187300 | up |
| **LP_3D1.tig12098.1** | 1 | 1 | 5 | 30 | 20 | 10 | 10.531414103009800 | 3.0211407665146300 | 0.8281666008411270 | 3.6479867256735700 | 0.000264303290079889 | 0.014041421051019900 | 1.8525889375490900 | up |
| **LP_3D1.tig10759.1** | 0 | 1 | 1 | 84 | 73 | 32 | 29.98821540169670 | 6.477265194160430 | 1.1119977918393500 | 5.824890338537810 | 5.71501135697914E-09 | 8.14210076660349E-07 | 6.08926352709675 | up |
| **LP_3D2.tig17435.1** | 3 | 8 | 1 | 87 | 46 | 16 | 25.01752921791240 | 3.524975199716030 | 0.6902862761032630 | 5.106541042094710 | 3.28109371557861E-07 | 3.52578019398991E-05 | 4.452744766203700 | up |
| **LP_3D1.tig01834.3** | 3 | 6 | 4 | 42 | 32 | 17 | 16.465599228418800 | 2.7269020200150200 | 0.639554770515894 | 4.2637505741930100 | 2.01023835113081E-05 | 0.0014706820987134000 | 2.8324811938226000 | up |
| **LP_3D1.tig00149.5** | 16 | 13 | 17 | 360 | 183 | 117 | 110.91483288243400 | 3.7425581531595700 | 0.42244709272517400 | 8.859235197990390 | 8.05652341617104E-19 | 3.78490143464663E-16 | 15.421945425793100 | up |
| **LP_3D1.tig00156.6** | 0 | 1 | 2 | 37 | 25 | 9 | 11.515394814562900 | 4.4757290473598600 | 1.0384792162353900 | 4.309887937463890 | 1.63337293869122E-05 | 0.0012233080988129400 | 2.9124641490446800 | up |
| **FM2.tig05929.1** | 3 | 4 | 12 | 251 | 98 | 42 | 63.326158830749500 | 4.261133590029700 | 0.5981179847709910 | 7.124235850659500 | 1.04659697475582E-12 | 2.41844735894286E-10 | 9.616463361229610 | up |
| **LP_3D1.tig01177.2** | 0 | 2 | 1 | 61 | 38 | 21 | 19.274683855761700 | 5.237325202306550 | 0.9737637283004860 | 5.378435291944250 | 7.51359771763879E-08 | 8.84286671344051E-06 | 5.053406920959490 | up |
| **LP_3D1.tig14934.1** | 4 | 6 | 2 | 38 | 28 | 13 | 14.388328948621700 | 2.6251872345146300 | 0.6830410073401080 | 3.843381592472190 | 0.00012135056088798500 | 0.007185596493414070 | 2.143537174217910 | up |
| **LP_3D1.tig10553.1** | 3 | 3 | 4 | 122 | 99 | 39 | 42.31685301259390 | 4.606093827581410 | 0.61811510269127 | 7.451838351023140 | 9.20484685229529E-14 | 2.46815810999399E-11 | 10.607627022880900 | up |
| **FM1.tig04406.1** | 9 | 17 | 23 | 254 | 165 | 78 | 85.68150035169720 | 3.258560793115280 | 0.43934501127506900 | 7.416860802990040 | 1.19928872449075E-13 | 3.17086360668263E-11 | 10.498822438559200 | up |
| **LP_3D1.tig04292.1** | 0 | 0 | 1 | 27 | 10 | 7 | 6.996954092665430 | 5.253504639441750 | 1.5782650350446700 | 3.3286580661612700 | 0.0008726547030632580 | 0.0390291554646978 | 1.4086108461307400 | up |
| **LP_3D1.tig11150.1** | 0 | 0 | 1 | 62 | 40 | 19 | 19.05344635481490 | 6.717438468798100 | 1.4826645581262400 | 4.530652892443500 | 5.88016816841383E-06 | 0.0004908343018112830 | 3.3090650943419200 | up |
| **LP_3D2.tig02510.3** | 0 | 1 | 5 | 75 | 60 | 31 | 27.073648124240000 | 4.716818289674310 | 0.748648225009344 | 6.300446768060440 | 2.96788927867434E-10 | 5.02112101328103E-08 | 7.299199311633650 | up |
| **LP_3D1.tig06316.1** | 1 | 5 | 2 | 207 | 137 | 83 | 68.44020379516390 | 5.65500000286909 | 0.631382735061233 | 8.956532525902300 | 3.35048639078322E-19 | 1.6706877095094E-16 | 15.777104722286100 | up |
| **LP_3D3.tig02462.1** | 0 | 1 | 1 | 25 | 15 | 12 | 8.541429648443100 | 4.629406913351610 | 1.2050717120824600 | 3.8416028415036300 | 0.0001222335026255720 | 0.007215330692368240 | 2.1417437595698600 | up |
| **LP_3D1.tig05846.2** | 4 | 21 | 11 | 575 | 241 | 154 | 156.77397812463700 | 4.66556179666353 | 0.4900794089945970 | 9.520011881819280 | 1.73159261881717E-21 | 1.15802802843132E-18 | 17.936280928997400 | up |
| **LP_3D1.tig17621.1** | 25 | 35 | 53 | 1129 | 565 | 287 | 326.57100268110300 | 4.038561684594610 | 0.4066753188875790 | 9.930678103706170 | 3.06166281770935E-23 | 2.38411264209162E-20 | 19.622673229349900 | up |
| **LP_3D2.tig20906.1** | 0 | 0 | 1 | 22 | 22 | 7 | 8.133542502746640 | 5.47426106019389 | 1.5501681912447200 | 3.531398135448970 | 0.00041336904296376700 | 0.020558148072856800 | 1.687016010200300 | up |
| **LP_3D1.tig01854.2** | 26 | 13 | 14 | 103 | 30 | 65 | 40.73290530975140 | 1.813943868056750 | 0.4976774312620440 | 3.644818418743300 | 0.0002675806577606270 | 0.01417578983262150 | 1.848452734171000 | up |
| **LP_3D1.tig00123.6** | 5 | 11 | 6 | 2692 | 1544 | 702 | 771.7519807916800 | 7.7093208264298400 | 0.4828986710434130 | 15.964676004951700 | 2.2520809382328E-57 | 4.26731803112811E-53 | 52.369844989196700 | up |
| **LP_3D1.tig00188.2** | 14 | 16 | 17 | 684 | 437 | 307 | 233.0748406659290 | 4.841032386628820 | 0.3694365974862380 | 13.103824633424800 | 3.13074753065882E-39 | 9.88707463223891E-36 | 35.004932187574700 | up |
| **LP_3D1.tig11212.2** | 16 | 3 | 10 | 676 | 1112 | 870 | 435.6725517925470 | 6.454398705234450 | 0.42476186557012500 | 15.1953346766928 | 3.79719285018436E-52 | 5.39628568921825E-48 | 47.26790506604860 | up |
| **LP_3D1.tig04764.2** | 0 | 5 | 2 | 55 | 21 | 15 | 15.290819700970900 | 3.6173237893967800 | 0.8066348060119290 | 4.484462810724890 | 7.30978529638287E-06 | 0.0005961617577803220 | 3.224635886266590 | up |
| **FM3.tig07970.2** | 7 | 15 | 25 | 380 | 291 | 125 | 132.2188135412280 | 4.0037444428298000 | 0.4371911264602160 | 9.157881302959470 | 5.29261864115217E-20 | 2.94959712408133E-17 | 16.530237298847900 | up |
| **LP_3D1.tig00778.4** | 10 | 9 | 8 | 65 | 44 | 47 | 29.539058789601500 | 2.46199444578695 | 0.47952145214818900 | 5.134273836462500 | 2.83235457173956E-07 | 3.08439072089149E-05 | 4.510830612080580 | up |
| **LP_3D2.tig03516.1** | 3 | 5 | 2 | 137 | 71 | 40 | 40.34465024308200 | 4.532973385794170 | 0.6316389809387710 | 7.176525709444120 | 7.15052105111312E-13 | 1.70337330176021E-10 | 9.768690164058380 | up |
| **LP_3D1.tig02383.3** | 2 | 0 | 1 | 50 | 23 | 7 | 12.817084943619100 | 4.608961174398090 | 1.0550027743379800 | 4.368672089312960 | 1.25004294245194E-05 | 0.0009628548924617970 | 3.0164391585157300 | up |
| **LP_3D1.tig00027.2** | 5 | 4 | 15 | 56 | 28 | 64 | 28.141691334943100 | 2.5988116229951600 | 0.565731807965112 | 4.593716645247260 | 4.35420524812817E-06 | 0.0003767348513391870 | 3.423964201819090 | up |
| **LP_3D2.tig00092.2** | 3 | 11 | 6 | 128 | 111 | 43 | 47.42597903472410 | 3.734435580394510 | 0.5298523392182570 | 7.048068497544600 | 1.81418335637361E-12 | 4.15835697149427E-10 | 9.381078231672250 | up |
| **C0_3D2.tig04404.2** | 16 | 43 | 25 | 9 | 4 | 8 | 17.28530156560320 | -2.0395913750861000 | 0.6011854217756250 | -3.392616156696020 | 0.0006922857317029880 | 0.03194235585929900 | 1.4956330562951300 | down |
| **LP_3D1.tig01448.1** | 4 | 26 | 25 | 899 | 489 | 213 | 257.4452315106510 | 4.790710723873380 | 0.8126277903613740 | 5.89533213199976 | 3.73928482832742E-09 | 5.46425825363168E-07 | 6.262468783067980 | up |
| **LP_3D1.tig03358.1** | 0 | 2 | 3 | 61 | 68 | 18 | 23.757654085307200 | 4.794029758592220 | 0.8239784080472130 | 5.81814973762945 | 5.95025883288428E-09 | 8.4349741485114E-07 | 6.073916244097510 | up |
| **LP_3D2.tig31828.1** | 0 | 1 | 0 | 42 | 19 | 5 | 10.282037305406000 | 5.820015355208740 | 1.5604937024957400 | 3.729598745512790 | 0.00019178493723942200 | 0.010605072721181900 | 1.9744863490899300 | up |
| **LP_3D1.tig02076.1** | 0 | 1 | 0 | 29 | 8 | 14 | 8.237936331018520 | 5.488944524733210 | 1.5554954374688600 | 3.5287435710290300 | 0.00041753750752826900 | 0.020693042384868800 | 1.6841756526970300 | up |
| **LP_3D3.tig04298.1** | 2 | 1 | 5 | 84 | 91 | 51 | 37.216118759818500 | 4.749421953250970 | 0.6428689014619240 | 7.387854572604910 | 1.49216812362312E-13 | 3.85555895397075E-11 | 10.413912651847000 | up |
| **LP_3D1.tig01877.1** | 0 | 0 | 2 | 51 | 23 | 11 | 13.480156743582500 | 5.312564319763190 | 1.1927741856323500 | 4.453956485440470 | 8.43021931966495E-06 | 0.0006768584989072800 | 3.169502113540660 | up |
| **LP_3D2.tig00279.6** | 9 | 29 | 19 | 716 | 376 | 193 | 209.37219162892300 | 4.406589736537840 | 0.4398892940599010 | 10.017497120395400 | 1.27695710427868E-23 | 1.06747980283414E-20 | 19.97164033325910 | up |
| **FM1.tig03000.2** | 4 | 5 | 0 | 19 | 24 | 29 | 13.320625281802600 | 2.9514786653070600 | 0.7435922659715760 | 3.969216464954310 | 7.21093535400518E-05 | 0.004559573083408500 | 2.341075818782840 | up |
| **LP_3D1.tig10343.2** | 0 | 1 | 3 | 82 | 48 | 21 | 24.122379103717700 | 5.147738122291600 | 0.8827900026440540 | 5.831214792729370 | 5.50252988185837E-09 | 7.91876737048707E-07 | 6.101342415109700 | up |
| **LP_3D1.tig00393.4** | 1 | 0 | 0 | 41 | 23 | 9 | 11.495685362601500 | 5.979805153016710 | 1.5265427733830000 | 3.917220832119080 | 8.95756855997544E-05 | 0.005492912457301020 | 2.260197322444880 | up |
| **LP_3D1.tig19434.1** | 0 | 1 | 0 | 59 | 14 | 14 | 13.639844843363600 | 6.230873702453590 | 1.5298307240745 | 4.072917091022 | 4.64279549362464E-05 | 0.003068008902880500 | 2.5131433844667800 | up |
| **LP_3D3.tig03347.1** | 0 | 1 | 4 | 32 | 13 | 14 | 10.107629254614500 | 3.496854724277310 | 0.911866518053169 | 3.8348318038292300 | 0.00012565024782082800 | 0.007401645945466260 | 2.1306716930328400 | up |
| **LP_3D2.tig12092.1** | 0 | 10 | 12 | 276 | 250 | 150 | 110.64601326345300 | 4.905631666007110 | 0.9339200292041370 | 5.252732046219810 | 1.49859479315029E-07 | 1.69359087508208E-05 | 4.771191494922620 | up |
| **C0_3D3.tig08612.2** | 3 | 10 | 20 | 51 | 32 | 51 | 27.107427536490100 | 1.9996146531188000 | 0.5411510478473710 | 3.695113704524850 | 0.00021978840662399100 | 0.011944428273939600 | 1.9228346331401900 | up |
| **LP_3D1.tig11837.2** | 0 | 1 | 0 | 44 | 28 | 11 | 13.064910538163800 | 6.168616335637350 | 1.5118776336815100 | 4.080102911911200 | 4.50157667657629E-05 | 0.0029893939974296600 | 2.5244168418583400 | up |
| **LP_3D1.tig00019.2** | 1 | 9 | 7 | 228 | 107 | 46 | 61.68211786402000 | 4.393770277165840 | 0.5896194122377300 | 7.451875202837300 | 9.20227541549313E-14 | 2.46815810999399E-11 | 10.607627022880900 | up |
| **LP_3D1.tig02076.2** | 2 | 2 | 4 | 325 | 100 | 62 | 76.4431292527107 | 5.815700994009980 | 0.6957807612234110 | 8.358525153503920 | 6.35071767021702E-17 | 2.48970031698956E-14 | 13.60385292539840 | up |
| **LP_3D1.tig01398.2** | 1 | 2 | 2 | 153 | 97 | 58 | 49.1739635921867 | 5.857687902787380 | 0.7550886122673800 | 7.757616533505810 | 8.65403482181147E-15 | 2.68818912265504E-12 | 11.570540180545200 | up |
| **LP_3D1.tig00429.2** | 1 | 7 | 11 | 79 | 81 | 41 | 34.867168592977600 | 3.3436728286590100 | 0.543896512146646 | 6.147626899577330 | 7.86507706011815E-10 | 1.2453768954942E-07 | 6.9046991956665300 | up |
| **LP_3D1.tig04687.3** | 5 | 20 | 22 | 278 | 99 | 83 | 79.40809323248220 | 3.2144653426002500 | 0.4933198193224080 | 6.51598662104318 | 7.22134117618202E-11 | 1.34149391882375E-08 | 7.872411291704140 | up |
| **LP_3D1.tig15444.1** | 2 | 4 | 4 | 56 | 23 | 7 | 14.839342826683500 | 2.99466788884867 | 0.7710103769686550 | 3.884082469321190 | 0.00010271704144613600 | 0.006204672107659630 | 2.2072811643017100 | up |
| **C0_3D1.tig06650.1** | 224 | 274 | 273 | 29 | 79 | 69 | 157.8575504164560 | -2.153569261127420 | 0.36338878720982800 | -5.9263503358564200 | 3.09741418887885E-09 | 4.56146397841498E-07 | 6.340895750381990 | down |
| **LP_3D3.tig27700.1** | 3 | 3 | 2 | 54 | 25 | 12 | 15.450059278182900 | 3.3989358965861700 | 0.7597393725856810 | 4.473818284576070 | 7.68350458870059E-06 | 0.0006239554547781220 | 3.2048464142191800 | up |
| **FM3.tig27450.1** | 0 | 1 | 1 | 42 | 20 | 4 | 10.419535310444500 | 4.932910722395680 | 1.2475657818726600 | 3.954028552298970 | 7.68462330865684E-05 | 0.0048268774804485900 | 2.316333724641100 | up |
| **LP_3D1.tig18660.1** | 7 | 32 | 27 | 318 | 264 | 172 | 130.2719221956660 | 3.4579948539435600 | 0.3981780470937300 | 8.68454421127231 | 3.80264034367841E-18 | 1.71556420901904E-15 | 14.765593022812800 | up |
| **LP_3D1.tig00393.3** | 0 | 2 | 1 | 86 | 51 | 27 | 26.105009212295600 | 5.683630235669030 | 0.9588520988098460 | 5.9275358970624500 | 3.07514140711613E-09 | 4.54042631915626E-07 | 6.342903367547150 | up |
| **C0_3D3.tig32987.1** | 9 | 11 | 8 | 107 | 26 | 49 | 33.467321649090800 | 2.6178196224138900 | 0.5443328848082080 | 4.809225559349140 | 1.5151616010312E-06 | 0.0001430720285890680 | 3.8444452650300700 | up |
| **C0_3D1.tig00786.1** | 89 | 137 | 86 | 37 | 20 | 41 | 68.13462086209880 | -1.7124577991004900 | 0.3880158405069890 | -4.41337084811526 | 1.01773441660395E-05 | 0.0008012896525187200 | 3.0962104654993700 | down |
| **LP_3D1.tig02400.1** | 0 | 2 | 3 | 51 | 33 | 15 | 16.24878211368270 | 4.224785739456610 | 0.8452693187667200 | 4.998153423598450 | 5.78819248560947E-07 | 5.9178021914473E-05 | 4.2278395556672200 | up |
| **LP_3D1.tig00028.4** | 3 | 9 | 14 | 146 | 85 | 28 | 44.296368524242800 | 3.226254821713830 | 0.5654278276715970 | 5.705864946547440 | 1.1575375475596E-08 | 1.58044067857075E-06 | 5.801221800648750 | up |
| **LP_90D2.tig00322.4** | 66 | 168 | 74 | 26 | 30 | 17 | 62.71150777654110 | -2.1346233585632000 | 0.42602993315897000 | -5.010500888365230 | 5.42885477468695E-07 | 5.58052892707196E-05 | 4.253324636327250 | down |
| **LP_3D1.tig12544.1** | 0 | 3 | 0 | 29 | 18 | 5 | 8.516822789441700 | 4.018039879287130 | 1.1135518905320600 | 3.6083095125161200 | 0.0003081986780263070 | 0.016028869032392900 | 1.7950971195871400 | up |
| **LP_3D1.tig11571.1** | 0 | 4 | 4 | 39 | 23 | 9 | 12.306896038723700 | 3.0687441975287800 | 0.806786730649403 | 3.803662208299670 | 0.00014257256188454300 | 0.008253092953489670 | 2.0833832634620500 | up |
| **LP_3D1.tig06712.2** | 1 | 2 | 4 | 69 | 32 | 9 | 18.035199920109300 | 3.867502318026540 | 0.8119414723674600 | 4.763277218430130 | 1.90473880375295E-06 | 0.0001740753654330160 | 3.7592626844455000 | up |
| **LP_3D1.tig02054.3** | 6 | 16 | 12 | 387 | 249 | 99 | 119.92060805286300 | 4.341508001069340 | 0.4695721289772330 | 9.245667988271580 | 2.33775875855348E-20 | 1.36119316908034E-17 | 16.86608023900480 | up |
| **C0_3D3.tig12474.1** | 37 | 40 | 36 | 6 | 9 | 13 | 23.597841954809700 | -2.0532079546238100 | 0.5114540550860860 | -4.014452391580360 | 5.95839684659746E-05 | 0.003831505302543360 | 2.4166305690727800 | down |
| **LP_3D2.tig06311.1** | 3 | 0 | 0 | 23 | 16 | 6 | 7.546696551277180 | 3.778254015072590 | 1.1083937875066400 | 3.408765059547910 | 0.0006525765465143870 | 0.030454413641894500 | 1.516349757828660 | up |
| **LP_3D3.tig02273.1** | 74 | 74 | 179 | 17 | 56 | 37 | 72.01224369829710 | -1.5971825115769900 | 0.45696185261046600 | -3.495220667661510 | 0.00047366996623495800 | 0.023112248266631900 | 1.6361578060440400 | down |
| **LP_3D1.tig00185.4** | 14 | 15 | 22 | 218 | 111 | 64 | 69.77876424759150 | 2.8512151761391100 | 0.44238787409748500 | 6.445057251977950 | 1.15556786854602E-10 | 2.05919296199055E-08 | 7.686302954789870 | up |
| **LP_3D1.tig03907.1** | 2 | 9 | 9 | 450 | 330 | 210 | 159.53177878379000 | 5.557162401685640 | 0.4589870069527740 | 12.107450358082600 | 9.64456554395686E-34 | 2.28435553477595E-30 | 29.64123630201340 | up |
| **LP_3D1.tig08843.2** | 585 | 838 | 641 | 4285 | 1401 | 1889 | 1540.0840394498400 | 1.793853980177510 | 0.34839039165507100 | 5.14897661687967 | 2.6191152676873E-07 | 2.85765081365998E-05 | 4.543990840263580 | up |
| **LP_3D1.tig25857.2** | 0 | 1 | 1 | 25 | 12 | 9 | 7.533747298689560 | 4.443852002729020 | 1.2332479582758500 | 3.603372681793680 | 0.0003141146433107450 | 0.01626215564571890 | 1.7888218866023800 | up |
| **C0_90D1.tig00006.20** | 76 | 144 | 62 | 31 | 22 | 12 | 57.29891971558680 | -2.1961042863158500 | 0.43429675706364400 | -5.056690501591790 | 4.26594715802294E-07 | 4.48239863581912E-05 | 4.348489522714400 | down |
| **LP_3D1.tig04687.2** | 1 | 2 | 1 | 34 | 27 | 6 | 11.028650873150100 | 3.9620130431614600 | 0.9662551572558580 | 4.100379711724890 | 4.12472791700435E-05 | 0.00277479477446287 | 2.5567691320419300 | up |
| **LP_3D2.tig03924.3** | 4 | 1 | 0 | 30 | 22 | 8 | 10.2300259100101 | 3.45995622212961 | 0.9187850034805450 | 3.765795272041430 | 0.00016601979097918500 | 0.009381108368003770 | 2.027745847157090 | up |
| **LP_3D2.tig01068.4** | 5 | 6 | 5 | 197 | 166 | 98 | 75.55249043308060 | 4.767368546577960 | 0.49089789888918900 | 9.711527707422750 | 2.69270522604786E-22 | 1.91333535718363E-19 | 18.718208902942000 | up |
| **C0_3D1.tig08802.2** | 19 | 13 | 12 | 83 | 46 | 30 | 32.40418201014880 | 1.750006659740030 | 0.48002667726645900 | 3.6456445914747000 | 0.0002667223937280280 | 0.014156708189981100 | 1.8490377198895800 | up |
| **LP_3D1.tig00271.1** | 4 | 14 | 15 | 151 | 115 | 41 | 53.22384100827380 | 3.1361484674665200 | 0.5056897589822360 | 6.201724301829670 | 5.58478509692772E-10 | 9.07048882385303E-08 | 7.04236930745132 | up |
| **C0_3D3.tig14076.1** | 104 | 247 | 320 | 1683 | 1430 | 690 | 708.2056144301340 | 2.439194096660980 | 0.3651901348574430 | 6.679244217846020 | 2.40177634917402E-11 | 4.77374043946844E-09 | 8.321141198394010 | up |
| **LP_3D1.tig10728.1** | 0 | 2 | 1 | 53 | 34 | 17 | 16.74129463685640 | 5.029556985064550 | 0.9878339580900500 | 5.091500392220830 | 3.55241137839565E-07 | 3.81012876990379E-05 | 4.419060346341800 | up |
| **LP_3D1.tig00205.1** | 1 | 2 | 1 | 92 | 88 | 27 | 32.975974378989900 | 5.597695142914860 | 0.8556642581362440 | 6.541929372049990 | 6.07301843810785E-11 | 1.14311501031205E-08 | 7.941910072464630 | up |
| **LP_3D1.tig01278.4** | 0 | 3 | 5 | 30 | 26 | 9 | 11.435975767748000 | 2.950527785108590 | 0.8074166597740080 | 3.6542815278724000 | 0.0002579031599444770 | 0.013752819068521400 | 1.8616082705308400 | up |
| **LP_3D1.tig03277.1** | 10 | 23 | 25 | 428 | 266 | 152 | 142.12263954827700 | 3.787578480832180 | 0.40510424645137700 | 9.349639047258890 | 8.79473445895361E-21 | 5.49380967383756E-18 | 17.260126390243800 | up |
| **C0_3D1.tig22617.1** | 20 | 30 | 16 | 2 | 2 | 2 | 12.02846343453270 | -3.5184600027489500 | 0.8070794636487070 | -4.3594963832215800 | 1.30362108269801E-05 | 0.0009973666278057690 | 3.001145167501530 | down |
| **LP_3D1.tig00327.3** | 0 | 1 | 0 | 41 | 18 | 14 | 11.589506610232500 | 5.992305529691880 | 1.515771725693200 | 3.9533034085006700 | 7.70795964835522E-05 | 0.004830859605410720 | 2.3159755838095700 | up |
| **LP_3D2.tig00647.5** | 2 | 4 | 10 | 57 | 39 | 26 | 21.86013364005370 | 2.864487218217290 | 0.598429324672568 | 4.786675886554520 | 1.69566338836991E-06 | 0.00015749997599981600 | 3.8027195080531000 | up |
| **LP_3D1.tig02400.2** | 0 | 2 | 2 | 40 | 32 | 12 | 13.758455567671400 | 4.309204407464340 | 0.9196950108484630 | 4.685471114482720 | 2.79316691655258E-06 | 0.0002473170924788650 | 3.6067458678168900 | up |
| **LP_3D1.tig01448.2** | 0 | 1 | 0 | 48 | 23 | 7 | 12.159555333073000 | 6.064789109854890 | 1.5384975406236100 | 3.942020672582020 | 8.07980105469551E-05 | 0.005019631595127500 | 2.299328155778160 | up |
| **C0_90D1.tig06815.1** | 6 | 1 | 0 | 12 | 19 | 29 | 11.20879359598780 | 3.041747195905820 | 0.8584051886055910 | 3.5434864983131000 | 0.0003948735293122530 | 0.019771396352786300 | 1.703962657601100 | up |
| **LP_3D1.tig09861.1** | 1 | 1 | 10 | 66 | 53 | 18 | 23.289961755898500 | 3.4350366101122900 | 0.696165432983967 | 4.934224607201080 | 8.04697961955835E-07 | 7.94357492961059E-05 | 4.099984003518680 | up |
| **LP_3D1.tig00406.4** | 0 | 1 | 0 | 240 | 82 | 31 | 54.260499218986400 | 8.230742295372650 | 1.7193063973043300 | 4.787245780203860 | 1.69085713922642E-06 | 0.0001573105958745100 | 3.803242023881170 | up |
| **LP_3D1.tig02050.2** | 3 | 3 | 3 | 30 | 36 | 12 | 13.764179177431000 | 3.027887595394660 | 0.7254750133203870 | 4.17366213832298 | 2.99742166807612E-05 | 0.002090655640758120 | 2.679717495462860 | up |
| **LP_3D2.tig16465.1** | 4 | 6 | 1 | 153 | 96 | 45 | 47.721141247237200 | 4.6371083069505200 | 0.6113078545115470 | 7.585553289930670 | 3.31071213045625E-14 | 9.50492076039321E-12 | 11.022051499363200 | up |
| **LP_3D3.tig01055.4** | 9 | 6 | 4 | 30 | 41 | 21 | 17.89542021663980 | 2.191467028423630 | 0.5858082476270340 | 3.740928942705610 | 0.00018334135468674400 | 0.010197690124430500 | 1.9914981890046100 | up |
| **LP_3D1.tig07344.1** | 4 | 0 | 0 | 142 | 90 | 60 | 46.69840218793040 | 6.088766607144210 | 0.8237605238798110 | 7.391428006851880 | 1.45260207905558E-13 | 3.77218587377105E-11 | 10.423406915626100 | up |
| **LP_3D3.tig05275.1** | 3 | 6 | 5 | 95 | 93 | 37 | 37.65010265390630 | 3.922975631920010 | 0.5650092676431990 | 6.943205813744910 | 3.83300349946649E-12 | 8.51121421590519E-10 | 9.070008478734300 | up |
| **LP_3D3.tig03814.1** | 9 | 18 | 16 | 36 | 91 | 97 | 44.111680281112100 | 2.3685275578050900 | 0.4891673008354360 | 4.841958065798660 | 1.28565900014055E-06 | 0.0001226229628573650 | 3.911428194512330 | up |
| **LP_3D1.tig21779.1** | 0 | 1 | 1 | 24 | 12 | 9 | 7.385020704618660 | 4.413600128680630 | 1.2342917326022800 | 3.575816002085150 | 0.0003491371579398300 | 0.017891389751679000 | 1.747355923376540 | up |
| **LP_3D1.tig13450.1** | 4 | 2 | 2 | 19 | 19 | 24 | 11.473823998990100 | 2.899937777649220 | 0.7543754673141930 | 3.8441570587838600 | 0.00012096751779467000 | 0.007172551790707190 | 2.1443263071688000 | up |
| **LP_3D1.tig00942.2** | 13 | 14 | 18 | 116 | 203 | 204 | 93.12914037932360 | 3.5071260252025900 | 0.4062360247872350 | 8.633222587877180 | 5.96472040963325E-18 | 2.62840722236901E-15 | 14.580307348120300 | up |
| **LP_3D1.tig00772.2** | 7 | 12 | 13 | 695 | 534 | 290 | 244.30402170124500 | 5.486998225086890 | 0.4067385786388300 | 13.490233071692900 | 1.78535262995402E-41 | 7.80679771151818E-38 | 37.10752707385800 | up |
| **LP_3D2.tig07840.1** | 49 | 70 | 47 | 4 | 13 | 6 | 31.48172800404250 | -2.9080047944373400 | 0.5143039191906100 | -5.654253615282250 | 1.56524948769148E-08 | 2.06442243916061E-06 | 5.685201429029200 | down |
| **FM3.tig20166.1** | 0 | 8 | 13 | 220 | 133 | 79 | 71.09643199586770 | 4.314514522503350 | 1.0415157397552000 | 4.142534152693130 | 3.43489197786733E-05 | 0.002361020973178580 | 2.626900155024080 | up |
| **LP_3D1.tig04264.2** | 0 | 2 | 0 | 120 | 81 | 31 | 36.419080976438200 | 6.759857977511680 | 1.1216342173095700 | 6.026793649115260 | 1.67244308718852E-09 | 2.56946019706031E-07 | 6.590158105486480 | up |
| **LP_3D1.tig00188.4** | 2 | 8 | 9 | 113 | 108 | 40 | 43.99883261964210 | 3.703093181057510 | 0.5392150049311870 | 6.867563304418960 | 6.53077751349556E-12 | 1.41156672150059E-09 | 8.850298588934220 | up |
| **LP_3D1.tig17502.1** | 0 | 0 | 1 | 19 | 19 | 16 | 8.830188389358770 | 5.588458128562450 | 1.5222491977106500 | 3.6711848079585800 | 0.0002414286836914490 | 0.012971657395501300 | 1.8870045303358000 | up |
| **LP_3D1.tig01398.1** | 4 | 10 | 15 | 246 | 207 | 84 | 88.82405245093550 | 4.131611529907250 | 0.4708944975397920 | 8.773964341254860 | 1.72483870495258E-18 | 7.95536155330325E-16 | 15.099340077860700 | up |
| **FM1.tig10521.1** | 2 | 2 | 7 | 98 | 47 | 18 | 26.97275090822070 | 3.7887131419772900 | 0.6875688708490620 | 5.510303480288020 | 3.58215546352906E-08 | 4.40752440095908E-06 | 5.355805274678660 | up |
| **LP_3D1.tig07213.1** | 6 | 8 | 11 | 382 | 375 | 224 | 159.86073759108200 | 5.22267668447048 | 0.4134445451574750 | 12.632109301336300 | 1.40461859950223E-36 | 3.9922772144352E-33 | 32.3987793099399 | up |
| **LP_3D1.tig00027.7** | 9 | 3 | 14 | 61 | 27 | 42 | 25.188586487355100 | 2.257909417206560 | 0.5557569785704180 | 4.062763949477730 | 4.84950631351284E-05 | 0.0031906271573106100 | 2.49612394258903 | up |
| **LP_3D1.tig00371.4** | 2 | 8 | 7 | 765 | 161 | 106 | 160.7806333698790 | 5.820010583488540 | 0.978950181223898 | 5.94515501923936 | 2.76195347700566E-09 | 4.08862618230173E-07 | 6.38842259460217 | up |
| **LP_3D1.tig01660.2** | 0 | 1 | 1 | 59 | 28 | 8 | 14.918204336133200 | 5.461122967364210 | 1.1989608838479100 | 4.554880013964630 | 5.24154880996427E-06 | 0.0004460416797940400 | 3.3506245572995000 | up |
| **LP_3D1.tig02383.1** | 0 | 0 | 2 | 70 | 39 | 18 | 20.067386882348100 | 5.894683681047650 | 1.151406669568110 | 5.119549709798640 | 3.0626603619186E-07 | 3.30354702605812E-05 | 4.481019506621010 | up |
| **FM2.tig32971.1** | 0 | 4 | 7 | 222 | 139 | 66 | 68.39431486081060 | 5.196491691061200 | 0.6057601780882330 | 8.578463687496290 | 9.61486595788908E-18 | 4.17219126241378E-15 | 14.37963579077600 | up |
| **LP_3D1.tig22862.2** | 6 | 1 | 5 | 31 | 52 | 19 | 18.213227121803400 | 2.9991869045497600 | 0.667057742996799 | 4.496142854253850 | 6.91972549274519E-06 | 0.000565973806669209 | 3.2472036675436300 | up |
| **LP_3D1.tig01952.3** | 2 | 3 | 8 | 85 | 56 | 25 | 28.026414284010400 | 3.5895565701157600 | 0.6218233634457330 | 5.772630591145400 | 7.80434155536479E-09 | 1.08734753851645E-06 | 5.963631624325330 | up |
| **LP_3D1.tig00319.2** | 3 | 1 | 2 | 103 | 38 | 23 | 26.420951578165800 | 4.657047158176910 | 0.7824444071113550 | 5.951920821275850 | 2.6501358533074E-09 | 3.94363802568741E-07 | 6.404102954446070 | up |
| **LP_3D1.tig36122.2** | 27 | 29 | 30 | 177 | 97 | 79 | 70.08643918678900 | 1.9533420643316200 | 0.37651722525509100 | 5.187922180740140 | 2.12653425944758E-07 | 2.35639064285181E-05 | 4.627752710501440 | up |
| **FM1.tig00358.5** | 4 | 5 | 7 | 181 | 115 | 59 | 58.16910227689290 | 4.380360335767850 | 0.5323704570423300 | 8.228030458533810 | 1.90316695611844E-16 | 7.07437660561006E-14 | 13.150311824167200 | up |
| **LP_3D1.tig03195.2** | 0 | 1 | 0 | 39 | 40 | 18 | 15.455961959082400 | 6.411353876564340 | 1.4855572135410000 | 4.3157906125218500 | 1.59032673663327E-05 | 0.0011946126758118400 | 2.9227728813469200 | up |
| **LP_3D1.tig31718.1** | 2 | 11 | 6 | 82 | 39 | 18 | 24.64174604021760 | 2.781931992751160 | 0.6258887075502560 | 4.444771025890070 | 8.7985525773754E-06 | 0.000701477869931143 | 3.15398602544587 | up |
| **LP_3D1.tig00026.7** | 18 | 23 | 25 | 629 | 469 | 303 | 232.33437331008200 | 4.328749628531150 | 0.3422145261026920 | 12.649228183938000 | 1.12979235086279E-36 | 3.38016032551555E-33 | 32.4710627000669 | up |
| **LP_3D1.tig00051.6** | 0 | 8 | 3 | 135 | 98 | 40 | 44.38132249768570 | 4.551308074737460 | 0.6325310169353340 | 7.195391139534840 | 6.22819612952294E-13 | 1.49384729528579E-10 | 9.825693794892950 | up |
| **LP_3D1.tig04541.2** | 0 | 0 | 1 | 42 | 25 | 11 | 12.29496573546650 | 6.0801632520762000 | 1.5149644233112300 | 4.013403323879330 | 5.98495211729642E-05 | 0.0038442328034770000 | 2.415190319701740 | up |
| **LP_3D2.tig01151.4** | 1 | 12 | 13 | 139 | 96 | 42 | 47.45258455241030 | 3.341393625060340 | 0.542823358678808 | 6.15558186956627 | 7.48023410002761E-10 | 1.18774834473762E-07 | 6.925275566147080 | up |
| **LP_3D1.tig00188.1** | 2 | 4 | 6 | 55 | 41 | 13 | 18.906246095656200 | 3.093026413967810 | 0.6745144374926180 | 4.585559985143630 | 4.52771254903999E-06 | 0.0003905643898874950 | 3.4083073564920200 | up |
| **LP_3D1.tig12486.2** | 1 | 2 | 8 | 40 | 44 | 17 | 17.676109651076000 | 3.130993972431980 | 0.6913607010300250 | 4.528741607336460 | 5.93360068230485E-06 | 0.0004945682269583860 | 3.3057737878936100 | up |
| **LP_3D2.tig06585.2** | 3 | 3 | 4 | 70 | 81 | 30 | 30.14985703631930 | 4.092917590419320 | 0.6294210313197020 | 6.502670528561360 | 7.89064916682305E-11 | 1.45631153210408E-08 | 7.836745711423070 | up |
| **LP_3D1.tig00078.6** | 0 | 5 | 0 | 77 | 56 | 23 | 25.160412026086500 | 4.888753896020480 | 1.3254241831161200 | 3.6884447698297200 | 0.00022562898887760100 | 0.012203501306134400 | 1.9135155480321900 | up |
| **LP_3D1.tig00088.5** | 1 | 3 | 3 | 169 | 123 | 49 | 54.337001126878900 | 5.513413712329150 | 0.6860791988377470 | 8.03611845639564 | 9.27291026737981E-16 | 3.23385634447365E-13 | 12.490279276386400 | up |
| **LP_3D1.tig01834.1** | 0 | 7 | 2 | 90 | 66 | 33 | 31.09775901994810 | 4.315610250683150 | 0.6700215621728150 | 6.4410020428119500 | 1.18687275136913E-10 | 2.10180004833577E-08 | 7.677408602311050 | up |
| **LP_3D2.tig06858.4** | 0 | 1 | 1 | 43 | 40 | 16 | 15.852472223820000 | 5.546311633987930 | 1.1500690121901200 | 4.822590275192170 | 1.41705860371768E-06 | 0.000134478624922089 | 3.871346740316670 | up |
| **C0_3D1.tig04125.2** | 12 | 30 | 45 | 7 | 8 | 5 | 17.440418187308800 | -2.159982679666030 | 0.6131316252575830 | -3.522869463402090 | 0.00042690156528907700 | 0.02102878637682630 | 1.677185790797100 | down |
| **LP_3D1.tig03826.1** | 3 | 16 | 6 | 173 | 118 | 62 | 59.43273340756950 | 3.7409238585617400 | 0.5081402461044520 | 7.361990881928240 | 1.8118735102442E-13 | 4.66045021220956E-11 | 10.331572127251200 | up |
| **LP_3D2.tig20209.1** | 3 | 6 | 12 | 64 | 37 | 14 | 21.263558000919100 | 2.3664698538451900 | 0.6221197803660880 | 3.8038813883278800 | 0.00014244640611051000 | 0.008253092953489670 | 2.0833832634620500 | up |
| **LP_3D1.tig01003.5** | 2 | 4 | 5 | 95 | 71 | 22 | 31.008722431965300 | 3.999809387268100 | 0.6465648952312740 | 6.18624583049375 | 6.16138992747338E-10 | 9.95011961440978E-08 | 7.002171698393380 | up |
| **C0_3D2.tig22242.1** | 12 | 30 | 36 | 1330 | 809 | 396 | 408.3410018685970 | 4.9409823366652200 | 0.40755829767798700 | 12.12337563684960 | 7.94192854711149E-34 | 1.96286490548066E-30 | 29.70710958976490 | up |
| **C0_3D2.tig11730.1** | 40 | 59 | 40 | 17 | 17 | 15 | 31.116295075895900 | -1.561437339517000 | 0.4367677042811780 | -3.5749835077361600 | 0.0003502499739154460 | 0.017920755866087800 | 1.7466436765071500 | down |
| **LP_3D2.tig05109.1** | 3 | 10 | 9 | 197 | 108 | 44 | 57.71487519571030 | 3.89446597171456 | 0.5427413425811310 | 7.1755469247902400 | 7.20186898160681E-13 | 1.705792676081E-10 | 9.768073754590260 | up |
| **LP_3D1.tig23296.1** | 0 | 0 | 1 | 26 | 10 | 8 | 7.027436500142720 | 5.259023896506780 | 1.5722530491665400 | 3.3448966114548900 | 0.0008231324801141100 | 0.037402850385361000 | 1.4270952999529100 | up |
| **LP_3D2.tig13820.1** | 0 | 2 | 0 | 63 | 35 | 18 | 18.40443280259950 | 5.764860193556260 | 1.1518428087720700 | 5.0049018404707000 | 5.58905041735399E-07 | 5.7244967743151E-05 | 4.242262685013200 | up |
| **C0_3D2.tig08860.1** | 147 | 189 | 180 | 3544 | 3085 | 1778 | 1415.24744978554 | 3.9471622229083200 | 0.2750937257112880 | 14.348426932320800 | 1.08981204937727E-46 | 6.19503659468509E-43 | 42.2079561238605 | up |
| **LP_3D2.tig02564.4** | 2 | 0 | 7 | 26 | 11 | 21 | 10.83648267645890 | 2.64350364714137 | 0.8109795865751600 | 3.2596426481030500 | 0.0011155266813094600 | 0.048332404115119100 | 1.3157616019651500 | up |
| **C0_3D1.tig00750.3** | 98 | 165 | 215 | 904 | 342 | 358 | 331.16539808529300 | 1.6744449625422100 | 0.37023529283486800 | 4.522650851897710 | 6.10699325969036E-06 | 0.0005075322103027760 | 3.294536390236720 | up |
| **LP_3D1.tig02375.1** | 31 | 96 | 94 | 1666 | 915 | 407 | 500.3404375956050 | 3.675382579134360 | 0.4242997856797210 | 8.66223058125391 | 4.62621397454573E-18 | 2.0545088545551E-15 | 14.687291982668800 | up |
| **LP_3D1.tig19176.1** | 1 | 0 | 0 | 31 | 19 | 10 | 9.560887963295420 | 5.7083264325084700 | 1.5298866326895800 | 3.731208777524320 | 0.00019056321287885800 | 0.010558056370466500 | 1.9764160235893500 | up |
| **LP_3D1.tig05622.1** | 1 | 7 | 6 | 279 | 185 | 126 | 95.34039504478030 | 5.32384196034005 | 0.5196686174913960 | 10.244686288812100 | 1.25029819603303E-24 | 1.18455334922496E-21 | 20.9264453749984 | up |
| **C0_3D1.tig01042.2** | 136 | 157 | 148 | 570 | 475 | 484 | 319.7767792838240 | 1.7357237719310800 | 0.2541397137012780 | 6.829801398026660 | 8.50322681518328E-12 | 1.81335528411595E-09 | 8.74151709782696 | up |
| **LP_3D1.tig01387.2** | 4 | 8 | 11 | 509 | 246 | 115 | 138.64034133779900 | 5.143426037927670 | 0.5118238839836590 | 10.049210673591600 | 9.26061858444574E-24 | 7.85701288705699E-21 | 20.10474253452560 | up |
| **LP_3D1.tig05625.2** | 0 | 2 | 2 | 37 | 30 | 4 | 11.56523354300040 | 4.0508790870288300 | 0.9990350056120640 | 4.054791938493720 | 5.01789462120719E-05 | 0.003293790066310890 | 2.4823040845676600 | up |
| **C0_3D2.tig09257.1** | 33 | 39 | 14 | 7 | 10 | 5 | 18.061616492723700 | -2.046520824238580 | 0.5995144885415860 | -3.4136303014411900 | 0.0006410349679268760 | 0.03004091735515520 | 1.5222868094775000 | down |
| **LP_3D1.tig13855.1** | 3 | 5 | 11 | 32 | 20 | 41 | 18.356823024286800 | 2.2707544795890000 | 0.6107175727362340 | 3.7181744573276500 | 0.00020066767503810600 | 0.010999955629258600 | 1.958609066660600 | up |
| **LP_3D1.tig01752.2** | 31 | 26 | 19 | 164 | 145 | 147 | 86.3375011845439 | 2.517807138253790 | 0.34989267039519600 | 7.195941359417390 | 6.20312863881622E-13 | 1.49384729528579E-10 | 9.825693794892950 | up |
| **C0_90D3.tig25561.1** | 231 | 273 | 319 | 55 | 87 | 69 | 171.4452660543900 | -2.0087120971809000 | 0.30257424396414800 | -6.638741192455590 | 3.16373327790849E-11 | 6.18015182758447E-09 | 8.209000855482030 | down |
| **LP_3D1.tig00948.3** | 0 | 2 | 1 | 42 | 34 | 18 | 15.284511103624600 | 4.892898156980630 | 0.9841315332475730 | 4.971792887109680 | 6.63365522171083E-07 | 6.68599523188213E-05 | 4.174833937460470 | up |
| **LP_3D1.tig00078.7** | 0 | 0 | 2 | 42 | 23 | 7 | 11.424781390751500 | 5.0697102559458000 | 1.2185224239869900 | 4.1605391547557900 | 3.1749717638149E-05 | 0.002192968042698150 | 2.658967697087170 | up |
| **C0_3D1.tig23232.1** | 10 | 33 | 37 | 215 | 78 | 63 | 68.58231655513160 | 2.0786122261419600 | 0.48123577063165500 | 4.319321947771330 | 1.5650932178887E-05 | 0.0011799432887385000 | 2.92813886555811 | up |
| **LP_3D1.tig04601.1** | 9 | 17 | 37 | 718 | 481 | 270 | 240.85157113103300 | 4.4722708075942200 | 0.416287321779154 | 10.743230873523500 | 6.37727802587234E-27 | 7.71311424214283E-24 | 23.11277023607370 | up |
| **LP_3D1.tig00205.2** | 3 | 7 | 7 | 203 | 132 | 40 | 60.84342324872290 | 4.363144110576690 | 0.5711177581186340 | 7.63965758121351 | 2.17800193763006E-14 | 6.48212147353825E-12 | 11.188282834644000 | up |
| **C0_3D1.tig03010.4** | 19 | 64 | 66 | 17 | 10 | 5 | 29.39388597602230 | -2.277236714532690 | 0.5472079807375250 | -4.161556107905140 | 3.16086312673456E-05 | 0.002185879129430970 | 2.6603738565091300 | down |
| **LP_3D1.tig01944.1** | 0 | 5 | 4 | 77 | 39 | 22 | 22.957643635122700 | 3.8578160563413300 | 0.704954176125017 | 5.472435212096940 | 4.43893064025546E-08 | 5.36874494138982E-06 | 5.270127228031210 | up |
| **LP_3D1.tig06200.3** | 4 | 3 | 0 | 25 | 33 | 24 | 14.402189551820000 | 3.4775529666974700 | 0.7583709535732200 | 4.585556646535930 | 4.52778490519587E-06 | 0.0003905643898874950 | 3.4083073564920200 | up |
| **LP_3D1.tig00234.2** | 2 | 5 | 6 | 99 | 55 | 23 | 29.59835602176340 | 3.6736339135059700 | 0.6279441118906560 | 5.850256167615220 | 4.90816497513155E-09 | 7.11746525539165E-07 | 6.147674644227160 | up |
| **LP_3D1.tig00088.8** | 3 | 10 | 9 | 122 | 118 | 70 | 52.78666583100860 | 3.7520677202801900 | 0.47316583337911100 | 7.929709745703370 | 2.19658672025415E-15 | 7.17614782257741E-13 | 12.14410862377270 | up |
| **C0_3D1.tig02434.1** | 342 | 292 | 302 | 1219 | 1614 | 1195 | 806.0963152569480 | 2.0396736376576200 | 0.2610766958945000 | 7.812545775751060 | 5.60442791922458E-15 | 1.75045991795781E-12 | 11.756847829262900 | up |
| **LP_3D3.tig11795.1** | 0 | 1 | 1 | 32 | 25 | 14 | 11.507784960399000 | 5.073308165546340 | 1.170865996311100 | 4.332953712491590 | 1.47122050739687E-05 | 0.001116575831014350 | 2.9521117769608700 | up |
| **LP_3D1.tig01430.2** | 1 | 6 | 5 | 85 | 68 | 34 | 31.345569140794100 | 3.8869088493022600 | 0.6011405123061640 | 6.465890702309950 | 1.00703849155561E-10 | 1.81730485880885E-08 | 7.740572212266710 | up |
| **LP_3D1.tig02458.3** | 2 | 2 | 4 | 127 | 74 | 38 | 38.620434598570200 | 4.806545405334350 | 0.6697793023536120 | 7.176312239634900 | 7.1616891392504E-13 | 1.70337330176021E-10 | 9.768690164058380 | up |
| **LP_3D1.tig02365.2** | 1 | 0 | 5 | 139 | 84 | 55 | 44.67240712773470 | 5.451918716663030 | 0.7208488645722560 | 7.563192486816420 | 3.9329492830648E-14 | 1.11784250997909E-11 | 10.951619378797500 | up |
| **LP_3D1.tig04474.3** | 3 | 1 | 6 | 67 | 50 | 34 | 25.55840215528070 | 3.840172031996260 | 0.6332586817956970 | 6.064144310042300 | 1.32658006166173E-09 | 2.05475323174826E-07 | 6.687240327814750 | up |
| **LP_3D1.tig00158.2** | 11 | 15 | 15 | 267 | 191 | 70 | 89.01250829063100 | 3.5882499023811000 | 0.45147126021549200 | 7.94790326336253 | 1.89694685170317E-15 | 6.3059616248577E-13 | 12.2002486764938 | up |
| **LP_3D2.tig22393.1** | 0 | 2 | 2 | 227 | 85 | 63 | 59.01429883481370 | 6.456251616879240 | 0.8469733548443420 | 7.622732852163670 | 2.48360545137571E-14 | 7.31505450173329E-12 | 11.135782433761000 | up |
| **LP_3D2.tig00066.6** | 40 | 98 | 110 | 572 | 277 | 123 | 191.28965754930500 | 1.883171679460850 | 0.43744432689445700 | 4.304940225948360 | 1.67030829031349E-05 | 0.001246045600562600 | 2.904466063848340 | up |
| **C0_3D1.tig06583.3** | 44 | 161 | 145 | 56 | 24 | 35 | 75.69566449906100 | -1.6436311820838400 | 0.4450615081306270 | -3.6930427638811400 | 0.00022158674318942700 | 0.012030657513469900 | 1.9197106364450000 | down |
| **LP_3D1.tig11820.1** | 2 | 1 | 3 | 187 | 117 | 72 | 60.05211618947750 | 5.879904240224500 | 0.7002231783034290 | 8.397185957869770 | 4.57305530990679E-17 | 1.85682377922608E-14 | 13.731229310768100 | up |
| **LP_90D3.tig04623.1** | 1250 | 1526 | 1590 | 318 | 525 | 460 | 940.9072766739720 | -1.782559735375550 | 0.2798423342749160 | -6.369871592138640 | 1.89186524513268E-10 | 3.25888120786568E-08 | 7.486931470068370 | down |
| **FM3.tig11724.2** | 0 | 1 | 2 | 41 | 23 | 8 | 11.617721959225700 | 4.4901513669842500 | 1.0493727051470600 | 4.278890945953270 | 1.87826855306321E-05 | 0.0013814180864018600 | 2.859674862233810 | up |
| **LP_3D1.tig27045.1** | 6 | 8 | 5 | 58 | 17 | 20 | 18.06128226397360 | 2.229086759432010 | 0.6265872757736710 | 3.5575040311498100 | 0.00037439535763324300 | 0.018867468177891600 | 1.7242863738224700 | up |
| **C0_3D2.tig00886.1** | 169 | 146 | 165 | 427 | 875 | 556 | 381.00076225860700 | 1.8949637827923400 | 0.3157507479386990 | 6.001454613055210 | 1.95557609105291E-09 | 2.98028747710195E-07 | 6.525741842065560 | up |
| **LP_3D3.tig15688.1** | 1 | 0 | 1 | 26 | 20 | 10 | 9.133961999527620 | 4.716904516381260 | 1.198119380822110 | 3.9369236420703600 | 8.25328542449989E-05 | 0.005120450111530820 | 2.2906918608284700 | up |
| **LP_3D3.tig14080.1** | 0 | 1 | 1 | 47 | 43 | 17 | 17.096642946760800 | 5.657276538583270 | 1.145413798803120 | 4.939067911085720 | 7.849687374848E-07 | 7.77378882967308E-05 | 4.109367260894530 | up |
| **LP_3D1.tig03145.1** | 10 | 21 | 21 | 1043 | 378 | 213 | 261.1049729921970 | 4.872873298066190 | 0.469051555652715 | 10.388779739330100 | 2.78898367316127E-25 | 2.7813995947518E-22 | 21.555736612959300 | up |
| **LP_3D1.tig04891.1** | 0 | 0 | 1 | 49 | 32 | 13 | 14.791265702313500 | 6.349568203783250 | 1.5015254007267300 | 4.228745115274160 | 2.34998394621432E-05 | 0.001682428682903690 | 2.7740633361844200 | up |
| **LP_3D3.tig25683.1** | 3 | 1 | 1 | 24 | 9 | 15 | 8.53398840657504 | 3.181061633828060 | 0.9185227853304390 | 3.46323649737624 | 0.0005337190649422450 | 0.025667732865179300 | 1.5906124892414600 | up |
| **LP_3D1.tig04292.3** | 1 | 1 | 0 | 236 | 102 | 36 | 57.87659649709540 | 7.424052377428980 | 1.13315092518205 | 6.551688934319360 | 5.68899510928972E-11 | 1.07438846175274E-08 | 7.968838664360210 | up |
| **LP_3D1.tig00427.3** | 2 | 3 | 1 | 26 | 21 | 6 | 9.242441937287280 | 3.0407695061755300 | 0.8814157211831900 | 3.449869832244060 | 0.0005608569158265980 | 0.026769027187374400 | 1.5723674112124700 | up |
| **LP_3D1.tig02271.1** | 0 | 2 | 3 | 62 | 34 | 24 | 19.654340777432700 | 4.508988608685130 | 0.8154543219301750 | 5.529418984514520 | 3.2129313762929E-08 | 4.00524307204759E-06 | 5.397371122115170 | up |
| **LP_3D1.tig02621.1** | 0 | 4 | 2 | 96 | 67 | 32 | 31.480220214870900 | 4.9392052852385400 | 0.7435238572136210 | 6.642968127140360 | 3.07428339328145E-11 | 6.02612549969255E-09 | 8.219961827980220 | up |
| **LP_3D1.tig03195.3** | 4 | 8 | 7 | 1413 | 1115 | 413 | 462.01274223961600 | 7.178968931158570 | 0.48921078905643300 | 14.674592408325700 | 9.37773641065092E-49 | 7.61539180376359E-45 | 44.118307747766500 | up |
| **LP_90D2.tig18018.1** | 3 | 6 | 14 | 1595 | 964 | 453 | 473.2038706249000 | 6.9452042777340800 | 0.4851422403817690 | 14.315810291572900 | 1.7432042615662E-46 | 9.00840420443008E-43 | 42.04535213539070 | up |
| **C0_3D1.tig03405.1** | 96 | 186 | 131 | 830 | 250 | 298 | 284.59932924666300 | 1.6551989936979100 | 0.3938606272280110 | 4.20249925804259 | 2.63984114047E-05 | 0.0018664399207713600 | 2.7289859851094500 | up |
| **LP_3D1.tig00603.3** | 0 | 2 | 0 | 34 | 21 | 11 | 10.64330695319740 | 4.9577373839796800 | 1.1952495982970400 | 4.147867852073170 | 3.35585998968292E-05 | 0.0023122892256184900 | 2.635957844458220 | up |
| **LP_3D3.tig06987.1** | 2 | 3 | 3 | 197 | 85 | 45 | 52.01173172921700 | 5.248005737733120 | 0.6804854231465570 | 7.712150119934080 | 1.23715483474684E-14 | 3.82206883593393E-12 | 11.41770149551310 | up |
| **LP_3D1.tig06772.1** | 1 | 3 | 5 | 66 | 33 | 10 | 18.247394307028400 | 3.4988023602341500 | 0.7585314608373430 | 4.6126001898086300 | 3.97662756745991E-06 | 0.0003456443334438210 | 3.4613705586260500 | up |
| **LP_3D1.tig00318.2** | 4 | 5 | 4 | 121 | 90 | 52 | 43.593850071242000 | 4.2531530142763800 | 0.5522222711919460 | 7.70188606319 | 1.34072408817909E-14 | 4.11964652932649E-12 | 11.385140045362100 | up |
| **LP_3D1.tig03642.1** | 2 | 5 | 5 | 102 | 15 | 26 | 24.154736315617900 | 3.4806103657022500 | 0.7180724995297460 | 4.847157310691670 | 1.25243115217402E-06 | 0.00011965453587450800 | 3.92207083346566 | up |
| **LP_3D1.tig25269.1** | 42 | 50 | 27 | 15 | 7 | 4 | 24.100796373083800 | -2.2875558763837500 | 0.5447480000743660 | -4.199291922267680 | 2.67750948160715E-05 | 0.0018883750183865800 | 2.7239117535535500 | down |
| **LP_3D1.tig03276.1** | 0 | 1 | 2 | 31 | 26 | 14 | 11.675765372914800 | 4.492471125566320 | 1.0111887557086200 | 4.442762145251600 | 8.88113272389946E-06 | 0.000706081104461629 | 3.151145410567040 | up |
| **C0_3D1.tig28777.2** | 54 | 80 | 101 | 205 | 1337 | 415 | 353.29539283690400 | 3.0123663065626700 | 0.7793035092851270 | 3.8654596966026600 | 0.00011088015874524900 | 0.006634718551445980 | 2.1781774954290100 | up |
| **LP_3D1.tig11901.2** | 4 | 5 | 10 | 58 | 34 | 71 | 29.81481058145970 | 3.0734599852984900 | 0.5530626941798440 | 5.557163803746020 | 2.74193421938649E-08 | 3.45599225501165E-06 | 5.461427239459770 | up |
| **C0_3D3.tig05573.1** | 127 | 153 | 82 | 73 | 29 | 33 | 82.31225642650600 | -1.5116802758333700 | 0.3969504266532250 | -3.8082344150091400 | 0.000139962561538256 | 0.008126835353056350 | 2.0900785388177900 | down |
| **LP_3D1.tig06084.1** | 0 | 0 | 1 | 89 | 43 | 22 | 24.07674674448300 | 7.057372885694200 | 1.4815963485738700 | 4.763357369561140 | 1.90398205039931E-06 | 0.0001740753654330160 | 3.7592626844455000 | up |
| **LP_3D2.tig09621.1** | 1 | 0 | 0 | 67 | 47 | 28 | 22.527990598732400 | 6.9582667160935500 | 1.4660187730575900 | 4.7463694489949200 | 2.07100288112604E-06 | 0.00018716400441591400 | 3.727777671552470 | up |
| **LP_3D1.tig00547.4** | 105 | 113 | 121 | 3220 | 2120 | 1201 | 1083.0544250371100 | 4.176894650717310 | 0.31300427800590000 | 13.344528954452700 | 1.27473784358006E-40 | 4.83083151455391E-37 | 36.31597810918610 | up |
| **LP_3D1.tig15072.1** | 0 | 1 | 0 | 13 | 23 | 6 | 6.77491553904309 | 5.202740553441120 | 1.57818326628312 | 3.29666437643483 | 0.0009784034267624740 | 0.04298094497242100 | 1.3667240404081700 | up |
| **LP_3D1.tig08235.1** | 21 | 23 | 19 | 189 | 57 | 39 | 54.61286580367110 | 2.06180531080087 | 0.49826206172451900 | 4.137993777139730 | 3.50355871164415E-05 | 0.0023995156019688100 | 2.6198764218820400 | up |
| **LP_3D1.tig00715.3** | 14 | 10 | 8 | 36 | 31 | 48 | 24.25577361231970 | 1.7945334640297400 | 0.5093765720282550 | 3.5229996088830700 | 0.0004266919900166080 | 0.02102878637682630 | 1.677185790797100 | up |
| **LP_3D1.tig00318.3** | 0 | 8 | 6 | 91 | 49 | 31 | 29.02696638250390 | 3.5389893145115100 | 0.6218932659268790 | 5.690669940342490 | 1.26541900739737E-08 | 1.71268436846436E-06 | 5.766322666027170 | up |
| **LP_3D3.tig17511.1** | 0 | 2 | 0 | 49 | 35 | 25 | 17.576723496444200 | 5.694313502576780 | 1.1336401742053900 | 5.023034320892970 | 5.08614513572454E-07 | 5.25676218618657E-05 | 4.279281669845390 | up |
| **LP_3D1.tig00158.3** | 6 | 10 | 20 | 491 | 359 | 192 | 169.5953998006360 | 4.778292595291140 | 0.426003202415662 | 11.216564965229600 | 3.38151387972431E-29 | 5.33950434702579E-26 | 25.272499055582400 | up |
| **LP_3D1.tig00993.1** | 4 | 0 | 1 | 29 | 23 | 14 | 11.310802170818200 | 3.620330245481220 | 0.8748080293358060 | 4.1384282311971200 | 3.49693222990874E-05 | 0.0023978662558403200 | 2.620175043910390 | up |
| **C0_3D1.tig11902.1** | 15 | 14 | 13 | 43 | 68 | 66 | 35.95116950269240 | 2.0298221558935900 | 0.44703925373837000 | 4.540590426722450 | 5.60969125484063E-06 | 0.0004724191101946900 | 3.325672542717630 | up |
| **LP_3D3.tig04248.1** | 5 | 2 | 8 | 27 | 19 | 33 | 15.417905460719100 | 2.357250405769970 | 0.6447193184564470 | 3.656242861488280 | 0.0002559388918404360 | 0.013673727731832300 | 1.8641130719231900 | up |
| **LP_3D2.tig02558.3** | 3 | 4 | 1 | 30 | 31 | 12 | 12.82316798014550 | 3.098476603546910 | 0.7584563052248120 | 4.085240747821980 | 4.40311389033509E-05 | 0.002930854907448460 | 2.5330056808635800 | up |
| **LP_3D1.tig00918.3** | 0 | 4 | 1 | 117 | 58 | 46 | 35.542216785157500 | 5.385899789096640 | 0.7808103871824760 | 6.897833171163950 | 5.28017013115477E-12 | 1.15442796579036E-09 | 8.937633161088750 | up |
| **LP_3D1.tig04477.1** | 15 | 48 | 40 | 1146 | 633 | 318 | 343.5285296943220 | 4.265520636469050 | 0.4175513548475640 | 10.215559324496200 | 1.68902206229456E-24 | 1.50019467392397E-21 | 20.823852380727600 | up |
| **LP_3D1.tig02250.2** | 0 | 4 | 2 | 21 | 17 | 10 | 8.548871873676880 | 2.9373891682240800 | 0.8819402977812760 | 3.3305986534618700 | 0.0008665944978607200 | 0.038788633252671300 | 1.4112955228304800 | up |
| **FM2.tig00193.1** | 37 | 86 | 163 | 32 | 24 | 43 | 62.98713580540540 | -1.5352078637723300 | 0.4664490064937360 | -3.2912662314630600 | 0.0009973747312057680 | 0.043679327115093900 | 1.3597240606067200 | down |
| **C0_3D2.tig01615.2** | 566 | 978 | 774 | 4354 | 1477 | 1859 | 1597.4611566802400 | 1.6533321284811500 | 0.35133635974306900 | 4.705838387151920 | 2.52824753586275E-06 | 0.00022491115990002900 | 3.6479889947077400 | up |
| **C0_3D1.tig25149.1** | 23 | 29 | 15 | 109 | 203 | 188 | 92.99008039561240 | 2.8578728215125200 | 0.399483953860114 | 7.153911424720840 | 8.43394981454623E-13 | 1.989327291319E-10 | 9.701293759298680 | up |
| **LP_3D1.tig08794.3** | 1 | 1 | 7 | 22 | 13 | 24 | 11.073775349964000 | 2.687025760073470 | 0.7884904099440940 | 3.4078103248763600 | 0.0006548639790501360 | 0.030512904007463100 | 1.515516457241280 | up |
| **LP_3D2.tig10834.1** | 0 | 1 | 1 | 33 | 20 | 6 | 9.439413966902720 | 4.78433759874777 | 1.2268557037627200 | 3.899674251889930 | 9.63221935672752E-05 | 0.005856080313723800 | 2.2323929757393000 | up |
| **LP_3D2.tig01827.1** | 3 | 0 | 4 | 27 | 28 | 5 | 10.482702872650000 | 2.990073828674700 | 0.8760001501903270 | 3.4133257032262500 | 0.0006417519433132560 | 0.0300497440013526 | 1.5221592234706800 | up |
| **LP_3D1.tig03264.1** | 7 | 7 | 6 | 113 | 32 | 30 | 30.562431755379100 | 3.0225918416482300 | 0.5890745600197580 | 5.131085344352420 | 2.88076248307188E-07 | 3.12513250668361E-05 | 4.505131563695430 | up |
| **LP_3D1.tig06011.1** | 0 | 1 | 9 | 192 | 58 | 32 | 44.98058596821340 | 4.7460507612913300 | 1.4023227886991100 | 3.3844210473781900 | 0.000713285295202782 | 0.03283133814234990 | 1.4837114159001100 | up |
| **LP_3D1.tig01689.2** | 3 | 10 | 6 | 57 | 16 | 25 | 18.593084738375600 | 2.297678655246530 | 0.632472075691567 | 3.632853913327580 | 0.00028030372928254000 | 0.014780951290413700 | 1.8302976141900200 | up |
| **LP_3D1.tig02625.2** | 3 | 4 | 1 | 53 | 41 | 22 | 19.6028208096215 | 3.7684201661028700 | 0.7003529387885500 | 5.3807444181234900 | 7.41784478361875E-08 | 8.76647373648249E-06 | 5.057175063960290 | up |
| **LP_3D1.tig11032.1** | 0 | 1 | 0 | 36 | 20 | 8 | 10.083989860661400 | 5.790580254965650 | 1.5373415059280300 | 3.7666193442621800 | 0.0001654730015743390 | 0.009359515198500810 | 2.0287466461410300 | up |
| **LP_3D1.tig03934.3** | 30 | 40 | 28 | 118 | 141 | 104 | 74.69670526254970 | 1.8281976410659700 | 0.34189352919930900 | 5.347271840293340 | 8.92898603508384E-08 | 1.03206726752022E-05 | 4.986291995576270 | up |
| **LP_3D1.tig09680.1** | 1 | 2 | 1 | 45 | 22 | 6 | 11.881217832748500 | 4.076547359051930 | 0.9735711548299370 | 4.187210496970840 | 2.82403891001115E-05 | 0.0019794388636200200 | 2.7034579071992200 | up |
| **LP_3D1.tig04474.2** | 0 | 1 | 0 | 21 | 15 | 16 | 8.503337386801850 | 5.53276812742121 | 1.5278179847639700 | 3.6213529246260000 | 0.0002930663969409420 | 0.015368412669841200 | 1.8133709863928100 | up |
| **C0_3D3.tig15736.2** | 0 | 0 | 4 | 17 | 23 | 6 | 7.847451320531470 | 3.454489884678550 | 1.0411686713750400 | 3.3178964942503400 | 0.0009069808747227020 | 0.040310655061463600 | 1.394580144360780 | up |
| **LP_3D1.tig00158.1** | 12 | 12 | 14 | 337 | 220 | 109 | 110.49024924341200 | 4.034427293918210 | 0.42776649420205500 | 9.43137751226619 | 4.04738141770785E-21 | 2.61447041692731E-18 | 17.582616267905100 | up |
| **LP_3D1.tig04350.2** | 0 | 3 | 0 | 50 | 10 | 13 | 11.82027235702590 | 4.511603019181180 | 1.0828355671947600 | 4.1664710283475000 | 3.09351186566784E-05 | 0.0021471389744064500 | 2.6681398447745000 | up |
| **LP_3D1.tig11306.1** | 0 | 2 | 1 | 46 | 27 | 46 | 19.800473718003600 | 5.269271558314240 | 0.9628396962624300 | 5.472636388766060 | 4.43389271878439E-08 | 5.36874494138982E-06 | 5.270127228031210 | up |
| **C0_3D1.tig06650.2** | 119 | 105 | 138 | 15 | 38 | 23 | 73.01737342180620 | -2.3059209795632300 | 0.386920859026569 | -5.959670888161870 | 2.52746434792086E-09 | 3.78088712783056E-07 | 6.42240628759172 | down |
| **C0_3D3.tig13664.1** | 44 | 99 | 145 | 531 | 313 | 151 | 202.33863312601800 | 1.7129118849888900 | 0.41514657079154600 | 4.126041271936660 | 3.69061064084907E-05 | 0.002500509676746900 | 2.6019714604325900 | up |
| **LP_3D1.tig02572.2** | 14 | 11 | 21 | 149 | 22 | 28 | 38.31027542719380 | 1.9981456089340600 | 0.5975918701965230 | 3.3436626376409000 | 0.0008268021480369210 | 0.03747971938210430 | 1.4262036694187300 | up |
| **LP_3D1.tig00471.1** | 4 | 11 | 12 | 267 | 238 | 131 | 104.90976505166100 | 4.48708720059621 | 0.4337788120208320 | 10.344182510188500 | 4.44695271875239E-25 | 4.35839702237034E-22 | 21.360673210810500 | up |
| **C0_3D3.tig32233.1** | 11 | 16 | 28 | 390 | 331 | 191 | 153.1688274049210 | 3.9810861793997700 | 0.3783803065028430 | 10.521388431112400 | 6.88514114159516E-26 | 7.38463864516937E-23 | 22.1316707512997 | up |
| **LP_3D1.tig10343.1** | 1 | 0 | 0 | 32 | 11 | 9 | 8.276924635527640 | 5.496502903832990 | 1.5579458941617000 | 3.528044795670220 | 0.00041864130384955000 | 0.020729673272933500 | 1.6834075429076 | up |
| **LP_3D1.tig08647.2** | 5 | 12 | 21 | 40 | 57 | 53 | 30.594431786109400 | 1.9558889955978100 | 0.4956377315846110 | 3.9462068179204300 | 7.93990377087845E-05 | 0.004954377934748470 | 2.305010867206120 | up |
| **LP_3D2.tig21479.1** | 0 | 0 | 2 | 18 | 25 | 9 | 8.527131366218760 | 4.630130700095070 | 1.2221505343166000 | 3.7885109649640200 | 0.00015155290509693800 | 0.008675755176470740 | 2.061692711960750 | up |
| **LP_3D1.tig12892.1** | 62 | 62 | 62 | 417 | 77 | 102 | 123.59471844858900 | 1.5664800682420300 | 0.47253330892070500 | 3.3150680357750200 | 0.0009162082238664250 | 0.040625473077758900 | 1.391201568687190 | up |
| **LP_3D1.tig03917.2** | 3 | 2 | 2 | 117 | 79 | 39 | 37.95701579741540 | 4.971010767017380 | 0.6885115464566990 | 7.219938129723150 | 5.20112324131731E-13 | 1.26891781396001E-10 | 9.896566505643300 | up |
| **LP_3D1.tig07415.1** | 3 | 13 | 16 | 377 | 252 | 110 | 120.48364051972700 | 4.449522776014620 | 0.4775561176318960 | 9.317277303615960 | 1.19363941777591E-20 | 7.29596050574964E-18 | 17.1369175255817 | up |
| **LP_3D1.tig04122.2** | 0 | 1 | 0 | 27 | 17 | 8 | 8.27539516891432 | 5.499658595820190 | 1.547097010785990 | 3.5548246538374100 | 0.0003782312397127290 | 0.01901021646460660 | 1.7210129379031600 | up |
| **LP_3D1.tig00425.2** | 7 | 8 | 15 | 333 | 229 | 101 | 108.47574440800500 | 4.374795437887950 | 0.4616123953432730 | 9.47720529608978 | 2.61184384487721E-21 | 1.70655475128787E-18 | 17.7678797737195 | up |
| **LP_3D1.tig00088.7** | 5 | 4 | 4 | 79 | 80 | 27 | 31.319055116124300 | 3.7418365192356800 | 0.5960928936493120 | 6.2772708064475700 | 3.44567642740627E-10 | 5.7949549265062E-08 | 7.236949937654790 | up |
| **LP_3D1.tig17030.1** | 0 | 2 | 2 | 78 | 47 | 13 | 21.939551869458700 | 5.008110905192740 | 0.9121244846431570 | 5.490600230024550 | 4.00570092825199E-08 | 4.87589013418596E-06 | 5.311946088667750 | up |
| **LP_3D2.tig04427.1** | 1 | 2 | 4 | 106 | 84 | 42 | 37.59960693371170 | 4.969111149738110 | 0.6833667139474960 | 7.27151476406252 | 3.55478514841713E-13 | 8.90183972518818E-11 | 10.05052022931800 | up |
| **C0_3D2.tig00354.2** | 35 | 89 | 44 | 26 | 15 | 16 | 36.92822386049220 | -1.614705310469660 | 0.46194192859598800 | -3.4954725053370800 | 0.0004732232047723410 | 0.023110286147151000 | 1.636194677136240 | down |
| **LP_3D1.tig16269.1** | 1 | 3 | 5 | 582 | 451 | 203 | 195.07203223159700 | 7.014073801676290 | 0.5968181682940530 | 11.752446849474000 | 6.86031390782293E-32 | 1.34473980720757E-28 | 27.871361738877500 | up |
| **LP_3D1.tig00597.2** | 8 | 18 | 16 | 204 | 187 | 91 | 82.88300877329590 | 3.445739427142480 | 0.4140875663263990 | 8.321282036337260 | 8.70169163981245E-17 | 3.34221392746715E-14 | 13.475965755356700 | up |
| **C0_3D3.tig05165.1** | 814 | 1129 | 830 | 5012 | 1831 | 2343 | 1915.9709250779300 | 1.6468058097651100 | 0.3332261690006160 | 4.94200624970142 | 7.73227365989011E-07 | 7.68390933387452E-05 | 4.1144177682286500 | up |
| **LP_3D1.tig05030.1** | 0 | 1 | 1 | 74 | 31 | 11 | 18.156785596950300 | 5.748846797597160 | 1.1828674402304000 | 4.860093871953560 | 1.17330105364664E-06 | 0.00011323649982095700 | 3.946013563290840 | up |
| **C0_3D1.tig28777.1** | 57 | 91 | 105 | 294 | 1403 | 492 | 393.6432667957670 | 3.0675822754836200 | 0.44326147794780500 | 6.920480186290470 | 4.5011527442145E-12 | 9.95595438696004E-10 | 9.001917101771860 | up |
| **LP_3D1.tig00906.3** | 80 | 106 | 135 | 1012 | 761 | 340 | 384.0037743677900 | 2.6320675362754800 | 0.3431223576734470 | 7.670929851736580 | 1.70753917842101E-14 | 5.21855186007218E-12 | 11.28244999631450 | up |
| **LP_3D1.tig03239.3** | 0 | 4 | 3 | 50 | 20 | 8 | 13.133602335296100 | 3.384473149574450 | 0.8501089083974050 | 3.9812230128898900 | 6.85615843415128E-05 | 0.004344908876135220 | 2.3620193273871500 | up |
| **LP_3D1.tig00689.3** | 2 | 2 | 4 | 290 | 296 | 142 | 116.28470113120200 | 6.4270457900097900 | 0.6090011196664170 | 10.553421960094700 | 4.89800419223835E-26 | 5.45935388838802E-23 | 22.2628587527736 | up |
| **C0_3D1.tig09957.4** | 132 | 149 | 136 | 78 | 49 | 27 | 94.01196891765930 | -1.5273383170758500 | 0.35353386671857100 | -4.320203694351250 | 1.55885241600768E-05 | 0.0011783639040951700 | 2.928720569366700 | down |
| **LP_3D1.tig03277.2** | 1 | 4 | 6 | 189 | 139 | 85 | 66.91254274026140 | 5.155781499518780 | 0.5655519956981440 | 9.116370446459560 | 7.76823770521413E-20 | 4.20557592717045E-17 | 16.376174521895800 | up |
| **LP_3D2.tig01408.1** | 5 | 7 | 7 | 75 | 86 | 35 | 34.06537160008610 | 3.286872345899140 | 0.5258406538155600 | 6.250700325372760 | 4.0861624956966E-10 | 6.79175166865127E-08 | 7.1680182018727700 | up |
| **LP_3D1.tig15129.1** | 9 | 6 | 2 | 35 | 29 | 31 | 18.230878038939700 | 2.407499711625560 | 0.5979980587732680 | 4.025932319185620 | 5.67500114349634E-05 | 0.003682596347055360 | 2.4338458819742300 | up |
| **LP_3D1.tig00429.3** | 3 | 1 | 2 | 57 | 58 | 31 | 24.146902564015900 | 4.521603136880340 | 0.7327788623280150 | 6.170487945729420 | 6.8079565831647E-10 | 1.0901360337183E-07 | 6.962519304805280 | up |
| **LP_3D2.tig01066.5** | 4 | 0 | 3 | 553 | 454 | 212 | 192.5782481342680 | 7.346043076324630 | 0.6448345716114820 | 11.392135905440700 | 4.5763222984928E-30 | 7.88306185023706E-27 | 26.10330506596390 | up |
| **LP_3D1.tig22879.1** | 0 | 1 | 4 | 30 | 16 | 10 | 9.563395405388800 | 3.412590430846440 | 0.9221597904682220 | 3.700649785558000 | 0.00021504809789149000 | 0.011720430608477200 | 1.931056432050860 | up |
| **LP_3D1.tig06390.2** | 1 | 6 | 2 | 90 | 50 | 53 | 32.19377539525960 | 4.358465673785030 | 0.6381762530094230 | 6.829564173270290 | 8.51729898599629E-12 | 1.81335528411595E-09 | 8.74151709782696 | up |
| **LP_3D1.tig00694.2** | 15 | 37 | 24 | 708 | 434 | 306 | 240.7080455446880 | 4.176432168312060 | 0.3691090998378480 | 11.314898955747200 | 1.10729809881027E-29 | 1.79841029791057E-26 | 25.745111219282200 | up |
| **LP_3D2.tig23224.1** | 0 | 3 | 5 | 48 | 25 | 12 | 14.493996350632600 | 3.329297142315580 | 0.7834047588264080 | 4.249779063511300 | 2.13981470282706E-05 | 0.001549525691493050 | 2.809801218662410 | up |
| **LP_3D1.tig07276.1** | 1 | 1 | 2 | 51 | 59 | 20 | 21.0774164057458 | 4.93669453402671 | 0.8719710002327300 | 5.661535226181950 | 1.50024701049807E-08 | 1.9879147158919E-06 | 5.701602251333090 | up |
| **LP_3D1.tig12212.1** | 0 | 1 | 0 | 50 | 37 | 23 | 17.51794415649440 | 6.593518400831810 | 1.4758223536029100 | 4.4676911043765800 | 7.9068366619917E-06 | 0.0006402622935198260 | 3.193642073997270 | up |
| **C0_3D1.tig15970.1** | 20 | 18 | 24 | 7 | 4 | 4 | 12.774561838496500 | -2.1153766796581200 | 0.6429719402708350 | -3.289998438760910 | 0.001001879386449440 | 0.04384282811602630 | 1.358101439113800 | down |
| **LP_3D1.tig17525.1** | 0 | 2 | 2 | 32 | 33 | 9 | 12.187700925495800 | 4.126579862729390 | 0.9432352830750440 | 4.3749210157579300 | 1.21476454826265E-05 | 0.0009407805278745260 | 3.0265116801329000 | up |
| **FM3.tig02399.1** | 3 | 7 | 9 | 127 | 95 | 34 | 42.98764261680200 | 3.6637463888898900 | 0.5508834511459390 | 6.650674260169970 | 2.91753341674709E-11 | 5.73865699221413E-09 | 8.241189732883380 | up |
| **LP_3D1.tig06851.1** | 0 | 0 | 2 | 64 | 32 | 20 | 18.436649515764900 | 5.7698827454694000 | 1.150449477963530 | 5.0153291004859800 | 5.29427655317808E-07 | 5.46194465817437E-05 | 4.262652704557190 | up |
| **LP_3D1.tig00254.7** | 3 | 4 | 8 | 81 | 60 | 22 | 27.86433587014980 | 3.352141798895300 | 0.6031621218937770 | 5.557613247281540 | 2.73488539406466E-08 | 3.45476800501346E-06 | 5.461581111104640 | up |
| **LP_3D2.tig08967.1** | 3 | 17 | 15 | 558 | 526 | 261 | 217.88524555272600 | 5.1974900714699 | 0.42289204713615700 | 12.290347162278200 | 1.02066757050105E-34 | 2.63726582023327E-31 | 30.57884609377800 | up |
| **FM3.tig24962.2** | 5 | 21 | 9 | 519 | 356 | 242 | 182.09568178490800 | 4.924257933505900 | 0.4219271408911230 | 11.670872660871500 | 1.79571709912316E-31 | 3.40258461665519E-28 | 27.468191065804400 | up |
| **FM2.tig02055.1** | 10 | 29 | 40 | 488 | 359 | 161 | 170.6059092440800 | 3.6008943357808800 | 0.42359057843803300 | 8.500883917340630 | 1.88152450924068E-17 | 7.98173587520792E-15 | 14.097902647616600 | up |
| **LP_3D1.tig01995.3** | 1 | 0 | 0 | 68 | 51 | 25 | 22.765830648304000 | 6.974019279934720 | 1.4690946270339 | 4.747154575069990 | 2.06298291666958E-06 | 0.00018703391371305000 | 3.7280796383670300 | up |
| **LP_3D1.tig02808.1** | 0 | 2 | 0 | 48 | 30 | 18 | 15.390108316354300 | 5.5011691597781700 | 1.1537510056198400 | 4.768073122348190 | 1.85996280287436E-06 | 0.0001710816260350250 | 3.7667966306698300 | up |
| **LP_3D2.tig08196.1** | 1 | 0 | 2 | 76 | 46 | 14 | 21.52096259176030 | 5.393685560916440 | 0.9970262486245950 | 5.4097728804603400 | 6.31047277144947E-08 | 7.50457792244864E-06 | 5.124673728618070 | up |
| **LP_3D1.tig00471.3** | 4 | 3 | 0 | 34 | 28 | 12 | 12.806795304698200 | 3.2954409705428400 | 0.7976597163992960 | 4.131386984689090 | 3.60580959068549E-05 | 0.002448891830137600 | 2.6110303976486000 | up |
| **LP_3D1.tig04541.1** | 5 | 1 | 1 | 102 | 63 | 29 | 31.467097669592200 | 4.675808121409900 | 0.7195559131580260 | 6.498185944839870 | 8.12942884700653E-11 | 1.49552551070578E-08 | 7.825206174365020 | up |
| **FM1.tig29958.2** | 1 | 1 | 0 | 41 | 13 | 19 | 11.883382388717300 | 5.105229405977050 | 1.182727987603150 | 4.316486512104140 | 1.58532363265173E-05 | 0.0011936122105706900 | 2.9231367470460500 | up |
| **LP_3D1.tig12471.1** | 1 | 0 | 8 | 310 | 287 | 140 | 117.62456387247900 | 6.297383650657840 | 1.066625125442730 | 5.904027104221770 | 3.54733843536669E-09 | 5.21055435034675E-07 | 6.283116069696750 | up |
| **LP_3D1.tig00142.3** | 2 | 3 | 4 | 68 | 49 | 20 | 22.865133805608200 | 3.836992446720660 | 0.6808514196183010 | 5.635579711167740 | 1.7447041836666E-08 | 2.28520067558819E-06 | 5.641075656218270 | up |
| **LP_3D1.tig04981.1** | 5 | 2 | 7 | 141 | 81 | 45 | 44.07770044408180 | 4.156034334343150 | 0.5724371101036530 | 7.2602461667633800 | 3.86386487508581E-13 | 9.63339468527426E-11 | 10.016220646074800 | up |
| **LP_3D2.tig33377.1** | 2 | 1 | 2 | 46 | 38 | 19 | 17.045443364005900 | 4.276619546210170 | 0.8145188948821740 | 5.250485376191070 | 1.51698948236345E-07 | 1.70758944801882E-05 | 4.767616537565300 | up |
| **LP_3D2.tig16972.1** | 110 | 91 | 100 | 307 | 287 | 389 | 211.06407385499300 | 1.6606678261899400 | 0.3013333203183500 | 5.511066032908330 | 3.56666874020858E-08 | 4.39798881859343E-06 | 5.356745879120150 | up |
| **LP_3D2.tig33127.1** | 0 | 0 | 1 | 24 | 21 | 5 | 7.915892572755740 | 5.435731718474680 | 1.5658379954360200 | 3.4714521772484200 | 0.0005176515254408360 | 0.025022024628983300 | 1.6016775527712500 | up |
| **LP_3D1.tig02076.3** | 0 | 2 | 2 | 144 | 51 | 37 | 36.68326357544060 | 5.762891214601440 | 0.8697330416903340 | 6.62604608352145 | 3.44797569113306E-11 | 6.71233486857736E-09 | 8.173126385321850 | up |
| **FM2.tig43595.1** | 9 | 24 | 21 | 169 | 118 | 60 | 63.26694076497340 | 2.6067943358530100 | 0.43606004904764600 | 5.978062749720460 | 2.25806760275654E-09 | 3.39576330366919E-07 | 6.469062589185710 | up |
| **LP_3D1.tig00149.2** | 32 | 55 | 52 | 1450 | 1009 | 479 | 482.64649450110600 | 4.3146921164338400 | 0.3568026641192020 | 12.092656670838000 | 1.15490763455363E-33 | 2.52502786485388E-30 | 29.597733824877800 | up |
| **C0_90D3.tig23157.1** | 460 | 632 | 710 | 153 | 267 | 216 | 402.9662588184800 | -1.5379674583183000 | 0.2982294463291210 | -5.156993976446670 | 2.50945872542579E-07 | 2.75730551294613E-05 | 4.559515110849000 | down |
| **LP_3D1.tig02091.2** | 0 | 0 | 1 | 43 | 38 | 7 | 13.763762818816700 | 6.245071502806260 | 1.5268306605086200 | 4.0902188201577600 | 4.30966397063594E-05 | 0.00287876437615511 | 2.5407938802151200 | up |
| **LP_3D1.tig08059.2** | 0 | 0 | 3 | 23 | 19 | 3 | 7.415421528616650 | 3.8149876450404500 | 1.1562793531437900 | 3.2993650147500600 | 0.0009690381899756540 | 0.04270153171253180 | 1.3695565464651500 | up |
| **C0_3D1.tig06545.2** | 1665 | 2514 | 2025 | 11926 | 4169 | 5141 | 4382.503045536930 | 1.6948209457379100 | 0.33952220132069800 | 4.9917823905042900 | 5.98246351528894E-07 | 6.10186430746714E-05 | 4.2145374543752700 | up |
| **LP_3D2.tig00283.2** | 2 | 13 | 8 | 132 | 109 | 33 | 46.37912892462250 | 3.4908890774553300 | 0.55814334046255 | 6.2544669520957300 | 3.98875912181267E-10 | 6.64929654778419E-08 | 7.177224297790920 | up |
| **LP_3D2.tig27770.1** | 1 | 2 | 5 | 23 | 21 | 11 | 9.988680222094920 | 2.715024087805820 | 0.7924090022063740 | 3.426291322090160 | 0.0006118837287266190 | 0.02884123595312160 | 1.5399861324366900 | up |
| **LP_3D1.tig10823.1** | 3 | 5 | 9 | 180 | 161 | 87 | 70.38453045501740 | 4.579824289569550 | 0.4960084302850030 | 9.233359777651380 | 2.62273367629445E-20 | 1.49089295828958E-17 | 16.826553536488600 | up |
| **C0_3D2.tig00650.3** | 51 | 124 | 132 | 811 | 207 | 209 | 241.01754321806400 | 1.9142927155610600 | 0.4517809046535370 | 4.23721475574338 | 2.26309653365623E-05 | 0.0016317867195390100 | 2.7873366057416900 | up |
| **LP_3D2.tig16555.1** | 0 | 0 | 1 | 14 | 20 | 6 | 6.451150518558560 | 5.131285338968530 | 1.5789372052467000 | 3.249834966151680 | 0.0011547199876356200 | 0.04957708285282990 | 1.3047190309806800 | up |
| **LP_3D1.tig03925.1** | 0 | 1 | 0 | 33 | 22 | 3 | 9.055135300780330 | 5.633760457798200 | 1.5803619482626900 | 3.5648545347421700 | 0.0003640580431997420 | 0.01846108783736780 | 1.7337427113357700 | up |
| **LP_3D1.tig00528.2** | 0 | 1 | 3 | 42 | 31 | 13 | 14.075996372878500 | 4.344917046380170 | 0.919089322296494 | 4.727415432837030 | 2.27395720140892E-06 | 0.00020453021695267400 | 3.6892425209751300 | up |
| **LP_3D1.tig04358.1** | 3 | 7 | 6 | 162 | 96 | 49 | 50.5578278728918 | 4.171943345184600 | 0.5474384020631520 | 7.620845248454700 | 2.52019938238283E-14 | 7.38457391193568E-12 | 11.131674558311900 | up |
| **LP_3D1.tig00618.6** | 3 | 3 | 2 | 23 | 20 | 10 | 9.697691283706830 | 2.6409079943908600 | 0.7844135231686620 | 3.3667293033435100 | 0.0007606531434242900 | 0.03484232710552280 | 1.4578928463355500 | up |
| **LP_3D1.tig00328.4** | 0 | 5 | 6 | 111 | 112 | 58 | 46.22392907002030 | 4.61000521187214 | 0.5860435394472520 | 7.866318629193030 | 3.65229598447037E-15 | 1.16394827994301E-12 | 11.934066317138400 | up |
| **LP_3D1.tig10987.1** | 0 | 3 | 4 | 80 | 49 | 18 | 23.928922239511900 | 4.3036480484507800 | 0.7564023895097850 | 5.689627780314020 | 1.27316588052584E-08 | 1.71907635340835E-06 | 5.7647048335354600 | up |
| **LP_3D1.tig18820.2** | 0 | 1 | 0 | 53 | 50 | 29 | 21.076284443468400 | 6.861631274725900 | 1.464024483232700 | 4.68682822815559 | 2.77471659888846E-06 | 0.00024606671616819700 | 3.6089471264924800 | up |
| **LP_3D2.tig18867.1** | 6 | 7 | 24 | 405 | 318 | 185 | 149.27907306642200 | 4.548071443785860 | 0.43595764351514600 | 10.432370005293500 | 1.76431821264952E-25 | 1.7909405142511E-22 | 21.746918838920100 | up |
| **LP_3D1.tig02564.1** | 1 | 1 | 1 | 28 | 10 | 6 | 7.3101861921104400 | 3.7688006212532000 | 1.1056878142129500 | 3.408557617084630 | 0.0006530729216196570 | 0.030454413641894500 | 1.516349757828660 | up |
| **LP_3D1.tig02957.2** | 0 | 14 | 9 | 157 | 98 | 47 | 50.84265089336710 | 3.658204030276900 | 1.1190831279502400 | 3.268929661174870 | 0.0010795513466346600 | 0.04695263680141330 | 1.3283400132775700 | up |
| **LP_3D3.tig07435.1** | 6 | 9 | 11 | 296 | 262 | 124 | 111.60639050805800 | 4.630961418529090 | 0.4412651039909840 | 10.49473746426980 | 9.13312508346898E-26 | 9.614305469811E-23 | 22.017082083398800 | up |
| **LP_3D2.tig21355.1** | 4 | 5 | 12 | 155 | 65 | 24 | 40.99574948279880 | 3.434018023105140 | 0.604790823963726 | 5.6780260001284500 | 1.36258007559496E-08 | 1.82249092699283E-06 | 5.739334625076800 | up |
| **LP_3D1.tig01494.1** | 0 | 0 | 3 | 80 | 51 | 15 | 23.057269090398700 | 5.5064858864616400 | 1.0011127951986300 | 5.500365106580320 | 3.7900561635656E-08 | 4.63324177672874E-06 | 5.334115036390970 | up |
| **LP_3D2.tig15018.1** | 1 | 3 | 0 | 29 | 26 | 9 | 10.668396061850400 | 3.9073711168342000 | 0.9552783803770470 | 4.090295768330870 | 4.30823435075797E-05 | 0.00287876437615511 | 2.5407938802151200 | up |
| **LP_3D1.tig00185.1** | 1 | 2 | 1 | 53 | 56 | 17 | 20.36962351358060 | 4.88532816109888 | 0.8817468400271950 | 5.5405111074151500 | 3.01590108286822E-08 | 3.77618715981595E-06 | 5.422946488828160 | up |
| **LP_3D1.tig00019.1** | 3 | 8 | 9 | 164 | 103 | 49 | 52.594600701936000 | 3.8957710335870600 | 0.5254396810268590 | 7.414306863869910 | 1.22262536441909E-13 | 3.21759902038902E-11 | 10.492468078970000 | up |
| **LP_3D1.tig07939.2** | 1 | 1 | 1 | 34 | 10 | 8 | 8.56096375963228 | 4.011155524603800 | 1.0851648445173400 | 3.6963559452461500 | 0.0002187162699225390 | 0.011908933298608000 | 1.9241271371969300 | up |
| **LP_3D1.tig00942.1** | 27 | 18 | 16 | 160 | 239 | 237 | 114.0950494146830 | 3.3289365423125400 | 0.37944036494624700 | 8.773279940272390 | 1.73535901593738E-18 | 7.95536155330325E-16 | 15.099340077860700 | up |
| **LP_3D1.tig01332.4** | 0 | 1 | 0 | 424 | 236 | 127 | 122.95976439225300 | 9.414089209764620 | 1.4424480224436200 | 6.526466855849960 | 6.73392091846141E-11 | 1.25917675858533E-08 | 7.899913300956500 | up |
| **LP_3D2.tig05164.1** | 1 | 5 | 2 | 54 | 34 | 19 | 18.077091954490200 | 3.659450696183520 | 0.722838400495567 | 5.06261246452133 | 4.1354995533532E-07 | 4.35337911315487E-05 | 4.3611735107470700 | up |
| **FM1.tig00358.3** | 0 | 8 | 2 | 53 | 38 | 14 | 17.96517894988900 | 3.3124422890505200 | 0.7418260445057000 | 4.465254777159650 | 7.99735254090402E-06 | 0.0006457521380506940 | 3.1899341472265300 | up |
| **C0_3D1.tig01274.1** | 96 | 71 | 94 | 41 | 31 | 22 | 58.87482340245000 | -1.5546947246923000 | 0.36855645837764800 | -4.218335317025590 | 2.4611262794785E-05 | 0.0017487840419619400 | 2.75726381840872 | down |
| **LP_3D1.tig02210.1** | 2 | 5 | 6 | 97 | 85 | 35 | 36.15196430328940 | 3.9793868857766200 | 0.5832674303326910 | 6.822576881254650 | 8.94217447764557E-12 | 1.88965765123332E-09 | 8.723616869714380 | up |
| **C0_3D3.tig14050.2** | 6 | 9 | 23 | 317 | 258 | 107 | 112.97661819568800 | 4.087437222032940 | 0.4603797790693860 | 8.878403022598650 | 6.78270948210833E-19 | 3.27632004044541E-16 | 15.484613681694700 | up |
| **LP_3D3.tig02250.1** | 0 | 0 | 8 | 516 | 423 | 251 | 189.28236072195000 | 7.184473979969100 | 1.5359796062864100 | 4.677454017335070 | 2.9045867941587E-06 | 0.0002567826381243410 | 3.590434343616020 | up |
| **LP_3D1.tig09918.1** | 1 | 5 | 5 | 62 | 23 | 13 | 16.948180107020100 | 3.064278995164870 | 0.7251278532801420 | 4.225846492178570 | 2.38044231951996E-05 | 0.0016999528097124600 | 2.769563134366390 | up |
| **LP_3D1.tig08185.2** | 0 | 1 | 3 | 30 | 20 | 9 | 9.85090497243543 | 3.8034989071101600 | 0.9671472043249600 | 3.9326990659760900 | 8.39973682104727E-05 | 0.005184397824022070 | 2.2853016803341200 | up |
| **LP_3D2.tig23290.1** | 0 | 2 | 3 | 18 | 26 | 6 | 8.63112769015474 | 3.24992440768152 | 0.9476967139635840 | 3.4292874078767800 | 0.0006051683201023250 | 0.028595838035092800 | 1.5436971714137500 | up |
| **LP_3D1.tig00028.7** | 5 | 8 | 3 | 70 | 29 | 14 | 20.149668634944900 | 2.7095510887534600 | 0.6554677384232890 | 4.133767277808700 | 3.56864841043644E-05 | 0.002432371929151790 | 2.61397001721662 | up |
| **LP_3D1.tig00381.4** | 2 | 1 | 2 | 108 | 16 | 6 | 20.48970264547670 | 4.553726859046520 | 0.9604740104204940 | 4.741124496489920 | 2.12535275910802E-06 | 0.00019177091681189800 | 3.7172172554855700 | up |
| **LP_3D1.tig13370.1** | 2 | 2 | 1 | 119 | 106 | 53 | 44.65261487573660 | 5.709987600790650 | 0.7511883875913230 | 7.601272457232280 | 2.93233004344016E-14 | 8.50450516935488E-12 | 11.07035095053710 | up |
| **LP_3D1.tig10056.1** | 0 | 1 | 2 | 32 | 16 | 6 | 8.823968804236980 | 4.076126296208550 | 1.0880658872110800 | 3.7462127469655500 | 0.00017952435736383200 | 0.010044352455066000 | 1.9980780563529200 | up |
| **LP_3D1.tig06261.2** | 1 | 0 | 6 | 46 | 15 | 11 | 12.304386765064500 | 3.2737322867100200 | 0.867570591318445 | 3.773447739549280 | 0.00016100695832133800 | 0.009143297248527950 | 2.038897161092110 | up |
| **LP_3D1.tig01873.2** | 13 | 23 | 19 | 74 | 59 | 39 | 36.37262694818640 | 1.5753124509384500 | 0.4250647811785460 | 3.7060526317205000 | 0.00021051457106429000 | 0.011495389809942000 | 1.939476297171010 | up |
| **LP_3D1.tig00425.1** | 6 | 7 | 12 | 127 | 65 | 32 | 38.95250587504360 | 3.0678509767425300 | 0.5343308886807410 | 5.741481620718270 | 9.38517464496292E-09 | 1.29805414280515E-06 | 5.886707192411530 | up |
| **LP_3D1.tig16131.2** | 0 | 0 | 1 | 15 | 15 | 11 | 6.712496545188900 | 5.185769089264730 | 1.5555406240810400 | 3.3337407001686600 | 0.0008568649725402480 | 0.03841363514515010 | 1.4155145928926600 | up |
| **LP_3D1.tig01415.1** | 22 | 44 | 45 | 216 | 72 | 69 | 74.10847752274790 | 1.6037958193730600 | 0.4406764636694890 | 3.6393952289131600 | 0.0002732790955784730 | 0.01445074436107750 | 1.8401097817252600 | up |
| **LP_3D1.tig00516.3** | 3 | 3 | 1 | 49 | 39 | 28 | 19.607340051557500 | 3.9711293168478100 | 0.7119423497982290 | 5.577880453344660 | 2.43466873994447E-08 | 3.10311086372519E-06 | 5.508202708255990 | up |
| **C0_3D1.tig27778.1** | 7 | 7 | 3 | 122 | 126 | 79 | 54.93054731664110 | 4.187864951394450 | 0.49205643314924900 | 8.510944414630170 | 1.72522332612558E-17 | 7.37370826869238E-15 | 14.132314048694000 | up |
| **C0_3D3.tig18947.1** | 89 | 144 | 134 | 56 | 48 | 27 | 81.65694127018650 | -1.5543661005375400 | 0.3471611587273650 | -4.477361771217670 | 7.55711270323443E-06 | 0.0006154499593343290 | 3.2108072526732800 | down |
| **LP_3D2.tig06213.2** | 3 | 7 | 8 | 60 | 53 | 20 | 23.773238069300900 | 2.8048233225079600 | 0.582099941572825 | 4.818456629508290 | 1.4467296473947E-06 | 0.00013706557801025200 | 3.8630715981440500 | up |
| **LP_3D1.tig25476.1** | 3 | 1 | 0 | 35 | 30 | 23 | 14.73401847795280 | 4.376140346799830 | 0.8838525362615880 | 4.951210939903500 | 7.37531070387013E-07 | 7.36818166891912E-05 | 4.132639674771520 | up |
| **LP_3D1.tig00156.2** | 2 | 3 | 1 | 50 | 27 | 15 | 15.364871899140700 | 3.8411395910896100 | 0.8005521502143670 | 4.798112890036030 | 1.6016750270943E-06 | 0.0001502429322032600 | 3.8232059491243600 | up |
| **LP_3D1.tig00123.4** | 1 | 2 | 0 | 75 | 39 | 26 | 22.430820749906600 | 5.451026983678920 | 0.9640005203872310 | 5.6545892542560900 | 1.56219402976221E-08 | 2.06442243916061E-06 | 5.685201429029200 | up |
| **FM2.tig30154.2** | 13 | 9 | 14 | 240 | 168 | 86 | 83.48821846397130 | 3.6819358005029800 | 0.43354270040824400 | 8.492671649265220 | 2.01940234924062E-17 | 8.50317974389503E-15 | 14.070418640521600 | up |
| **C0_3D1.tig00750.2** | 40 | 78 | 86 | 528 | 137 | 156 | 161.63397973904400 | 1.924546165113990 | 0.43842239277908100 | 4.389707726639230 | 1.13503109841287E-05 | 0.0008838471614969780 | 3.0536228284905900 | up |
| **C0_3D1.tig03238.1** | 85 | 76 | 78 | 235 | 316 | 224 | 164.84137948529000 | 1.6323269657572400 | 0.29356409203611500 | 5.560376796888440 | 2.69192829068928E-08 | 3.40807714218779E-06 | 5.467490583492390 | up |
| **LP_90D2.tig06135.2** | 4 | 19 | 7 | 933 | 788 | 433 | 344.75916427063700 | 6.092071127252380 | 0.42840693052539600 | 14.220290787035300 | 6.85674283345352E-46 | 3.24809621973054E-42 | 41.488371113950900 | up |
| **LP_3D1.tig08134.2** | 8 | 11 | 22 | 210 | 114 | 94 | 72.69790616703920 | 3.278477158800850 | 0.43511068299407000 | 7.534811915536300 | 4.89039193858674E-14 | 1.36271730269099E-11 | 10.865594229723100 | up |
| **LP_3D1.tig00266.4** | 0 | 0 | 1 | 51 | 46 | 12 | 17.103101499415400 | 6.560829353058080 | 1.499910719258150 | 4.374146586740190 | 1.21908444847908E-05 | 0.0009415605363287140 | 3.0261517523883300 | up |
| **LP_3D1.tig02250.1** | 2 | 1 | 0 | 28 | 18 | 8 | 8.943319569872290 | 4.0603774248191300 | 1.0600399063958100 | 3.8304005352257200 | 0.0001279348403501620 | 0.007520637021411520 | 2.123745371753200 | up |
| **LP_3D1.tig00906.1** | 2 | 1 | 2 | 38 | 19 | 8 | 10.907314408718500 | 3.592009614658360 | 0.9004440809532980 | 3.98915345287795 | 6.63095165430047E-05 | 0.0042210128419788400 | 2.374583326549200 | up |
| **LP_3D1.tig01316.2** | 4 | 7 | 10 | 147 | 76 | 39 | 44.222480557747 | 3.5495265672234900 | 0.5397086551427660 | 6.576745681954180 | 4.80856181635123E-11 | 9.14189620235737E-09 | 8.03896371402452 | up |
| **LP_3D1.tig02225.1** | 1 | 6 | 6 | 73 | 69 | 21 | 27.547839998403400 | 3.5673308639570900 | 0.6317568259095930 | 5.646683530203110 | 1.63572533069628E-08 | 2.15237977832014E-06 | 5.667081096817530 | up |
| **LP_3D1.tig02054.1** | 1 | 1 | 3 | 67 | 35 | 12 | 18.422949029173700 | 4.406708904452670 | 0.8590994736506410 | 5.129451291277000 | 2.90587946244998E-07 | 3.1403938791439E-05 | 4.503015877783210 | up |
| **LP_3D1.tig00769.3** | 6 | 7 | 3 | 75 | 159 | 108 | 58.126810890479200 | 4.36510131361647 | 0.5258661951033850 | 8.300783268181540 | 1.0342722157546E-16 | 3.9195469403047E-14 | 13.406764130096100 | up |
| **LP_3D3.tig04152.1** | 5 | 17 | 11 | 56 | 60 | 60 | 33.91064753667070 | 2.377749858034450 | 0.4710754784214290 | 5.047492316947330 | 4.4764676691693E-07 | 4.66052755776427E-05 | 4.33156491969244 | up |
| **LP_3D2.tig19735.1** | 0 | 1 | 0 | 19 | 28 | 9 | 8.988327683294690 | 5.618832325910200 | 1.5374991597744500 | 3.6545270871786900 | 0.00025765646142369800 | 0.013752564835333400 | 1.8616162989278300 | up |
| **LP_3D1.tig00156.1** | 2 | 0 | 0 | 99 | 48 | 33 | 28.521228077704100 | 6.387610555487130 | 1.1195344064080200 | 5.7055955752012400 | 1.15936980027092E-08 | 1.58044067857075E-06 | 5.801221800648750 | up |
| **C0_3D3.tig04409.1** | 33 | 68 | 86 | 331 | 209 | 111 | 132.65838788857400 | 1.7244108966353700 | 0.39063463122323100 | 4.414383054660460 | 1.01298456832422E-05 | 0.0007986561412814190 | 3.0976401644750800 | up |
| **LP_3D1.tig00406.1** | 0 | 3 | 0 | 43 | 27 | 13 | 13.442833154146800 | 4.702535211667430 | 1.020952280885330 | 4.606028410641860 | 4.10432339337224E-06 | 0.00035619887526144200 | 3.448307456204950 | up |
| **LP_3D1.tig03792.2** | 1 | 1 | 1 | 55 | 69 | 68 | 31.681184115155700 | 5.95360080455204 | 0.9230124445111530 | 6.450184761815640 | 1.11713873617462E-10 | 1.99697331628447E-08 | 7.699627738167770 | up |
| **LP_3D1.tig01585.3** | 2 | 1 | 1 | 69 | 52 | 28 | 24.1126057605285 | 5.127938161123760 | 0.850318515592856 | 6.030608609702540 | 1.63343349389178E-09 | 2.51976342290997E-07 | 6.598640232610460 | up |
| **LP_3D2.tig11730.2** | 10 | 16 | 10 | 34 | 51 | 49 | 27.840999091833200 | 1.855307146448650 | 0.48006067546620000 | 3.864734691394810 | 0.00011121001432124400 | 0.006647458742472280 | 2.177344349441640 | up |
| **LP_3D2.tig14911.1** | 0 | 1 | 0 | 28 | 23 | 7 | 9.185023451654900 | 5.653271622952290 | 1.5427101777303600 | 3.664506596611310 | 0.00024781585316790300 | 0.013277183952242600 | 1.8768940276554400 | up |
| **LP_3D1.tig04852.2** | 11 | 0 | 5 | 60 | 63 | 64 | 33.05806325355810 | 3.470957450017130 | 0.5784694542600060 | 6.000243270333550 | 1.97022128240128E-09 | 2.99457831010965E-07 | 6.523664325356930 | up |
| **C0_3D1.tig09178.2** | 11 | 14 | 4 | 119 | 87 | 55 | 46.094886413085800 | 3.079278334470160 | 0.4768197502672790 | 6.457950478653810 | 1.06130456453328E-10 | 1.90315009371906E-08 | 7.720526959320200 | up |
| **LP_3D1.tig04199.2** | 0 | 2 | 1 | 31 | 22 | 8 | 9.97620717292685 | 4.257866748795830 | 1.0532327357337800 | 4.042664649831080 | 5.28471780536152E-05 | 0.0034411200875804700 | 2.4633001711850800 | up |
| **LP_3D1.tig19527.2** | 1 | 1 | 0 | 32 | 20 | 17 | 11.28322084423700 | 5.028640543143420 | 1.1668671836700200 | 4.309522637638480 | 1.63607311630615E-05 | 0.001223718109163460 | 2.9123186130190800 | up |
| **LP_3D2.tig02061.2** | 0 | 3 | 1 | 56 | 44 | 17 | 18.916783730429100 | 4.784250644288090 | 0.8916139195056920 | 5.365832160785980 | 8.05768444969465E-08 | 9.3860465685019E-06 | 5.0275172953831400 | up |
| **C0_90D2.tig25437.1** | 16 | 43 | 28 | 9 | 5 | 2 | 16.846798346001500 | -2.5108853637823500 | 0.638386861964012 | -3.9331720519083900 | 8.3832187737393E-05 | 0.005180563759795710 | 2.285622976848200 | down |
| **LP_3D1.tig05705.1** | 7 | 11 | 10 | 111 | 29 | 28 | 30.726425429221300 | 2.4862562165821800 | 0.5707037279688620 | 4.3564744625565800 | 1.32174106087504E-05 | 0.0010098705726537900 | 2.9957342828352700 | up |
| **LP_3D1.tig01045.2** | 8 | 8 | 12 | 55 | 25 | 54 | 26.44435538739390 | 2.216028655728770 | 0.5235722581327470 | 4.2325173293786600 | 2.31090100888557E-05 | 0.001658625856693180 | 2.780251568586130 | up |
| **LP_3D2.tig30016.1** | 1 | 1 | 1 | 30 | 29 | 6 | 10.584656565942200 | 4.334101238026580 | 1.0540443253281000 | 4.111877587953890 | 3.92454379104884E-05 | 0.002649533156795390 | 2.576830641266400 | up |
| **LP_3D1.tig11574.1** | 3 | 2 | 5 | 38 | 21 | 14 | 13.119718829653900 | 2.781935268853580 | 0.7164108649603490 | 3.883156167665820 | 0.00010310929751666000 | 0.006215533422412030 | 2.2065215938977900 | up |
| **LP_3D1.tig00149.4** | 0 | 6 | 5 | 74 | 60 | 21 | 25.945122319671700 | 3.7385811250736400 | 0.6670719515045040 | 5.604464580832250 | 2.08899701396444E-08 | 2.69272188795484E-06 | 5.569808499452850 | up |
| **LP_3D1.tig03256.2** | 1 | 2 | 4 | 39 | 23 | 9 | 12.16323606265530 | 3.251870986825590 | 0.815006828021343 | 3.9899923227887700 | 6.60754339319317E-05 | 0.004210827401189080 | 2.375632559635870 | up |
| **LP_3D1.tig02167.1** | 3 | 9 | 5 | 149 | 70 | 38 | 42.74416457243910 | 3.823388451454700 | 0.5728217132487970 | 6.674656988419820 | 2.47811080607824E-11 | 4.90829995719573E-09 | 8.30906890442623 | up |
| **C0_3D1.tig19155.1** | 12 | 11 | 10 | 38 | 23 | 49 | 23.598944133966300 | 1.6959240321893500 | 0.5188561328645890 | 3.2685824157579300 | 0.001080876936391500 | 0.046974349731784900 | 1.328139223145530 | up |
| **LP_3D1.tig01756.2** | 1 | 3 | 6 | 77 | 37 | 20 | 22.462239208986000 | 3.6573000589250200 | 0.690154990820321 | 5.2992445285050200 | 1.16282814125637E-07 | 1.32999930965228E-05 | 4.876148584457190 | up |
| **LP_3D2.tig21288.1** | 0 | 1 | 2 | 35 | 20 | 12 | 10.972143055884100 | 4.40116142023325 | 1.0298248558238900 | 4.273698964774150 | 1.92256567070137E-05 | 0.001410170910335730 | 2.8507282483960200 | up |
| **LP_3D1.tig06851.2** | 0 | 1 | 0 | 39 | 23 | 14 | 12.075478997272300 | 6.052320386946160 | 1.5070551466429700 | 4.015991319513390 | 5.91964345192005E-05 | 0.0038108961724167000 | 2.4189728833875700 | up |
| **C0_3D2.tig16597.2** | 6 | 13 | 12 | 192 | 138 | 69 | 67.66322991056830 | 3.603365745070710 | 0.4554061544440760 | 7.91242215307656 | 2.52428717410959E-15 | 8.19960596641485E-13 | 12.086207017216700 | up |
| **LP_3D1.tig02054.4** | 0 | 1 | 0 | 79 | 55 | 23 | 24.651347455204300 | 7.091205577982980 | 1.4731349674073100 | 4.813683562520660 | 1.48173369712176E-06 | 0.00014014833945571800 | 3.853412043601100 | up |
| **LP_3D2.tig07878.1** | 0 | 1 | 0 | 33 | 13 | 6 | 8.182596270098120 | 5.484712189678130 | 1.5697488364710900 | 3.494006214400620 | 0.0004758299421015180 | 0.023197729896021300 | 1.634554512603450 | up |
| **LP_3D1.tig00871.1** | 0 | 0 | 5 | 65 | 42 | 20 | 20.632292934850200 | 4.5863687177636700 | 0.8409786284749500 | 5.453609119747430 | 4.93576342392141E-08 | 5.93178587384382E-06 | 5.226814534563160 | up |
| **LP_3D1.tig05809.2** | 0 | 1 | 1 | 35 | 25 | 7 | 10.699501731774300 | 4.969741539198550 | 1.2088801911474100 | 4.111029013124560 | 3.93899695660481E-05 | 0.002656136204011870 | 2.5757496585457000 | up |
| **LP_3D1.tig00081.4** | 0 | 4 | 2 | 81 | 32 | 14 | 20.5395802496688 | 4.305182150417130 | 0.827533237139626 | 5.202428080469640 | 1.96701490196106E-07 | 2.18815972802303E-05 | 4.659920979200080 | up |
| **LP_3D1.tig21241.1** | 515 | 770 | 570 | 3465 | 1084 | 1570 | 1276.194725888670 | 1.6422332089267400 | 0.3550138894429500 | 4.6258280528222700 | 3.73104741975169E-06 | 0.00032529354382789100 | 3.4877245561701800 | up |
| **C0_3D1.tig00655.2** | 204 | 286 | 218 | 685 | 1162 | 787 | 543.3074058010870 | 1.8443656558353400 | 0.29029169692338800 | 6.35349090374461 | 2.10482640121668E-10 | 3.60388122822778E-08 | 7.443229530338720 | up |
| **LP_3D1.tig03460.1** | 0 | 0 | 5 | 88 | 69 | 27 | 29.53796571529880 | 5.1403450931700000 | 1.3020914450038900 | 3.9477604379426900 | 7.8885682254755E-05 | 0.0049277545140346700 | 2.307350935554600 | up |
| **LP_3D1.tig02048.2** | 30 | 75 | 55 | 1846 | 1056 | 454 | 547.7929173239260 | 4.299802205003280 | 0.4058081246867200 | 10.595653323409200 | 3.12153670918806E-26 | 3.5488750846759E-23 | 22.449909286927600 | up |
| **LP_3D1.tig20180.1** | 0 | 1 | 3 | 51 | 42 | 13 | 17.138071984916100 | 4.640779248012830 | 0.9116229013119520 | 5.0906786581755500 | 3.56784225135852E-07 | 3.81947255703343E-05 | 4.41799660603734 | up |
| **LP_3D1.tig03536.3** | 3 | 9 | 3 | 97 | 54 | 42 | 32.90021206326290 | 3.6067800561747100 | 0.5687625041312160 | 6.341451888928690 | 2.276098393161E-10 | 3.88542982460171E-08 | 7.410560930498290 | up |
| **LP_3D1.tig00088.6** | 4 | 8 | 7 | 215 | 161 | 113 | 80.59798233356760 | 4.612539256398840 | 0.46515267670565600 | 9.91618341114613 | 3.54035725555443E-23 | 2.7196163269188E-20 | 19.565492360250700 | up |
| **LP_3D1.tig00088.4** | 1 | 1 | 0 | 45 | 40 | 24 | 17.604831878722400 | 5.687787153478360 | 1.1276530631329300 | 5.0439158456024800 | 4.56099958394333E-07 | 4.73901620529267E-05 | 4.324311806207300 | up |
| **C0_3D1.tig25961.2** | 23 | 41 | 48 | 286 | 220 | 110 | 115.23025273414800 | 2.382712969639890 | 0.3739554952064450 | 6.37164849877256 | 1.87007097683218E-10 | 3.25089249779895E-08 | 7.487997391741850 | up |
| **LP_3D1.tig00385.2** | 7 | 8 | 11 | 524 | 303 | 149 | 156.4391668963470 | 5.132140177496450 | 0.466827229161468 | 10.993660731219500 | 4.0995403376882E-28 | 5.68386269502161E-25 | 24.245356421862600 | up |
| **LP_3D2.tig06705.1** | 1 | 1 | 0 | 85 | 59 | 29 | 27.426957834989600 | 6.339009930729470 | 1.1163739386591600 | 5.6782138235357500 | 1.36108509460623E-08 | 1.82249092699283E-06 | 5.739334625076800 | up |
| **LP_3D1.tig02048.3** | 1 | 2 | 2 | 424 | 260 | 85 | 119.85718737812300 | 7.156790064724460 | 0.7746923559681400 | 9.238235035610440 | 2.50596555011495E-20 | 1.43890516864934E-17 | 16.841967827390300 | up |
| **LP_3D2.tig11498.1** | 0 | 4 | 5 | 63 | 51 | 27 | 23.649301436860200 | 3.901045887219840 | 0.6725091491924310 | 5.800732810705 | 6.60257343899876E-09 | 9.29121714077603E-07 | 6.031927390107720 | up |
| **LP_3D1.tig09553.2** | 2 | 6 | 4 | 116 | 84 | 48 | 40.99321587362300 | 4.2893431474934900 | 0.5751235016894300 | 7.458125315507900 | 8.77621811762084E-14 | 2.37563866141027E-11 | 10.624219615580400 | up |
| **LP_3D2.tig02994.2** | 314 | 225 | 269 | 1089 | 1337 | 974 | 682.5142949266510 | 2.0008079528397600 | 0.2702317237617890 | 7.40404540587353 | 1.3209722366869E-13 | 3.46039939145008E-11 | 10.460873773030100 | up |
| **LP_3D1.tig06555.1** | 1 | 0 | 1 | 20 | 25 | 2 | 7.591355997898140 | 4.439352421707800 | 1.2895584894452100 | 3.442536696119680 | 0.0005762856727222980 | 0.027351311673618100 | 1.5630218415808300 | up |
| **LP_3D2.tig11645.1** | 4 | 4 | 1 | 24 | 38 | 17 | 14.104905574640600 | 3.0533870334529200 | 0.7216084437114410 | 4.231362673291990 | 2.32279792841314E-05 | 0.0016650623989993100 | 2.7785694864619900 | up |
| **LP_3D1.tig00185.6** | 0 | 0 | 2 | 51 | 24 | 12 | 13.816050860167000 | 5.3483269623021800 | 1.185893652281630 | 4.509954962666440 | 6.48413902408239E-06 | 0.0005349649968417470 | 3.2716746332606300 | up |
| **LP_3D1.tig04398.1** | 3 | 1 | 0 | 89 | 61 | 34 | 29.59379214093760 | 5.423159945180770 | 0.8446028633361010 | 6.4209585126905700 | 1.35418934310267E-10 | 2.39064885741215E-08 | 7.6214842091152600 | up |
| **C0_90D2.tig00036.10** | 9 | 49 | 54 | 5 | 10 | 2 | 20.90184126971420 | -2.7615962068956600 | 0.6777747160747640 | -4.074504612519400 | 4.6112399371041E-05 | 0.0030515242633839600 | 2.51548317251654 | down |
| **LP_3D1.tig18540.1** | 2 | 1 | 1 | 34 | 25 | 9 | 11.271705832650500 | 3.98238332684138 | 0.9398983257707270 | 4.2370363023849200 | 2.26489527964865E-05 | 0.0016317867195390100 | 2.7873366057416900 | up |
| **LP_3D1.tig00062.5** | 3 | 2 | 7 | 112 | 97 | 47 | 42.26749636785220 | 4.333001054836240 | 0.5786364431600910 | 7.4882961591090700 | 6.977344522812E-14 | 1.91607318550361E-11 | 10.717587906816300 | up |
| **C0_3D1.tig02919.2** | 54 | 43 | 46 | 15 | 11 | 20 | 31.67316582158420 | -1.6883974913173600 | 0.4527448399151290 | -3.729247343015260 | 0.00019205256529234600 | 0.010609551092364800 | 1.9743029914269600 | down |
| **LP_3D1.tig01698.2** | 0 | 5 | 3 | 62 | 30 | 20 | 18.79813879408550 | 3.7298028221534100 | 0.7385770815138280 | 5.049984511445450 | 4.41845888237787E-07 | 4.61704586707298E-05 | 4.335635811020060 | up |
| **LP_3D1.tig06991.1** | 0 | 5 | 1 | 33 | 10 | 13 | 9.776858471364690 | 3.1534066785825200 | 0.9060705280840700 | 3.4803103962012200 | 0.0005008331759608870 | 0.024312435429117500 | 1.6141715347622000 | up |
| **LP_3D1.tig13991.1** | 0 | 4 | 2 | 39 | 35 | 16 | 15.121536646896000 | 3.8345538579343100 | 0.7995794954849570 | 4.795713096180130 | 1.62097145884543E-06 | 0.00015180250836584600 | 3.81872105215302 | up |
| **LP_3D1.tig00393.2** | 4 | 13 | 9 | 174 | 109 | 37 | 53.86501646519450 | 3.5270488379797500 | 0.5362678184941590 | 6.577028709057550 | 4.79942136646595E-11 | 9.14189620235737E-09 | 8.03896371402452 | up |
| **LP_3D1.tig32322.1** | 3 | 0 | 2 | 42 | 36 | 26 | 17.410427953415500 | 4.298242978602350 | 0.8053585901321120 | 5.3370548613596600 | 9.44684631197326E-08 | 1.08705663685044E-05 | 4.963747828102440 | up |
| **LP_3D1.tig00722.4** | 28 | 29 | 26 | 281 | 466 | 380 | 196.85389066429400 | 3.714503478536340 | 0.3387868565454270 | 10.964131006771400 | 5.68439910800072E-28 | 7.51464342544885E-25 | 24.124091622134200 | up |
| **LP_3D1.tig05027.2** | 1 | 1 | 0 | 25 | 15 | 5 | 7.308201091980660 | 4.380631055711840 | 1.2525653675485600 | 3.4973273005985500 | 0.00046994487619181900 | 0.02298968716619960 | 1.6384669383835100 | up |
| **LP_3D1.tig01874.4** | 6 | 6 | 4 | 72 | 78 | 33 | 31.5460254047637 | 3.4275034050547600 | 0.5510179450477250 | 6.220311762728350 | 4.96168002613896E-10 | 8.1516387597072E-08 | 7.088755074357610 | up |
| **LP_3D1.tig00271.5** | 4 | 3 | 7 | 101 | 82 | 42 | 37.72889660670880 | 3.922530524698000 | 0.5574788135260710 | 7.036196586356110 | 1.9755816107671E-12 | 4.49207746656223E-10 | 9.347552762871310 | up |
| **C0_3D3.tig29403.1** | 218 | 486 | 783 | 2073 | 1753 | 823 | 974.2339351820270 | 1.582922870120370 | 0.38259521151654100 | 4.137330584577720 | 3.51369701189264E-05 | 0.0024035632568115200 | 2.619144443652800 | up |
| **LP_3D3.tig12971.2** | 29 | 21 | 34 | 67 | 103 | 77 | 54.011141496187700 | 1.5003350413364000 | 0.38430031839342300 | 3.9040692123508800 | 9.45887110475125E-05 | 0.005763017448548600 | 2.23935006551375 | up |
| **C0_3D1.tig02954.2** | 45 | 34 | 30 | 207 | 384 | 280 | 159.61277936716700 | 2.9374275817366700 | 0.3540871967108120 | 8.295774625637500 | 1.07879892676283E-16 | 4.06121357561808E-14 | 13.39134417074800 | up |
| **LP_3D1.tig04388.1** | 14 | 39 | 35 | 192 | 32 | 83 | 62.918000197376600 | 1.739673660347470 | 0.5181734916870230 | 3.357318906228870 | 0.0007870228020907050 | 0.03596327265662870 | 1.4441407943569400 | up |
| **LP_3D2.tig29577.1** | 1 | 2 | 0 | 39 | 24 | 10 | 11.85904261303810 | 4.5034228881797000 | 1.0304555246569900 | 4.370322425782300 | 1.2406321024966E-05 | 0.0009569027390287520 | 3.0191322022960000 | up |
| **LP_3D2.tig08997.1** | 14 | 12 | 17 | 53 | 39 | 68 | 33.386900008727200 | 1.85743873310183 | 0.45823667246190600 | 4.053448457371650 | 5.04681232747761E-05 | 0.0033089509429696100 | 2.4803096714486500 | up |
| **LP_3D1.tig08904.1** | 0 | 1 | 0 | 48 | 23 | 10 | 12.697182337717600 | 6.127393918047390 | 1.522269107806850 | 4.025171296338780 | 5.69338566156837E-05 | 0.0036896013216945800 | 2.4330205588117400 | up |
| **LP_3D1.tig02829.4** | 2 | 0 | 0 | 46 | 30 | 18 | 15.130251498069400 | 5.454685246189740 | 1.148702457089520 | 4.748562356182590 | 2.0486772494868E-06 | 0.00018633129319532300 | 3.7297142018897500 | up |
| **LP_3D2.tig18785.1** | 3 | 4 | 6 | 270 | 132 | 84 | 78.04590474505320 | 5.131028523127410 | 0.5608685503943960 | 9.148361981643180 | 5.78033241084627E-20 | 3.1594518836015E-17 | 16.50038825427230 | up |
| **LP_3D1.tig00106.5** | 0 | 0 | 2 | 35 | 21 | 12 | 10.966370009923500 | 5.005498540719060 | 1.1907732252657400 | 4.203569944732350 | 2.62737912585498E-05 | 0.0018599422965034400 | 2.7305005292761000 | up |
| **LP_3D1.tig04891.2** | 0 | 2 | 0 | 17 | 13 | 8 | 6.323846929056920 | 4.175630385880870 | 1.2601207283325800 | 3.313674866221870 | 0.0009207851017534930 | 0.040783129799982000 | 1.3895194484278200 | up |
| **LP_3D1.tig00760.4** | 0 | 4 | 3 | 67 | 52 | 31 | 24.797685151272100 | 4.351466299066810 | 0.7080143394141310 | 6.14601436274238 | 7.94540923623742E-10 | 1.2546021889831E-07 | 6.901493959057210 | up |
| **LP_3D1.tig01443.2** | 0 | 4 | 0 | 60 | 25 | 13 | 15.820273184276500 | 4.518047141530870 | 0.9476267918942220 | 4.767749477090750 | 1.86295235316528E-06 | 0.0001710816260350250 | 3.7667966306698300 | up |
| **C0_3D3.tig32528.1** | 121 | 186 | 412 | 24 | 79 | 67 | 146.0328205704700 | -2.0832037953763900 | 0.4565775433534670 | -4.5626505852120800 | 5.05118402960548E-06 | 0.0004311329672115970 | 3.365388766914930 | down |
| **LP_3D2.tig18262.1** | 0 | 0 | 2 | 24 | 22 | 7 | 8.59101758243887 | 4.644160281915880 | 1.2300696165579400 | 3.7755263762318200 | 0.00015967012680524500 | 0.00908553389213626 | 2.0416495472116400 | up |
| **LP_3D3.tig21863.1** | 3 | 6 | 7 | 141 | 105 | 54 | 49.73834417102400 | 4.146714228375040 | 0.5287308882331550 | 7.842769016639060 | 4.40718228244778E-15 | 1.38412307649582E-12 | 11.858825290611600 | up |
| **LP_3D1.tig23315.1** | 0 | 2 | 1 | 17 | 12 | 11 | 6.864810710215630 | 3.6802157448817200 | 1.0841366914350800 | 3.3946049183246500 | 0.0006872770058240780 | 0.03180194074329330 | 1.4975463759783200 | up |
| **LP_3D1.tig05957.1** | 1 | 0 | 1 | 104 | 76 | 46 | 35.960526834827000 | 6.734434937860160 | 1.0968432817788700 | 6.139833328730620 | 8.26081089733565E-10 | 1.30079167717187E-07 | 6.8857922504784 | up |
| **LP_3D1.tig01972.1** | 1 | 3 | 3 | 49 | 45 | 28 | 20.50498183302550 | 4.055392088095780 | 0.708829556231665 | 5.721251395970810 | 1.05742297585784E-08 | 1.45542879086293E-06 | 5.837009038244290 | up |
| **LP_3D1.tig10523.1** | 0 | 4 | 2 | 47 | 25 | 20 | 15.461334255293000 | 3.8681022507369100 | 0.7978902336993950 | 4.847912767151660 | 1.24767238501786E-06 | 0.00011940056687936000 | 3.922993611297160 | up |
| **LP_3D1.tig00136.6** | 1 | 3 | 6 | 151 | 101 | 33 | 45.82557155268380 | 4.738863472208320 | 0.6528328010688200 | 7.258923669965480 | 3.90182565404481E-13 | 9.68555804821735E-11 | 10.01387535168420 | up |
| **LP_3D1.tig06084.2** | 1 | 2 | 3 | 92 | 47 | 28 | 27.051137447150200 | 4.7081843067251200 | 0.7539215330683970 | 6.244926163022840 | 4.24000362567606E-10 | 7.00648273551033E-08 | 7.154499943788820 | up |
| **LP_3D1.tig02123.3** | 0 | 0 | 2 | 37 | 25 | 18 | 12.965817667499800 | 5.250228287186450 | 1.161689666788510 | 4.519475757842210 | 6.19929453177327E-06 | 0.0005137010169951180 | 3.28928957448409 | up |
| **C0_3D1.tig00961.8** | 35 | 34 | 20 | 11 | 5 | 2 | 17.84582352534980 | -2.4078968459691400 | 0.6179453466188630 | -3.896617814413740 | 9.75453664646085E-05 | 0.005924109355428060 | 2.2273769332668600 | down |
| **LP_3D1.tig00328.2** | 0 | 4 | 2 | 43 | 30 | 21 | 15.82906245573910 | 3.9029375924531400 | 0.7849136653344400 | 4.9724418937083400 | 6.61147908810598E-07 | 6.67548008460719E-05 | 4.175517495416840 | up |
| **LP_3D2.tig16453.1** | 2 | 7 | 7 | 98 | 107 | 67 | 45.96738969124580 | 4.027630370963710 | 0.511621158773109 | 7.8722904670755900 | 3.48206550661903E-15 | 1.1182938628461E-12 | 11.951444058486500 | up |
| **LP_3D1.tig09738.1** | 0 | 1 | 1 | 146 | 147 | 56 | 55.104978783936800 | 7.360348209148290 | 1.0960623514840500 | 6.715264144583090 | 1.87725842050852E-11 | 3.78414024517045E-09 | 8.422032776452340 | up |
| **LP_3D1.tig05599.1** | 4 | 6 | 1 | 46 | 24 | 16 | 15.329006354137900 | 2.8703209399736000 | 0.6993388959560270 | 4.104334760402180 | 4.05479965471479E-05 | 0.0027309844356903100 | 2.56368077496575 | up |
| **LP_3D1.tig13522.1** | 5 | 7 | 5 | 56 | 20 | 12 | 16.45649790163290 | 2.2665758170364300 | 0.6615041552949330 | 3.4263969453462800 | 0.0006116458108767600 | 0.02884123595312160 | 1.5399861324366900 | up |
| **LP_3D1.tig01387.1** | 8 | 9 | 18 | 410 | 217 | 82 | 115.46627992318700 | 4.238068982643950 | 0.49544119956686700 | 8.55413111858487 | 1.18759856341758E-17 | 5.11432123768731E-15 | 14.291211996807600 | up |
| **FM3.tig00480.3** | 13 | 14 | 23 | 1 | 2 | 5 | 9.669392088530930 | -2.6637890223724600 | 0.8013300087702130 | -3.3242097428256000 | 0.0008866948127507080 | 0.039532679710442300 | 1.403043746142380 | down |
| **C0_3D1.tig09957.2** | 50 | 49 | 44 | 12 | 20 | 15 | 31.670786866136500 | -1.6646482531241000 | 0.44061006238139500 | -3.778053193172800 | 0.0001580591232667310 | 0.009002876615327950 | 2.0456187018325400 | down |
| **LP_3D1.tig04911.1** | 0 | 0 | 1 | 65 | 59 | 25 | 23.551897332006800 | 7.024475277840230 | 1.467898695273970 | 4.785395136909740 | 1.70651266393136E-06 | 0.00015773449167671300 | 3.8020733294550800 | up |
| **LP_3D1.tig13150.2** | 1 | 1 | 1 | 36 | 36 | 7 | 12.753020937170000 | 4.6145755593345000 | 1.0369059869142100 | 4.45033167670993 | 8.573777095483E-06 | 0.0006864455760461000 | 3.163393889541300 | up |
| **LP_3D1.tig00948.1** | 0 | 2 | 0 | 73 | 44 | 30 | 23.452372797213800 | 6.117465827244040 | 1.1226494652501800 | 5.44913262474211 | 5.06160597752449E-08 | 6.07018970026117E-06 | 5.216797736521230 | up |
| **LP_3D1.tig05927.2** | 1 | 0 | 5 | 40 | 18 | 11 | 11.722060654197600 | 3.4367565037847200 | 0.8729987534190620 | 3.936725556966510 | 8.26009807770914E-05 | 0.005120450111530820 | 2.2906918608284700 | up |
| **LP_3D1.tig02253.1** | 1 | 1 | 3 | 85 | 29 | 22 | 21.952007047714100 | 4.668397594327380 | 0.8364709048157710 | 5.58106392876339 | 2.39051751604162E-08 | 3.06056234683302E-06 | 5.514198769045120 | up |
| **C0_3D1.tig04868.2** | 237 | 351 | 357 | 1372 | 683 | 938 | 636.27524786503 | 1.608307791846560 | 0.2969390498093850 | 5.416289278486550 | 6.0848626228771E-08 | 7.26668100414809E-06 | 5.138663904232090 | up |
| **C0_3D1.tig03304.1** | 590 | 885 | 556 | 303 | 209 | 204 | 454.0588716421870 | -1.5700374784956600 | 0.28319700694433500 | -5.543976242673590 | 2.95678970356514E-08 | 3.710346814551E-06 | 5.430585493995160 | down |
| **LP_3D3.tig31163.1** | 0 | 0 | 2 | 37 | 49 | 17 | 16.54705142682310 | 5.6099520595680000 | 1.1530508300522600 | 4.865312016915780 | 1.14276381425551E-06 | 0.0001106650920295640 | 3.955989350483120 | up |
| **LP_3D1.tig05042.3** | 1 | 2 | 4 | 29 | 16 | 11 | 9.937592319788390 | 2.9245924923382000 | 0.8210432138275810 | 3.562044534421260 | 0.00036797794295121900 | 0.018593516592944000 | 1.7306384643174800 | up |
| **FM1.tig03101.2** | 5 | 7 | 11 | 115 | 74 | 23 | 36.62379353010990 | 3.1056801950403500 | 0.5631540165000240 | 5.514797202978340 | 3.49182362819805E-08 | 4.31505900315039E-06 | 5.365013261462690 | up |
| **LP_3D1.tig12419.1** | 2 | 1 | 4 | 38 | 28 | 32 | 16.93854026430290 | 3.7522290796875600 | 0.727881157721417 | 5.155002351529010 | 2.53627746390794E-07 | 2.7725902391509E-05 | 4.557114310020090 | up |
| **C0_3D1.tig09957.3** | 73 | 47 | 52 | 27 | 21 | 13 | 38.824107655833100 | -1.5878495319642200 | 0.42888256417805500 | -3.702294438122710 | 0.0002136584552585990 | 0.011655868415715000 | 1.933455364005010 | down |
| **LP_3D1.tig01886.1** | 0 | 2 | 0 | 57 | 30 | 7 | 14.757348645962300 | 5.443350356329910 | 1.209220447730680 | 4.501536809558060 | 6.74638782118207E-06 | 0.0005533887672367890 | 3.2569696604762200 | up |
| **LP_3D1.tig00266.6** | 0 | 0 | 3 | 57 | 24 | 22 | 16.660522331624800 | 5.024344966352660 | 0.9983172665049240 | 5.0328138507938700 | 4.83332402660674E-07 | 5.00455927672969E-05 | 4.300634162362890 | up |
| **LP_3D1.tig02242.1** | 2 | 0 | 4 | 67 | 39 | 26 | 21.73743558747260 | 4.372562171355670 | 0.7636928078666020 | 5.72555106754318 | 1.03098525884694E-08 | 1.42248439415423E-06 | 5.846952489481140 | up |
| **LP_3D1.tig00213.1** | 777 | 1071 | 967 | 5020 | 1742 | 2201 | 1883.5620253777700 | 1.590396670944190 | 0.3373680823174550 | 4.714129030877510 | 2.42746811048313E-06 | 0.00021696450430882700 | 3.6636113115143500 | up |
| **LP_3D1.tig01544.1** | 69 | 124 | 135 | 2630 | 1619 | 912 | 862.517208254009 | 3.8954327780187700 | 0.34372592827264500 | 11.332961693040800 | 9.01054612495173E-30 | 1.50648380727318E-26 | 25.822035532062100 | up |
| **LP_3D1.tig02638.2** | 38 | 27 | 27 | 82 | 70 | 151 | 65.8188005640768 | 1.6876862551258200 | 0.42313160042310900 | 3.988561131898970 | 6.64752733302585E-05 | 0.004226830998275780 | 2.3739851162551000 | up |
| **LP_3D1.tig15396.2** | 1 | 0 | 0 | 42 | 26 | 19 | 13.906557317263400 | 6.255165461612310 | 1.4911819633014000 | 4.1947700653270300 | 2.73148531536296E-05 | 0.0019216742914827700 | 2.7163202198925900 | up |
| **LP_3D1.tig17836.1** | 4 | 10 | 9 | 44 | 17 | 36 | 19.448945168096900 | 2.034837754115400 | 0.5826347190639210 | 3.4924759674202700 | 0.00047856465787138600 | 0.023311060819793400 | 1.6324379625283400 | up |
| **LP_3D1.tig00027.10** | 1 | 0 | 4 | 23 | 16 | 19 | 10.153988445754700 | 3.486017009436450 | 0.8879789212053320 | 3.9257880183738700 | 8.64462621938885E-05 | 0.00532975897441604 | 2.2732924304570900 | up |
| **C0_3D1.tig17099.2** | 26 | 31 | 26 | 91 | 141 | 90 | 65.66496858518570 | 1.8960846993943100 | 0.3652394132673520 | 5.1913474573632400 | 2.0877759786033E-07 | 2.31796143561923E-05 | 4.6348937937551200 | up |
| **LP_3D1.tig00603.4** | 0 | 1 | 0 | 37 | 15 | 8 | 9.449290879550770 | 5.695392584910870 | 1.548495696146020 | 3.6780164123709800 | 0.00023505483823583800 | 0.012677127399920500 | 1.8969791451686900 | up |
| **LP_3D2.tig03814.2** | 11 | 9 | 17 | 54 | 53 | 49 | 31.29310366312380 | 2.021314824123490 | 0.4520844900194080 | 4.471099692087910 | 7.7818410261876E-06 | 0.0006310395907755130 | 3.199943392711560 | up |
| **LP_3D1.tig06324.1** | 19 | 26 | 20 | 67 | 86 | 46 | 42.55143635660130 | 1.5449561599082800 | 0.40376955370943900 | 3.8263314945734100 | 0.0001300671109798510 | 0.0076144849883106300 | 2.1183594651839600 | up |
| **LP_3D1.tig00463.1** | 1 | 10 | 14 | 298 | 157 | 89 | 88.91583366922160 | 4.368203688492400 | 0.5187771894192900 | 8.42019228598329 | 3.75866891747714E-17 | 1.53713334254668E-14 | 13.813288456885200 | up |
| **C0_3D2.tig07573.1** | 149 | 458 | 310 | 1935 | 509 | 512 | 610.3135115334680 | 1.6044596146924700 | 0.4451701073452600 | 3.604149488519240 | 0.00031317677567736400 | 0.016228380869079100 | 1.7897248082515200 | up |
| **LP_3D1.tig00106.2** | 1 | 1 | 0 | 194 | 110 | 38 | 53.241978469504200 | 7.3035418294823000 | 1.1203987142089200 | 6.51869886751798 | 7.09198671350429E-11 | 1.32178355648902E-08 | 7.878839655227350 | up |
| **LP_3D1.tig08479.1** | 1 | 4 | 4 | 26 | 34 | 10 | 12.45745192867490 | 2.8853670840648700 | 0.7568752377611860 | 3.8122096484484000 | 0.00013772997602995200 | 0.008005378821495510 | 2.096618112065430 | up |
| **LP_3D1.tig00381.1** | 0 | 1 | 0 | 72 | 12 | 13 | 15.08071133466460 | 6.377405539194640 | 1.5433905734097600 | 4.1320749582558700 | 3.59503135208717E-05 | 0.002444492311117170 | 2.6118113244237200 | up |
| **LP_3D1.tig00051.2** | 1 | 2 | 0 | 92 | 69 | 31 | 30.555771307942400 | 5.905601438222690 | 0.9454643439216470 | 6.246244478905600 | 4.20438924868177E-10 | 6.96788649683135E-08 | 7.156898932369120 | up |
| **LP_3D1.tig04065.1** | 5 | 6 | 11 | 93 | 72 | 29 | 33.95123407876960 | 3.0532616772662 | 0.5358568089725430 | 5.697905907215320 | 1.21287954994563E-08 | 1.64942913915452E-06 | 5.782666337369450 | up |
| **LP_3D1.tig00025.8** | 6 | 8 | 11 | 165 | 74 | 39 | 47.27118176534880 | 3.3758671890098600 | 0.5304170092586900 | 6.364553040499160 | 1.95859232454901E-10 | 3.36363083652533E-08 | 7.473191674722410 | up |
| **LP_3D3.tig31737.2** | 1 | 1 | 0 | 18 | 12 | 7 | 6.15547759147177 | 4.116779976965480 | 1.2556407711923900 | 3.278628785728310 | 0.0010431275453134600 | 0.04547284149796310 | 1.3422479068389100 | up |
| **LP_3D1.tig02360.2** | 0 | 5 | 4 | 47 | 33 | 10 | 15.405227104199200 | 3.2389260087803200 | 0.7668917610058380 | 4.223446089096350 | 2.40594941559206E-05 | 0.0017138620868337100 | 2.766024128350330 | up |
| **LP_3D1.tig06664.2** | 0 | 1 | 3 | 76 | 34 | 13 | 19.602755916398300 | 4.842886014198410 | 0.9261935371972680 | 5.228805665016140 | 1.70608581125344E-07 | 1.91286879567459E-05 | 4.718314817358310 | up |
| **LP_3D1.tig15188.1** | 6 | 4 | 3 | 60 | 17 | 15 | 16.49281401728590 | 2.7071651195840000 | 0.6990335918147590 | 3.8727253615322600 | 0.00010762508161091600 | 0.006460346107890730 | 2.1897442144011100 | up |
| **LP_3D1.tig02907.1** | 2 | 0 | 2 | 44 | 47 | 15 | 17.278862044001200 | 4.631212560398400 | 0.8954206159623110 | 5.172108479344340 | 2.31467132697143E-07 | 2.5598733770757E-05 | 4.591781516327740 | up |
| **LP_3D1.tig00254.2** | 0 | 3 | 7 | 47 | 39 | 21 | 18.471786164104700 | 3.357309683557700 | 0.6913507774066230 | 4.856159554996890 | 1.19684275761424E-06 | 0.00011530599111410000 | 3.9381481268753100 | up |
| **LP_3D2.tig07643.1** | 6 | 8 | 10 | 222 | 225 | 118 | 93.40553922863100 | 4.4816121820119600 | 0.4382631294470830 | 10.225848082785300 | 1.51892538623329E-24 | 1.41546415705625E-21 | 20.849101123536400 | up |
| **LP_3D1.tig10484.1** | 2 | 5 | 5 | 93 | 68 | 34 | 32.554180078289800 | 3.9399718428348500 | 0.5960830231399420 | 6.609770266699690 | 3.84916887253674E-11 | 7.41715269692716E-09 | 8.129762780075410 | up |
| **LP_3D1.tig09826.1** | 0 | 3 | 0 | 30 | 26 | 9 | 10.635866309995900 | 4.351761866302780 | 1.0528418506680400 | 4.133348103080750 | 3.575166082103E-05 | 0.002433895999247250 | 2.6136979831799100 | up |
| **LP_3D1.tig11212.1** | 10 | 9 | 8 | 775 | 1323 | 988 | 504.17087316824400 | 6.7843330839617400 | 0.4052268203153860 | 16.742063318221500 | 6.47058642408854E-63 | 1.83910242638657E-58 | 57.73539408262940 | up |
| **LP_3D1.tig00949.2** | 3 | 2 | 6 | 84 | 91 | 60 | 39.33273617222510 | 4.3518384684410600 | 0.5724047723599640 | 7.602729184976740 | 2.89949851200086E-14 | 8.45240989306096E-12 | 11.07301945033500 | up |
| **LP_3D2.tig14880.2** | 2 | 3 | 1 | 55 | 41 | 18 | 18.83972348464820 | 4.153356136137480 | 0.7737619073343520 | 5.367744388510930 | 7.97274366650563E-08 | 9.32532127001055E-06 | 5.030336197225510 | up |
| **LP_3D3.tig00847.1** | 1 | 5 | 9 | 216 | 158 | 38 | 66.1248787287486 | 4.686955905462250 | 0.6117509665248790 | 7.661542297328980 | 1.83713351818306E-14 | 5.54211731492562E-12 | 11.256324285359000 | up |
| **LP_3D1.tig01278.1** | 3 | 7 | 4 | 120 | 73 | 37 | 38.237001474399300 | 3.9467789828977500 | 0.5777946118609230 | 6.830764603681960 | 8.44632312122993E-12 | 1.81181599179742E-09 | 8.741885911408040 | up |
| **C0_3D2.tig28830.1** | 29 | 39 | 14 | 139 | 57 | 64 | 54.906033024432400 | 1.574738197068900 | 0.4492012470406330 | 3.505640751096250 | 0.0004555095861442740 | 0.022341192773400600 | 1.650893644063850 | up |
| **LP_3D2.tig33833.1** | 0 | 2 | 0 | 27 | 25 | 17 | 11.304215264135500 | 5.042620084388740 | 1.1699421489804200 | 4.310144812530510 | 1.63147674577085E-05 | 0.0012233080988129400 | 2.9124641490446800 | up |
| **LP_3D1.tig08194.1** | 4 | 5 | 10 | 113 | 111 | 68 | 49.521900108511000 | 3.8748371189266100 | 0.4875774867411840 | 7.947120661425150 | 1.90896457304359E-15 | 6.30901692759669E-13 | 12.200038307345600 | up |
| **LP_3D1.tig00981.2** | 1 | 3 | 2 | 72 | 39 | 21 | 21.57109790405070 | 4.367815842907300 | 0.771144769825132 | 5.664067259248570 | 1.47826418923116E-08 | 1.96336279992629E-06 | 5.70699944189384 | up |
| **LP_3D1.tig04172.2** | 0 | 1 | 0 | 27 | 20 | 4 | 8.02861450783046 | 5.456820500179410 | 1.5751728773954900 | 3.4642676867329900 | 0.0005316771501580040 | 0.025591183404514600 | 1.5919096307143300 | up |
| **LP_3D1.tig11416.1** | 0 | 4 | 7 | 29 | 18 | 19 | 12.308360212966300 | 2.5427555267011700 | 0.7310881449517280 | 3.4780423458638600 | 0.0005050901252580170 | 0.024477278917555000 | 1.6112368633555000 | up |
| **C0_3D1.tig11707.3** | 659 | 650 | 706 | 3197 | 2939 | 2147 | 1658.7593927078100 | 1.966371269590840 | 0.23669086284282900 | 8.307761634620360 | 9.75241824949745E-17 | 3.72064574089049E-14 | 13.429381679105200 | up |
| **LP_3D1.tig09939.1** | 5 | 3 | 2 | 35 | 20 | 7 | 11.30729609976390 | 2.5123473572505000 | 0.7729048541307 | 3.2505260431779500 | 0.0011519171748772900 | 0.04949412834913060 | 1.3054463197901700 | up |
| **LP_3D1.tig15904.2** | 42 | 67 | 28 | 20 | 6 | 11 | 28.864017867737500 | -1.9600777656700200 | 0.5217973337736250 | -3.7563966674470900 | 0.00017237739826678000 | 0.009711390688280590 | 2.012718573902540 | down |
| **C0_3D1.tig06409.1** | 98 | 101 | 106 | 764 | 912 | 676 | 428.8029444955930 | 2.8852078207309700 | 0.25976630502343900 | 11.106936369097700 | 1.16073758647252E-28 | 1.73637179218501E-25 | 24.76035727800380 | up |
| **C0_3D1.tig03107.2** | 22 | 35 | 61 | 184 | 123 | 54 | 75.75025917194210 | 1.533471393309480 | 0.43275670406854700 | 3.5434954072175000 | 0.0003948601881904290 | 0.019771396352786300 | 1.703962657601100 | up |
| **LP_3D1.tig00019.4** | 1 | 1 | 0 | 81 | 33 | 15 | 20.249312446087100 | 5.894991086823360 | 1.1634860512208200 | 5.066662450003460 | 4.0485125992186E-07 | 4.29361378176457E-05 | 4.367177023707910 | up |
| **LP_3D1.tig00026.2** | 3 | 6 | 4 | 188 | 99 | 41 | 52.978600707361000 | 4.553171905054890 | 0.599691124156075 | 7.592528422798350 | 3.13722637604127E-14 | 9.05257022061248E-12 | 11.04322809768400 | up |
| **LP_3D1.tig05571.2** | 0 | 0 | 1 | 20 | 17 | 17 | 8.844753754905260 | 5.590370140509340 | 1.522399887929110 | 3.672077346323130 | 0.00024058683823371200 | 0.012950907972912300 | 1.8876997826404700 | up |
| **LP_3D1.tig01177.3** | 4 | 6 | 11 | 130 | 112 | 58 | 50.73732735981810 | 3.761134420676090 | 0.4922480405775460 | 7.640730100750060 | 2.15993412002645E-14 | 6.46218184488967E-12 | 11.189620825160700 | up |
| **LP_3D1.tig22315.1** | 357 | 383 | 528 | 61 | 193 | 164 | 280.1243755900390 | -1.6240207706629200 | 0.3807171444609920 | -4.265688567721730 | 1.99286611222466E-05 | 0.0014598514709975600 | 2.8356913281933200 | down |
| **LP_3D1.tig12216.1** | 1 | 5 | 3 | 57 | 11 | 6 | 12.749818962291600 | 2.9281166129418800 | 0.8601903010237430 | 3.4040335138132000 | 0.0006639860621285150 | 0.030912602540291100 | 1.5098644300286700 | up |
| **C0_3D3.tig19695.2** | 0 | 5 | 2 | 35 | 30 | 12 | 13.18882685023500 | 3.3849212204825400 | 0.8021275851628730 | 4.219928703480800 | 2.44379591687351E-05 | 0.001738643039983420 | 2.759789573649100 | up |
| **LP_3D1.tig05571.1** | 0 | 3 | 0 | 133 | 87 | 88 | 49.67000863882210 | 6.617814616746420 | 0.9099560509800020 | 7.27267499306058 | 3.52437570488408E-13 | 8.86474057274936E-11 | 10.052333969535400 | up |
| **LP_3D1.tig02340.2** | 0 | 2 | 0 | 51 | 26 | 5 | 12.87983061829520 | 5.243538360483140 | 1.2325336541713600 | 4.2542760132650500 | 2.09726475007506E-05 | 0.0015284489066412400 | 2.815749074395950 | up |
| **LP_3D2.tig04626.2** | 0 | 4 | 5 | 23 | 43 | 17 | 14.654666738251500 | 3.154384845241490 | 0.7429151313456200 | 4.245955846299760 | 2.1766349469495E-05 | 0.0015741833786176100 | 2.8029446775151700 | up |
| **LP_3D1.tig04358.2** | 1 | 0 | 1 | 39 | 38 | 15 | 14.783784800844000 | 5.432971277260960 | 1.152705848288990 | 4.713233029332980 | 2.43817098493894E-06 | 0.0002175790104220630 | 3.662383002804510 | up |
| **LP_3D1.tig00626.2** | 0 | 6 | 6 | 107 | 79 | 57 | 40.44166305698710 | 4.278172543989300 | 0.5766573934014350 | 7.418915621205060 | 1.18083107333338E-13 | 3.15137757575756E-11 | 10.501499559617400 | up |
| **LP_3D1.tig01494.3** | 0 | 1 | 2 | 126 | 52 | 32 | 33.10436682846270 | 6.033160193743520 | 0.9645771368001260 | 6.2547202951107 | 3.98228975613647E-10 | 6.64929654778419E-08 | 7.177224297790920 | up |
| **LP_3D1.tig02084.1** | 3 | 2 | 8 | 137 | 63 | 34 | 38.488672179814000 | 4.072821854118930 | 0.6198971457076430 | 6.57015745647547 | 5.02620833625864E-11 | 9.52382709582074E-09 | 8.021188497770790 | up |
| **LP_3D1.tig15206.1** | 0 | 1 | 3 | 41 | 34 | 15 | 14.755743127013000 | 4.414691978755320 | 0.9065452233745440 | 4.869797848939050 | 1.11712476091047E-06 | 0.00010855206330590700 | 3.9643619172684900 | up |
| **LP_3D1.tig11694.1** | 0 | 0 | 1 | 97 | 68 | 32 | 30.975777388440000 | 7.421995870029090 | 1.4612534531881000 | 5.079198173209520 | 3.79031176795248E-07 | 4.05000512122667E-05 | 4.392544427620130 | up |
| **LP_3D1.tig02625.1** | 2 | 0 | 2 | 30 | 11 | 12 | 9.018398581056630 | 3.6356519253864200 | 0.9760523199879680 | 3.7248535257118600 | 0.00019542866199362700 | 0.010754251975825500 | 1.9684197920704900 | up |
| **LP_3D3.tig04725.1** | 5 | 13 | 15 | 197 | 108 | 80 | 65.97641777558950 | 3.4708607137714100 | 0.4548273964305130 | 7.631160174190770 | 2.32650402710962E-14 | 6.88802715734615E-12 | 11.161905149263100 | up |
| **LP_3D2.tig12113.1** | 17 | 14 | 15 | 46 | 43 | 73 | 34.41724089478630 | 1.7800473366678000 | 0.4579283508186610 | 3.887174343946000 | 0.00010141791371095500 | 0.006133086494573660 | 2.2123209104386800 | up |
| **LP_3D2.tig00856.1** | 1 | 1 | 0 | 175 | 110 | 64 | 55.075607222410200 | 7.353179308281710 | 1.091894935489200 | 6.734328614673220 | 1.64688593518812E-11 | 3.34347253520603E-09 | 8.475802239940520 | up |
| **LP_3D3.tig22194.1** | 12 | 10 | 7 | 48 | 112 | 85 | 44.8401855329941 | 3.027726473267950 | 0.48799319204027800 | 6.204444083756900 | 5.48905850948894E-10 | 8.94055962670198E-08 | 7.048635296060690 | up |
| **LP_3D1.tig00406.3** | 0 | 0 | 1 | 240 | 47 | 22 | 47.16120290933520 | 8.02777391894193 | 1.8583961130280700 | 4.319732409395490 | 1.56218510647156E-05 | 0.0011793149054100400 | 2.9283702123595800 | up |
| **LP_3D2.tig06111.2** | 2 | 6 | 5 | 64 | 43 | 16 | 21.260677107455100 | 3.1523539705665400 | 0.6478669533058800 | 4.865742811052450 | 1.14027721521817E-06 | 0.00011061272747282800 | 3.9561948987709200 | up |
| **LP_3D2.tig17674.2** | 0 | 0 | 1 | 15 | 22 | 7 | 7.092456344250290 | 5.270806318082530 | 1.5647457976795900 | 3.368474499755030 | 0.0007558538866797760 | 0.034650414667993400 | 1.4602915637377800 | up |
| **LP_3D1.tig02084.2** | 4 | 7 | 2 | 50 | 43 | 13 | 18.685782964014200 | 2.927223570713220 | 0.6664866400166830 | 4.392021377412680 | 1.1230165398176E-05 | 0.0008756910179140090 | 3.057649104889090 | up |
| **LP_3D2.tig18755.1** | 0 | 0 | 1 | 23 | 21 | 7 | 8.125583981781230 | 5.473043831595460 | 1.5502288821789000 | 3.530474689584480 | 0.0004148147019423810 | 0.0206120120034219 | 1.6858796132991500 | up |
| **LP_3D1.tig00318.1** | 1 | 6 | 3 | 123 | 68 | 32 | 36.31871792929140 | 4.387351410604960 | 0.6518081124350520 | 6.731047568914890 | 1.68445880655459E-11 | 3.40758223696069E-09 | 8.467553654311410 | up |
| **LP_3D3.tig25618.1** | 4 | 9 | 11 | 272 | 215 | 105 | 96.90526812238320 | 4.544271253044600 | 0.4625355289027270 | 9.824696632115950 | 8.81390021083799E-23 | 6.50683321409202E-20 | 19.186630325143800 | up |
| **LP_3D1.tig00271.2** | 0 | 1 | 9 | 101 | 82 | 47 | 37.89504369185530 | 4.482634901962370 | 1.1249505626297600 | 3.9847394640022900 | 6.75542031476798E-05 | 0.0042906353943350300 | 2.367478388976090 | up |
| **LP_3D1.tig00949.3** | 1 | 3 | 2 | 50 | 46 | 31 | 21.187998655226900 | 4.3365841253542 | 0.7377139079733040 | 5.878409066826390 | 4.14228251397314E-09 | 6.03764229504623E-07 | 6.21913262067519 | up |
| **LP_3D1.tig07889.1** | 3 | 3 | 6 | 95 | 49 | 21 | 27.561260976097100 | 3.6804276805183200 | 0.6465192303062090 | 5.6926809103190500 | 1.25059975328497E-08 | 1.6966668967896E-06 | 5.770403413478510 | up |
| **LP_3D1.tig00603.2** | 1 | 1 | 3 | 133 | 87 | 37 | 40.86675525844670 | 5.588016063602490 | 0.7816283549362790 | 7.149198245319610 | 8.72861970144788E-13 | 2.05032391292894E-10 | 9.68817752309782 | up |
| **LP_3D3.tig27245.1** | 3 | 3 | 15 | 120 | 111 | 41 | 46.01828001503880 | 3.6186695561855000 | 0.5580447164375860 | 6.48455123683665 | 8.89962776214194E-11 | 1.62147224403512E-08 | 7.790090481073990 | up |
| **LP_3D1.tig00266.2** | 0 | 0 | 4 | 28 | 14 | 10 | 8.790113826177440 | 3.6295887981578900 | 1.0046702239431200 | 3.6127166025808300 | 0.0003030058106815150 | 0.015802170007514400 | 1.8012832701609000 | up |
| **LP_3D1.tig02572.1** | 11 | 12 | 10 | 122 | 13 | 28 | 30.74293966811870 | 2.190063213473060 | 0.6341749189813600 | 3.453405595085410 | 0.0005535560187273490 | 0.026509597206871200 | 1.5765968709904900 | up |
| **LP_3D1.tig10165.1** | 1 | 5 | 13 | 29 | 32 | 33 | 18.31472370137430 | 2.2840271293845300 | 0.6218763112906070 | 3.672799699741570 | 0.0002399075290581770 | 0.012926581506457000 | 1.8885163110959300 | up |
| **LP_3D1.tig06584.1** | 5 | 18 | 14 | 330 | 276 | 117 | 119.36315608383800 | 4.210013319995430 | 0.443311577994105 | 9.496736672308200 | 2.16570829396358E-21 | 1.43150799965534E-18 | 17.844206220680100 | up |
| **LP_3D1.tig00385.4** | 16 | 29 | 30 | 1460 | 951 | 449 | 459.0252583884280 | 5.164322488770130 | 0.3858132843732050 | 13.38554865253050 | 7.34529549987938E-41 | 2.98245230493317E-37 | 36.52542649279420 | up |
| **LP_3D2.tig00699.3** | 2 | 5 | 5 | 44 | 34 | 23 | 17.967984040550600 | 3.005299890745200 | 0.6306515154231630 | 4.765389152721940 | 1.884894993353E-06 | 0.0001728175095115350 | 3.762412257811970 | up |
| **LP_3D1.tig03476.1** | 0 | 0 | 1 | 24 | 11 | 10 | 7.245086430133620 | 5.302119275540510 | 1.5582314493708000 | 3.402651947296700 | 0.0006673523543559550 | 0.03101851560373200 | 1.508378989260430 | up |
| **LP_3D3.tig30795.1** | 0 | 0 | 2 | 10 | 19 | 8 | 6.217998921885430 | 4.153409528391720 | 1.273699059377240 | 3.2609033490395100 | 0.0011105788843615300 | 0.04815473431085540 | 1.3173610089754700 | up |
| **LP_3D1.tig12537.1** | 0 | 4 | 8 | 186 | 56 | 23 | 42.48932775122190 | 4.381939560631440 | 1.2612038288169100 | 3.4744102900020500 | 0.0005119775286869030 | 0.024789917051283700 | 1.6057249264080600 | up |
| **LP_3D1.tig01278.2** | 3 | 5 | 9 | 146 | 90 | 33 | 44.52589700542560 | 3.8891269739207200 | 0.5756934815663080 | 6.755551519081720 | 1.42292933501589E-11 | 2.92008729418694E-09 | 8.53460416539507 | up |
| **C0_3D2.tig21151.1** | 10 | 21 | 12 | 2 | 2 | 1 | 7.934479958556790 | -3.1629688602442500 | 0.9186619275646910 | -3.443017246430420 | 0.000575262627140763 | 0.02734180939784000 | 1.5631727485284200 | down |
| **C0_3D3.tig14745.1** | 18 | 26 | 41 | 160 | 170 | 105 | 83.297093736223 | 2.2964732576865200 | 0.3637140766276080 | 6.313952099351320 | 2.71998159070784E-10 | 4.62926208155051E-08 | 7.334488231325810 | up |
| **LP_3D1.tig00319.3** | 5 | 3 | 3 | 180 | 84 | 61 | 52.737807577522600 | 4.783339787400120 | 0.5957924862832440 | 8.028533251972040 | 9.86448774061408E-16 | 3.40979378492715E-13 | 12.46727188516480 | up |
| **LP_3D2.tig19085.1** | 1 | 0 | 1 | 20 | 25 | 10 | 9.025028010283730 | 4.697582425612270 | 1.1997995493083800 | 3.9153060428470400 | 9.02896044605548E-05 | 0.005524771329989490 | 2.2576856926978600 | up |
| **LP_3D1.tig23927.1** | 0 | 2 | 1 | 56 | 56 | 18 | 20.813755951416100 | 5.351001617591400 | 0.9766892342839970 | 5.478714651251560 | 4.2842662939686E-08 | 5.20382729659498E-06 | 5.283677125135130 | up |
| **LP_3D1.tig02057.2** | 8 | 13 | 12 | 915 | 714 | 426 | 329.7833099293080 | 5.880400577935330 | 0.3893305129444010 | 15.103878022463400 | 1.52683155292493E-51 | 1.73585479252035E-47 | 46.76048660718070 | up |
| **LP_3D2.tig09179.1** | 0 | 1 | 4 | 39 | 20 | 5 | 10.632630204431200 | 3.583254680146710 | 0.9598595136458740 | 3.733103260638940 | 0.00018913500900432700 | 0.010499394127784200 | 1.9788357613635700 | up |
| **LP_3D3.tig10776.2** | 0 | 1 | 1 | 22 | 19 | 15 | 9.259617331020220 | 4.747036813710670 | 1.187005315993710 | 3.999170643761320 | 6.35648381242867E-05 | 0.004071886626619200 | 2.3902043225473100 | up |
| **LP_3D1.tig00266.3** | 3 | 1 | 1 | 41 | 29 | 16 | 14.375251808054900 | 4.006024529729010 | 0.8368111835928680 | 4.787250228335920 | 1.69081967705262E-06 | 0.0001573105958745100 | 3.803242023881170 | up |
| **LP_3D1.tig00185.5** | 198 | 271 | 474 | 5164 | 3612 | 1786 | 1809.8033387767000 | 3.407975416374020 | 0.3550023377516700 | 9.599867533148320 | 8.00472337265332E-22 | 5.4822710857648E-19 | 18.261039493396900 | up |
| **LP_3D2.tig19262.1** | 1 | 2 | 0 | 42 | 28 | 6 | 12.215126849203300 | 4.548459536974360 | 1.0578834900890300 | 4.299584575794440 | 1.71118564613679E-05 | 0.0012731982729665700 | 2.8951039591506200 | up |
| **LP_3D1.tig04292.2** | 0 | 1 | 3 | 32 | 18 | 7 | 9.476569927408220 | 3.7461054474870100 | 0.990280820398182 | 3.782871858490340 | 0.00015502923629436900 | 0.0088569215448778 | 2.052717202310750 | up |
| **LP_3D1.tig18006.1** | 0 | 1 | 1 | 27 | 18 | 5 | 8.05447517085645 | 4.547696534639150 | 1.2459373328692900 | 3.6500202816510700 | 0.0002622196034623780 | 0.013956810261066400 | 1.8552138255742200 | up |
| **FM1.tig42081.1** | 0 | 5 | 1 | 55 | 41 | 13 | 17.90608210705030 | 4.094705456468700 | 0.8221194510421780 | 4.980669720535090 | 6.33646030195129E-07 | 6.4091830224986E-05 | 4.193197326399040 | up |
| **LP_3D1.tig07541.1** | 0 | 1 | 1 | 29 | 12 | 10 | 8.307862676521390 | 4.59063525139182 | 1.2242147263559900 | 3.7498611579819500 | 0.000176932506086753 | 0.00991886420956753 | 2.0035380552407700 | up |
| **LP_3D1.tig06285.2** | 28 | 62 | 92 | 1087 | 581 | 289 | 334.3608587289610 | 3.346558550752300 | 0.41819713641234400 | 8.002346882290890 | 1.22069919601851E-15 | 4.15512849087857E-13 | 12.38141554179160 | up |
| **LP_3D1.tig00025.9** | 3 | 4 | 5 | 132 | 99 | 42 | 44.66422601049490 | 4.415810826118170 | 0.5855809286838040 | 7.540906149459960 | 4.66716922334973E-14 | 1.3069223374449E-11 | 10.883750219168700 | up |
| **LP_3D1.tig01003.2** | 1 | 2 | 2 | 91 | 69 | 27 | 30.010252490779500 | 5.131738133880720 | 0.7908939637857760 | 6.488528638297620 | 8.66786824476507E-11 | 1.58943538830216E-08 | 7.798757121775110 | up |
| **LP_3D1.tig26322.1** | 0 | 0 | 2 | 26 | 16 | 6 | 7.769151078814610 | 4.49536970745758 | 1.2519219385584000 | 3.590774767182390 | 0.00032969645751194800 | 0.01699147337014200 | 1.7697689608143500 | up |
| **LP_3D2.tig03976.2** | 3 | 7 | 1 | 58 | 56 | 23 | 23.36331399005970 | 3.551081113464270 | 0.6498231456548870 | 5.464688565202650 | 4.63720211805656E-08 | 5.59664022082643E-06 | 5.25207261075129 | up |
| **C0_3D2.tig10681.1** | 44 | 43 | 23 | 11 | 6 | 5 | 22.113631881982700 | -2.4094984988076400 | 0.5583077835332570 | -4.315717190183350 | 1.59085547645275E-05 | 0.0011946126758118400 | 2.9227728813469200 | down |
| **LP_3D1.tig00371.2** | 0 | 1 | 0 | 256 | 48 | 34 | 51.85045781753100 | 8.164879982245990 | 1.7934033525327600 | 4.55272929579117 | 5.29543942865213E-06 | 0.00044995404233442500 | 3.3468318421798300 | up |
| **LP_3D2.tig19172.1** | 1 | 1 | 3 | 38 | 28 | 18 | 14.088336005152400 | 3.9955445874192800 | 0.832653960660785 | 4.798565522042860 | 1.59806030855071E-06 | 0.0001501516334538270 | 3.8234699388820900 | up |
| **LP_3D2.tig00056.3** | 3 | 4 | 5 | 77 | 47 | 23 | 24.93166632529100 | 3.522611145837350 | 0.6261833764375670 | 5.625526448622620 | 1.8494306537006E-08 | 2.41125884196354E-06 | 5.617756166809360 | up |
| **LP_3D1.tig29830.1** | 0 | 2 | 0 | 62 | 25 | 15 | 16.151228053520900 | 5.574135993072680 | 1.174314348288850 | 4.7467153928545600 | 2.06746542428173E-06 | 0.00018714183446384600 | 3.7278291176527300 | up |
| **LP_3D1.tig01150.4** | 40 | 81 | 88 | 942 | 829 | 362 | 369.3573618745480 | 3.2766678211852500 | 0.3543288632191110 | 9.247532903236880 | 2.29733861321761E-20 | 1.36033555696203E-17 | 16.866353950040300 | up |
| **C0_3D1.tig07866.1** | 40 | 18 | 22 | 2 | 1 | 0 | 14.149120652246900 | -4.838350146017250 | 1.00806495938231 | -4.799641234412060 | 1.58950110826491E-06 | 0.00014959468625715100 | 3.8250838327428400 | down |
| **LP_3D1.tig00019.8** | 2 | 8 | 4 | 87 | 54 | 19 | 27.10744647157340 | 3.4201976390744400 | 0.6361757606805770 | 5.376183517925180 | 7.60812471019929E-08 | 8.93561671800162E-06 | 5.048875468006460 | up |
| **C0_3D1.tig10768.1** | 190 | 199 | 209 | 18 | 60 | 46 | 120.53425476491800 | -2.307849522090270 | 0.3850936184128590 | -5.992957067431900 | 2.06059265725502E-09 | 3.11527631919313E-07 | 6.506503426186640 | down |
| **LP_3D1.tig04491.1** | 2 | 5 | 5 | 150 | 76 | 40 | 43.360330869911200 | 4.37673298649243 | 0.606592605195169 | 7.215275868858030 | 5.38247457643463E-13 | 1.30755028759584E-10 | 9.883541599435520 | up |
| **LP_3D1.tig03233.3** | 2 | 0 | 1 | 26 | 28 | 12 | 10.927117268839900 | 4.3670338277367000 | 1.019611247777210 | 4.283038106196830 | 1.84358582843845E-05 | 0.0013592559846638600 | 2.866698746168250 | up |
| **LP_3D1.tig11347.1** | 6 | 8 | 11 | 261 | 226 | 97 | 95.75919437147090 | 4.459648115087150 | 0.4597289949842590 | 9.70060223249536 | 2.99724230047839E-22 | 2.10343504408265E-19 | 18.677070894834100 | up |
| **C0_3D2.tig31832.1** | 8 | 13 | 10 | 33 | 57 | 21 | 22.76464396978910 | 1.7697504158571000 | 0.5306041530875490 | 3.335349724571590 | 0.0008519218502916890 | 0.038282606782475200 | 1.4169984974182100 | up |
| **LP_3D3.tig22721.1** | 0 | 3 | 1 | 13 | 16 | 14 | 7.596729959718770 | 3.3864373941927100 | 0.9872504871875720 | 3.430170395600230 | 0.0006032023218661240 | 0.028526652235008200 | 1.5447491922816500 | up |
| **LP_3D1.tig00673.1** | 0 | 2 | 2 | 119 | 68 | 25 | 33.47823766070690 | 5.629404942464760 | 0.8737439978693810 | 6.442853920818950 | 1.17247535235971E-10 | 2.08279254390274E-08 | 7.681353985705240 | up |
| **LP_3D3.tig00600.1** | 16 | 9 | 10 | 38 | 39 | 47 | 26.1475970331585 | 1.7666466138300100 | 0.48822906739658200 | 3.6184789718695400 | 0.0002963395727365710 | 0.015497169284462200 | 1.809747622888350 | up |
| **LP_3D1.tig00341.4** | 1 | 9 | 8 | 407 | 207 | 77 | 109.68819064588800 | 5.179318127278200 | 0.8929397627805910 | 5.800299575807820 | 6.61965510073761E-09 | 9.29121714077603E-07 | 6.031927390107720 | up |
| **LP_3D1.tig15510.2** | 0 | 0 | 1 | 21 | 25 | 12 | 9.350916261525660 | 5.676097239363610 | 1.5228399531978500 | 3.7273104290731500 | 0.00019353402837938700 | 0.010660311863591300 | 1.9722300899946600 | up |
| **LP_3D2.tig19961.1** | 0 | 1 | 1 | 18 | 23 | 6 | 7.678570400948040 | 4.47327713862674 | 1.240137329502910 | 3.6070820805142400 | 0.00030965972660721700 | 0.016090134514613600 | 1.7934403251545300 | up |
| **LP_3D1.tig02921.1** | 0 | 4 | 5 | 98 | 46 | 31 | 28.78814266035320 | 4.202540280401020 | 0.6816823869225430 | 6.164953592791810 | 7.05038143194052E-10 | 1.12578351825466E-07 | 6.948545113806440 | up |
| **LP_3D3.tig32193.1** | 5 | 2 | 4 | 84 | 36 | 13 | 22.33470068121240 | 3.470593395415490 | 0.7063622087577160 | 4.913333913374620 | 8.9540586734706E-07 | 8.77574940161097E-05 | 4.056715786874940 | up |
| **LP_3D2.tig03656.1** | 6 | 16 | 17 | 337 | 244 | 184 | 127.7337273634850 | 4.229479175998930 | 0.39233926791659900 | 10.780157689691200 | 4.27152874840575E-27 | 5.27858808050271E-24 | 23.277482227247900 | up |
| **LP_3D1.tig01572.2** | 1 | 1 | 1 | 70 | 48 | 50 | 27.39593358258920 | 5.742925036673580 | 0.9284344037061130 | 6.185601280767970 | 6.18661987792505E-10 | 9.96256110370112E-08 | 7.001629001915150 | up |
| **LP_3D1.tig23314.1** | 5 | 15 | 5 | 378 | 281 | 126 | 126.97076768141300 | 4.883068214132690 | 0.47782804196210600 | 10.219300219554600 | 1.62509770300936E-24 | 1.46632823694551E-21 | 20.833768802179200 | up |
| **C0_3D1.tig14211.1** | 60 | 119 | 144 | 40 | 28 | 18 | 66.81306330913710 | -1.9680145691367100 | 0.399366550020638 | -4.9278402736408700 | 8.31435418399521E-07 | 8.191151881962E-05 | 4.08665502121834 | down |
| **LP_3D1.tig04903.1** | 0 | 2 | 2 | 47 | 21 | 10 | 12.717587457671900 | 4.192684345689030 | 0.950804052228146 | 4.409619769566350 | 1.03552275181737E-05 | 0.000812439146653371 | 3.0902091586720300 | up |
| **LP_3D2.tig17643.1** | 5 | 2 | 7 | 40 | 44 | 14 | 17.703486138582500 | 2.7151544243492300 | 0.6509381507645220 | 4.17114040890107 | 3.03079033056591E-05 | 0.0021087549123747800 | 2.6759738927064200 | up |
| **LP_3D1.tig00371.3** | 1 | 1 | 1 | 252 | 48 | 41 | 52.851292689560500 | 6.7058268709460700 | 0.99386056515546 | 6.747251179945090 | 1.50672312729884E-11 | 3.08092360328427E-09 | 8.51131907061634 | up |
| **LP_3D1.tig05453.1** | 9 | 19 | 26 | 299 | 237 | 136 | 114.85462945394300 | 3.5678428077656400 | 0.3938192361397170 | 9.05959506381212 | 1.30935728734155E-19 | 7.02173726405006E-17 | 16.153555424786400 | up |
| **LP_3D1.tig18680.1** | 18 | 35 | 54 | 14 | 5 | 4 | 21.172265903638700 | -2.2802932284651900 | 0.5914888041172790 | -3.8551756391538800 | 0.00011564657071942000 | 0.006876495096804840 | 2.1626328623413400 | down |
| **LP_3D1.tig00339.2** | 26 | 53 | 75 | 256 | 187 | 79 | 106.85622510942800 | 1.6848958068283400 | 0.41370393982548200 | 4.07270911545852 | 4.64694461321155E-05 | 0.003068008902880500 | 2.5131433844667800 | up |
| **LP_3D1.tig06703.1** | 2 | 7 | 2 | 111 | 62 | 24 | 32.34390871320890 | 4.061840502073740 | 0.6598036852142380 | 6.156134912697340 | 7.45417298140048E-10 | 1.1869256670244E-07 | 6.925576478528120 | up |
| **LP_3D2.tig00576.3** | 0 | 6 | 0 | 50 | 43 | 34 | 21.2416446687271 | 4.34565612952786 | 0.7666644371120920 | 5.668263609431630 | 1.44251913227014E-08 | 1.92202471013371E-06 | 5.716241033209760 | up |
| **LP_3D1.tig32638.1** | 0 | 0 | 1 | 23 | 16 | 7 | 7.342158406599670 | 5.323933275284210 | 1.5600561109501000 | 3.4126549922886100 | 0.0006433333196084600 | 0.030098997986125800 | 1.5214479620918600 | up |
| **LP_3D1.tig00871.2** | 1 | 1 | 1 | 33 | 15 | 17 | 10.808543754676700 | 4.361249039857480 | 1.0181897061332400 | 4.283336409302480 | 1.84111478017733E-05 | 0.0013591970088205300 | 2.8667175898881100 | up |
| **LP_3D1.tig00626.3** | 3 | 8 | 11 | 97 | 77 | 46 | 38.33852268669730 | 3.253731936062250 | 0.5030795572439160 | 6.467629004620210 | 9.95525430112219E-11 | 1.80224977944997E-08 | 7.744185018951110 | up |
| **LP_3D1.tig01311.3** | 1 | 12 | 4 | 433 | 237 | 86 | 119.71580347354400 | 5.390061211209570 | 0.9606552766624670 | 5.610817264165620 | 2.01373319528182E-08 | 2.61348546771221E-06 | 5.582779910454290 | up |
| **LP_3D1.tig00123.5** | 5 | 15 | 7 | 57 | 35 | 28 | 23.44255431709590 | 2.08323711129778 | 0.5363860512285460 | 3.8838390866546700 | 0.00010281996878204500 | 0.006204672107659630 | 2.2072811643017100 | up |
| **LP_3D1.tig01334.1** | 5 | 4 | 10 | 289 | 211 | 111 | 99.09107742012290 | 4.92044865510672 | 0.4865001394600430 | 10.11397172582070 | 4.79020534347853E-24 | 4.18921881153903E-21 | 20.377866954949800 | up |
| **LP_3D1.tig02458.1** | 2 | 0 | 1 | 18 | 18 | 9 | 7.632826361264910 | 3.815428885370290 | 1.0678625408317000 | 3.5729588214590400 | 0.0003529702836811590 | 0.018027489466177400 | 1.7440647495339100 | up |
| **LP_3D1.tig01332.3** | 1 | 1 | 0 | 532 | 324 | 130 | 153.52941002567600 | 8.836479617144390 | 1.0950867579239600 | 8.069205068186940 | 7.07573359449855E-16 | 2.49826134272839E-13 | 12.60236213210760 | up |
| **LP_3D1.tig20632.2** | 0 | 0 | 1 | 24 | 5 | 24 | 8.813901761590540 | 5.581995371392450 | 1.5663027509339900 | 3.5638035929285600 | 0.0003655194950429930 | 0.018502186728155800 | 1.7327769403619100 | up |
| **LP_3D1.tig24178.1** | 0 | 2 | 0 | 65 | 20 | 8 | 14.55951924971470 | 5.423861933826150 | 1.2211051640820600 | 4.441764799105920 | 8.92240579358626E-06 | 0.0007083717281234790 | 3.1497387802953200 | up |
| **C0_3D1.tig05112.1** | 39 | 70 | 70 | 23 | 23 | 13 | 38.99678749899610 | -1.6597702250221100 | 0.4207050292716870 | -3.9452112752145200 | 7.97296487589561E-05 | 0.0049695525040601500 | 2.3036827166524300 | down |
| **LP_3D1.tig13197.2** | 0 | 3 | 1 | 67 | 33 | 22 | 19.72528500755070 | 4.846064748123810 | 0.8874255287710350 | 5.460812869373910 | 4.73959425871641E-08 | 5.70809821264268E-06 | 5.243508563090360 | up |
| **C0_90D2.tig00001.1** | 290 | 463 | 328 | 120 | 49 | 55 | 215.65090629891000 | -2.345810573504900 | 0.34421377989671700 | -6.814981591407440 | 9.42758369354602E-12 | 1.98485553725787E-09 | 8.702271096787450 | down |
| **LP_3D1.tig34529.1** | 38 | 72 | 90 | 645 | 619 | 321 | 283.42952785666500 | 2.921335977309350 | 0.33643842171393100 | 8.68312234502552 | 3.85049826528275E-18 | 1.72347695976376E-15 | 14.763594518066500 | up |
| **C0_3D1.tig04125.4** | 92 | 283 | 297 | 98 | 67 | 54 | 144.92814018772200 | -1.6640054063062600 | 0.3879417188882840 | -4.289318021982180 | 1.79222591217853E-05 | 0.0013285906196872300 | 2.876608818154030 | down |
| **LP_3D1.tig03817.3** | 0 | 3 | 0 | 40 | 27 | 8 | 12.100608364193100 | 4.54722074018888 | 1.0552844709556000 | 4.308999957207010 | 1.63994399996359E-05 | 0.0012250015332185300 | 2.9118633677337900 | up |

Note- Genes were identified using differential expression analysis with thresholds of |log₂ fold change| ≥ 1.5 and adjusted *P* < 0.05. Positive log₂ fold-change values indicate genes upregulated in LP-inoculated silage relative to the control, while negative values indicate downregulation.
