# Supplementary material for: Metatranscriptomic insights into the effect of Lactiplantibacillus plantarum inoculation on fermentation metabolism, bacterial community function and antibiotic resistome of corn silage
Source: Front Microbiol. 2026 Sep 9;17:1876540. doi: 10.3389/fmicb.2026.1876540 (PMC13597671; doi:10.3389/fmicb.2026.1876540)
Supplement: Supplementary file 3 [file Table_3.docx]

Table S3. Differentially expressed genes between LP-inoculated and control silage at day 90

| **Gene** | **C0_90D1** | **C0_90D2** | **C0_90D3** | **LP_90D1** | **LP_90D2** | **LP_90D3** | **baseMean** | **log2FoldChange** | **lfcSE** | **stat** | **pvalue** | **padj** | **-log10padj** | **type** |
| --- | --- | --- | --- | --- | --- | --- | --- | --- | --- | --- | --- | --- | --- | --- |
| **LP_90D1.tig00136.2** | 0 | 3 | 27 | 2233 | 736 | 1053 | 1414.5366232715200 | 8.63115354573831 | 1.6215959158507300 | 5.322629060279900 | 1.02278170692563E-07 | 9.1309247092353E-06 | 5.039485238261730 | up |
| **C0_90D1.tig19040.1** | 20 | 21 | 25 | 0 | 5 | 5 | 9.63373552232736 | -2.2619781923033500 | 0.6581047442890580 | -3.4371096879827800 | 0.000587957446363796 | 0.028869575259765900 | 1.5395596055788200 | down |
| **C0_90D3.tig00604.1** | 1 | 5 | 104 | 1205 | 1021 | 695 | 872.6184613231650 | 6.046921819230320 | 1.5298400124499300 | 3.952649799992230 | 7.7290512648307E-05 | 0.0043963422944025900 | 2.356908501238770 | up |
| **C0_90D1.tig00064.4** | 75 | 25 | 285 | 18158 | 6240 | 7890 | 11457.85776090460 | 7.926593786278620 | 1.251361031211750 | 6.334378000090800 | 2.38300639811531E-10 | 4.57277015095817E-08 | 7.339820627764140 | up |
| **C0_90D3.tig27834.1** | 122 | 18 | 199 | 13462 | 6980 | 3496 | 8470.633531816150 | 7.636355940172050 | 1.2493604014430400 | 6.112212241841430 | 9.82593806133294E-10 | 1.42025214688245E-07 | 6.847634545561710 | up |
| **LP_90D1.tig00135.10** | 0 | 0 | 3 | 241 | 106 | 86 | 152.12551139829900 | 8.704354772938680 | 1.6448733616308800 | 5.291808461356780 | 1.21112701648576E-07 | 1.04762486926018E-05 | 4.979794200534830 | up |
| **C0_90D3.tig00197.3** | 0 | 0 | 18 | 614 | 352 | 214 | 398.6020351960820 | 7.54241431958722 | 1.920262474513010 | 3.92780383916008 | 8.57250849308849E-05 | 0.00481864454672949 | 2.3170751087864700 | up |
| **LP_90D1.tig00008.39** | 55 | 5 | 78 | 9294 | 2298 | 3162 | 5618.259353505820 | 8.33591853796195 | 1.347499967166480 | 6.186210568517290 | 6.1627676060079E-10 | 9.75206860649279E-08 | 7.010903252086510 | up |
| **C0_90D3.tig01308.1** | 0 | 0 | 4 | 129 | 81 | 80 | 89.65343803571000 | 7.523256325892360 | 1.5105455308437100 | 4.980489612709830 | 6.34236068837606E-07 | 4.86816278216525E-05 | 4.31263490821228 | up |
| **LP_90D2.tig20064.1** | 0 | 0 | 3 | 1596 | 584 | 940 | 1042.9010276304400 | 11.48607867440000 | 1.60089905956332 | 7.1747675818693100 | 7.24301269759085E-13 | 6.36413028841739E-10 | 9.19626093792528 | up |
| **LP_90D1.tig01461.2** | 11 | 7 | 5 | 17 | 25 | 31 | 19.46295786827770 | 2.421161244722970 | 0.687965192142881 | 3.519307767855970 | 0.0004326744628991490 | 0.02185562426889510 | 1.6604367841107600 | up |
| **C0_90D3.tig08581.2** | 9 | 0 | 80 | 2168 | 1235 | 669 | 1397.2919310860200 | 7.024464069220270 | 1.730504809097350 | 4.059199392161350 | 4.92412538464253E-05 | 0.002864313980275780 | 2.542979377276480 | up |
| **LP_90D1.tig01126.1** | 9 | 1 | 29 | 3808 | 1098 | 1516 | 2351.0422285117900 | 8.940427666164870 | 1.3848816765272000 | 6.455733957419610 | 1.07695660277939E-10 | 2.40937386050985E-08 | 7.61809580566429 | up |
| **LP_90D2.tig17948.2** | 0 | 0 | 1 | 129 | 28 | 48 | 77.94099991147490 | 9.159725038507130 | 1.9775928644153100 | 4.631754696998910 | 3.62579594866574E-06 | 0.00025168752792099900 | 3.5991383048699600 | up |
| **C0_90D3.tig06141.1** | 0 | 9 | 96 | 3301 | 2135 | 1777 | 2264.1635311928100 | 7.503000206864230 | 1.7027103776164 | 4.406504068746890 | 1.05052335983643E-05 | 0.0006758366948281050 | 3.170158231725500 | up |
| **LP_90D2.tig06001.1** | 0 | 0 | 1 | 164 | 45 | 53 | 99.12544998669220 | 9.508631697944840 | 1.964150477805710 | 4.841091253134330 | 1.29128054771297E-06 | 9.4136607440149E-05 | 4.026241457150850 | up |
| **LP_90D3.tig01731.1** | 0 | 0 | 4 | 39 | 43 | 148 | 47.397701744665900 | 6.587794435524780 | 1.2807986056710700 | 5.143505314852460 | 2.69659021806992E-07 | 2.19601099490577E-05 | 4.658365489807120 | up |
| **LP_90D1.tig00070.3** | 0 | 0 | 2 | 164 | 78 | 70 | 105.90069591751200 | 8.750528157160220 | 1.6492002746597800 | 5.305922083335440 | 1.12104839435074E-07 | 9.84343357692372E-06 | 5.006853384962530 | up |
| **C0_90D2.tig22470.1** | 7 | 5 | 1 | 330 | 116 | 158 | 211.4855814644560 | 6.956558002630240 | 1.2340019358848200 | 5.637396344635540 | 1.72640456863183E-08 | 1.8299340362555E-06 | 5.737564565027260 | up |
| **C0_90D1.tig17269.1** | 20 | 18 | 4 | 307 | 28 | 87 | 181.81252756267100 | 5.016867741438380 | 1.3995370652871700 | 3.5846622900330000 | 0.0003375146423046870 | 0.017417737854576800 | 1.7590082500786000 | up |
| **LP_90D3.tig15278.1** | 0 | 0 | 2 | 136 | 15 | 29 | 77.34618925518930 | 8.307381382181940 | 1.944416459209760 | 4.272429058514650 | 1.93355077647335E-05 | 0.0011845748050581400 | 2.9264375086869600 | up |
| **C0_90D2.tig09689.1** | 33 | 61 | 124 | 3 | 3 | 7 | 29.600156653891800 | -3.3520364818544000 | 0.819492655520764 | -4.090380138580100 | 4.30666735321791E-05 | 0.0025316076805738100 | 2.596603595401600 | down |
| **LP_90D1.tig00128.6** | 0 | 0 | 1 | 222 | 104 | 80 | 140.85304634105700 | 10.017517795244000 | 1.886145548868700 | 5.311105392291960 | 1.08962290520926E-07 | 9.62471406932063E-06 | 5.016612163633570 | up |
| **C0_90D1.tig22040.1** | 6 | 0 | 14 | 159 | 25 | 109 | 104.24599042000800 | 5.358406518438220 | 1.5362374748246300 | 3.4880066436668000 | 0.0004866359869210500 | 0.024215035718788300 | 1.615914886003720 | up |
| **C0_90D3.tig00213.4** | 9 | 0 | 3 | 239 | 318 | 68 | 177.0179153675320 | 6.797645511572000 | 1.386387106983140 | 4.903136704988600 | 9.4318296120009E-07 | 7.06097217298426E-05 | 4.151135500039450 | up |
| **C0_90D3.tig07651.1** | 16 | 22 | 67 | 0 | 2 | 2 | 13.335147577985700 | -4.22316279812765 | 1.0938623162832600 | -3.8607809550265500 | 0.00011302519505560600 | 0.00624801032733712 | 2.2042582612847500 | down |
| **C0_90D1.tig00064.13** | 101 | 28 | 267 | 17930 | 6159 | 8220 | 11376.995943967400 | 7.861234552457190 | 1.2101461528918500 | 6.496103411700680 | 8.24270091889285E-11 | 2.03818998284558E-08 | 7.690755337181410 | up |
| **LP_90D1.tig00180.6** | 1 | 0 | 4 | 109 | 66 | 46 | 72.58244731398510 | 6.860980249194960 | 1.379353675775180 | 4.974054421060030 | 6.55668890793038E-07 | 5.02113041162586E-05 | 4.2991984987383800 | up |
| **LP_3D1.tig00951.3** | 0 | 0 | 5 | 137 | 104 | 50 | 92.64784949444210 | 7.257186582919600 | 1.6005829536112700 | 4.534089636869980 | 5.78524585362082E-06 | 0.00038865910222645000 | 3.410431156852280 | up |
| **C0_90D3.tig01176.1** | 26 | 8 | 43 | 2532 | 1179 | 783 | 1595.4529070880600 | 7.365402730767110 | 1.1597557647970300 | 6.350822263044420 | 2.14167044109578E-10 | 4.15745548591553E-08 | 7.381172392096360 | up |
| **LP_90D2.tig04988.1** | 0 | 0 | 5 | 109 | 89 | 184 | 95.11984194806500 | 7.2877773193815300 | 1.3681142427657200 | 5.326877750098460 | 9.99153620294779E-08 | 8.96794092151139E-06 | 5.047307261424020 | up |
| **LP_90D3.tig03920.2** | 0 | 4 | 3 | 608 | 1171 | 210 | 498.9393794416080 | 9.178880682660310 | 1.230399334653300 | 7.460082612322560 | 8.64682476062645E-14 | 1.31231287242071E-10 | 9.881962611174580 | up |
| **C0_3D2.tig29292.1** | 202 | 237 | 525 | 8 | 85 | 64 | 142.95420879145200 | -2.258227349492240 | 0.6013542278731190 | -3.7552365059096400 | 0.00017317786908377400 | 0.009403897157920150 | 2.02669212901456 | down |
| **LP_90D3.tig01224.3** | 0 | 1 | 1 | 70 | 36 | 182 | 67.29581574598940 | 8.068834859489070 | 1.4076318579743100 | 5.7322053445854300 | 9.91331523172565E-09 | 1.10700897080966E-06 | 5.955848859737950 | up |
| **LP_90D2.tig25774.1** | 0 | 1 | 0 | 63 | 54 | 7 | 40.9805238796464 | 8.224810982433970 | 1.9841128621129200 | 4.1453342395428 | 3.39318391271119E-05 | 0.002026744501994880 | 2.69320099643158 | up |
| **C0_90D1.tig16573.1** | 17 | 3 | 5 | 3248 | 1482 | 809 | 2005.9140168310600 | 9.255426753776410 | 1.2653532138949300 | 7.314500530082790 | 2.58340488454438E-13 | 3.19471502555749E-10 | 9.495567875642560 | up |
| **LP_90D1.tig00208.3** | 0 | 3 | 0 | 38 | 56 | 7 | 28.421993395478500 | 6.2136998367683500 | 1.6366823023900000 | 3.7965216754006900 | 0.00014674053771850500 | 0.008031999694890420 | 2.095176316696490 | up |
| **C0_3D2.tig03844.1** | 3 | 0 | 0 | 131 | 22 | 43 | 77.79830848540500 | 7.590721867523100 | 1.8197429664954900 | 4.171315404032860 | 3.0284633631407E-05 | 0.0018186576120846200 | 2.740249055295290 | up |
| **LP_90D1.tig00079.8** | 187 | 11 | 280 | 35832 | 6245 | 10573 | 21087.723249250200 | 8.454783799772710 | 1.4506010083172300 | 5.828469545585590 | 5.59380024170347E-09 | 6.55338232527148E-07 | 6.183534494501950 | up |
| **LP_90D1.tig00070.11** | 5 | 0 | 4 | 549 | 232 | 139 | 337.59568630272200 | 8.189776036826540 | 1.4481612125094200 | 5.655293047543390 | 1.55580591159398E-08 | 1.65964228697161E-06 | 5.779985508065490 | up |
| **LP_90D1.tig00113.7** | 1 | 1 | 1 | 1173 | 658 | 223 | 729.5808072962350 | 10.913800265420500 | 1.4497385088975200 | 7.528116414400910 | 5.14774878429406E-14 | 9.04622021888392E-11 | 10.043532844107000 | up |
| **LP_90D3.tig00014.34** | 0 | 0 | 11 | 228 | 186 | 92 | 157.41204459078100 | 6.901096167654990 | 1.7489891901625300 | 3.945762619043800 | 7.95463918376177E-05 | 0.004501651656044430 | 2.3466281143356800 | up |
| **LP_90D2.tig25809.2** | 0 | 25 | 95 | 2651 | 1125 | 1597 | 1770.9503725111500 | 6.943126675995720 | 1.6539644589675100 | 4.197869330475200 | 2.69438014619283E-05 | 0.0016268111880873800 | 2.7886628494787200 | up |
| **C0_90D1.tig01862.3** | 14 | 1 | 34 | 3036 | 1130 | 1378 | 1933.4407959879700 | 8.31603454278182 | 1.3509052006362600 | 6.155897940777100 | 7.46532897521812E-10 | 1.13299940524344E-07 | 6.94577031811503 | up |
| **LP_90D3.tig05606.1** | 0 | 0 | 3 | 1003 | 709 | 387 | 670.5756022184220 | 10.846782599074400 | 1.5630638618316000 | 6.93943661800493 | 3.93666691158698E-12 | 2.19068952518296E-09 | 8.659419168333530 | up |
| **C0_90D3.tig00964.1** | 28 | 8 | 57 | 5631 | 1711 | 2259 | 3494.7178325001600 | 8.237864738871820 | 1.2095287414129800 | 6.810805280450210 | 9.70539235096288E-12 | 4.69642529284492E-09 | 8.328232581698160 | up |
| **LP_90D1.tig01533.3** | 0 | 0 | 3 | 40 | 34 | 22 | 28.750150346881500 | 6.25991562913438 | 1.4726160278841900 | 4.250881092288830 | 2.12931197606969E-05 | 0.0012973649191421700 | 2.8869378496067800 | up |
| **C0_90D3.tig00839.1** | 15 | 21 | 179 | 7216 | 3994 | 3616 | 4823.374840624130 | 7.543255075816990 | 1.2511474837001200 | 6.02906945351375 | 1.6490641315062E-09 | 2.21145697762347E-07 | 6.65532150511304 | up |
| **C0_90D1.tig12151.2** | 10 | 7 | 31 | 1702 | 611 | 748 | 1080.0212628817300 | 7.509387022177810 | 1.1486468270549500 | 6.5375943634749100 | 6.2516201619032E-11 | 1.64358539831328E-08 | 7.78420772568477 | up |
| **C0_90D3.tig00120.7** | 7 | 4 | 33 | 1633 | 656 | 782 | 1054.0345743874900 | 7.614848784598630 | 1.2126770568700800 | 6.2793707042273700 | 3.39946165252499E-10 | 5.97392763769247E-08 | 7.223740042317960 | up |
| **LP_90D1.tig07958.2** | 1 | 0 | 0 | 593 | 368 | 186 | 383.73040343275300 | 11.467278536252500 | 1.8726292998791200 | 6.123624433833610 | 9.1470358383726E-10 | 1.35137336109479E-07 | 6.8692246463430900 | up |
| **LP_90D1.tig00065.4** | 13 | 10 | 86 | 3783 | 2092 | 1664 | 2495.1740918274800 | 7.560382136003780 | 1.207829278719010 | 6.25947910786043 | 3.86265475455809E-10 | 6.58011120407858E-08 | 7.181766766734130 | up |
| **LP_90D1.tig00264.1** | 0 | 2 | 0 | 415 | 433 | 150 | 294.07601300926100 | 10.196901954819100 | 1.576421960242210 | 6.4683836003226 | 9.90567766345294E-11 | 2.33272688931139E-08 | 7.632136104501610 | up |
| **C0_90D3.tig00802.1** | 8 | 1 | 24 | 839 | 552 | 296 | 555.4594766626540 | 7.087779520371410 | 1.2856457632698100 | 5.513011221959710 | 3.52745731046069E-08 | 3.42378698078407E-06 | 5.465493263839490 | up |
| **LP_90D1.tig00049.13** | 1 | 0 | 0 | 115 | 62 | 49 | 75.15905327740700 | 9.105123439038000 | 1.8479012515394000 | 4.927278138619670 | 8.33830186597062E-07 | 6.27044056312822E-05 | 4.202701944430250 | up |
| **LP_90D2.tig02881.4** | 5 | 3 | 7 | 471 | 167 | 222 | 300.99282294028800 | 7.309964140325180 | 1.1317980742906100 | 6.458717598461110 | 1.05593949674454E-10 | 2.39841930998662E-08 | 7.6200748879202100 | up |
| **LP_90D1.tig00049.16** | 3 | 0 | 8 | 387 | 171 | 161 | 248.42347004784100 | 7.505629094025920 | 1.4022839222957200 | 5.35243182545959 | 8.67800022063075E-08 | 7.87363449365869E-06 | 5.103824749702190 | up |
| **C0_90D1.tig03193.1** | 223 | 47 | 323 | 22828 | 6822 | 9471 | 14224.921063535300 | 7.571728503464490 | 1.202025689746750 | 6.2991403328990100 | 2.99300929813466E-10 | 5.37277351910851E-08 | 7.2698014660522800 | up |
| **LP_90D1.tig02322.2** | 0 | 0 | 4 | 37 | 43 | 148 | 46.352649553479200 | 6.555373693665530 | 1.274667236945290 | 5.1428117893539900 | 2.70656736918598E-07 | 2.19659402653994E-05 | 4.65825020176199 | up |
| **LP_90D1.tig00052.12** | 0 | 0 | 3 | 312 | 153 | 105 | 197.96674664417200 | 9.08577321037111 | 1.638039564891650 | 5.546736113771540 | 2.91051500998683E-08 | 2.94482380813486E-06 | 5.530940684389220 | up |
| **LP_90D1.tig00092.5** | 5 | 1 | 6 | 254 | 94 | 123 | 163.79252595443600 | 6.7394916074513800 | 1.209607769419900 | 5.571633861680230 | 2.52361350778158E-08 | 2.56893083571095E-06 | 5.5902475882495900 | up |
| **LP_90D1.tig01533.1** | 0 | 1 | 5 | 60 | 35 | 17 | 38.97800243219140 | 5.706887342307720 | 1.452200145774050 | 3.9298214911456500 | 8.5008947832437E-05 | 0.004786448160501250 | 2.319986640340110 | up |
| **C0_90D1.tig04097.1** | 41 | 7 | 90 | 6325 | 1959 | 2665 | 3952.4411744726100 | 7.848220533600780 | 1.2683880595422800 | 6.187554727086380 | 6.1104636424695E-10 | 9.71534621706735E-08 | 7.0125417182260400 | up |
| **LP_90D1.tig03173.1** | 2 | 14 | 1 | 95 | 27 | 58 | 63.47328717240130 | 4.840032723734780 | 1.349143935106880 | 3.587484328239110 | 0.00033388379381546000 | 0.017257037138861300 | 1.7630337662510700 | up |
| **LP_3D3.tig17949.1** | 13 | 27 | 32 | 18 | 106 | 86 | 44.13548478822920 | 2.0015057744699000 | 0.45990802475038200 | 4.351969669492570 | 1.34919930218642E-05 | 0.0008448624667976910 | 3.073213983088440 | up |
| **LP_90D1.tig00725.1** | 1 | 1 | 0 | 211 | 257 | 31 | 147.96246532204600 | 9.171529642757810 | 1.6039465414078000 | 5.718101823211550 | 1.07720589809533E-08 | 1.19491122031578E-06 | 5.922664360790540 | up |
| **C0_90D3.tig00250.2** | 24 | 5 | 85 | 4900 | 1244 | 1694 | 2974.8307693845000 | 7.733306922839640 | 1.3560543395173900 | 5.702800173621270 | 1.17855115575864E-08 | 1.29870113992163E-06 | 5.886490778269480 | up |
| **LP_90D1.tig00049.3** | 37 | 12 | 49 | 3891 | 1361 | 1734 | 2466.743562593230 | 7.637881305819230 | 1.1226085278384700 | 6.803690793731640 | 1.01972284599458E-11 | 4.72882307012682E-09 | 8.325246934896620 | up |
| **LP_90D1.tig06711.1** | 0 | 0 | 1 | 47 | 11 | 20 | 28.931228606614400 | 7.711967587167120 | 1.9531490497088000 | 3.94847878522989 | 7.8649384390635E-05 | 0.0044660277132974700 | 2.3500785863038100 | up |
| **LP_90D2.tig02621.2** | 1 | 5 | 39 | 1456 | 579 | 716 | 942.2791343132670 | 7.449987911099940 | 1.4108379498703900 | 5.280541193114600 | 1.28802860783926E-07 | 1.10555236984949E-05 | 4.956420680133700 | up |
| **LP_90D3.tig15024.1** | 16 | 14 | 14 | 7 | 89 | 50 | 27.775513366676600 | 2.047716496027380 | 0.5826405062319180 | 3.5145453742488300 | 0.00044050740994041800 | 0.022117446481955800 | 1.6552650149266 | up |
| **LP_90D1.tig00161.10** | 6 | 0 | 2 | 379 | 99 | 138 | 231.42471676730300 | 7.778738714067280 | 1.5100601585568900 | 5.151277364672100 | 2.58718226268424E-07 | 2.11724089629324E-05 | 4.674229725835620 | up |
| **LP_90D1.tig00198.2** | 2 | 0 | 18 | 750 | 289 | 157 | 453.7436989292050 | 7.5541203295359800 | 1.6576792251055100 | 4.5570459079953500 | 5.18780884025443E-06 | 0.0003520645312342580 | 3.453377725753870 | up |
| **C0_90D2.tig16152.1** | 49 | 21 | 99 | 5706 | 2254 | 2928 | 3708.1698255543800 | 7.458221165476480 | 1.0922271552519700 | 6.828452423668120 | 8.58355259479178E-12 | 4.2775557848881E-09 | 8.368804317985320 | up |
| **C0_90D3.tig00197.6** | 3 | 6 | 38 | 1703 | 921 | 733 | 1118.0132922256700 | 7.623664493778580 | 1.2557287433093400 | 6.0711077407428000 | 1.27030922943719E-09 | 1.78211575049069E-07 | 6.749064091451330 | up |
| **C0_90D2.tig05318.3** | 177 | 52 | 278 | 26460 | 9336 | 11640 | 16743.065861365300 | 8.03940646532899 | 1.1402879764820200 | 7.050329943960230 | 1.78494036518786E-12 | 1.19194747706515E-09 | 8.923742881276440 | up |
| **LP_90D3.tig15142.1** | 6 | 2 | 14 | 846 | 451 | 378 | 556.4500990132750 | 7.666483091253310 | 1.1497220764803500 | 6.668118537588430 | 2.59103481309767E-11 | 9.50681993126573E-09 | 8.021964731986560 | up |
| **C0_90D1.tig05053.1** | 68 | 35 | 251 | 16476 | 4901 | 6447 | 10198.132889707100 | 7.877582623100960 | 1.226651874371250 | 6.422019798517640 | 1.34477926554909E-10 | 2.85992578964449E-08 | 7.543645235949990 | up |
| **LP_90D1.tig09165.2** | 2 | 0 | 15 | 462 | 246 | 167 | 298.79465922031200 | 7.177225620694740 | 1.5253956460289000 | 4.705156750236810 | 2.53670972399532E-06 | 0.00017793739700521000 | 3.749732766889830 | up |
| **C0_90D1.tig05258.1** | 400 | 139 | 1363 | 86045 | 40216 | 26361 | 54108.09985936240 | 7.8573482781290100 | 1.2315418378234500 | 6.38009041740361 | 1.76983459128271E-10 | 3.60323214441089E-08 | 7.443307756809190 | up |
| **C0_90D3.tig03470.2** | 8 | 0 | 10 | 747 | 407 | 266 | 482.7120277582170 | 7.723460318459140 | 1.4108925477868600 | 5.474166215261580 | 4.39576331886169E-08 | 4.21753280038715E-06 | 5.374941531152170 | up |
| **C0_90D3.tig08581.1** | 3 | 1 | 22 | 621 | 439 | 232 | 417.0083679434810 | 7.043190192223250 | 1.324104368472490 | 5.319210750998740 | 1.04218357475301E-07 | 9.25611654476302E-06 | 5.033571185773160 | up |
| **C0_90D3.tig00043.2** | 10 | 2 | 50 | 3319 | 1579 | 1035 | 2091.79079200545 | 8.116559607377970 | 1.3629947351668300 | 5.954945678043640 | 2.60158228802836E-09 | 3.25334198557974E-07 | 6.487670282047480 | up |
| **LP_90D1.tig07760.2** | 0 | 0 | 10 | 582 | 277 | 289 | 381.9488361622000 | 8.325170810491890 | 1.7596544119281400 | 4.731139679506500 | 2.23262825870291E-06 | 0.0001589450424943100 | 3.7987530132096700 | up |
| **LP_90D1.tig00688.3** | 0 | 3 | 0 | 188 | 23 | 59 | 109.94302026953300 | 8.192417271746540 | 1.8574107941420100 | 4.410665264563010 | 1.03053519139724E-05 | 0.0006642575194124000 | 3.1776635206650300 | up |
| **C0_90D3.tig03480.1** | 11 | 6 | 37 | 2512 | 822 | 1026 | 1570.6004272648400 | 7.885652868916900 | 1.2033674215503800 | 6.552988495198990 | 5.63968630125112E-11 | 1.53092264969491E-08 | 7.815046751600440 | up |
| **C0_90D3.tig05912.2** | 16 | 10 | 111 | 3706 | 1033 | 1376 | 2281.2177732291500 | 7.100840625897470 | 1.3332754332899900 | 5.325861745142520 | 1.00475535522311E-07 | 8.99404197199583E-06 | 5.046045090065430 | up |
| **LP_90D3.tig18002.1** | 0 | 3 | 11 | 409 | 342 | 338 | 307.35772879879400 | 7.5040959720275200 | 1.2545333011090200 | 5.981583721527240 | 2.20978426597304E-09 | 2.82691520523271E-07 | 6.548687218200200 | up |
| **LP_90D1.tig00144.5** | 0 | 0 | 3 | 89 | 56 | 189 | 80.90106872840880 | 7.775891307958480 | 1.3761899557277300 | 5.650303779354790 | 1.60164539434504E-08 | 1.70309993859193E-06 | 5.7687598667141300 | up |
| **LP_3D1.tig00026.6** | 6 | 0 | 0 | 23 | 60 | 27 | 24.390679143256000 | 4.86619409810138 | 1.3942811457242900 | 3.4901096619028800 | 0.00048282237726554600 | 0.024061128887342300 | 1.6186840005189400 | up |
| **LP_90D1.tig00432.3** | 0 | 1 | 0 | 155 | 157 | 45 | 107.69393470641000 | 9.62854875228306 | 1.8371054305235600 | 5.2411519732642800 | 1.59577218350278E-07 | 1.35231567093844E-05 | 4.868921919234270 | up |
| **LP_90D2.tig25689.1** | 11 | 4 | 12 | 9 | 32 | 30 | 16.492638081343500 | 1.9275995451242300 | 0.5701973779362600 | 3.3805829695339400 | 0.0007233223336596120 | 0.03425675091994440 | 1.4652538300585700 | up |
| **C0_90D3.tig19414.1** | 6 | 0 | 21 | 1760 | 1299 | 450 | 1153.878581996320 | 8.445934854058480 | 1.5135663751477700 | 5.580154919359840 | 2.40304455991589E-08 | 2.46120413530772E-06 | 5.608852363883130 | up |
| **C0_3D1.tig02805.1** | 0 | 0 | 21 | 183 | 121 | 1094 | 269.02614129726800 | 6.748532009703140 | 1.8786587965060300 | 3.592207388725500 | 0.00032788873662163000 | 0.016973452755131200 | 1.7702298041187200 | up |
| **LP_3D2.tig30883.1** | 1 | 0 | 1 | 89 | 16 | 15 | 50.947261507563800 | 7.634338607618480 | 1.767567004367020 | 4.319122606813070 | 1.56650740521574E-05 | 0.0009650205858440660 | 3.0154634221762900 | up |
| **LP_90D1.tig03600.1** | 0 | 0 | 1 | 173 | 193 | 209 | 145.01447171076200 | 10.058184382933900 | 1.6321180039195000 | 6.162657576706700 | 7.15340868475543E-10 | 1.0956200118133E-07 | 6.960340043832540 | up |
| **LP_90D3.tig00178.4** | 0 | 0 | 1 | 57 | 34 | 171 | 58.559798320604300 | 8.7474100499517 | 1.711701230563560 | 5.1103603209257 | 3.21544966417846E-07 | 2.58078482781862E-05 | 4.588248203122360 | up |
| **C0_3D1.tig26098.1** | 59 | 14 | 56 | 2095 | 735 | 949 | 1340.3913944599800 | 6.334112013814330 | 1.1353833086077300 | 5.578831365401660 | 2.42139842601332E-08 | 2.47241810538711E-06 | 5.6068780847530300 | up |
| **LP_90D1.tig09822.2** | 0 | 0 | 2 | 140 | 38 | 34 | 83.10382711013540 | 8.405924724310610 | 1.8081358385539500 | 4.648945364101200 | 3.33636566869279E-06 | 0.0002320789860666320 | 3.6343641815969300 | up |
| **C0_90D1.tig08143.3** | 16 | 3 | 1 | 294 | 38 | 73 | 171.55990011389200 | 5.9799521156529200 | 1.5354207505774800 | 3.894666731190020 | 9.83338344571195E-05 | 0.005472113997814600 | 2.261844863753930 | up |
| **C0_90D3.tig05712.3** | 0 | 33 | 120 | 4337 | 2526 | 1958 | 2887.274375670780 | 7.300167075200250 | 1.6760502007699300 | 4.35557781732716 | 1.32716356565641E-05 | 0.0008345134518587940 | 3.0785666583604600 | up |
| **LP_90D1.tig00144.4** | 0 | 0 | 10 | 187 | 23 | 150 | 123.14940533193700 | 6.68286606780081 | 1.8994309922878000 | 3.518351598418180 | 0.00043423661284835300 | 0.02190139919394810 | 1.6595281389133200 | up |
| **C0_90D1.tig02405.2** | 24 | 0 | 30 | 2929 | 1318 | 796 | 1819.8225160071600 | 8.054657716606040 | 1.5973490792015400 | 5.042515641372680 | 4.59451186200782E-07 | 3.58425599440606E-05 | 4.445600979761140 | up |
| **LP_90D3.tig22345.1** | 0 | 0 | 2 | 195 | 79 | 75 | 122.9377169620260 | 8.96899938767908 | 1.687805663259760 | 5.31400005517028 | 1.07244585442665E-07 | 9.4981152873876E-06 | 5.022362563289230 | up |
| **C0_90D1.tig00255.3** | 201 | 73 | 412 | 22039 | 8001 | 11616 | 14279.556241843100 | 7.382816461664280 | 1.1179687445244900 | 6.603777160876220 | 4.00811873517635E-11 | 1.25072034064302E-08 | 7.902839787103600 | up |
| **C0_90D1.tig12856.1** | 10 | 11 | 7 | 8 | 42 | 32 | 17.681065241342500 | 2.0230222832618100 | 0.4946304037502350 | 4.0899675149838500 | 4.3143361042683E-05 | 0.002531658491835050 | 2.596594878873720 | up |
| **C0_90D1.tig02899.2** | 393 | 148 | 1155 | 56279 | 19006 | 29498 | 36248.58027063660 | 7.43673265158272 | 1.1750586621566700 | 6.328818203793810 | 2.47045962762171E-10 | 4.6028572574359E-08 | 7.336972493120180 | up |
| **C0_90D3.tig04734.1** | 7 | 4 | 27 | 1502 | 809 | 554 | 972.169481629482 | 7.702993973986970 | 1.1771710390843700 | 6.543648899125580 | 6.00356515913091E-11 | 1.59089711982716E-08 | 7.798357904411260 | up |
| **LP_90D1.tig05019.1** | 0 | 0 | 3 | 634 | 826 | 339 | 485.9904182456260 | 10.375214981105800 | 1.4236681865673400 | 7.287663711950940 | 3.15375969156834E-13 | 3.4489822757128E-10 | 9.462309037462100 | up |
| **C0_90D1.tig00667.4** | 65 | 29 | 137 | 6076 | 1039 | 2095 | 3634.620933223470 | 6.9772507630685 | 1.2547909373731100 | 5.56048864815304 | 2.69020351312982E-08 | 2.73018860485992E-06 | 5.563807350314320 | up |
| **LP_90D1.tig04078.1** | 0 | 0 | 1 | 82 | 17 | 16 | 47.42303526302730 | 8.437288357880440 | 2.055648126205840 | 4.104441927740510 | 4.05292060059235E-05 | 0.0023950967421801400 | 2.620676939975320 | up |
| **C0_90D1.tig01862.4** | 30 | 13 | 81 | 6017 | 1858 | 2546 | 3759.7907242802100 | 7.939206270581040 | 1.1888918400290300 | 6.677820473885310 | 2.4252186284697E-11 | 9.30754307884767E-09 | 8.031164945024960 | up |
| **C0_90D3.tig03066.2** | 41 | 10 | 121 | 7082 | 4544 | 1885 | 4573.6157594098 | 7.753455304386550 | 1.2396877050738800 | 6.254361701461310 | 3.99144981682921E-10 | 6.73083423909649E-08 | 7.17193110472362 | up |
| **C0_90D1.tig00284.1** | 20 | 10 | 196 | 12130 | 6090 | 3418 | 7633.779997073420 | 8.136043020156100 | 1.3896915747211400 | 5.854567422119340 | 4.78253168635077E-09 | 5.72343908514573E-07 | 6.242342935038490 | up |
| **C0_90D3.tig00043.1** | 61 | 16 | 214 | 12605 | 7014 | 4285 | 8132.408756670820 | 7.832641016977940 | 1.2414442197572600 | 6.3092975844774500 | 2.80304699084382E-10 | 5.08646391180892E-08 | 7.293584032451570 | up |
| **C0_90D1.tig00169.5** | 186 | 15 | 151 | 22896 | 3906 | 6457 | 13428.182779057900 | 8.212559853499110 | 1.3929038191977000 | 5.895999235775990 | 3.72420695202987E-09 | 4.57160095298991E-07 | 6.339931685424170 | up |
| **LP_90D3.tig00899.1** | 13 | 5 | 7 | 1065 | 129 | 252 | 612.092627543378 | 7.562920716443640 | 1.3498157587573500 | 5.6029281532511500 | 2.10760602380837E-08 | 2.16525715473655E-06 | 5.664490517670440 | up |
| **C0_90D1.tig22215.1** | 11 | 23 | 17 | 0 | 2 | 0 | 6.610350123753330 | -4.092503862657110 | 1.1834544066384500 | -3.4581001513034000 | 0.0005439991168796190 | 0.02686921081877750 | 1.5707450891337600 | down |
| **C0_90D1.tig00244.3** | 110 | 54 | 324 | 16779 | 5877 | 8931 | 10851.441480941500 | 7.493736313389280 | 1.1386158673160100 | 6.581443776164680 | 4.65901690320092E-11 | 1.35445507847661E-08 | 7.868235394095740 | up |
| **LP_90D1.tig00180.3** | 4 | 0 | 11 | 993 | 472 | 317 | 626.4236127816470 | 8.400602870374050 | 1.4540445349494900 | 5.777404108647770 | 7.586193069589E-09 | 8.6448942116214E-07 | 6.063240316859720 | up |
| **LP_3D1.tig08621.1** | 25 | 52 | 25 | 24 | 298 | 95 | 77.15278082169200 | 2.340305296706020 | 0.6919286428375000 | 3.382292843245740 | 0.0007188347088997280 | 0.03414106983705980 | 1.4667228740516400 | up |
| **LP_90D1.tig00319.4** | 0 | 1 | 15 | 151 | 133 | 55 | 105.70171576679600 | 5.767082167116520 | 1.5465030081879300 | 3.7291115093749100 | 0.0001921561101829290 | 0.010364944043453700 | 1.984433038161040 | up |
| **C0_90D3.tig04325.2** | 1 | 6 | 55 | 721 | 546 | 368 | 506.559139591252 | 6.082401182809810 | 1.3985811092827900 | 4.348979935764280 | 1.36772223570619E-05 | 0.0008535864995886720 | 3.0687524623751900 | up |
| **C0_90D3.tig23033.1** | 121 | 183 | 497 | 12 | 14 | 45 | 112.13371367491700 | -2.7808283261245200 | 0.7977716571705830 | -3.48574470041609 | 0.0004907691360535460 | 0.024348128801919500 | 1.6135344094894700 | down |
| **C0_90D3.tig11417.1** | 0 | 2 | 13 | 344 | 271 | 196 | 244.20790295415200 | 7.078242154851080 | 1.3887520968485900 | 5.096836340275050 | 3.45376664937976E-07 | 2.74566225371764E-05 | 4.561352886752590 | up |
| **C0_90D3.tig00310.4** | 1 | 8 | 77 | 1273 | 1055 | 775 | 921.0807989364310 | 6.480714664053490 | 1.4186703502940400 | 4.568161069067440 | 4.92021883981987E-06 | 0.000334584901920052 | 3.475493660387220 | up |
| **C0_90D3.tig01025.2** | 21 | 2 | 43 | 3652 | 1016 | 1422 | 2249.0495325423200 | 8.096248949078160 | 1.3506462554282500 | 5.994351901202350 | 2.04298549925472E-09 | 2.66457979822718E-07 | 6.574371269076370 | up |
| **LP_90D3.tig23907.1** | 0 | 0 | 1 | 15 | 20 | 90 | 23.309448730694100 | 7.419702984031060 | 1.6546503477777900 | 4.484151587672760 | 7.32046097726114E-06 | 0.00048304915329994500 | 3.3160086747954900 | up |
| **LP_90D1.tig00161.11** | 0 | 0 | 1 | 34 | 4 | 9 | 19.676025390938900 | 7.147068597486960 | 2.0898205427788800 | 3.419943699081140 | 0.0006263409724916000 | 0.030440900626669600 | 1.5165425026436900 | up |
| **LP_90D1.tig00093.12** | 4 | 0 | 0 | 423 | 59 | 127 | 247.20081595401700 | 8.84151573468303 | 1.8735191798731200 | 4.719202146242120 | 2.36771447992156E-06 | 0.0001678463243526560 | 3.775088164876720 | up |
| **LP_90D1.tig00112.5** | 45 | 13 | 50 | 4839 | 1202 | 1904 | 2967.1365536134400 | 7.75642042661581 | 1.1873625012356900 | 6.532478850008900 | 6.46899347185828E-11 | 1.68744705493653E-08 | 7.7727698446359800 | up |
| **LP_90D3.tig06676.1** | 0 | 0 | 1 | 18 | 10 | 8 | 11.945078746448700 | 6.386586741789680 | 1.8360705144316400 | 3.4783994904284400 | 0.0005044175632918650 | 0.0249881276272286 | 1.6022662845549100 | up |
| **LP_90D1.tig08164.2** | 0 | 0 | 1 | 11 | 28 | 78 | 20.54369637504830 | 7.246056219207330 | 1.5837094737567900 | 4.575369623835490 | 4.75379936050543E-06 | 0.0003239277690773800 | 3.4895518200042400 | up |
| **LP_3D2.tig05170.3** | 4 | 0 | 0 | 110 | 78 | 111 | 83.82080732326380 | 7.287545962073270 | 1.456239665661390 | 5.004358921073240 | 5.60482417357222E-07 | 4.33193227618988E-05 | 4.363318341591250 | up |
| **C0_90D3.tig00369.6** | 7 | 1 | 41 | 2882 | 1728 | 1045 | 1882.432629465090 | 8.309045298896900 | 1.3872985252716200 | 5.9893708149585700 | 2.10654377970808E-09 | 2.7360370632972E-07 | 6.562878023809950 | up |
| **C0_90D1.tig00064.9** | 171 | 61 | 522 | 37060 | 13123 | 16335 | 23464.575664712900 | 7.982911125558130 | 1.2011844261805400 | 6.645866322910740 | 3.01438749441554E-11 | 1.0166402429398E-08 | 7.992832703072450 | up |
| **LP_90D1.tig00381.3** | 0 | 0 | 2 | 396 | 167 | 184 | 254.76156627716100 | 10.022899890938600 | 1.655120505136560 | 6.055691932903440 | 1.39815354926151E-09 | 1.92904747340051E-07 | 6.714657084340470 | up |
| **LP_90D1.tig08657.1** | 2 | 0 | 1 | 68 | 36 | 120 | 57.59573663711180 | 7.2110623058925100 | 1.285996958048470 | 5.607371200034160 | 2.05422777617572E-08 | 2.12348641544059E-06 | 5.672950512972370 | up |
| **C0_90D2.tig13248.1** | 26 | 28 | 82 | 1 | 0 | 6 | 18.046116573181900 | -3.657917908474090 | 1.1207998994639300 | -3.263667234645230 | 0.001099802556641980 | 0.04948963283520100 | 1.3054857682183000 | down |
| **LP_90D1.tig00060.20** | 5 | 0 | 12 | 1207 | 627 | 520 | 787.2605054472600 | 8.545017403291910 | 1.4051842647806600 | 6.081065392961630 | 1.19386585869936E-09 | 1.68906725237767E-07 | 6.772353058093260 | up |
| **C0_90D3.tig03350.1** | 41 | 9 | 89 | 5160 | 1544 | 2282 | 3236.0703934494300 | 7.547278520766550 | 1.2360671507573900 | 6.105880668491210 | 1.02235373592798E-09 | 1.46503729136907E-07 | 6.834151320545580 | up |
| **LP_90D1.tig00375.6** | 0 | 2 | 0 | 710 | 409 | 166 | 447.40763796673000 | 10.805029072023100 | 1.716236072408500 | 6.2957708707635700 | 3.05875915301175E-10 | 5.46143900320371E-08 | 7.26269291245805 | up |
| **LP_90D2.tig00046.14** | 0 | 0 | 2 | 13 | 24 | 6 | 10.966129875134400 | 5.393746918596190 | 1.4844382592272500 | 3.6335272855362800 | 0.0002795728667217890 | 0.014700249522793400 | 1.8326752934523300 | up |
| **LP_90D1.tig00056.17** | 18 | 2 | 31 | 2531 | 738 | 1217 | 1596.614114963550 | 7.964918294942550 | 1.2894153908707300 | 6.177154663528510 | 6.52671297838844E-10 | 1.01831971792248E-07 | 6.992115846822590 | up |
| **LP_90D2.tig06146.3** | 3 | 1 | 1 | 737 | 167 | 238 | 441.02997179174900 | 9.415257277060910 | 1.4082999070790800 | 6.685548461470040 | 2.3006137072754E-11 | 9.1446656038355E-09 | 8.038832170894430 | up |
| **LP_90D1.tig01063.1** | 29 | 9 | 45 | 2649 | 1214 | 738 | 1655.4680276709100 | 7.3072473187716800 | 1.1684985070008100 | 6.253535862469570 | 4.01262367947313E-10 | 6.7325372881371E-08 | 7.171821232571140 | up |
| **C0_90D3.tig00202.3** | 3 | 6 | 64 | 2356 | 1444 | 770 | 1534.78351948655 | 7.455231089598970 | 1.375743524977270 | 5.419055917215500 | 5.99145776338979E-08 | 5.60360737428072E-06 | 5.25153230185823 | up |
| **LP_90D1.tig00175.1** | 0 | 0 | 5 | 162 | 77 | 29 | 99.25672351970580 | 7.362438563944800 | 1.7727889661894200 | 4.153025940684990 | 3.28107527856781E-05 | 0.0019632943096075400 | 2.70701459216883 | up |
| **C0_90D3.tig11086.1** | 82 | 163 | 322 | 2 | 10 | 38 | 77.00094583623980 | -3.106571620400340 | 0.8799837279734040 | -3.5302603010111900 | 0.0004151510029967490 | 0.02103410749477760 | 1.677075910949880 | down |
| **LP_90D3.tig00397.5** | 8 | 12 | 11 | 13 | 23 | 37 | 18.88047272669840 | 1.8870613777222100 | 0.5574514731083100 | 3.385158114660800 | 0.0007113726488449980 | 0.03383478827960920 | 1.4706365367595000 | up |
| **LP_90D2.tig10797.1** | 3 | 0 | 0 | 18 | 32 | 7 | 14.925756696247800 | 5.167947785474630 | 1.495538279763390 | 3.4555770690752700 | 0.0005491161842862630 | 0.02708189110359530 | 1.5673210125430100 | up |
| **LP_90D1.tig00071.13** | 0 | 3 | 19 | 832 | 416 | 356 | 541.3984532243050 | 7.683950757117630 | 1.5142508495781100 | 5.074423936601040 | 3.88671663338006E-07 | 3.06793337285879E-05 | 4.5131540763117100 | up |
| **LP_90D1.tig00243.5** | 7 | 4 | 16 | 820 | 413 | 356 | 535.4734626692860 | 7.312791745849890 | 1.0997612851533300 | 6.649435513480870 | 2.94219218553138E-11 | 1.01291112933907E-08 | 7.994428657039290 | up |
| **LP_90D2.tig06480.1** | 0 | 9 | 36 | 146387 | 91245 | 71728 | 98416.1412545565 | 14.164502291726900 | 1.5138445213270200 | 9.356642701530800 | 8.23105695448791E-21 | 9.16089202177989E-17 | 16.03806223579990 | up |
| **C0_90D1.tig00169.3** | 306 | 19 | 188 | 39296 | 6500 | 12073 | 23150.075151589700 | 8.441113115647080 | 1.4129263640805900 | 5.974205967300960 | 2.31213537654193E-09 | 2.91320332405127E-07 | 6.535629203174620 | up |
| **LP_90D1.tig00359.1** | 0 | 1 | 8 | 237 | 437 | 166 | 204.66997876815500 | 7.55334253216601 | 1.2499245284574400 | 6.043038887706090 | 1.51238182867202E-09 | 2.06954577366927E-07 | 6.684124963545830 | up |
| **C0_90D2.tig17931.1** | 16 | 19 | 39 | 0 | 0 | 1 | 9.272692494980330 | -5.326944910868180 | 1.4451061644782800 | -3.6861962406695100 | 0.00022763084862374600 | 0.012141160390891800 | 1.915739803600310 | down |
| **LP_90D1.tig00062.2** | 53 | 12 | 93 | 7300 | 2164 | 2865 | 4519.285951623490 | 7.835957145374680 | 1.2223101755186500 | 6.410776333470120 | 1.44780472742598E-10 | 3.04029887069347E-08 | 7.5170837218146000 | up |
| **LP_90D1.tig14299.1** | 3 | 3 | 8 | 1113 | 583 | 304 | 701.4344073411860 | 8.658357781145980 | 1.1795520034477700 | 7.340378173949120 | 2.12991168456181E-13 | 2.73521620137824E-10 | 9.563008339767640 | up |
| **LP_90D1.tig00049.12** | 0 | 0 | 1 | 109 | 49 | 45 | 69.76463902618060 | 8.997401540906570 | 1.8739337119030900 | 4.80134461734465 | 1.57603774803319E-06 | 0.00011414820904355800 | 3.942530898090540 | up |
| **C0_90D1.tig01009.3** | 31 | 9 | 91 | 3288 | 1632 | 1870 | 2209.5658007900700 | 7.092022602348300 | 1.1535653452238500 | 6.147915791430160 | 7.85076917811584E-10 | 1.17546785689735E-07 | 6.929789242134930 | up |
| **C0_90D1.tig09193.1** | 298 | 515 | 354 | 5 | 11 | 18 | 152.7961594535520 | -4.531988732935110 | 0.5060295804461040 | -8.955975911407800 | 3.36743522804189E-19 | 2.24870589658181E-15 | 14.64806734129350 | down |
| **LP_90D3.tig12878.1** | 1 | 1 | 6 | 136 | 64 | 43 | 86.36628541312630 | 6.4408128317178900 | 1.313740531807310 | 4.902652141558930 | 9.45513327881963E-07 | 7.06258266323285E-05 | 4.151036455990090 | up |
| **LP_90D1.tig04144.2** | 0 | 1 | 18 | 1170 | 613 | 499 | 763.2877503983210 | 8.399675729630590 | 1.6149973262063200 | 5.201046214337530 | 1.98169813967519E-07 | 1.65417297964037E-05 | 4.781419077507030 | up |
| **LP_90D1.tig00176.2** | 3 | 2 | 6 | 2993 | 95 | 502 | 1648.7973085168800 | 10.234847799035100 | 1.584108087680590 | 6.460952935364830 | 1.04045678274383E-10 | 2.39584907027819E-08 | 7.620540544383700 | up |
| **C0_90D1.tig01758.4** | 560 | 177 | 1159 | 100290 | 32348 | 42497 | 62834.184819762800 | 8.057456063945220 | 1.1768415949694800 | 6.846678515092930 | 7.55843118770701E-12 | 3.88259167578999E-09 | 8.410878280913080 | up |
| **C0_90D3.tig08057.1** | 37 | 47 | 84 | 3 | 1 | 5 | 23.172383756173800 | -3.432755363882470 | 0.8703101415673630 | -3.944289742160580 | 8.00368413243716E-05 | 0.004521742969508370 | 2.3446941279917100 | down |
| **C0_90D3.tig00805.2** | 22 | 13 | 63 | 2763 | 696 | 1153 | 1709.0220318805400 | 7.138355131352880 | 1.1970572471848100 | 5.963252925572750 | 2.47265226483932E-09 | 3.10373633348571E-07 | 6.508115179738570 | up |
| **LP_90D2.tig04427.2** | 0 | 0 | 1 | 32 | 18 | 79 | 30.3729423354231 | 7.787863006757210 | 1.7133156498274900 | 4.545492249219440 | 5.48070141477069E-06 | 0.000369687150581371 | 3.432165644175570 | up |
| **C0_90D3.tig03109.1** | 73 | 142 | 615 | 21727 | 11517 | 14081 | 14933.568264165800 | 7.214004798108230 | 1.1208551388309600 | 6.436161595005390 | 1.22532581574368E-10 | 2.63950991366876E-08 | 7.57847670250426 | up |
| **LP_90D1.tig00209.9** | 0 | 4 | 27 | 1020 | 569 | 346 | 658.9505897714580 | 7.474751097857160 | 1.5734683622127800 | 4.750493417831020 | 2.02920899996816E-06 | 0.00014477192158106200 | 3.839315661100660 | up |
| **LP_90D1.tig00426.1** | 8 | 0 | 6 | 534 | 137 | 189 | 325.29032785862900 | 7.490247133393550 | 1.4974938562485500 | 5.001855000699480 | 5.67812915735226E-07 | 4.3683653095584E-05 | 4.359681050410580 | up |
| **C0_90D2.tig00475.4** | 8 | 165 | 604 | 6344 | 5081 | 6566 | 4993.3037084523400 | 5.726635637874500 | 1.3240128078787400 | 4.32521166245317 | 1.52385472732741E-05 | 0.0009432532910833550 | 3.0253716708374400 | up |
| **LP_90D1.tig00177.5** | 13 | 1 | 35 | 1698 | 952 | 812 | 1131.0069297071600 | 7.543404902719950 | 1.3051726792787000 | 5.779622131600850 | 7.48685882001625E-09 | 8.56091538155899E-07 | 6.067479795614290 | up |
| **LP_90D2.tig00043.6** | 9 | 3 | 22 | 28 | 26 | 385 | 76.84602673268450 | 4.115857323518340 | 1.172733276378530 | 3.5096278125818700 | 0.00044873433269499300 | 0.022496682634163900 | 1.647881518328450 | up |
| **C0_90D1.tig23884.2** | 8 | 4 | 33 | 1541 | 805 | 769 | 1023.4118254489300 | 7.535351135888180 | 1.1650221457959800 | 6.467989611253110 | 9.93153240057233E-11 | 2.33272688931139E-08 | 7.632136104501610 | up |
| **C0_90D3.tig11779.1** | 32 | 51 | 122 | 1 | 2 | 12 | 27.52706860442520 | -3.265691781400070 | 0.9476647038579320 | -3.446041377404340 | 0.0005688632494810650 | 0.0279731591118163 | 1.5532584843844600 | down |
| **LP_90D1.tig00144.2** | 0 | 0 | 4 | 70 | 29 | 147 | 61.65382578265260 | 6.978774887858120 | 1.4848574900981500 | 4.699962746860520 | 2.60208954135665E-06 | 0.00018214081278062300 | 3.7395927297709800 | up |
| **C0_90D3.tig01175.4** | 47 | 29 | 140 | 8011 | 4250 | 3723 | 5287.69034831165 | 7.632787422892330 | 1.0771972783648500 | 7.085784169895630 | 1.38259146986217E-12 | 9.61736387233917E-10 | 9.016943952139460 | up |
| **C0_90D1.tig00882.3** | 36 | 1 | 1 | 2349 | 377 | 649 | 1373.2430749775900 | 8.04892663140404 | 1.719262918288210 | 4.681614746520540 | 2.8462400471533E-06 | 0.00019881403542761800 | 3.7015529595081100 | up |
| **LP_90D1.tig00180.5** | 0 | 0 | 6 | 263 | 115 | 76 | 163.7107948016520 | 7.828288848635040 | 1.7381330885199900 | 4.503848928680590 | 6.67336540720326E-06 | 0.00044297613833222600 | 3.3536196671623700 | up |
| **LP_90D1.tig00213.6** | 0 | 0 | 1 | 188 | 181 | 245 | 156.42230055072700 | 10.168208286063200 | 1.6452664974692400 | 6.180280399378490 | 6.39878476518306E-10 | 1.00304706349623E-07 | 6.998678689175800 | up |
| **LP_90D1.tig00057.13** | 1999 | 403 | 3955 | 273092 | 178442 | 48297 | 173290.7255996220 | 7.772291045172950 | 1.256905623055120 | 6.183671154466720 | 6.26277621003956E-10 | 9.86357711684013E-08 | 7.005965555609380 | up |
| **LP_90D1.tig00946.2** | 1 | 2 | 9 | 339 | 95 | 126 | 208.68974647778500 | 7.150833287650190 | 1.335446206171670 | 5.354639711134080 | 8.5727060826882E-08 | 7.79929382547347E-06 | 5.107944718025420 | up |
| **LP_90D3.tig00122.1** | 1 | 1 | 1 | 1272 | 619 | 338 | 792.6291356134480 | 11.033554814562200 | 1.4218998696901100 | 7.75972700311651 | 8.51123943969526E-15 | 2.02986981179989E-11 | 10.692531815205000 | up |
| **LP_90D1.tig00071.12** | 0 | 0 | 4 | 325 | 152 | 165 | 213.27012883915400 | 8.784656571706070 | 1.590293282325670 | 5.523922328879640 | 3.31514062304175E-08 | 3.24601848277832E-06 | 5.488649011636780 | up |
| **LP_90D1.tig14603.1** | 1 | 0 | 1 | 66 | 24 | 26 | 41.520108072398100 | 7.332837237553040 | 1.5852582019821900 | 4.625642200358360 | 3.73439488822044E-06 | 0.0002586881969352540 | 3.5872233862151500 | up |
| **LP_90D1.tig00016.10** | 51 | 25 | 130 | 9060 | 3999 | 6540 | 6202.51073818258 | 7.926854056822480 | 1.0508736553106200 | 7.543108552359190 | 4.58898852491202E-14 | 8.51231876990486E-11 | 10.069952121254600 | up |
| **LP_90D1.tig00052.8** | 1 | 0 | 2 | 71 | 33 | 18 | 44.27135105569620 | 6.856889766773400 | 1.5182153003519500 | 4.516414612067120 | 6.28954616733E-06 | 0.0004208450039699020 | 3.375877824152050 | up |
| **LP_90D1.tig00052.7** | 1 | 1 | 3 | 237 | 99 | 65 | 146.41366484810200 | 7.873668367900710 | 1.345650713922840 | 5.851197704155640 | 4.88045670991854E-09 | 5.81977032455251E-07 | 6.235094154310200 | up |
| **C0_90D3.tig00962.3** | 0 | 5 | 26 | 272 | 350 | 129 | 209.12798183832000 | 5.796891211332790 | 1.4335249271442900 | 4.043802170137870 | 5.25912995970282E-05 | 0.0030432771269413800 | 2.5166584981113100 | up |
| **C0_90D1.tig01819.3** | 275 | 89 | 547 | 49421 | 14128 | 20054 | 30601.608179396500 | 8.075183747056900 | 1.1910179339482100 | 6.780068978716200 | 1.20118518284756E-11 | 5.3155377089362E-09 | 8.274452796575860 | up |
| **LP_90D1.tig02896.2** | 4 | 6 | 20 | 1383 | 651 | 397 | 866.3877154901430 | 7.882075884170250 | 1.194128083049560 | 6.600695516716300 | 4.09233284299224E-11 | 1.26517501198767E-08 | 7.897849394264250 | up |
| **C0_90D1.tig07432.1** | 103 | 11 | 112 | 13039 | 3340 | 4567 | 7919.972380301260 | 8.105355311824130 | 1.3034675440831400 | 6.218302364809140 | 5.02562422908967E-10 | 8.18539353097926E-08 | 7.086960436078030 | up |
| **C0_90D3.tig07389.1** | 38 | 33 | 98 | 0 | 5 | 4 | 22.02810830345450 | -3.8010979286041300 | 0.8639030456714130 | -4.399912637939560 | 1.08294467044804E-05 | 0.0006940199539652540 | 3.1586280428413700 | down |
| **LP_90D1.tig08343.2** | 0 | 0 | 1 | 75 | 5 | 4 | 40.518048810189500 | 8.211263646461770 | 2.3175311376543600 | 3.5431082297227000 | 0.0003954403768587800 | 0.020096436442827700 | 1.6968809460831500 | up |
| **LP_90D1.tig00049.15** | 0 | 0 | 1 | 90 | 40 | 21 | 55.27077750034690 | 8.659245626489670 | 1.9470769419938800 | 4.447305311736810 | 8.69542071275973E-06 | 0.0005637502954919120 | 3.24891321747678 | up |
| **LP_90D1.tig00381.1** | 0 | 0 | 4 | 1175 | 196 | 501 | 710.7956412046720 | 10.531260421483300 | 1.7771240380057900 | 5.926013151733080 | 3.10377740212996E-09 | 3.83822309924879E-07 | 6.415869785213710 | up |
| **LP_90D1.tig02361.2** | 0 | 0 | 2 | 29 | 18 | 30 | 21.96367125637900 | 6.432249454636760 | 1.4836588995431000 | 4.335396401839810 | 1.45497686743685E-05 | 0.0009029781157406890 | 3.0443227749669200 | up |
| **LP_90D2.tig13935.2** | 82 | 66 | 42 | 2 | 0 | 0 | 25.39571200908640 | -5.538698003291480 | 1.4749462918396700 | -3.7551862287698400 | 0.00017321263745907000 | 0.009403897157920150 | 2.02669212901456 | down |
| **C0_90D3.tig11584.1** | 11 | 18 | 16 | 0 | 0 | 2 | 5.90102119178421 | -3.894057659180820 | 1.15159569701514 | -3.381445128072260 | 0.0007210563246372040 | 0.03419793980584030 | 1.4660000564557200 | down |
| **LP_90D1.tig00056.14** | 4 | 0 | 19 | 1436 | 765 | 454 | 915.9755673592860 | 8.353628414729430 | 1.5263115258512800 | 5.473082180959280 | 4.42274881185212E-08 | 4.23126533177451E-06 | 5.373529740323990 | up |
| **LP_90D1.tig02523.2** | 2 | 9 | 44 | 1343 | 589 | 834 | 902.4985008258450 | 7.088922576749570 | 1.279359933437260 | 5.540991547002530 | 3.00763737604469E-08 | 2.99767177160466E-06 | 5.523215921716130 | up |
| **LP_90D3.tig11059.1** | 6 | 8 | 6 | 11 | 18 | 37 | 15.835418207592400 | 2.3556597685716900 | 0.6388391784625530 | 3.687406546105830 | 0.00022655125446428500 | 0.012102911736492800 | 1.9171101339012700 | up |
| **C0_90D3.tig11977.1** | 66 | 121 | 243 | 3 | 14 | 29 | 60.08018577663280 | -2.761710877298770 | 0.721911351990048 | -3.825554023614850 | 0.00013047831585209300 | 0.00717716719602228 | 2.14404693652314 | down |
| **C0_90D3.tig05195.1** | 2 | 1 | 27 | 968 | 450 | 283 | 607.4333716282140 | 7.395006815590430 | 1.4649622468996700 | 5.047916307222670 | 4.46654719905201E-07 | 3.49258886250931E-05 | 4.456852535247720 | up |
| **LP_90D2.tig00446.1** | 2 | 0 | 0 | 74 | 28 | 10 | 43.9618122377309 | 7.360129197287130 | 1.851080156802510 | 3.976126679463060 | 7.00468216370678E-05 | 0.004004783095274070 | 2.39742100094717 | up |
| **C0_90D1.tig00064.22** | 145 | 48 | 503 | 32641 | 11673 | 14508 | 20702.19822445500 | 7.922544961424230 | 1.2289076323238800 | 6.446818908954630 | 1.1422212804028E-10 | 2.50905436390586E-08 | 7.600489928685970 | up |
| **LP_90D2.tig01797.2** | 0 | 0 | 4 | 919 | 474 | 518 | 615.189416085528 | 10.315624370000400 | 1.5581175141209800 | 6.620568908642330 | 3.57819064745567E-11 | 1.14877122622978E-08 | 7.9397664509274400 | up |
| **LP_90D3.tig04214.1** | 0 | 0 | 9 | 29 | 44 | 127 | 39.906351836756900 | 5.176366370414140 | 1.4719102299616700 | 3.51676771113203 | 0.00043683589233433000 | 0.021966134953540600 | 1.658246352547720 | up |
| **LP_90D2.tig22735.1** | 0 | 1 | 1 | 185 | 269 | 105 | 146.41011585530700 | 9.194423174381490 | 1.3856331089771200 | 6.635539461935080 | 3.23317852626251E-11 | 1.05835880209195E-08 | 7.975367073934280 | up |
| **LP_90D3.tig11070.1** | 0 | 5 | 0 | 2816 | 2849 | 757 | 1945.8980707923700 | 11.611220765800400 | 1.6356744309512800 | 7.098735876825770 | 1.25903160348033E-12 | 9.1386535236097E-10 | 9.039117787892380 | up |
| **C0_90D3.tig14601.1** | 4 | 1 | 21 | 724 | 453 | 252 | 475.4888099877020 | 7.225705785741360 | 1.3126684398984000 | 5.5045932134245700 | 3.70021645341322E-08 | 3.58105875834823E-06 | 5.445988552985010 | up |
| **LP_90D3.tig00418.3** | 2 | 2 | 0 | 7 | 7 | 15 | 7.209910847265930 | 3.6150149695213700 | 1.0845258186815000 | 3.3332677814128000 | 0.0008583228871864090 | 0.03965316958896030 | 1.4017220925839900 | up |
| **LP_90D1.tig00113.4** | 1 | 1 | 4 | 9086 | 4855 | 2888 | 5782.843408197570 | 12.933407891163300 | 1.2895315726126000 | 10.029539536562200 | 1.13042537030945E-23 | 1.88718863446311E-19 | 18.72418468764010 | up |
| **C0_3D1.tig00270.2** | 771 | 551 | 489 | 4304 | 2820 | 3118 | 3285.804803465820 | 3.722012397894180 | 0.8653617418876200 | 4.30110578932612 | 1.69947896347E-05 | 0.001045007423780850 | 2.980880624293980 | up |
| **C0_3D1.tig01699.1** | 2 | 4 | 85 | 1638 | 875 | 798 | 1092.5073498906600 | 6.64874040203452 | 1.4888970464233400 | 4.46554744534301 | 7.98642703617856E-06 | 0.0005198027530428190 | 3.284161424665440 | up |
| **LP_90D2.tig00454.2** | 1 | 2 | 27 | 858 | 324 | 448 | 557.1749120353950 | 7.274317257262940 | 1.427375308940100 | 5.096289120108500 | 3.46376004782229E-07 | 2.74706613388928E-05 | 4.561130885087100 | up |
| **C0_90D1.tig00064.17** | 66 | 30 | 247 | 17831 | 6651 | 8620 | 11438.4345800594 | 8.090269458638610 | 1.1907012985292700 | 6.794541560197770 | 1.08657490665806E-11 | 4.90265534573056E-09 | 8.309568636370080 | up |
| **C0_90D3.tig06706.1** | 11 | 10 | 45 | 0 | 1 | 1 | 8.307406238193290 | -4.428376502030000 | 1.3459598343792500 | -3.29012529862926 | 0.0010014277879717000 | 0.045430261430145600 | 1.3426547640142800 | down |
| **C0_90D3.tig05919.1** | 16 | 17 | 54 | 0 | 0 | 2 | 10.930875862531500 | -4.814262769125740 | 1.3275001196744000 | -3.6265629643080900 | 0.00028721880741388800 | 0.015054864616550000 | 1.822323145601220 | down |
| **LP_90D2.tig13556.1** | 3 | 25 | 267 | 8189 | 3771 | 3647 | 5316.717191406220 | 7.2411152882786100 | 1.5212059908495100 | 4.760114890314640 | 1.93482787670352E-06 | 0.00013863083256492200 | 3.8581401685436 | up |
| **C0_90D1.tig00430.3** | 93 | 19 | 190 | 13300 | 4424 | 5625 | 8354.787889061530 | 7.79494325519712 | 1.2266106144568800 | 6.354863689687350 | 2.0861152377987E-10 | 4.09725303969769E-08 | 7.387507213896280 | up |
| **LP_90D1.tig00688.2** | 3 | 0 | 0 | 2266 | 330 | 640 | 1317.7844593551100 | 11.684535687806400 | 1.8571610249329800 | 6.2916115139925000 | 3.14186985157029E-10 | 5.5799942805362E-08 | 7.253366246211680 | up |
| **C0_90D3.tig02318.2** | 6 | 3 | 41 | 1837 | 1042 | 769 | 1209.096232532430 | 7.640151937855610 | 1.27107503626221 | 6.0107796313289600 | 1.84633240488139E-09 | 2.43664793148557E-07 | 6.613207217002730 | up |
| **LP_90D1.tig01238.3** | 0 | 0 | 13 | 172 | 99 | 411 | 162.50527771366800 | 6.706525118254140 | 1.7026111172044600 | 3.938964717477990 | 8.1833962141743E-05 | 0.0046154631114031400 | 2.3357847157353400 | up |
| **LP_90D3.tig05529.2** | 0 | 0 | 1 | 46 | 46 | 77 | 41.00457409206770 | 8.218551336512550 | 1.620914500865340 | 5.070317609056510 | 3.9715236371801E-07 | 3.12748119626902E-05 | 4.504805292787590 | up |
| **LP_90D1.tig00078.3** | 24 | 9 | 42 | 3804 | 1878 | 1118 | 2397.295983185520 | 7.996782632662010 | 1.1440990560198700 | 6.989589398387840 | 2.75691897211141E-12 | 1.6437637064255E-09 | 8.78416061281247 | up |
| **C0_90D2.tig02357.1** | 100 | 3131 | 19862 | 210055 | 164967 | 163581 | 156940.23607417200 | 5.8164231847804600 | 1.492415960266400 | 3.8973204117585300 | 9.72628988106821E-05 | 0.005421554137545680 | 2.2658762011719000 | up |
| **C0_90D1.tig00201.4** | 118 | 64 | 357 | 16539 | 8924 | 8579 | 11074.048328507400 | 7.379870635944 | 1.0753574910272800 | 6.862713746378520 | 6.75645322310572E-12 | 3.58081296295678E-09 | 8.44601836295268 | up |
| **LP_90D1.tig05133.1** | 1 | 0 | 0 | 355 | 170 | 93 | 220.70139535331200 | 10.667702085188200 | 1.92438545246101 | 5.543433136820780 | 2.96597962948929E-08 | 2.96500280985084E-06 | 5.527974890730700 | up |
| **LP_90D1.tig14925.1** | 0 | 0 | 1 | 63 | 8 | 5 | 34.775509892750400 | 7.986714377033890 | 2.2103880238120500 | 3.613263504414000 | 0.0003023671426317200 | 0.015774588320828900 | 1.802041965987180 | up |
| **C0_90D2.tig14414.1** | 8 | 24 | 28 | 0 | 1 | 2 | 7.7714073513571700 | -3.7889436358970000 | 1.1049929430371200 | -3.4289301662714000 | 0.0006059654221939020 | 0.029623103194190600 | 1.528369448531860 | down |
| **LP_90D1.tig04005.1** | 0 | 0 | 1 | 331 | 423 | 220 | 258.70992107357200 | 10.897019758871900 | 1.6952822288123000 | 6.427849931811180 | 1.29421412200045E-10 | 2.77003303329956E-08 | 7.557515051840180 | up |
| **C0_90D1.tig00244.4** | 143 | 86 | 390 | 18127 | 7345 | 10602 | 11998.13662866030 | 7.291114409275820 | 1.0712750275179100 | 6.806015469406570 | 1.00338927866839E-11 | 4.72882307012682E-09 | 8.325246934896620 | up |
| **LP_90D3.tig05078.2** | 0 | 0 | 8 | 446 | 709 | 527 | 400.000058387692 | 8.70506534772327 | 1.4360921617568800 | 6.061634190018650 | 1.34745400003527E-09 | 1.86681085506961E-07 | 6.7288996824584500 | up |
| **LP_90D3.tig07539.1** | 0 | 3 | 3 | 7 | 20 | 30 | 11.214021476907700 | 3.805497776938900 | 0.8315489077350780 | 4.5763968198865 | 4.73052856270173E-06 | 0.0003230012641718770 | 3.4907957779110100 | up |
| **LP_90D1.tig00008.20** | 0 | 0 | 4 | 526 | 268 | 198 | 337.9003310717650 | 9.451857283243190 | 1.6234900255207700 | 5.821937390844970 | 5.81693417205104E-09 | 6.76730366099693E-07 | 6.169584335510550 | up |
| **LP_90D1.tig01014.2** | 0 | 0 | 3 | 44 | 74 | 228 | 65.24089312274770 | 7.459020432094500 | 1.2146704152356200 | 6.140777233507920 | 8.21186789136207E-10 | 1.22404489743164E-07 | 6.912202652166600 | up |
| **LP_3D2.tig14292.1** | 6 | 6 | 3 | 781 | 699 | 340 | 548.1790480778720 | 8.15779731294313 | 1.0187171688970700 | 8.007911873886760 | 1.16672458685552E-15 | 3.89557672305189E-12 | 11.409428237837900 | up |
| **C0_90D3.tig06403.1** | 2 | 0 | 19 | 1178 | 590 | 393 | 749.723964561738 | 8.212231164857100 | 1.5869020112512200 | 5.175008353781100 | 2.27900783898887E-07 | 1.88350972118811E-05 | 4.72503213397016 | up |
| **C0_90D1.tig00430.4** | 46 | 21 | 128 | 9530 | 2963 | 3795 | 5923.60942931284 | 7.943421380604900 | 1.1909756196047300 | 6.669675894155770 | 2.56368953404731E-11 | 9.50681993126573E-09 | 8.021964731986560 | up |
| **LP_3D3.tig21514.1** | 21 | 43 | 78 | 0 | 4 | 9 | 19.16386350619000 | -3.039140556118860 | 0.8946980096009570 | -3.396833930003200 | 0.00068170324958469 | 0.03260944097476110 | 1.4866566462617400 | down |
| **LP_90D1.tig01190.1** | 34 | 0 | 2 | 5634 | 2481 | 1389 | 3465.09816691105 | 9.468028492957840 | 1.789165889060720 | 5.291867316969910 | 1.21073722287766E-07 | 1.04762486926018E-05 | 4.979794200534830 | up |
| **LP_90D1.tig00617.3** | 4 | 34 | 1156 | 374384 | 90746 | 193448 | 234909.07308865700 | 10.704570147216300 | 1.7657440664727300 | 6.062356572773230 | 1.3414141383616E-09 | 1.86618652773981E-07 | 6.729044950128880 | up |
| **LP_90D1.tig07785.1** | 3 | 0 | 5 | 270 | 153 | 79 | 172.9745835014140 | 7.421496627342260 | 1.3859235230751400 | 5.354910645340070 | 8.55987075170806E-08 | 7.79929382547347E-06 | 5.107944718025420 | up |
| **C0_90D1.tig17664.1** | 174 | 94 | 437 | 44938 | 23931 | 13443 | 28554.520938364000 | 8.35600400505102 | 1.1235805281370100 | 7.4369426986296800 | 1.03042219879714E-13 | 1.49585942589729E-10 | 9.825109217584950 | up |
| **LP_90D1.tig01885.2** | 30 | 28 | 233 | 10050 | 6086 | 2881 | 6478.092377885990 | 7.524616882766510 | 1.2572735293240100 | 5.984868612331480 | 2.16564605548048E-09 | 2.78110600563222E-07 | 6.555782457096390 | up |
| **LP_90D1.tig00067.2** | 0 | 0 | 8 | 362 | 139 | 147 | 228.84584967560100 | 7.902901003493460 | 1.7629843712131400 | 4.482683529437840 | 7.37102018183417E-06 | 0.000485426021402882 | 3.3138769468982300 | up |
| **LP_90D3.tig17630.1** | 0 | 1 | 0 | 77 | 89 | 52 | 59.18745050156350 | 8.755713250679370 | 1.7009523069999400 | 5.14753600947358 | 2.63930349857153E-07 | 2.15461380229352E-05 | 4.666630562423680 | up |
| **LP_90D3.tig03342.1** | 14 | 49 | 52 | 330 | 93 | 153 | 220.21843973759900 | 3.864489607393170 | 1.131507237299940 | 3.4153467870119900 | 0.0006370085458752850 | 0.030914358049752700 | 1.5098397673874800 | up |
| **C0_90D3.tig00040.7** | 125 | 31 | 481 | 28140 | 14823 | 9677 | 18061.784080889500 | 7.857479354128600 | 1.2676047569568000 | 6.198682444985810 | 5.69377782987289E-10 | 9.22861883308863E-08 | 7.034863291179060 | up |
| **LP_90D1.tig00659.2** | 0 | 0 | 24 | 172 | 290 | 1036 | 277.12406883958800 | 6.597602603518370 | 1.8085523981740000 | 3.648001910356380 | 0.00026428767370768200 | 0.01396182797367480 | 1.8550577173211800 | up |
| **C0_90D3.tig03299.2** | 0 | 4 | 12 | 120 | 65 | 46 | 79.50000604504980 | 5.329645707700070 | 1.4144183796690500 | 3.7680828984611400 | 0.00016450607650254800 | 0.008989678213328270 | 2.046255853614610 | up |
| **C0_90D3.tig00137.2** | 9 | 3 | 24 | 1646 | 838 | 535 | 1048.2415025346700 | 7.87806744001824 | 1.2020368559914600 | 6.553931687494150 | 5.60416112831034E-11 | 1.53092264969491E-08 | 7.815046751600440 | up |
| **LP_90D1.tig00309.5** | 3 | 2 | 8 | 1250 | 873 | 479 | 835.0402886970970 | 9.017363400619760 | 1.1246178048360400 | 8.018158135006980 | 1.07342558537771E-15 | 3.89557672305189E-12 | 11.409428237837900 | up |
| **C0_90D3.tig00237.2** | 7 | 5 | 21 | 1148 | 585 | 369 | 731.5361975893470 | 7.485191668449800 | 1.1481465746258100 | 6.519369420136340 | 7.06035724414992E-11 | 1.79952876354902E-08 | 7.744841207221250 | up |
| **C0_90D1.tig17959.1** | 31 | 28 | 78 | 1 | 8 | 10 | 19.858346427526400 | -2.3873118140871300 | 0.7184247845279210 | -3.3229808680053200 | 0.0008906102251095990 | 0.04095948320411070 | 1.3876455315981200 | down |
| **C0_90D3.tig01855.2** | 0 | 2 | 42 | 369 | 283 | 191 | 261.51357295214500 | 5.622584870636780 | 1.6702508132290400 | 3.366311709656830 | 0.0007618057056431600 | 0.03582525451509790 | 1.4458107155402500 | up |
| **C0_90D3.tig00310.5** | 0 | 0 | 33 | 527 | 352 | 364 | 376.2097574314780 | 6.580437631055780 | 1.9866885159212800 | 3.3122643928931500 | 0.0009254404010867370 | 0.04250279168072220 | 1.3715825435563900 | up |
| **C0_90D2.tig01792.1** | 159 | 56 | 462 | 30616 | 10025 | 12884 | 19199.61786046900 | 7.8463466967510200 | 1.2102475053238800 | 6.483257897442400 | 8.97628667389337E-11 | 2.17180605619294E-08 | 7.663178960154060 | up |
| **LP_90D1.tig05424.2** | 6 | 6 | 6 | 17 | 18 | 22 | 16.592730584731000 | 2.562801271479010 | 0.7537318123932270 | 3.400150065766340 | 0.0006734887978589190 | 0.03230907682717160 | 1.4906754509762600 | up |
| **LP_90D1.tig00493.5** | 0 | 0 | 3 | 161 | 220 | 172 | 137.19608566847500 | 8.535251259570230 | 1.2710718484563200 | 6.7150029873890000 | 1.8806236717193E-11 | 7.84901797187947E-09 | 8.105184676512430 | up |
| **LP_90D1.tig07957.2** | 0 | 1 | 6 | 251 | 152 | 79 | 162.74785064046600 | 7.583013095930790 | 1.4474149661980800 | 5.239004206132440 | 1.61445359421377E-07 | 1.36468331790389E-05 | 4.8649681172996300 | up |
| **LP_90D2.tig00242.8** | 0 | 1 | 5 | 547 | 387 | 259 | 373.0796109143990 | 9.001354850787180 | 1.3474867822618100 | 6.680106231304230 | 2.38769105802209E-11 | 9.30754307884767E-09 | 8.031164945024960 | up |
| **C0_90D3.tig07434.1** | 28 | 21 | 74 | 2 | 0 | 2 | 16.468152503323200 | -4.019348334227190 | 1.1863175442366400 | -3.3880880829537900 | 0.0007038165784508940 | 0.03352315511825520 | 1.474655113372200 | down |
| **C0_90D3.tig05977.1** | 25 | 34 | 79 | 1 | 8 | 10 | 19.89537631286750 | -2.3922597527945 | 0.72554368407167 | -3.297196027356210 | 0.0009765531754088890 | 0.04454389887121230 | 1.351211772390740 | down |
| **C0_90D1.tig03235.2** | 22 | 2 | 34 | 1909 | 254 | 480 | 1105.5970871211400 | 7.2399904653476500 | 1.4588441876242300 | 4.962826412009210 | 6.94746708945574E-07 | 5.30821461441277E-05 | 4.2750515266570800 | up |
| **C0_90D3.tig06405.3** | 252 | 39 | 464 | 31779 | 6523 | 10816 | 19074.19622923540 | 7.658430710732070 | 1.328358218361670 | 5.765335438039890 | 8.1495587345042E-09 | 9.16180527226803E-07 | 6.038018943085820 | up |
| **C0_90D1.tig03006.1** | 368 | 65 | 819 | 64639 | 16715 | 22542 | 39281.18019467820 | 7.981456233369520 | 1.3040545159905900 | 6.120492767364590 | 9.32864174856099E-10 | 1.37213224380045E-07 | 6.86260403003956 | up |
| **LP_90D1.tig00832.4** | 0 | 0 | 2 | 28 | 22 | 98 | 31.613234675011000 | 6.982245963936230 | 1.4169535776571700 | 4.9276462362873300 | 8.32261293153931E-07 | 6.27044056312822E-05 | 4.202701944430250 | up |
| **C0_3D1.tig01699.3** | 8 | 1 | 142 | 1793 | 1266 | 933 | 1250.1017180739800 | 6.102461011015730 | 1.5684500297110100 | 3.8907589629362400 | 9.99311632167325E-05 | 0.005551749764797810 | 2.2555701171599400 | up |
| **C0_90D3.tig14842.1** | 15 | 23 | 43 | 0 | 0 | 4 | 10.526827516982500 | -3.8396075293057400 | 1.1421035660762600 | -3.361873339120080 | 0.000774156253847582 | 0.03630379657263610 | 1.4400479550099600 | down |
| **LP_90D1.tig00832.3** | 0 | 0 | 3 | 80 | 70 | 337 | 99.01831993707980 | 8.071029716862760 | 1.3476909877170600 | 5.988783623562540 | 2.11416203639126E-09 | 2.7360370632972E-07 | 6.562878023809950 | up |
| **C0_90D1.tig21857.1** | 17 | 10 | 16 | 0 | 1 | 2 | 5.862692027942050 | -3.3386421199589200 | 1.0202060725260100 | -3.272517396110500 | 0.0010659431270249200 | 0.04817377281332980 | 1.3171893398211900 | down |
| **LP_90D3.tig11445.1** | 0 | 1 | 2 | 450 | 131 | 191 | 279.4669041496130 | 9.565966967825310 | 1.5115235141985100 | 6.328692129475510 | 2.4724786328027E-10 | 4.6028572574359E-08 | 7.336972493120180 | up |
| **LP_90D2.tig06818.1** | 1 | 1 | 0 | 14984 | 9540 | 7876 | 10175.067376875200 | 15.285495959880700 | 1.4797539763711600 | 10.329754948431200 | 5.16924725485415E-25 | 1.72595996592325E-20 | 19.76296928207400 | up |
| **LP_90D2.tig02223.3** | 0 | 1 | 0 | 256 | 423 | 79 | 199.5009718158920 | 10.522049586047700 | 1.7997494545815400 | 5.8463968744439600 | 5.02334744803461E-09 | 5.94767900505062E-07 | 6.225652478296860 | up |
| **C0_90D1.tig00064.15** | 143 | 48 | 432 | 28718 | 10330 | 13432 | 18318.199763840200 | 7.900311699045650 | 1.1985089387950300 | 6.5917837100059900 | 4.34573399549472E-11 | 1.33119002179425E-08 | 7.875759946383830 | up |
| **C0_90D3.tig00209.2** | 4 | 2 | 15 | 820 | 473 | 393 | 547.6668995986540 | 7.726751573927510 | 1.1596057863750400 | 6.663257173010080 | 2.67824396868599E-11 | 9.71998781200615E-09 | 8.012334279639750 | up |
| **LP_90D1.tig05175.1** | 0 | 3 | 0 | 1496 | 1113 | 178 | 950.46347208938 | 11.311758513240500 | 1.7625793158642600 | 6.417730204495150 | 1.3832110557526E-10 | 2.92304012281794E-08 | 7.534165223301870 | up |
| **C0_90D2.tig20821.2** | 12 | 7 | 63 | 3216 | 1103 | 1378 | 2027.9014757890600 | 7.666299525419560 | 1.25392226764585 | 6.1138554783084700 | 9.72523865194938E-10 | 1.41180866673886E-07 | 6.850224156407390 | up |
| **C0_90D3.tig17949.1** | 4 | 6 | 23 | 1077 | 613 | 458 | 710.6248843109020 | 7.460059183363060 | 1.1354117687272 | 6.570355697233800 | 5.01952086402199E-11 | 1.43245112930624E-08 | 7.8439201858594700 | up |
| **C0_90D1.tig05144.1** | 40 | 3 | 75 | 13919 | 5701 | 2943 | 8438.748656386800 | 9.164666093299480 | 1.3917243513153700 | 6.585115856194970 | 4.54530817574402E-11 | 1.34303800601697E-08 | 7.871911697256900 | up |
| **C0_90D1.tig00430.2** | 48 | 22 | 155 | 10343 | 3276 | 4396 | 6477.599636704840 | 7.8715782545778300 | 1.2020688372213100 | 6.548358971499250 | 5.81727548163224E-11 | 1.56639525045338E-08 | 7.805098642385300 | up |
| **C0_90D3.tig27795.2** | 0 | 0 | 1 | 61 | 22 | 25 | 38.37113393124710 | 8.125091743518190 | 1.9019708129479100 | 4.271932927785020 | 1.93785868812441E-05 | 0.0011850396288971800 | 2.926267126174050 | up |
| **C0_90D2.tig05155.1** | 170 | 101 | 597 | 26920 | 12237 | 14294 | 17775.99197001110 | 7.380820734814900 | 1.1099959595521200 | 6.649412253530190 | 2.94265714893798E-11 | 1.01291112933907E-08 | 7.994428657039290 | up |
| **C0_90D1.tig13328.1** | 17 | 15 | 25 | 1 | 1 | 0 | 7.777975519293710 | -4.199040446726590 | 1.1497672504974500 | -3.6520786662777800 | 0.00026012616798442700 | 0.013786274004495300 | 1.8605530940807900 | down |
| **LP_90D2.tig00454.4** | 6 | 4 | 11 | 1156 | 533 | 350 | 724.8949870003730 | 8.108355741690740 | 1.143441490527280 | 7.091185520958950 | 1.32967947578385E-12 | 9.4460995780738E-10 | 9.02474748052726 | up |
| **LP_90D1.tig00845.3** | 0 | 0 | 2 | 12 | 23 | 7 | 10.457046847140200 | 5.314846393470040 | 1.434958835970670 | 3.7038319568762100 | 0.00021236696527975500 | 0.011399872353256800 | 1.943100011522000 | up |
| **C0_90D3.tig09571.1** | 0 | 5 | 44 | 5721 | 10145 | 5712 | 5110.88781235936 | 9.779876665631990 | 1.4625217359755200 | 6.686995772482450 | 2.27798456777063E-11 | 9.1446656038355E-09 | 8.038832170894430 | up |
| **LP_90D1.tig12228.1** | 0 | 1 | 2 | 56 | 29 | 17 | 35.76367853988280 | 6.566510887584720 | 1.4775158298419800 | 4.444291394351420 | 8.81820210794693E-06 | 0.0005706026166322480 | 3.2436662411150800 | up |
| **LP_90D1.tig00069.18** | 1 | 0 | 1 | 351 | 98 | 130 | 214.72586105949000 | 9.725876593476170 | 1.6271007166613700 | 5.977427515017390 | 2.26688737367985E-09 | 2.87791264333827E-07 | 6.540922392849710 | up |
| **C0_90D3.tig25465.1** | 1 | 1 | 15 | 340 | 191 | 203 | 233.07392890988700 | 6.821128203371060 | 1.3280775651670800 | 5.136091733100650 | 2.80510775180651E-07 | 2.26231262620936E-05 | 4.64544738064128 | up |
| **C0_90D1.tig10318.1** | 21 | 5 | 36 | 3673 | 902 | 1485 | 2253.8599361896900 | 8.18171995417344 | 1.243186229471040 | 6.581250467723300 | 4.66507933824943E-11 | 1.35445507847661E-08 | 7.868235394095740 | up |
| **C0_90D3.tig08916.1** | 13 | 16 | 49 | 1 | 1 | 1 | 10.316902778124600 | -4.041746586876790 | 1.1425874935073600 | -3.5373628801676900 | 0.00040414397614471400 | 0.02050754288677180 | 1.688086371549810 | down |
| **LP_90D3.tig10371.1** | 0 | 3 | 14 | 360 | 126 | 184 | 232.46080797863800 | 6.824073304422620 | 1.480925562070900 | 4.607978604191250 | 4.06602490699068E-06 | 0.00028049691243700800 | 3.552071914867540 | up |
| **LP_90D1.tig01688.2** | 0 | 0 | 2 | 96 | 46 | 60 | 64.83401353930490 | 8.03343766810542 | 1.593534354805120 | 5.04127045889003 | 4.62451329668452E-07 | 3.59925115298367E-05 | 4.443787847554020 | up |
| **LP_90D3.tig00401.3** | 38 | 13 | 176 | 9898 | 5246 | 3583 | 6382.900582506640 | 7.850970884879320 | 1.256433317183200 | 6.248617238581670 | 4.14102333807906E-10 | 6.89710941756983E-08 | 7.161332884193770 | up |
| **C0_90D3.tig08348.1** | 18 | 16 | 37 | 0 | 0 | 3 | 9.224715661120420 | -4.03269482277572 | 1.127244655029830 | -3.5774796578379100 | 0.0003469232087641800 | 0.01787564663183210 | 1.7477382391180900 | down |
| **LP_90D2.tig01204.2** | 0 | 3 | 0 | 181 | 569 | 3 | 168.53809316992000 | 8.814911834063220 | 2.0133553874376300 | 4.378219508122620 | 1.19652806398064E-05 | 0.0007595223484458120 | 3.119459442783550 | up |
| **LP_90D1.tig00817.3** | 7 | 0 | 3 | 480 | 45 | 122 | 275.23869448383400 | 7.711856531039150 | 1.6739154689866200 | 4.6070764467621900 | 4.08369897889578E-06 | 0.00028113530970381700 | 3.5510846049564800 | up |
| **C0_90D1.tig00201.3** | 139 | 39 | 415 | 22984 | 16264 | 9152 | 15473.513813202300 | 7.726938755208290 | 1.1608036341380000 | 6.656542526201000 | 2.80344044854721E-11 | 1.00649541007035E-08 | 7.99718820128766 | up |
| **C0_90D1.tig01695.2** | 31 | 17 | 175 | 9938 | 5094 | 3101 | 6315.217851383010 | 7.866459290845350 | 1.2595381288831800 | 6.245511041273870 | 4.22416691265833E-10 | 6.98221331914599E-08 | 7.156006886843420 | up |
| **C0_90D3.tig04381.2** | 0 | 1 | 3 | 361 | 161 | 121 | 226.99532954464100 | 8.857896641490040 | 1.454323817909810 | 6.090732017454570 | 1.12395559676782E-09 | 1.60375014617439E-07 | 6.794863291033080 | up |
| **C0_90D3.tig00362.2** | 1 | 0 | 3 | 123 | 61 | 30 | 76.86491071416960 | 7.261459076522830 | 1.489304187232080 | 4.875739381367400 | 1.08401650232154E-06 | 8.00757234425089E-05 | 4.096499129021680 | up |
| **LP_90D3.tig00127.8** | 33 | 19 | 61 | 7625 | 2241 | 3064 | 4721.631629803660 | 8.387772388877180 | 1.1308101628303600 | 7.417489393518550 | 1.19361243759095E-13 | 1.66056356994684E-10 | 9.779744494020020 | up |
| **C0_90D3.tig28233.1** | 12 | 11 | 70 | 0 | 1 | 1 | 11.50764301462410 | -4.913962661599340 | 1.439900488594210 | -3.4127099063608800 | 0.0006432037092355060 | 0.031124534271977200 | 1.50689713835801 | down |
| **LP_90D3.tig20872.1** | 4 | 1 | 1 | 3 | 14 | 19 | 6.856139972353010 | 3.0301714415903300 | 0.8669201573701430 | 3.495329317041770 | 0.0004734771733845680 | 0.023666061889427100 | 1.6258740040896200 | up |
| **LP_90D1.tig00136.5** | 8 | 0 | 16 | 1298 | 410 | 528 | 808.92587516273 | 8.07813975277926 | 1.505232842120290 | 5.366704423881900 | 8.0188305521462E-08 | 7.33536255631807E-06 | 5.134578415928800 | up |
| **C0_90D3.tig03362.2** | 45 | 17 | 96 | 7860 | 2350 | 3026 | 4858.55727313608 | 7.949926723321450 | 1.187227425664550 | 6.696212159074290 | 2.13891130873275E-11 | 8.70928166918022E-09 | 8.060017663588910 | up |
| **LP_90D1.tig00113.6** | 2 | 0 | 0 | 349 | 197 | 252 | 243.75723778887200 | 9.865595973214040 | 1.5494751137923200 | 6.367056744182290 | 1.92689903268037E-10 | 3.82959713108125E-08 | 7.416846910874980 | up |
| **C0_90D1.tig00244.2** | 87 | 0 | 25 | 12410 | 3284 | 5417 | 7691.04040369832 | 9.012554864722370 | 1.7525487463944400 | 5.142541617323990 | 2.71046374235363E-07 | 2.19659402653994E-05 | 4.65825020176199 | up |
| **C0_90D3.tig17182.1** | 0 | 0 | 2 | 22 | 38 | 255 | 52.83346247458120 | 7.746872265313840 | 1.5014621052840400 | 5.159552304417520 | 2.47541062324251E-07 | 2.04077741480109E-05 | 4.690204360690670 | up |
| **LP_90D3.tig02381.2** | 0 | 0 | 1 | 10 | 11 | 19 | 9.455737211539460 | 6.027323257430960 | 1.6025194573604000 | 3.7611544931622200 | 0.000169130922076883 | 0.009227307773243540 | 2.034924993424550 | up |
| **LP_90D1.tig03102.3** | 1 | 0 | 0 | 286 | 132 | 136 | 185.86806061673600 | 10.418812998426300 | 1.8539261536997800 | 5.619864080149050 | 1.91107746680842E-08 | 2.01290112111251E-06 | 5.696177558250900 | up |
| **LP_3D2.tig05202.1** | 8 | 0 | 0 | 8823 | 1391 | 2777 | 5184.609428825300 | 12.220760595367200 | 1.9538181218557400 | 6.254809728021120 | 3.98000844491233E-10 | 6.73083423909649E-08 | 7.17193110472362 | up |
| **LP_90D1.tig08372.1** | 0 | 0 | 2 | 373 | 74 | 161 | 227.5186718233430 | 9.864882992831480 | 1.778794367597810 | 5.545825404289770 | 2.9257066299538E-08 | 2.95125131926065E-06 | 5.529993805775990 | up |
| **LP_90D3.tig00132.8** | 0 | 0 | 5 | 27 | 32 | 95 | 32.30318167707580 | 5.706718467063370 | 1.2977465625478100 | 4.397405958725570 | 1.09552325789717E-05 | 0.0006993962917385950 | 3.1552766746886800 | up |
| **LP_90D1.tig00144.6** | 0 | 0 | 10 | 95 | 61 | 137 | 78.11681442157160 | 6.012920979647620 | 1.6188672251836500 | 3.7142768017713700 | 0.00020378564084186300 | 0.010956841806874300 | 1.9603146085798200 | up |
| **LP_90D3.tig02905.3** | 0 | 1 | 0 | 740 | 702 | 174 | 501.7706571597130 | 11.854918028524900 | 1.8733664143546100 | 6.328136309953560 | 2.48139898271425E-10 | 4.6028572574359E-08 | 7.336972493120180 | up |
| **LP_90D2.tig26601.1** | 0 | 0 | 1 | 57 | 25 | 26 | 36.80880377855510 | 8.063337826674710 | 1.8643682803343000 | 4.324970507022830 | 1.52552270863162E-05 | 0.0009432532910833550 | 3.0253716708374400 | up |
| **C0_90D3.tig00962.4** | 8 | 13 | 212 | 5271 | 2891 | 1672 | 3391.0665581342500 | 6.926550257826880 | 1.4485248731062900 | 4.781795871391030 | 1.73736053829215E-06 | 0.00012501881683844100 | 3.9030246155193400 | up |
| **LP_90D1.tig06857.2** | 0 | 3 | 15 | 32293 | 26551 | 10303 | 21753.364090638300 | 13.305316840178300 | 1.4532130935301500 | 9.155792016611280 | 5.39605115045595E-20 | 4.50421879656434E-16 | 15.34638052139100 | up |
| **LP_90D2.tig00768.1** | 9 | 0 | 0 | 522 | 204 | 266 | 338.0027594420100 | 8.106231870238070 | 1.8119690834489400 | 4.473714228505770 | 7.68724651887313E-06 | 0.0005032734784679510 | 3.2981959554731300 | up |
| **C0_90D3.tig17245.1** | 0 | 2 | 5 | 588 | 251 | 148 | 361.3747792339470 | 8.732189387150890 | 1.4545135064424900 | 6.003512066731110 | 1.93094408445315E-09 | 2.53827921400811E-07 | 6.595460606656870 | up |
| **C0_90D1.tig00064.2** | 289 | 62 | 774 | 47395 | 16714 | 22111 | 30192.901422938900 | 7.763427906448510 | 1.237949763511560 | 6.271197858971950 | 3.5828098542631E-10 | 6.23054365749951E-08 | 7.2054740565269 | up |
| **LP_90D3.tig04769.1** | 6 | 4 | 10 | 15 | 24 | 26 | 17.11011926693650 | 2.4661720931217100 | 0.6771244044048680 | 3.6421255489812900 | 0.0002703961329050560 | 0.014240152179127600 | 1.846485369533270 | up |
| **C0_90D3.tig08533.1** | 16 | 40 | 100 | 2 | 2 | 6 | 21.05536055820580 | -3.264652162722660 | 0.9715343840957660 | -3.36030532337892 | 0.0007785637871705030 | 0.03640821609220720 | 1.4388005998436000 | down |
| **C0_90D3.tig00632.1** | 98 | 27 | 160 | 9118 | 1772 | 3249 | 5489.277116687350 | 7.259260550436680 | 1.2569071994266500 | 5.775494446804080 | 7.67274308456489E-09 | 8.68424470679787E-07 | 6.061267947387740 | up |
| **LP_90D1.tig00991.2** | 2 | 5 | 56 | 682 | 482 | 356 | 476.3780112896690 | 5.966306120072830 | 1.3503727105451800 | 4.418266211603230 | 9.94958254431316E-06 | 0.0006425659798299270 | 3.1920822715688900 | up |
| **LP_90D1.tig00422.2** | 0 | 1 | 8 | 389 | 135 | 333 | 268.98026086712700 | 7.954830082836710 | 1.4380244526627800 | 5.531776645457460 | 3.17003402210555E-08 | 3.12190519048394E-06 | 5.5055802902149500 | up |
| **C0_90D3.tig05195.2** | 7 | 2 | 21 | 1721 | 762 | 456 | 1065.6878694559800 | 8.170584143670560 | 1.280636424587670 | 6.380096635390690 | 1.76976273204976E-10 | 3.60323214441089E-08 | 7.443307756809190 | up |
| **LP_90D1.tig14350.1** | 0 | 2 | 3 | 464 | 214 | 219 | 301.6726675464000 | 8.941891755265040 | 1.346639020291360 | 6.64015494911944 | 3.13353564992047E-11 | 1.035897245695E-08 | 7.984683321657260 | up |
| **LP_90D3.tig16531.1** | 63 | 65 | 214 | 3 | 1 | 6 | 44.392127589018500 | -4.257795917608770 | 0.9913741926004420 | -4.2948424009710000 | 1.74817478960161E-05 | 0.0010729744126839700 | 2.969410634569610 | down |
| **LP_90D2.tig00102.8** | 6 | 0 | 20 | 1547 | 1394 | 396 | 1047.0089181403100 | 8.357843754669740 | 1.488364933077370 | 5.615453286304530 | 1.96047617654586E-08 | 2.05843833517892E-06 | 5.686462138670210 | up |
| **LP_90D3.tig00279.5** | 0 | 3 | 0 | 1 | 86 | 1041 | 159.80074886996300 | 8.740799203096120 | 2.152574680121670 | 4.060625298538800 | 4.89414632986028E-05 | 0.002851843836085600 | 2.5448742596698100 | up |
| **LP_90D1.tig00060.9** | 1 | 1 | 11 | 720 | 404 | 280 | 469.4876736690650 | 8.21991745410652 | 1.327008628196350 | 6.194321031114100 | 5.85368102623523E-10 | 9.35160553995062E-08 | 7.0291138204343000 | up |
| **C0_90D3.tig04319.3** | 23 | 57 | 131 | 2 | 4 | 12 | 28.872927539161700 | -2.9718247608114400 | 0.8915927369047070 | -3.3331639411157200 | 0.0008586433140500850 | 0.03965316958896030 | 1.4017220925839900 | down |
| **LP_90D1.tig00060.1** | 0 | 0 | 14 | 384 | 166 | 242 | 258.0110611777110 | 7.272895977908800 | 1.8281297902591000 | 3.9783258369625900 | 6.94022202190993E-05 | 0.003975143618711080 | 2.4006471760170800 | up |
| **LP_90D1.tig00378.3** | 0 | 2 | 0 | 676 | 987 | 109 | 495.86712972463600 | 10.953677316446700 | 1.696887108592790 | 6.4551597221635200 | 1.08104824770959E-10 | 2.40937386050985E-08 | 7.61809580566429 | up |
| **LP_90D3.tig00629.2** | 25 | 13 | 249 | 8756 | 4965 | 3767 | 5782.976716843520 | 7.387588090608420 | 1.3345896417369700 | 5.535475369787410 | 3.1038535216629E-08 | 3.06611139747937E-06 | 5.513412070475860 | up |
| **LP_90D2.tig00017.16** | 0 | 0 | 1 | 21 | 14 | 4 | 13.458884762832200 | 6.57590012975898 | 1.9300302077546600 | 3.407148812146920 | 0.000656453256632975 | 0.031628164192955800 | 1.4999260152928500 | up |
| **C0_90D3.tig00107.6** | 14 | 1 | 46 | 2389 | 1446 | 1043 | 1589.8308025406900 | 7.728320022778670 | 1.3462363737173600 | 5.740685791632940 | 9.42939049990828E-09 | 1.05650308523972E-06 | 5.976129230366870 | up |
| **LP_90D1.tig00148.4** | 10 | 4 | 46 | 3165 | 1308 | 1586 | 2054.5092441791800 | 8.133816509961740 | 1.2348465516847800 | 6.586904663469520 | 4.49090438580446E-11 | 1.34303800601697E-08 | 7.871911697256900 | up |
| **C0_90D1.tig01998.2** | 1 | 1 | 11 | 825 | 244 | 338 | 512.0171669940200 | 8.346704024889570 | 1.3971034708380700 | 5.974291954111810 | 2.31091630709894E-09 | 2.91320332405127E-07 | 6.535629203174620 | up |
| **LP_90D1.tig00056.2** | 0 | 0 | 32 | 570 | 383 | 883 | 476.25829215667800 | 6.968517829810530 | 1.9334205967857200 | 3.604243091956080 | 0.0003130639419176410 | 0.016256441612267700 | 1.7889745114652000 | up |
| **LP_90D1.tig00236.1** | 48 | 13 | 139 | 8537 | 4124 | 2717 | 5401.499557091120 | 7.775215781572690 | 1.2304539689255600 | 6.31898143118843 | 2.63292907951278E-10 | 4.80387262490995E-08 | 7.318408516471490 | up |
| **LP_90D1.tig09643.1** | 0 | 5 | 0 | 89 | 14 | 14 | 50.91198440033950 | 6.331556954899470 | 1.9416907300343300 | 3.2608472899221900 | 0.0011107984643994100 | 0.04991716006437690 | 1.3017501309268800 | up |
| **C0_90D3.tig24440.1** | 0 | 0 | 15 | 1259 | 1478 | 1205 | 1020.8104916098700 | 9.164654346978580 | 1.6983874824228400 | 5.396091552620670 | 6.81082031955284E-08 | 6.29934846674653E-06 | 5.200704366725660 | up |
| **C0_90D3.tig00369.5** | 18 | 7 | 59 | 4054 | 2070 | 1367 | 2588.9082342121400 | 7.970131129818170 | 1.1986059430362000 | 6.649500760549320 | 2.94088828880398E-11 | 1.01291112933907E-08 | 7.994428657039290 | up |
| **LP_90D3.tig00646.5** | 0 | 0 | 2 | 31 | 57 | 178 | 49.04327927959220 | 7.622935922088670 | 1.304183944577490 | 5.844985252105840 | 5.06613147675755E-09 | 5.97714006634126E-07 | 6.223506566932530 | up |
| **LP_90D1.tig01494.3** | 3 | 0 | 0 | 740 | 348 | 182 | 457.67628469013500 | 10.163135409256600 | 1.7131430174969800 | 5.932450067190320 | 2.98447169454804E-09 | 3.70440614904329E-07 | 6.431281403572600 | up |
| **C0_90D1.tig02339.2** | 1 | 1 | 14 | 551 | 259 | 161 | 345.98790453885800 | 7.48367903795135 | 1.4168589020675600 | 5.281880240178280 | 1.278647750074E-07 | 1.10317234437263E-05 | 4.957356634102790 | up |
| **LP_90D1.tig00549.6** | 0 | 0 | 1 | 184 | 108 | 103 | 124.7779806073450 | 9.84133887470618 | 1.803656569077520 | 5.4563263558203400 | 4.8608616973543E-08 | 4.5977141986675E-06 | 5.337458028673390 | up |
| **LP_90D3.tig05078.1** | 0 | 0 | 2 | 155 | 186 | 130 | 123.60645392058100 | 8.961161180826920 | 1.4071075769806500 | 6.368497567226260 | 1.90888799896181E-10 | 3.81651864654705E-08 | 7.418332611423950 | up |
| **C0_90D3.tig11498.1** | 9 | 16 | 51 | 0 | 1 | 1 | 9.48091629764606 | -4.625204536624570 | 1.3481302382102100 | -3.4308291628893500 | 0.0006017394305670960 | 0.02945964493725040 | 1.5307724918016700 | down |
| **LP_90D1.tig00198.1** | 2 | 0 | 3 | 733 | 306 | 186 | 449.4017987943840 | 9.481342129077030 | 1.4575226158019700 | 6.505108069187740 | 7.76375461005917E-11 | 1.94905265169373E-08 | 7.710176428696030 | up |
| **C0_3D1.tig01699.2** | 4 | 2 | 79 | 1053 | 730 | 721 | 756.5704908576270 | 6.207159349115600 | 1.426394521361530 | 4.351642730084720 | 1.35121314585632E-05 | 0.0008448624667976910 | 3.073213983088440 | up |
| **LP_90D1.tig00056.15** | 6 | 0 | 5 | 629 | 232 | 136 | 379.22642285546000 | 8.068370858499180 | 1.4954508361438200 | 5.395276570445030 | 6.84180825306085E-08 | 6.31052861219472E-06 | 5.199934259813920 | up |
| **C0_3D2.tig24015.1** | 535 | 457 | 1174 | 27 | 150 | 83 | 313.1361554912200 | -2.564143925431000 | 0.5288231205492730 | -4.848774241882060 | 1.2422670146235E-06 | 9.09606433141757E-05 | 4.041146476785490 | down |
| **LP_90D2.tig00187.1** | 0 | 0 | 3 | 135 | 57 | 57 | 86.3184386444179 | 7.880471155136160 | 1.6254338340307200 | 4.848226356648610 | 1.2457021367165E-06 | 9.1012579087149E-05 | 4.040898578551550 | up |
| **LP_90D1.tig00060.8** | 1 | 0 | 9 | 265 | 112 | 65 | 163.29768917279400 | 7.068467019567200 | 1.580334945944750 | 4.472765117107210 | 7.72145775841266E-06 | 0.0005035385802649230 | 3.2979672489350500 | up |
| **C0_90D3.tig01450.3** | 0 | 0 | 9 | 212 | 134 | 73 | 139.43168213873300 | 7.013523037970100 | 1.7492223310625800 | 4.009509204990350 | 6.08450812775467E-05 | 0.003508733020338530 | 2.4548496760151100 | up |
| **LP_90D3.tig18124.1** | 10 | 14 | 8 | 6 | 37 | 37 | 17.191987130693500 | 1.7592683754237900 | 0.515795156390701 | 3.410788863808550 | 0.0006477522963651460 | 0.03126010669214810 | 1.505009544047730 | up |
| **C0_90D3.tig28181.2** | 1 | 0 | 4 | 188 | 65 | 51 | 114.44355536594000 | 7.529235340065850 | 1.5188778460215900 | 4.957103930238530 | 7.15517064277764E-07 | 5.45442905460508E-05 | 4.263250702712970 | up |
| **C0_90D1.tig01687.2** | 183 | 3 | 69 | 20265 | 2952 | 5367 | 11764.403170465200 | 8.448754275650690 | 1.5851987807898500 | 5.329775910779430 | 9.83340234773765E-08 | 8.87371543212466E-06 | 5.05189450262415 | up |
| **LP_90D1.tig04192.2** | 3 | 0 | 1 | 99 | 21 | 18 | 57.515818438506200 | 6.764249551588840 | 1.6429527432621100 | 4.117129710108580 | 3.83620189256627E-05 | 0.002279127135069310 | 2.64223144817678 | up |
| **C0_90D2.tig05971.1** | 0 | 15 | 127 | 1324 | 942 | 641 | 920.7759671871750 | 5.749477909497360 | 1.7163830769898900 | 3.3497638065626500 | 0.0008088049616980660 | 0.037664140678015000 | 1.4240719367155000 | up |
| **LP_90D1.tig00092.8** | 7 | 2 | 10 | 494 | 169 | 214 | 312.63518090756400 | 7.021359876579230 | 1.188511408445950 | 5.907692451820980 | 3.46932923570699E-09 | 4.2744440535432E-07 | 6.369120362849080 | up |
| **LP_90D2.tig01352.1** | 2 | 0 | 1 | 30 | 15 | 12 | 19.700713726697600 | 5.610323422806240 | 1.4459491815466900 | 3.8800280773388200 | 0.00010444440747983300 | 0.005792847709874000 | 2.2371078886648900 | up |
| **C0_90D1.tig19779.1** | 214 | 62 | 542 | 32617 | 16059 | 9335 | 20534.70304702910 | 7.66394934965056 | 1.2112949229316100 | 6.3270713057246800 | 2.49857914250844E-10 | 4.60911928117206E-08 | 7.336382052455980 | up |
| **LP_90D2.tig08060.2** | 0 | 0 | 5 | 40 | 23 | 26 | 28.138991594841600 | 5.502043800374060 | 1.5502554802827200 | 3.5491206903333500 | 0.0003865198807449890 | 0.019673037039930500 | 1.7061285903669100 | up |
| **LP_90D2.tig02156.1** | 1 | 0 | 8 | 896 | 438 | 508 | 597.7295260933800 | 9.097185331226540 | 1.4355449699962600 | 6.337095334080870 | 2.34137130853415E-10 | 4.51884662547091E-08 | 7.344972398841850 | up |
| **C0_3D3.tig14076.1** | 3 | 0 | 4 | 21 | 22 | 9 | 15.961855816476700 | 4.04934270793935 | 1.2374074697964500 | 3.2724408141850400 | 0.0010662319359385000 | 0.04817377281332980 | 1.3171893398211900 | up |
| **LP_90D3.tig14892.1** | 8 | 1 | 14 | 2730 | 965 | 1183 | 1721.4622095109300 | 9.226007894885120 | 1.272292391231970 | 7.251483981564590 | 4.12229785863133E-13 | 4.17089100611641E-10 | 9.379771159005930 | up |
| **LP_90D1.tig01512.2** | 5 | 3 | 15 | 470 | 213 | 268 | 313.8588887269810 | 6.7772109275196800 | 1.1155524025079200 | 6.075206249642390 | 1.23828461271922E-09 | 1.74451835164903E-07 | 6.758324457584180 | up |
| **LP_90D2.tig23991.1** | 0 | 0 | 3 | 177 | 178 | 38 | 121.12456100805700 | 8.373648357064060 | 1.62859311135895 | 5.141645447632300 | 2.72342696998298E-07 | 2.20175552302086E-05 | 4.657230905572020 | up |
| **LP_90D1.tig03220.1** | 0 | 0 | 2 | 22 | 15 | 89 | 26.29976584472880 | 6.7188318917595400 | 1.4665273004379000 | 4.581457085560780 | 4.61747367014052E-06 | 0.000316576649635158 | 3.4995211214022300 | up |
| **C0_90D3.tig08409.2** | 1 | 0 | 1 | 143 | 46 | 56 | 88.8442145681342 | 8.44530960449772 | 1.6023717444632100 | 5.270505819688470 | 1.3604831198641E-07 | 1.15880537982506E-05 | 4.935989497224820 | up |
| **LP_90D1.tig03561.2** | 6 | 0 | 3 | 2746 | 1537 | 1323 | 1821.5642539329700 | 10.609195906987200 | 1.338181688310630 | 7.928068362959470 | 2.22580779516271E-15 | 6.20646079847234E-12 | 11.20715598337570 | up |
| **LP_90D1.tig00338.7** | 0 | 0 | 3 | 977 | 749 | 448 | 670.7968126991230 | 10.845407742804800 | 1.5209651481160300 | 7.130608979593440 | 9.99258213000432E-13 | 8.20476819488728E-10 | 9.085933684337080 | up |
| **LP_90D3.tig01403.4** | 1 | 0 | 1 | 577 | 237 | 269 | 370.43136082460300 | 10.515375832842500 | 1.551164167138350 | 6.77902188279768 | 1.20992202785094E-11 | 5.3155377089362E-09 | 8.274452796575860 | up |
| **LP_90D1.tig01767.2** | 0 | 2 | 0 | 5246 | 2954 | 2297 | 3447.486313106430 | 13.751802797091700 | 1.6213059635584000 | 8.481929448350190 | 2.21489864590311E-17 | 1.04178378165764E-13 | 12.982222407885700 | up |
| **C0_90D1.tig19190.1** | 12 | 6 | 46 | 3731 | 2270 | 1279 | 2430.8754810957600 | 8.277349687317730 | 1.177465829135200 | 7.029800341125080 | 2.06829285990079E-12 | 1.32804289036976E-09 | 8.87678789880074 | up |
| **C0_90D1.tig06202.1** | 118 | 26 | 214 | 17249 | 5945 | 7678 | 10912.513636532700 | 7.929882676327450 | 1.1928550519966900 | 6.64781748885067 | 2.97470837973085E-11 | 1.01349528664116E-08 | 7.994178266695190 | up |
| **C0_90D2.tig00173.1** | 94 | 151 | 386 | 5 | 11 | 48 | 87.80320659390630 | -2.8069305792033400 | 0.8576377185142640 | -3.2728627934717600 | 0.0010646414492956700 | 0.04817377281332980 | 1.3171893398211900 | down |
| **C0_90D1.tig00064.20** | 101 | 51 | 375 | 23595 | 8249 | 10313 | 14918.648196075000 | 7.852611618154830 | 1.1959081363256100 | 6.566233124127550 | 5.16040055763636E-11 | 1.46017469677051E-08 | 7.835595181678110 | up |
| **LP_90D3.tig02667.1** | 4 | 0 | 0 | 773 | 927 | 131 | 542.2629150160600 | 9.983429645522190 | 1.6984438101942100 | 5.877986416507120 | 4.15286986806562E-09 | 5.06059021988478E-07 | 6.295798828162070 | up |
| **C0_90D3.tig05701.1** | 17 | 11 | 34 | 1 | 0 | 2 | 8.496700096195860 | -3.716669526141030 | 1.1365562928849200 | -3.2701147751397400 | 0.0010750385562681900 | 0.048506030209781900 | 1.3142042670876800 | down |
| **C0_90D1.tig03430.2** | 100 | 1 | 86 | 21117 | 5569 | 7786 | 12880.024788921600 | 9.068419620192020 | 1.5460394676201400 | 5.8655809312237700 | 4.47562893581785E-09 | 5.39482940570477E-07 | 6.268022283943530 | up |
| **LP_90D3.tig13665.1** | 0 | 0 | 3 | 44 | 18 | 77 | 36.594352277511800 | 6.621541499650930 | 1.4788741142396700 | 4.477420651219710 | 7.55502941108019E-06 | 0.0004965647185168430 | 3.304024140797920 | up |
| **LP_90D3.tig16663.1** | 1362 | 1238 | 2766 | 43 | 246 | 472 | 786.3233786739200 | -2.450266018323440 | 0.6206428903104690 | -3.9479482590990900 | 7.882383441423E-05 | 0.004468334477515660 | 2.3498543251933000 | down |
| **LP_90D1.tig00175.5** | 1 | 0 | 4 | 636 | 266 | 153 | 388.8670246146310 | 9.303260856994950 | 1.5111538167594000 | 6.156395698318370 | 7.44191469877226E-10 | 1.13299940524344E-07 | 6.94577031811503 | up |
| **C0_3D1.tig08250.1** | 2 | 1 | 0 | 93 | 12 | 19 | 53.23444429106610 | 7.070396223840990 | 1.707197041721670 | 4.141523240170710 | 3.45006907235615E-05 | 0.002057042076016060 | 2.6867568248853900 | up |
| **LP_90D2.tig05800.1** | 0 | 2 | 38 | 7504 | 4382 | 5441 | 5261.9635814611400 | 10.118862062986000 | 1.6417706568254800 | 6.163383430516310 | 7.12068029542235E-10 | 1.0956200118133E-07 | 6.960340043832540 | up |
| **LP_90D1.tig02175.1** | 2 | 0 | 0 | 504 | 88 | 167 | 298.66106049892200 | 10.14309076168850 | 1.831876282211630 | 5.536995516663750 | 3.07704425056724E-08 | 3.05772114530327E-06 | 5.514602123429280 | up |
| **LP_90D1.tig12266.2** | 0 | 3 | 11 | 235 | 75 | 90 | 146.8841702828470 | 6.4321808669406 | 1.48906823260238 | 4.319601161391610 | 1.56311443494089E-05 | 0.0009647103117974370 | 3.015603079273190 | up |
| **C0_90D1.tig06316.2** | 21 | 9 | 57 | 4895 | 2313 | 1319 | 3053.204821998420 | 8.150811734018080 | 1.1976671327532100 | 6.805573527997630 | 1.00647457495919E-11 | 4.72882307012682E-09 | 8.325246934896620 | up |
| **C0_90D3.tig18597.1** | 46 | 39 | 36 | 22 | 155 | 136 | 66.1084971750323 | 1.7535655901099400 | 0.4133305141691700 | 4.24252633182615 | 2.21017599277346E-05 | 0.0013393024722815400 | 2.8731213294793400 | up |
| **LP_90D1.tig00040.1** | 0 | 6 | 12 | 941 | 502 | 262 | 595.6096516632100 | 8.097415622553970 | 1.444828191031880 | 5.60441419458383 | 2.08960474794743E-08 | 2.15338928793879E-06 | 5.666877451680930 | up |
| **LP_90D3.tig03708.1** | 2 | 0 | 8 | 669 | 131 | 243 | 402.1414855261470 | 8.357149784052130 | 1.5557105074336300 | 5.371918325497770 | 7.79033950229301E-08 | 7.15070026566675E-06 | 5.1456514258040700 | up |
| **LP_90D1.tig02322.1** | 0 | 0 | 1 | 41 | 34 | 162 | 48.921148068032400 | 8.488590112587360 | 1.6763676911923800 | 5.063680335278660 | 4.11238999173097E-07 | 3.2307903396213E-05 | 4.49069122437169 | up |
| **LP_3D3.tig14660.1** | 1 | 1 | 1 | 103 | 21 | 30 | 61.15965683258320 | 7.316753979813990 | 1.5080826493392400 | 4.851692964586400 | 1.22412028627484E-06 | 8.98289060185286E-05 | 4.046583889342780 | up |
| **LP_90D1.tig00055.13** | 2 | 1 | 7 | 309 | 143 | 99 | 195.1295149362420 | 7.297357276513150 | 1.2737299122298100 | 5.729124523532860 | 1.00950272142934E-08 | 1.12354287886014E-06 | 5.949410348525670 | up |
| **LP_90D1.tig00370.4** | 0 | 0 | 4 | 317 | 118 | 138 | 200.8834070274740 | 8.7000391800124 | 1.6496487225721700 | 5.273873801718890 | 1.33573775918837E-07 | 1.1406380573284E-05 | 4.9428521422967800 | up |
| **C0_90D3.tig00777.3** | 13 | 2 | 36 | 2423 | 987 | 1162 | 1564.2890994404100 | 7.9565586655284 | 1.2686712620026400 | 6.271568454201990 | 3.57429091688517E-10 | 6.23054365749951E-08 | 7.2054740565269 | up |
| **LP_90D1.tig03441.1** | 0 | 0 | 1 | 35 | 11 | 21 | 22.802941317613500 | 7.358748572003000 | 1.8772543064124400 | 3.919952958353340 | 8.85662544947089E-05 | 0.004969980960208130 | 2.303645275027710 | up |
| **C0_90D3.tig00604.2** | 0 | 0 | 13 | 244 | 184 | 98 | 166.60260019916000 | 6.743060273778440 | 1.7992100705106000 | 3.7477893128204000 | 0.00017840000646563 | 0.009660240951254550 | 2.015012041028070 | up |
| **LP_90D1.tig00105.1** | 5 | 6 | 21 | 1882 | 605 | 837 | 1183.9595328796100 | 8.237255551408960 | 1.1746356031577000 | 7.0126050404612500 | 2.33921113826819E-12 | 1.44636890177105E-09 | 8.839720924489300 | up |
| **LP_90D1.tig01866.1** | 0 | 2 | 2 | 371 | 118 | 182 | 235.36126176020300 | 8.898185747088620 | 1.4191017160315900 | 6.2702945437707700 | 3.60365764937725E-10 | 6.23432773342264E-08 | 7.205210370959440 | up |
| **LP_90D1.tig00056.7** | 0 | 0 | 2 | 157 | 61 | 40 | 95.79633006591510 | 8.609992819767410 | 1.7531350130416100 | 4.91119780035049 | 9.05217154883626E-07 | 6.79197653582233E-05 | 4.168003823114590 | up |
| **LP_90D1.tig00056.8** | 0 | 0 | 1 | 65 | 31 | 23 | 41.33443171206650 | 8.233749783896620 | 1.8856378316173100 | 4.366559498243930 | 1.26218917206412E-05 | 0.0007981673156448650 | 3.097906060222390 | up |
| **LP_90D2.tig08125.1** | 3 | 2 | 2 | 10 | 12 | 10 | 9.082574531281080 | 3.0660261879018000 | 0.919586595148565 | 3.334135364822780 | 0.0008556500579987180 | 0.0396245489410807 | 1.4020356684591000 | up |
| **C0_90D2.tig05326.1** | 454 | 127 | 731 | 57768 | 20950 | 25860 | 36715.81300978840 | 7.800703524220520 | 1.1416980776389000 | 6.832545028325570 | 8.34212084929124E-12 | 4.22022837934826E-09 | 8.374664046382640 | up |
| **LP_90D1.tig04612.1** | 1 | 1 | 3 | 151 | 76 | 56 | 97.24078372201950 | 7.2755642931269300 | 1.2826868931518600 | 5.672128039953070 | 1.41034500082748E-08 | 1.51903255589125E-06 | 5.8184329182422800 | up |
| **C0_90D3.tig18448.1** | 15 | 17 | 71 | 0 | 1 | 3 | 13.063094632413200 | -4.190348972063720 | 1.1914252532862100 | -3.5170892680894900 | 0.00043630702074272000 | 0.021966134953540600 | 1.658246352547720 | down |
| **LP_90D1.tig00060.7** | 4 | 0 | 0 | 119 | 59 | 35 | 75.28650394661180 | 7.120092071661810 | 1.700160667573210 | 4.187893654677340 | 2.81555383536071E-05 | 0.0016969048196544900 | 2.770342516820870 | up |
| **C0_90D3.tig01000.4** | 144 | 69 | 413 | 28679 | 9271 | 12651 | 18051.231408535300 | 7.869555887251120 | 1.169431791364440 | 6.729384257690860 | 1.70382554314085E-11 | 7.20114317214304E-09 | 8.142598554409810 | up |
| **LP_90D1.tig09940.1** | 0 | 0 | 27 | 275 | 271 | 1333 | 371.03558629419200 | 6.851873719503210 | 1.8766979304091500 | 3.6510264163873200 | 0.00026119437028099600 | 0.013820949016342600 | 1.8594621350797500 | up |
| **LP_90D1.tig00914.3** | 0 | 2 | 0 | 1531 | 211 | 516 | 900.6512202677310 | 11.816889134735900 | 1.8605272602606900 | 6.351365759123680 | 2.13411604463627E-10 | 4.15745548591553E-08 | 7.381172392096360 | up |
| **C0_90D3.tig05818.2** | 13 | 3 | 44 | 1851 | 427 | 612 | 1116.34791031074 | 7.239607960962700 | 1.3585315026098200 | 5.3289952769258500 | 9.87575646459069E-08 | 8.887914625235E-06 | 5.051200125755690 | up |
| **C0_90D1.tig03937.2** | 48 | 7 | 129 | 8258 | 2570 | 3370 | 5146.721893773840 | 7.823240291323150 | 1.315697085052210 | 5.946080127564270 | 2.74639746630258E-09 | 3.4216218284469E-07 | 6.46576799213405 | up |
| **LP_90D1.tig00343.2** | 0 | 1 | 0 | 1075 | 1102 | 188 | 730.2383774054120 | 12.3968433233037 | 1.9102276041440600 | 6.48972054241594 | 8.59957367506573E-11 | 2.09584792289613E-08 | 7.67864023344555 | up |
| **LP_90D1.tig00319.3** | 0 | 0 | 6 | 82 | 59 | 50 | 58.24026291898300 | 6.316117590465690 | 1.5581515303174900 | 4.053596500449940 | 5.04361805324456E-05 | 0.002928719359648390 | 2.5333222419733800 | up |
| **C0_90D3.tig05157.1** | 19 | 26 | 79 | 1 | 2 | 5 | 16.6010116365043 | -3.372618954999280 | 0.9333205863840040 | -3.613569661059260 | 0.0003020101657020900 | 0.015774588320828900 | 1.802041965987180 | down |
| **C0_90D3.tig15854.1** | 1 | 0 | 4 | 248 | 155 | 67 | 159.63998597831300 | 8.011836053274200 | 1.444815908597840 | 5.5452296763879900 | 2.9356856083914E-08 | 2.95239779453556E-06 | 5.529825127766100 | up |
| **C0_90D3.tig00040.8** | 57 | 24 | 312 | 18465 | 10438 | 5838 | 11867.208802702100 | 7.959506750161950 | 1.2713940576258600 | 6.260456152379010 | 3.83852994701319E-10 | 6.57254750773453E-08 | 7.1822662660398000 | up |
| **C0_3D1.tig04314.1** | 1 | 0 | 0 | 41 | 9 | 8 | 23.854174122995200 | 7.428726058003720 | 2.0495001019116700 | 3.624652690221680 | 0.0002893500313805180 | 0.015142802817812100 | 1.8197937327850300 | up |
| **LP_90D3.tig14090.1** | 632 | 328 | 1340 | 97308 | 38602 | 47489 | 62839.00071125610 | 7.778901126256080 | 1.076510206925520 | 7.226035643890800 | 4.97297273683416E-13 | 4.74407390600445E-10 | 9.323848553893390 | up |
| **C0_90D1.tig03423.2** | 568 | 259 | 1129 | 98881 | 33667 | 42146 | 62225.410875002100 | 7.996565223098000 | 1.1221910182603200 | 7.125850316904770 | 1.03440082755086E-12 | 8.22324029311803E-10 | 9.08495701894059 | up |
| **C0_90D3.tig05755.1** | 28 | 23 | 84 | 2 | 2 | 5 | 18.574594539387100 | -3.2264235241467000 | 0.9037001646374190 | -3.5702367338189000 | 0.0003566587521176680 | 0.018320737037625800 | 1.73705705878306 | down |
| **LP_90D1.tig11156.1** | 0 | 0 | 1 | 141 | 289 | 9 | 112.23241291780200 | 9.69146530758379 | 2.02600137498856 | 4.783543302204580 | 1.72231766411058E-06 | 0.00012420402696973700 | 3.9058643231418600 | up |
| **LP_90D1.tig00052.11** | 1 | 0 | 1 | 276 | 116 | 110 | 175.01037695696200 | 9.42993677525882 | 1.5669529333662300 | 6.018008948744130 | 1.76575434842292E-09 | 2.34887537607541E-07 | 6.6291400249076 | up |
| **C0_90D3.tig00040.9** | 78 | 35 | 298 | 17805 | 9057 | 9262 | 11833.660451015000 | 7.878326649000540 | 1.1487871497454800 | 6.857951580278410 | 6.98549256606226E-12 | 3.64435330137895E-09 | 8.438379527033390 | up |
| **LP_90D1.tig01689.2** | 7 | 1 | 12 | 1123 | 324 | 424 | 691.171278120537 | 8.106709765903160 | 1.3036130344044000 | 6.218647368470800 | 5.01458842745186E-10 | 8.18539353097926E-08 | 7.086960436078030 | up |
| **LP_90D1.tig01859.1** | 47 | 14 | 45 | 8859 | 3488 | 1708 | 5333.604810169790 | 8.625963361370010 | 1.2121758389969700 | 7.1160990706659 | 1.11024504739153E-12 | 8.51223099612214E-10 | 9.069956599454570 | up |
| **C0_90D3.tig01226.1** | 62 | 12 | 77 | 5885 | 1850 | 2435 | 3677.818593070440 | 7.585988107236700 | 1.1980102170402500 | 6.332156436844360 | 2.41758177931434E-10 | 4.58639988804127E-08 | 7.338528081834960 | up |
| **LP_90D2.tig20552.1** | 4 | 1 | 1 | 4 | 14 | 11 | 6.242459202988390 | 2.798754886286390 | 0.846944264894345 | 3.3045325439868600 | 0.0009513493002351830 | 0.043513153130893900 | 1.361379444913880 | up |
| **C0_3D2.tig00886.1** | 1 | 0 | 4 | 921 | 157 | 307 | 545.6434198424410 | 9.794365379069650 | 1.5877971786731100 | 6.168524236360350 | 6.89302859096572E-10 | 1.06551542418405E-07 | 6.972440259145900 | up |
| **LP_90D2.tig00025.3** | 0 | 1 | 0 | 571 | 603 | 119 | 392.9226285463060 | 11.501761388851800 | 1.8829464122993900 | 6.10838487687295 | 1.00644415843023E-09 | 1.44845534507875E-07 | 6.839094889265760 | up |
| **LP_90D3.tig14563.2** | 2 | 0 | 4 | 201 | 98 | 57 | 126.46893406857600 | 7.389164503442800 | 1.419513845126360 | 5.205419115010490 | 1.93559244137445E-07 | 1.6197367424825E-05 | 4.790555566060990 | up |
| **C0_3D1.tig04495.2** | 3 | 2 | 3 | 252 | 434 | 34 | 193.32202211505000 | 7.570166834438510 | 1.225778980279020 | 6.175800822359770 | 6.58289347280506E-10 | 1.02230804727204E-07 | 6.990418220568970 | up |
| **LP_90D3.tig16027.3** | 0 | 0 | 2 | 59 | 31 | 12 | 36.754564954792700 | 7.211906491140850 | 1.748309340341290 | 4.125074621939050 | 3.7061457373336E-05 | 0.0022057843141502900 | 2.6564369559728400 | up |
| **LP_90D2.tig01354.4** | 0 | 2 | 20 | 1442 | 1925 | 1543 | 1222.7493421132900 | 8.865361010751250 | 1.3701700851737600 | 6.470263149576040 | 9.78323899878479E-11 | 2.33272688931139E-08 | 7.632136104501610 | up |
| **LP_90D3.tig24539.1** | 4 | 1 | 14 | 800 | 247 | 338 | 500.10394241462800 | 7.739700944315700 | 1.3136606394002000 | 5.891704989996180 | 3.82231113230493E-09 | 4.6748405273454E-07 | 6.330233199609690 | up |
| **C0_90D1.tig25808.1** | 59 | 25 | 210 | 10334 | 6173 | 3097 | 6669.256099294520 | 7.530044572893830 | 1.1939597136406300 | 6.306782789122080 | 2.84895206828499E-10 | 5.14181949232257E-08 | 7.288883173679990 | up |
| **LP_90D1.tig00056.1** | 0 | 0 | 11 | 115 | 157 | 393 | 137.38747815767700 | 6.701096802907270 | 1.5338042368484600 | 4.368938774531060 | 1.24851760229276E-05 | 0.0007910200042306080 | 3.1018125334212700 | up |
| **LP_90D3.tig02381.4** | 0 | 0 | 2 | 19 | 18 | 36 | 17.590565449163700 | 6.093524451099970 | 1.3511698684791600 | 4.509813749738730 | 6.48845664426223E-06 | 0.0004324213151602230 | 3.364092906697660 | up |
| **LP_90D1.tig04641.1** | 0 | 2 | 0 | 341 | 791 | 99 | 294.1984021776180 | 10.198283216595000 | 1.5789172807317600 | 6.459035784235970 | 1.05372196262203E-10 | 2.39841930998662E-08 | 7.6200748879202100 | up |
| **C0_90D1.tig00095.3** | 1460 | 618 | 6392 | 271527 | 154073 | 140428 | 182663.7275393070 | 7.464700106420090 | 1.1691239619446000 | 6.384866232665440 | 1.71547357686723E-10 | 3.53567575666789E-08 | 7.451527569322610 | up |
| **C0_90D3.tig06280.1** | 6 | 22 | 62 | 2200 | 643 | 991 | 1383.8183067693100 | 6.983004868004790 | 1.2408730077578900 | 5.627493566503040 | 1.82846980461764E-08 | 1.93198665526514E-06 | 5.713995877695230 | up |
| **C0_90D3.tig00040.3** | 9 | 14 | 49 | 3291 | 2101 | 1401 | 2197.4917535205400 | 7.967315397338920 | 1.0935689053720500 | 7.285608943524500 | 3.20220583267234E-13 | 3.4489822757128E-10 | 9.462309037462100 | up |
| **C0_90D3.tig03114.3** | 0 | 5 | 10 | 388 | 189 | 266 | 266.6155341260810 | 7.1916196012124 | 1.3153601451666900 | 5.467414857929280 | 4.566464296479E-08 | 4.34386542436288E-06 | 5.362123637922420 | up |
| **C0_90D3.tig00137.3** | 14 | 5 | 27 | 2618 | 1388 | 894 | 1679.1306255143100 | 8.192671269801440 | 1.1376750924501200 | 7.201239900715030 | 5.96674588507374E-13 | 5.53399106546464E-10 | 9.256961546355630 | up |
| **C0_90D1.tig19962.1** | 57 | 29 | 165 | 11449 | 5217 | 3117 | 7126.717832004870 | 7.847131692954060 | 1.184249746749460 | 6.626247305091620 | 3.44328121701916E-11 | 1.11619142286459E-08 | 7.952261319050040 | up |
| **C0_90D3.tig00856.4** | 1 | 1 | 15 | 856 | 357 | 200 | 523.6160598881320 | 7.999160628232480 | 1.4651687408539400 | 5.459549064342150 | 4.77345411899445E-08 | 4.52786532895186E-06 | 5.344106498728540 | up |
| **LP_90D1.tig00171.9** | 0 | 1 | 3 | 104 | 72 | 48 | 70.894366978067 | 7.1601507574590800 | 1.346100017474550 | 5.319181832336940 | 1.04234922304678E-07 | 9.25611654476302E-06 | 5.033571185773160 | up |
| **LP_90D1.tig04655.3** | 0 | 0 | 8 | 306 | 131 | 129 | 195.99926055205200 | 7.677745863984200 | 1.7437446394215500 | 4.403021916403500 | 1.06753376952217E-05 | 0.0006854593275110690 | 3.164018309454410 | up |
| **C0_90D1.tig02899.3** | 364 | 202 | 1143 | 58468 | 20027 | 29935 | 37587.031238211400 | 7.4803410804889800 | 1.141033315067580 | 6.555760451258990 | 5.53590363488392E-11 | 1.52758914433999E-08 | 7.815993436558210 | up |
| **C0_90D3.tig00294.1** | 73 | 143 | 283 | 6 | 12 | 28 | 69.63362798744600 | -2.8327764895248500 | 0.7120567788733290 | -3.978301412995580 | 6.94093482796297E-05 | 0.003975143618711080 | 2.4006471760170800 | down |
| **LP_90D1.tig00493.2** | 0 | 0 | 8 | 243 | 281 | 200 | 192.45137103426100 | 7.6469844545427200 | 1.5235380895917900 | 5.019227616811110 | 5.18796493308555E-07 | 4.01904782252421E-05 | 4.3958768261207800 | up |
| **C0_90D2.tig24584.1** | 15 | 13 | 16 | 0 | 2 | 2 | 6.088154857354480 | -2.9738852753686900 | 0.8868212890291160 | -3.3534211595489200 | 0.0007981914996334500 | 0.03727386850526050 | 1.4285955312252100 | down |
| **LP_90D3.tig07598.2** | 1 | 0 | 0 | 49 | 7 | 2 | 26.925062157982600 | 7.612349242521160 | 2.2715452462659800 | 3.351176585645620 | 0.0008046897171754100 | 0.03752483933906390 | 1.425681158493280 | up |
| **LP_90D3.tig06844.3** | 0 | 1 | 0 | 456 | 237 | 138 | 288.4693175268030 | 11.054938055614800 | 1.8966772675114400 | 5.82858151198259 | 5.59004908929329E-09 | 6.55338232527148E-07 | 6.183534494501950 | up |
| **LP_90D1.tig04527.2** | 0 | 2 | 0 | 107 | 144 | 20 | 77.51418755047860 | 8.26646920797084 | 1.6747104251582200 | 4.936058845629910 | 7.97170635550498E-07 | 6.02188469465963E-05 | 4.220267564491220 | up |
| **LP_90D1.tig15355.1** | 3 | 0 | 1 | 177 | 47 | 100 | 113.26212681414500 | 7.757789754739980 | 1.474283375524540 | 5.262075041699370 | 1.42438604115296E-07 | 1.21014823226606E-05 | 4.917161429260190 | up |
| **C0_90D1.tig00696.2** | 28 | 4 | 99 | 4296 | 2364 | 1548 | 2784.5728780559100 | 7.436159284256820 | 1.312508234384300 | 5.665609623961810 | 1.46502724196322E-08 | 1.57285513125112E-06 | 5.803311276483890 | up |
| **C0_90D3.tig03838.1** | 0 | 1 | 11 | 4998 | 1806 | 2378 | 3182.960481038650 | 11.119419548329000 | 1.5479491242016200 | 7.183323647063650 | 6.80367803555046E-13 | 6.13967583591877E-10 | 9.211854558238020 | up |
| **LP_90D1.tig01889.1** | 1 | 0 | 1 | 246 | 117 | 81 | 155.34442699420900 | 9.257094064693090 | 1.5759347399406000 | 5.874033886099870 | 4.25316374470189E-09 | 5.16395942806732E-07 | 6.287017178534920 | up |
| **LP_90D1.tig00128.1** | 8 | 0 | 19 | 1224 | 670 | 600 | 814.2690541756090 | 7.924968141636060 | 1.435139243783040 | 5.522090052213880 | 3.34990711684691E-08 | 3.27046926094741E-06 | 5.485389928434980 | up |
| **C0_90D3.tig01577.1** | 41 | 14 | 63 | 5013 | 1523 | 2010 | 3115.4894955535600 | 7.7141021190227 | 1.1598733164831400 | 6.6508143686007700 | 2.9147571528898E-11 | 1.01291112933907E-08 | 7.994428657039290 | up |
| **C0_90D3.tig24138.1** | 0 | 0 | 1 | 63 | 20 | 100 | 49.81095990037390 | 8.50903162922327 | 1.8083298279843000 | 4.7054644001023300 | 2.53288704095989E-06 | 0.00017793739700521000 | 3.749732766889830 | up |
| **C0_90D3.tig01276.1** | 38 | 65 | 149 | 2 | 5 | 16 | 34.733059021879 | -2.9245539658249300 | 0.8206241283275170 | -3.563816691309530 | 0.0003655012465738430 | 0.018717363683825200 | 1.7277553210997800 | down |
| **LP_90D2.tig26190.1** | 3 | 1 | 19 | 522 | 243 | 355 | 357.1925153196170 | 6.9912925165844700 | 1.2768776550502200 | 5.475303361236660 | 4.36762725839634E-08 | 4.20261402105463E-06 | 5.376480494891920 | up |
| **LP_90D1.tig00176.4** | 10 | 1 | 22 | 859 | 279 | 446 | 552.149931417199 | 7.066198737195170 | 1.3058901193758900 | 5.411020906240000 | 6.26664547276487E-08 | 5.81213960250406E-06 | 5.235663962875560 | up |
| **C0_90D2.tig18010.1** | 71 | 24 | 122 | 11057 | 3567 | 4824 | 6948.409935317490 | 7.998936404263730 | 1.1486133255051100 | 6.96399408455942 | 3.30758502271908E-12 | 1.87181281904352E-09 | 8.72773758279928 | up |
| **C0_90D1.tig01118.5** | 2 | 0 | 3 | 150 | 76 | 71 | 98.86206955993290 | 7.283141166094220 | 1.3386903157666500 | 5.440497387869170 | 5.31320189746061E-08 | 4.99725346913556E-06 | 5.301268621849160 | up |
| **C0_90D1.tig07944.2** | 53 | 1 | 9 | 6784 | 1528 | 2622 | 4122.13430074694 | 8.92943773640397 | 1.5397296185184900 | 5.799354398985830 | 6.65707100649164E-09 | 7.69110532303631E-07 | 6.114011241316940 | up |
| **C0_90D1.tig00667.2** | 56 | 9 | 94 | 4281 | 822 | 1531 | 2579.9169158574100 | 7.010786971863000 | 1.3214018483066500 | 5.30556770512105 | 1.12322866597021E-07 | 9.84343357692372E-06 | 5.006853384962530 | up |
| **C0_90D3.tig22790.1** | 0 | 0 | 23 | 100 | 237 | 467 | 151.7570145335310 | 5.778329767800050 | 1.7465327695328400 | 3.3084576874819300 | 0.0009381135934449630 | 0.04302565215870040 | 1.3662725381583700 | up |
| **LP_90D2.tig00017.14** | 1 | 0 | 2 | 236 | 108 | 63 | 146.52302973303800 | 8.603876275630640 | 1.5139133699250200 | 5.683202517761480 | 1.32195572387622E-08 | 1.44244377988572E-06 | 5.840901104731130 | up |
| **C0_90D3.tig00618.4** | 0 | 2 | 13 | 1091 | 298 | 438 | 672.376889371444 | 8.549970806128440 | 1.562582055065550 | 5.471693968589540 | 4.45754100231887E-08 | 4.25236675789785E-06 | 5.371369285526400 | up |
| **C0_90D3.tig00604.3** | 0 | 2 | 28 | 241 | 206 | 146 | 176.69215465226400 | 5.604540818418870 | 1.5530734351673100 | 3.6086772792009700 | 0.0003077621727649750 | 0.01600602988543570 | 1.795716376554410 | up |
| **LP_90D1.tig00250.1** | 0 | 21 | 73 | 3129 | 3433 | 1532 | 2305.1736996760100 | 7.678902630060210 | 1.55391726996011 | 4.941641861189510 | 7.7467419199716E-07 | 5.86521464775355E-05 | 4.231716089494040 | up |
| **C0_90D3.tig09406.1** | 16 | 17 | 28 | 1052 | 388 | 650 | 699.4942765646620 | 6.507843073451660 | 1.0063013707271600 | 6.46709153218092 | 9.99071386299467E-11 | 2.33272688931139E-08 | 7.632136104501610 | up |
| **C0_90D3.tig09702.1** | 12 | 15 | 37 | 0 | 0 | 1 | 7.9940224384291400 | -5.107740104427340 | 1.4923184925144400 | -3.422687670258100 | 0.0006200527842986800 | 0.030223273598465100 | 1.5196584973513500 | down |
| **C0_90D3.tig04643.1** | 18 | 19 | 113 | 8934 | 5358 | 4124 | 5961.014834526090 | 8.35718655285066 | 1.1463491626401900 | 7.2902627098387300 | 3.09351206816656E-13 | 3.4489822757128E-10 | 9.462309037462100 | up |
| **LP_90D1.tig00128.7** | 0 | 0 | 8 | 377 | 149 | 174 | 241.80426718394100 | 7.98244544933258 | 1.742906347682540 | 4.579962348491350 | 4.65059642909631E-06 | 0.0003181941888751980 | 3.4973077560611500 | up |
| **LP_90D1.tig00071.14** | 0 | 1 | 17 | 942 | 938 | 376 | 668.3544527178910 | 8.28439233570461 | 1.5518958568650700 | 5.338239869033240 | 9.38532396019422E-08 | 8.49231928745054E-06 | 5.070973685941370 | up |
| **LP_90D1.tig00065.1** | 1 | 0 | 0 | 177 | 87 | 68 | 113.46873227148700 | 9.70377467545254 | 1.87229037007377 | 5.182836396829940 | 2.18536721655175E-07 | 1.81060114127658E-05 | 4.742177210216510 | up |
| **LP_90D1.tig01671.2** | 0 | 0 | 3 | 61 | 31 | 84 | 48.143053186205300 | 7.020194708660520 | 1.4424950973319500 | 4.866702647132130 | 1.13475562726308E-06 | 8.36387541692869E-05 | 4.0775924447859000 | up |
| **C0_90D2.tig23581.1** | 24 | 22 | 67 | 0 | 1 | 3 | 14.445808437770300 | -4.322995709531950 | 1.0603709846565500 | -4.076870993345930 | 4.56458009270262E-05 | 0.0026738028897411900 | 2.5728706116787400 | down |
| **LP_90D1.tig06725.1** | 0 | 0 | 6 | 932 | 506 | 524 | 627.1842082848270 | 9.769662835456330 | 1.6105590702585200 | 6.066007149857670 | 1.31129295212553E-09 | 1.8319146601891E-07 | 6.737094761821130 | up |
| **C0_90D1.tig10566.1** | 39 | 8 | 18 | 2641 | 839 | 1274 | 1677.268421589740 | 7.626568832217820 | 1.1688485844055300 | 6.524856113930850 | 6.80668446898376E-11 | 1.74821836719153E-08 | 7.757404321276460 | up |
| **C0_90D2.tig10722.1** | 27 | 18 | 42 | 3620 | 1395 | 1839 | 2342.9713476121100 | 7.744572544988150 | 1.0359232304552200 | 7.47601011088915 | 7.66130647682094E-14 | 1.21811124740273E-10 | 9.914313046738570 | up |
| **LP_90D1.tig01014.3** | 0 | 0 | 4 | 67 | 34 | 109 | 55.33217883992500 | 6.8174075294183700 | 1.4438375833330200 | 4.721727435353760 | 2.33849935664442E-06 | 0.00016612798940212900 | 3.77955719103541 | up |
| **LP_90D1.tig00136.1** | 35 | 7 | 63 | 6421 | 1637 | 3103 | 4019.4099287129500 | 8.258993718812450 | 1.2384220448221800 | 6.668965360672580 | 2.5761304107954E-11 | 9.50681993126573E-09 | 8.021964731986560 | up |
| **C0_90D3.tig18284.1** | 7 | 1 | 31 | 1436 | 659 | 397 | 896.1963935529050 | 7.5551107012540700 | 1.3959724319507500 | 5.412077293458060 | 6.2297785893997E-08 | 5.79404115101578E-06 | 5.237018424631810 | up |
| **C0_90D3.tig00427.2** | 17 | 66 | 80 | 1 | 5 | 8 | 22.205233882491800 | -3.0357522480434300 | 0.8356983831079130 | -3.6325931812308300 | 0.00028058720237489100 | 0.01473038694983530 | 1.831785844605370 | down |
| **C0_90D1.tig00101.8** | 220 | 3 | 4 | 15489 | 2328 | 3854 | 8971.130058965820 | 8.171750978486940 | 1.8130130687080000 | 4.507276378493150 | 6.56650774070521E-06 | 0.00043675124891316000 | 3.3597658444553500 | up |
| **C0_90D1.tig08275.2** | 81 | 81 | 486 | 23719 | 16289 | 6261 | 15455.77783337390 | 7.617781885053890 | 1.196922805317370 | 6.364472170813030 | 1.95962446319117E-10 | 3.87159178707042E-08 | 7.412110440095230 | up |
| **LP_90D1.tig00161.8** | 7 | 1 | 2 | 1152 | 242 | 383 | 688.7819433311080 | 9.03814705428512 | 1.3877624407816100 | 6.51274799539517 | 7.37882474427904E-11 | 1.8664513589904E-08 | 7.728983323603910 | up |
| **C0_90D1.tig25377.1** | 2 | 0 | 0 | 122 | 41 | 27 | 73.1290806910168 | 8.10501106110041 | 1.795454138510810 | 4.51418439895263 | 6.35608964669532E-06 | 0.0004244469544270200 | 3.3721765783037300 | up |
| **C0_90D1.tig16327.1** | 46 | 45 | 88 | 3 | 9 | 12 | 26.652437801175200 | -2.352039031269750 | 0.5619369236825690 | -4.18559260326945 | 2.84423082072032E-05 | 0.0017110995112257800 | 2.7667247327619100 | down |
| **LP_90D3.tig05604.1** | 3 | 3 | 50 | 1580 | 779 | 508 | 1004.567714717120 | 7.223271346967520 | 1.417181182049670 | 5.096928634432240 | 3.45208390785994E-07 | 2.74566225371764E-05 | 4.561352886752590 | up |
| **LP_90D1.tig09375.1** | 0 | 0 | 7 | 283 | 217 | 104 | 191.37105962957800 | 7.832812971297100 | 1.661172591978620 | 4.715231282480670 | 2.41436341251076E-06 | 0.00017042955598376700 | 3.7684550874591100 | up |
| **LP_90D1.tig00071.8** | 1 | 3 | 40 | 2301 | 1415 | 968 | 1526.9695321420500 | 8.185388641257580 | 1.4369314179042300 | 5.6964365447559 | 1.22337394004112E-08 | 1.34365896329056E-06 | 5.871710946427680 | up |
| **LP_90D1.tig00628.4** | 21 | 6 | 16 | 2337 | 673 | 1057 | 1463.304789222700 | 8.050497364283700 | 1.155922346684340 | 6.964565904773640 | 3.29417902978882E-12 | 1.87181281904352E-09 | 8.72773758279928 | up |
| **LP_90D1.tig00493.6** | 0 | 0 | 3 | 163 | 167 | 134 | 126.02926735351900 | 8.417902451689680 | 1.368933373147610 | 6.149241896509730 | 7.78541655962399E-10 | 1.17093366445624E-07 | 6.931467707811190 | up |
| **LP_90D2.tig20535.1** | 2 | 1 | 4 | 8 | 16 | 9 | 8.384095768928650 | 2.984847669019420 | 0.8860676470931020 | 3.368645361120830 | 0.0007553855354816580 | 0.035573438144142600 | 1.4488741585738500 | up |
| **LP_90D1.tig00067.1** | 0 | 0 | 1 | 103 | 43 | 41 | 65.2898822770177 | 8.901057589795100 | 1.887489334476700 | 4.715818747798580 | 2.40740679505522E-06 | 0.0001702985285595310 | 3.768789104481540 | up |
| **LP_90D1.tig01643.3** | 0 | 0 | 2 | 72 | 12 | 15 | 41.530408751974600 | 7.39820698153949 | 1.8940226347034400 | 3.906081609577960 | 9.3804842537985E-05 | 0.005246314719431800 | 2.280145660216720 | up |
| **LP_90D1.tig00076.5** | 60 | 23 | 186 | 16214 | 6167 | 8079 | 10445.578783558000 | 8.303620408609940 | 1.1739334254496900 | 7.073331611994230 | 1.51257440848696E-12 | 1.0306805494892E-09 | 8.986875919674590 | up |
| **C0_90D1.tig18379.1** | 0 | 0 | 4 | 94 | 46 | 34 | 60.331437548212200 | 6.950035454836820 | 1.6366664713256700 | 4.246458014874220 | 2.17176453641292E-05 | 0.0013184190201143800 | 2.8799465402714000 | up |
| **C0_90D1.tig00481.7** | 66 | 48 | 192 | 16134 | 5192 | 7080 | 10141.14620582690 | 8.070800481155300 | 1.1320324798648200 | 7.1294778415890000 | 1.00750395636401E-12 | 8.20476819488728E-10 | 9.085933684337080 | up |
| **LP_90D1.tig00135.7** | 1 | 0 | 10 | 325 | 156 | 139 | 210.93438544776900 | 7.3044201165155400 | 1.51129374511039 | 4.8332232831295200 | 1.34339966663909E-06 | 9.77228136588508E-05 | 4.010004037206980 | up |
| **C0_90D1.tig05882.1** | 849 | 290 | 1993 | 107787 | 33601 | 49419 | 68047.45538911120 | 7.451114900623880 | 1.1790398901090100 | 6.319646148643020 | 2.6216290685323E-10 | 4.80387262490995E-08 | 7.318408516471490 | up |
| **C0_90D1.tig00064.21** | 423 | 135 | 1258 | 80295 | 28762 | 36175 | 51014.94359032970 | 7.833763485127310 | 1.2061174874527700 | 6.495025208258620 | 8.30195087205362E-11 | 2.03818998284558E-08 | 7.690755337181410 | up |
| **C0_90D3.tig01558.2** | 21 | 5 | 74 | 2792 | 1851 | 983 | 1848.7103551128000 | 7.232471318355890 | 1.2350256804949900 | 5.856130307717270 | 4.73776534437756E-09 | 5.69026068645404E-07 | 6.244867836927400 | up |
| **C0_90D1.tig00064.18** | 172 | 64 | 489 | 30483 | 11147 | 14760 | 19546.779599664000 | 7.772098996137720 | 1.1702561232584400 | 6.641365801613810 | 3.1078964411069E-11 | 1.035897245695E-08 | 7.984683321657260 | up |
| **C0_90D3.tig15877.2** | 17 | 21 | 46 | 1 | 3 | 1 | 11.3885953000107 | -3.49473287177315 | 0.9147403680328310 | -3.8204642474548800 | 0.00013320071369222500 | 0.007314866166890980 | 2.135793615336680 | down |
| **C0_90D1.tig00064.19** | 38 | 11 | 118 | 8110 | 2948 | 3980 | 5202.458173493230 | 7.984734204501750 | 1.217253093157070 | 6.5596335301087700 | 5.39401737188026E-11 | 1.50084038358092E-08 | 7.823665493110540 | up |
| **C0_3D1.tig08857.2** | 7 | 1 | 5 | 710 | 95 | 201 | 413.427755461322 | 7.947774606468690 | 1.4166917870010200 | 5.610094361663000 | 2.02216323870931E-08 | 2.10254947808707E-06 | 5.677253775407650 | up |
| **LP_90D1.tig00408.1** | 1 | 0 | 0 | 361 | 181 | 121 | 229.22768664995200 | 10.72239283481180 | 1.8875234574634700 | 5.680667327557850 | 1.34170218322533E-08 | 1.45922130930653E-06 | 5.835878836878420 | up |
| **LP_90D2.tig12458.1** | 1 | 2 | 11 | 938 | 752 | 544 | 665.7637283155180 | 8.61328359904524 | 1.1685545344387100 | 7.370887147498410 | 1.69496283219191E-13 | 2.26372456016223E-10 | 9.645176417271260 | up |
| **C0_90D3.tig00822.3** | 33 | 12 | 100 | 4638 | 1877 | 2352 | 3016.6389343678200 | 7.397950479150690 | 1.1689604137869300 | 6.3286578329752700 | 2.47302814962396E-10 | 4.6028572574359E-08 | 7.336972493120180 | up |
| **C0_90D2.tig11894.1** | 14 | 18 | 33 | 0 | 0 | 2 | 8.31124020911824 | -4.409308674414570 | 1.2002728572068100 | -3.673588591077190 | 0.0002391677029108710 | 0.012736156989619000 | 1.8949615963318300 | down |
| **LP_90D1.tig00180.4** | 0 | 0 | 4 | 263 | 125 | 60 | 162.48906046573500 | 8.394692970837420 | 1.712820007672320 | 4.901094646976720 | 9.53041246858986E-07 | 7.1029228105747E-05 | 4.148562904900470 | up |
| **C0_90D3.tig08365.1** | 0 | 0 | 44 | 1393 | 753 | 2653 | 1206.6695612618600 | 7.8570108155282100 | 2.02808714820606 | 3.8740992084477800 | 0.00010701981217093100 | 0.0059258449561778000 | 2.227249715589230 | up |
| **LP_90D3.tig01908.4** | 0 | 2 | 3 | 22 | 25 | 90 | 28.092650188283800 | 5.468892838368460 | 1.0522720648354000 | 5.1972232477956300 | 2.022873740878E-07 | 1.68433245222383E-05 | 4.7735721836698900 | up |
| **LP_90D1.tig11853.1** | 7 | 2 | 10 | 805 | 255 | 323 | 501.67973515693000 | 7.708936444671300 | 1.2089655777861800 | 6.3764730661626 | 1.81212601348358E-10 | 3.64488406410864E-08 | 7.438316281095970 | up |
| **LP_90D3.tig12502.1** | 0 | 0 | 4 | 12 | 19 | 111 | 24.94855344071950 | 5.664254650121650 | 1.4133534104316800 | 4.007670415845690 | 6.13206124683216E-05 | 0.0035300584994910200 | 2.452218097502930 | up |
| **LP_90D1.tig00209.3** | 0 | 0 | 9 | 617 | 475 | 225 | 416.48941285577900 | 8.600976830237100 | 1.7189188090567800 | 5.003713255634620 | 5.62363894589064E-07 | 4.33643604536588E-05 | 4.362867054289110 | up |
| **C0_90D1.tig00169.4** | 157 | 6 | 80 | 19623 | 3280 | 6106 | 11574.172225009500 | 8.509482296474800 | 1.4649554080500500 | 5.808697145124360 | 6.29608615030168E-09 | 7.29930626640357E-07 | 6.1367184137137300 | up |
| **LP_90D1.tig00625.4** | 0 | 1 | 0 | 646 | 725 | 332 | 478.050693938829 | 11.784692289942600 | 1.7446928334255200 | 6.754594312630160 | 1.43235470644945E-11 | 6.13139631969754E-09 | 8.212440611144840 | up |
| **LP_90D1.tig01056.2** | 0 | 1 | 0 | 476 | 607 | 90 | 339.6782307415440 | 11.291499340829600 | 1.885837361353510 | 5.987525526976220 | 2.13057512508403E-09 | 2.7466321564259E-07 | 6.561199499709910 | up |
| **C0_90D2.tig14840.1** | 8 | 22 | 26 | 0 | 2 | 1 | 7.275359447924980 | -3.7016142672530200 | 1.0891378773849000 | -3.3986645255060700 | 0.0006771572082558270 | 0.03243845340954640 | 1.4889398601240000 | down |
| **LP_3D1.tig34160.2** | 0 | 0 | 1 | 21 | 9 | 6 | 13.100022526468700 | 6.532039891401590 | 1.9241511092900400 | 3.3947645067292700 | 0.0006868765434485280 | 0.03276302987028990 | 1.4846159422259700 | up |
| **C0_90D1.tig00244.5** | 180 | 81 | 368 | 21123 | 8755 | 10850 | 13782.018316359800 | 7.457165673545320 | 1.0763916022315100 | 6.927930000648090 | 4.27042940085949E-12 | 2.33746503713602E-09 | 8.631254876409440 | up |
| **LP_90D2.tig06135.2** | 20 | 25 | 17 | 34 | 63 | 42 | 39.66669997754840 | 1.9766808357099000 | 0.6003550328506950 | 3.2925198050292600 | 0.000992939041694282 | 0.045106451242354300 | 1.3457613397497500 | up |
| **LP_90D1.tig00371.1** | 0 | 1 | 0 | 579 | 742 | 105 | 412.98748317973900 | 11.573835910704400 | 1.8917481966022100 | 6.118063668035880 | 9.47192251784346E-10 | 1.38709658310647E-07 | 6.857893298083300 | up |
| **C0_90D3.tig03515.2** | 39 | 6 | 147 | 6618 | 3564 | 2181 | 4249.4798838961300 | 7.497512580890080 | 1.325716644834610 | 5.655441236332550 | 1.55446408415015E-08 | 1.65964228697161E-06 | 5.779985508065490 | up |
| **C0_90D1.tig05586.1** | 167 | 59 | 487 | 43102 | 11672 | 16267 | 26420.535782723700 | 8.233823905260470 | 1.2463892022739600 | 6.606141877864770 | 3.94464870531775E-11 | 1.24252712850806E-08 | 7.905694120395250 | up |
| **C0_90D3.tig09441.1** | 28 | 28 | 96 | 0 | 8 | 7 | 20.614622686372500 | -2.9693234483666800 | 0.900788944130028 | -3.296358672823650 | 0.0009794688057699450 | 0.04461593991248660 | 1.3505099534118900 | down |
| **C0_90D1.tig02920.2** | 16 | 4 | 48 | 2853 | 1207 | 1493 | 1866.3438975616700 | 7.798829570222960 | 1.2081900899096200 | 6.4549689948262700 | 1.08241061150821E-10 | 2.40937386050985E-08 | 7.61809580566429 | up |
| **LP_90D1.tig00483.2** | 1 | 1 | 2 | 95 | 16 | 15 | 54.32372049904060 | 6.741026349161140 | 1.5367644024608400 | 4.386506050222570 | 1.15185962453907E-05 | 0.0007325607810235270 | 3.1351563357956800 | up |
| **LP_90D3.tig01132.1** | 21 | 27 | 44 | 7387 | 3450 | 1598 | 4541.862341053240 | 8.634260459258570 | 1.1234432482450000 | 7.685533268143890 | 1.52361006530709E-14 | 2.99245979238462E-11 | 10.523971676197300 | up |
| **C0_90D1.tig04943.3** | 50 | 19 | 81 | 5903 | 2001 | 2523 | 3718.828907144590 | 7.625504627021770 | 1.1277780899685300 | 6.761529324651600 | 1.36542672069278E-11 | 5.92080945158586E-09 | 8.227618915519320 | up |
| **LP_90D3.tig24367.2** | 0 | 0 | 1 | 123 | 41 | 56 | 77.61362647964040 | 9.15282864550692 | 1.9003931626507300 | 4.816281612348190 | 1.46258072543507E-06 | 0.00010616110400337300 | 3.9740345737525200 | up |
| **C0_90D1.tig05299.1** | 30 | 11 | 93 | 6411 | 2147 | 2884 | 4051.994607191550 | 7.941077537125390 | 1.2098309177908500 | 6.563791204498070 | 5.24566412357558E-11 | 1.47182755816861E-08 | 7.83214306970151 | up |
| **C0_90D1.tig01862.1** | 637 | 178 | 1247 | 91192 | 24702 | 36255 | 56231.60396325550 | 7.772445469449630 | 1.2150027131097000 | 6.397060175739610 | 1.58396915990751E-10 | 3.28491591802186E-08 | 7.4834757423314400 | up |
| **C0_90D2.tig12463.1** | 0 | 19 | 45 | 35399 | 28728 | 16177 | 24496.02164737520 | 11.643611743511100 | 1.5042350959978000 | 7.740553171834860 | 9.8985195186182E-15 | 2.06563542629465E-11 | 10.684946326727400 | up |
| **C0_90D1.tig23132.2** | 9 | 0 | 6 | 1085 | 250 | 403 | 658.2627477210600 | 8.406383294365250 | 1.5162914667391900 | 5.544041814363920 | 2.95568203200726E-08 | 2.96358160260332E-06 | 5.528183109905510 | up |
| **C0_90D2.tig13100.1** | 27 | 30 | 90 | 1 | 7 | 9 | 20.697476274664300 | -2.6343693270781600 | 0.7804475627718530 | -3.375459739693310 | 0.0007369247336161190 | 0.0348022346969004 | 1.4583928685427100 | down |
| **LP_90D2.tig22478.2** | 0 | 1 | 0 | 35 | 5 | 7 | 20.049236467522400 | 7.174319973609510 | 2.0971680875731000 | 3.420956105579420 | 0.0006240140285713 | 0.030372018075753800 | 1.5175263503258700 | up |
| **C0_90D3.tig14544.1** | 15 | 3 | 44 | 2382 | 619 | 1030 | 1478.1382759928800 | 7.593969558911830 | 1.311469176456280 | 5.790429310303360 | 7.02067417599149E-09 | 8.07903586963514E-07 | 6.092640463666080 | up |
| **C0_90D1.tig05886.1** | 66 | 44 | 49 | 7145 | 2000 | 4261 | 4616.043939956050 | 7.825993093240020 | 1.045170455774630 | 7.487767234522300 | 7.00551238895015E-14 | 1.16953526577328E-10 | 9.931986678087560 | up |
| **LP_90D1.tig00139.2** | 13 | 3 | 27 | 2420 | 699 | 1021 | 1504.7295913506900 | 8.134733546384060 | 1.2456387494003000 | 6.530572005969200 | 6.55189788655219E-11 | 1.69582417468288E-08 | 7.770619177977550 | up |
| **LP_90D2.tig20579.1** | 0 | 0 | 1 | 667 | 395 | 151 | 420.8785868481090 | 11.60097247880380 | 1.9211731717608700 | 6.038483489841190 | 1.55569341470872E-09 | 2.12012438464121E-07 | 6.673638658892640 | up |
| **LP_90D1.tig00627.2** | 2 | 0 | 1 | 199 | 31 | 0 | 108.36063823287200 | 8.115769229114960 | 2.362099694151400 | 3.43582840690837 | 0.0005907450026404460 | 0.028963854468666500 | 1.5381436433353000 | up |
| **C0_90D3.tig16347.1** | 14 | 18 | 48 | 0 | 0 | 3 | 10.216879901294900 | -4.193840218114650 | 1.2353134820991200 | -3.3949602905557300 | 0.0006863855505253590 | 0.03276302987028990 | 1.4846159422259700 | down |
| **LP_90D1.tig15959.1** | 0 | 0 | 3 | 26 | 12 | 33 | 20.168248056438300 | 5.734493337976900 | 1.4699108875556400 | 3.90125237286523 | 9.56963199021536E-05 | 0.005343151212730780 | 2.2722025349832300 | up |
| **C0_90D2.tig14898.1** | 183 | 64 | 300 | 26497 | 9277 | 12298 | 16853.16024181950 | 7.941172758386090 | 1.118152293272080 | 7.102049341729320 | 1.22920325578088E-12 | 9.12041500161505E-10 | 9.039985399740570 | up |
| **LP_90D1.tig00029.29** | 0 | 0 | 2 | 400 | 119 | 175 | 249.4014639914680 | 9.994980363177310 | 1.7162205044392400 | 5.823832274071980 | 5.75132977405994E-09 | 6.71437586804501E-07 | 6.172994350778090 | up |
| **C0_90D3.tig03495.1** | 1 | 7 | 30 | 1106 | 661 | 418 | 726.8043719827780 | 7.31087300117677 | 1.3217327190615000 | 5.53127943020726 | 3.17903430700093E-08 | 3.12190519048394E-06 | 5.5055802902149500 | up |
| **LP_90D2.tig01000.4** | 1 | 0 | 4 | 43 | 43 | 28 | 32.58185537669260 | 5.672953414250290 | 1.238182440530950 | 4.581678134457840 | 4.61259453970189E-06 | 0.000316576649635158 | 3.4995211214022300 | up |
| **C0_90D3.tig00506.3** | 6 | 4 | 43 | 2683 | 1303 | 838 | 1694.872078614090 | 8.04598408271876 | 1.2987805479528600 | 6.195029711063070 | 5.82740366926783E-10 | 9.35160553995062E-08 | 7.0291138204343000 | up |
| **C0_90D2.tig07214.1** | 75 | 107 | 306 | 5 | 7 | 28 | 66.99248642292500 | -2.9996719428880300 | 0.8435891786357390 | -3.555844502106020 | 0.0003767668826075310 | 0.01923527437826120 | 1.7159016143940800 | down |
| **LP_90D1.tig02677.2** | 0 | 0 | 2 | 73 | 76 | 43 | 54.25895746823930 | 7.766982294482200 | 1.4873344178159100 | 5.222082002168480 | 1.76922586228167E-07 | 1.49173440191219E-05 | 4.826308494592550 | up |
| **C0_3D2.tig11655.1** | 0 | 0 | 2 | 60 | 27 | 53 | 42.58582007121350 | 7.417123209800770 | 1.5609199830051200 | 4.75176388960127 | 2.01649762552271E-06 | 0.00014417310325177300 | 3.8411157534784100 | up |
| **LP_90D1.tig00052.10** | 0 | 0 | 1 | 54 | 29 | 25 | 35.61353079573760 | 8.014265391176880 | 1.8371519997314100 | 4.362331147530820 | 1.28683895473822E-05 | 0.0008106842615048010 | 3.091148258224820 | up |
| **C0_90D3.tig17598.1** | 17 | 10 | 40 | 1 | 1 | 0 | 8.922948541787000 | -4.366583131038020 | 1.3113357796773800 | -3.3298741624455000 | 0.0008688524109244380 | 0.0400692170557404 | 1.3971891435092400 | down |
| **LP_90D1.tig02705.2** | 73 | 118 | 2104 | 62123 | 35805 | 17767 | 39860.15714164830 | 7.1852882590991400 | 1.4699478788199900 | 4.888124512868580 | 1.01801140000176E-06 | 7.57024112130482E-05 | 4.120890287476880 | up |
| **LP_90D1.tig00092.4** | 6 | 2 | 13 | 693 | 148 | 268 | 421.80260221097000 | 7.329351664264940 | 1.2839434778243800 | 5.708469096072970 | 1.13996870977986E-08 | 1.26034487585562E-06 | 5.899510599972220 | up |
| **LP_90D1.tig00359.4** | 0 | 0 | 3 | 109 | 267 | 137 | 111.09721481789600 | 8.222691340422400 | 1.1680641516810400 | 7.039588817607790 | 1.92807950311939E-12 | 1.26228718685595E-09 | 8.898841826171440 | up |
| **C0_90D1.tig07812.2** | 12 | 2 | 3 | 910 | 109 | 222 | 523.2900174390230 | 7.864360850180740 | 1.4530367456999700 | 5.412361988403980 | 6.21987901537076E-08 | 5.79404115101578E-06 | 5.237018424631810 | up |
| **LP_90D2.tig20841.1** | 3 | 11 | 6 | 6 | 28 | 26 | 12.906059010382900 | 2.081787405600350 | 0.6103630999940160 | 3.4107360120897800 | 0.0006478778589046230 | 0.03126010669214810 | 1.505009544047730 | up |
| **LP_90D1.tig01551.1** | 7 | 1 | 2 | 263 | 37 | 65 | 152.732549409788 | 6.850604622454530 | 1.4680487068749200 | 4.66646957309587 | 3.06418995881977E-06 | 0.00021359131218169800 | 3.670416416195360 | up |
| **LP_90D2.tig01794.2** | 0 | 1 | 8 | 212 | 73 | 82 | 132.8725185467630 | 6.931133893680310 | 1.5275875051872900 | 4.537307270545220 | 5.69770604697588E-06 | 0.0003835498129082210 | 3.416178224762460 | up |
| **LP_90D1.tig00237.1** | 0 | 3 | 6 | 284 | 149 | 107 | 183.829643775464 | 7.386449980768290 | 1.3557509782730800 | 5.448235036626660 | 5.08721049055753E-08 | 4.79821669687078E-06 | 5.318920142335240 | up |
| **C0_90D1.tig00731.5** | 68 | 34 | 144 | 12331 | 4393 | 5710 | 7849.564934941920 | 8.003362809539200 | 1.1068609739428300 | 7.230684790547660 | 4.8056459037196E-13 | 4.71928561997923E-10 | 9.326123737548520 | up |
| **C0_90D3.tig02499.2** | 0 | 3 | 86 | 4792 | 3102 | 1944 | 3189.3897049320400 | 8.242837680829400 | 1.8116759257785100 | 4.549841151798320 | 5.3686428047722E-06 | 0.0003635975955548460 | 3.439378997399230 | up |
| **LP_90D1.tig00309.4** | 8 | 3 | 19 | 2981 | 1752 | 1138 | 1948.2550796318300 | 9.03377205724207 | 1.1395053776004200 | 7.927801162523200 | 2.23060078414053E-15 | 6.20646079847234E-12 | 11.20715598337570 | up |
| **LP_90D1.tig00076.12** | 0 | 0 | 3 | 256 | 177 | 82 | 168.52467752668300 | 8.850944532073460 | 1.5986742273400100 | 5.536427860478050 | 3.08702903400517E-08 | 3.05854042778631E-06 | 5.514485774623930 | up |
| **C0_3D3.tig01105.2** | 2 | 0 | 1 | 29 | 40 | 121 | 37.873575929172 | 6.612064369887850 | 1.1267823668770100 | 5.868093577124280 | 4.4083444334225E-09 | 5.33297870607044E-07 | 6.2730301503982400 | up |
| **LP_90D1.tig00179.7** | 3 | 0 | 3 | 457 | 95 | 145 | 272.36771965187700 | 8.476665179398480 | 1.5098240268137200 | 5.614339836204190 | 1.9731409280783E-08 | 2.06524145603782E-06 | 5.685029165850380 | up |
| **LP_90D3.tig00132.4** | 0 | 0 | 1 | 16 | 22 | 76 | 22.10077839365210 | 7.3360244743556000 | 1.6136155979392000 | 4.54632719448465 | 5.45901506391109E-06 | 0.0003689697448763720 | 3.433009244063010 | up |
| **C0_3D2.tig03578.1** | 5 | 1 | 4 | 123 | 29 | 35 | 74.25030202211840 | 5.828162667544600 | 1.3391326825616200 | 4.352192089282700 | 1.34783090321453E-05 | 0.0008448624667976910 | 3.073213983088440 | up |
| **C0_90D1.tig01945.2** | 89 | 23 | 206 | 16371 | 5278 | 7149 | 10287.550131350700 | 8.027350537411400 | 1.213959832806430 | 6.6125338915487800 | 3.77796583185664E-11 | 1.20135715390344E-08 | 7.92032786111442 | up |
| **C0_90D3.tig00368.5** | 13 | 4 | 42 | 3088 | 850 | 1150 | 1893.4017022695000 | 8.028023776756120 | 1.2879114530194600 | 6.2333662441891500 | 4.56517160578279E-10 | 7.5086953076592E-08 | 7.124435518376480 | up |
| **LP_90D1.tig02424.1** | 0 | 2 | 9 | 5349 | 2888 | 3469 | 3660.3325107949700 | 11.435779476171400 | 1.3504676312781100 | 8.468014494615030 | 2.4961125679898E-17 | 1.04178378165764E-13 | 12.982222407885700 | up |
| **LP_90D1.tig00055.9** | 0 | 0 | 4 | 293 | 117 | 97 | 182.39113775980200 | 8.5610820389802 | 1.6795093187872700 | 5.097370966159280 | 3.44403012989378E-07 | 2.74566225371764E-05 | 4.561352886752590 | up |
| **LP_90D1.tig00408.2** | 5 | 0 | 4 | 437 | 81 | 204 | 268.88844300543700 | 7.859559829081460 | 1.4843101765937200 | 5.295092597908350 | 1.18956116836183E-07 | 1.0343296315217E-05 | 4.985341033449950 | up |
| **LP_90D2.tig09082.1** | 0 | 1 | 0 | 55 | 15 | 22 | 33.91597415639220 | 7.944773766666690 | 1.9403293468739600 | 4.094549092640600 | 4.22990783602505E-05 | 0.00249087112411006 | 2.6036487419680100 | up |
| **LP_90D2.tig06515.1** | 0 | 0 | 4 | 58 | 84 | 14 | 43.56612698423820 | 6.47327388874062 | 1.5898285200328700 | 4.071680566283210 | 4.66751594878599E-05 | 0.0027293115589144500 | 2.563946885512180 | up |
| **LP_90D2.tig00177.2** | 0 | 0 | 2 | 193 | 97 | 68 | 123.21297399337100 | 8.971397767444500 | 1.6700922063601500 | 5.371797876356190 | 7.79554612807421E-08 | 7.15070026566675E-06 | 5.1456514258040700 | up |
| **LP_90D2.tig08133.2** | 1 | 0 | 0 | 58 | 40 | 17 | 38.00141697729910 | 8.110107465941520 | 1.8696334439790600 | 4.337806157703900 | 1.43911990725854E-05 | 0.0008948002715727270 | 3.0482738927666000 | up |
| **LP_90D2.tig23781.1** | 0 | 0 | 3 | 486 | 274 | 178 | 314.81269257315500 | 9.7556992958296 | 1.6040284886019200 | 6.08199877069061 | 1.1869347883451E-09 | 1.68640704885338E-07 | 6.77303759119731 | up |
| **LP_90D1.tig00171.7** | 0 | 0 | 1 | 51 | 28 | 13 | 32.21305942099980 | 7.8690582945648800 | 1.9121559155908500 | 4.115280678946800 | 3.86708550148175E-05 | 0.002293394632486220 | 2.639521208259710 | up |
| **LP_90D1.tig05075.1** | 181 | 38 | 306 | 5672 | 3763 | 2138 | 3816.7802845255400 | 5.839382403206990 | 1.1325361344187500 | 5.156023040451550 | 2.52249871263094E-07 | 2.06937861218758E-05 | 4.684160043823880 | up |
| **C0_90D1.tig00123.2** | 169 | 30 | 305 | 18664 | 6018 | 7913 | 11712.839615497900 | 7.536362355657010 | 1.2326060523354500 | 6.114169520243450 | 9.7061086754489E-10 | 1.41180866673886E-07 | 6.850224156407390 | up |
| **C0_90D2.tig02801.1** | 28 | 534 | 2064 | 25804 | 20712 | 23635 | 19815.83564912850 | 5.963223997683940 | 1.3330843324142200 | 4.473253381415520 | 7.70383986392509E-06 | 0.0005033728164708310 | 3.2981102412617900 | up |
| **LP_90D3.tig24402.1** | 0 | 0 | 2 | 47 | 32 | 21 | 31.89106732127050 | 6.992712814100290 | 1.5898627500123000 | 4.3983122530835900 | 1.09095943213318E-05 | 0.0006978169440516210 | 3.1562584894325100 | up |
| **C0_90D3.tig09607.1** | 3 | 2 | 5 | 271 | 148 | 82 | 173.5277295433890 | 7.1020440962088600 | 1.1770085549923900 | 6.0339783139934900 | 1.59971541984648E-09 | 2.17125602249E-07 | 6.663288963925130 | up |
| **FM1.tig00375.5** | 40 | 12 | 20 | 418 | 45 | 138 | 253.12401397274200 | 4.707164349519140 | 1.318903239175750 | 3.568999005917130 | 0.000358347785106093 | 0.018379223036724000 | 1.7356728519268200 | up |
| **LP_90D2.tig05309.1** | 0 | 0 | 3 | 16 | 29 | 107 | 27.638808040216200 | 6.215640624226760 | 1.2721644869742700 | 4.885878113930160 | 1.02968887252481E-06 | 7.64006261438465E-05 | 4.116903082135000 | up |
| **C0_90D3.tig01025.3** | 20 | 3 | 23 | 2317 | 753 | 1056 | 1463.313547772620 | 7.9687858681271900 | 1.2172954281136000 | 6.5463039489733100 | 5.89784853126056E-11 | 1.57538611688207E-08 | 7.802612986077620 | up |
| **C0_90D3.tig00250.3** | 9 | 1 | 50 | 2339 | 637 | 881 | 1436.4573671167500 | 7.6233420422983800 | 1.478230746277650 | 5.157071763995450 | 2.50841683540607E-07 | 2.06289482062496E-05 | 4.685522914531290 | up |
| **LP_90D1.tig03831.1** | 21 | 0 | 4 | 5318 | 1622 | 595 | 3075.2109418981700 | 9.8450612313593 | 1.7549761204130100 | 5.609797829637930 | 2.02563111313518E-08 | 2.10254947808707E-06 | 5.677253775407650 | up |
| **LP_90D3.tig00506.3** | 4 | 2 | 2 | 11 | 10 | 14 | 10.053190702335400 | 3.026313463450320 | 0.9263493394217160 | 3.2669246197547100 | 0.0010872262551147800 | 0.04898974012419360 | 1.3098948643388900 | up |
| **C0_90D3.tig20928.2** | 9 | 4 | 59 | 2441 | 2231 | 785 | 1682.5108673649900 | 7.59085496056728 | 1.2619513744613100 | 6.015172307100670 | 1.79695458652218E-09 | 2.38089351942021E-07 | 6.623260027090840 | up |
| **LP_90D1.tig00310.3** | 0 | 2 | 0 | 23682 | 5099 | 8054 | 14174.23036232810 | 15.79340396794720 | 1.7963086582905000 | 8.792143763854800 | 1.46732841530446E-18 | 8.16543807643344E-15 | 14.088020510367100 | up |
| **C0_90D1.tig10474.1** | 3 | 1 | 7 | 918 | 281 | 365 | 569.0083627283310 | 8.700600283690150 | 1.277253686133970 | 6.811959423679890 | 9.62782661475208E-12 | 4.69642529284492E-09 | 8.328232581698160 | up |
| **LP_90D1.tig07156.2** | 1 | 0 | 0 | 81 | 8 | 7 | 44.48460929809020 | 8.345643103587790 | 2.2269119205623500 | 3.7476305311079900 | 0.0001785129433922570 | 0.009660240951254550 | 2.015012041028070 | up |
| **C0_90D1.tig00095.2** | 1055 | 414 | 4594 | 218375 | 124582 | 112514 | 146841.68992011300 | 7.632950160999790 | 1.1777355892818500 | 6.481038894013660 | 9.10931836286838E-11 | 2.18813691235836E-08 | 7.659925507557310 | up |
| **C0_90D1.tig00064.16** | 189 | 48 | 511 | 33834 | 12097 | 15271 | 21495.431981325800 | 7.862764042093360 | 1.2203032943714100 | 6.44328674548365 | 1.16913505554352E-10 | 2.551388913042E-08 | 7.593223336014150 | up |
| **LP_90D2.tig00025.13** | 0 | 1 | 0 | 168 | 187 | 29 | 116.07184393482600 | 9.73804371030845 | 1.9063319800578500 | 5.10826226081197 | 3.25135159695324E-07 | 2.60334240936862E-05 | 4.584468706758430 | up |
| **LP_90D3.tig01458.3** | 0 | 0 | 2 | 35 | 29 | 103 | 36.88112616840760 | 7.202911695821880 | 1.3888922287849200 | 5.186083949885280 | 2.14762029845351E-07 | 1.78375358569811E-05 | 4.748665140858470 | up |
| **LP_90D1.tig03913.2** | 3 | 3 | 3 | 13 | 7 | 22 | 11.95285158081180 | 3.166312330134890 | 0.9582853245114760 | 3.3041436085323000 | 0.0009526701787437850 | 0.04351395977849010 | 1.3613713940297700 | up |
| **C0_90D1.tig00667.3** | 23 | 1 | 21 | 1469 | 204 | 549 | 877.5412084831490 | 7.245354576003960 | 1.462837030554900 | 4.952947200998580 | 7.30978138241133E-07 | 5.55959659629458E-05 | 4.254956719633630 | up |
| **LP_90D1.tig00128.5** | 5 | 2 | 6 | 591 | 268 | 274 | 383.82685845838500 | 7.8643430568750800 | 1.1286031990928900 | 6.968209077553560 | 3.21000944289373E-12 | 1.87181281904352E-09 | 8.72773758279928 | up |
| **C0_90D3.tig05133.1** | 10 | 19 | 52 | 0 | 2 | 3 | 10.519461770971300 | -3.542181980661530 | 1.0753826491791000 | -3.293880539513510 | 0.0009881448476365670 | 0.044949820596372400 | 1.3472720372825000 | down |
| **LP_90D1.tig00381.2** | 0 | 0 | 10 | 622 | 126 | 350 | 392.0974559626300 | 8.364459184154170 | 1.8597326956845900 | 4.497667435520960 | 6.87030511128371E-06 | 0.0004551440820647850 | 3.341851099737840 | up |
| **C0_90D1.tig21831.1** | 12 | 10 | 8 | 9 | 26 | 53 | 19.39696025045850 | 2.043894058052170 | 0.5924423687609830 | 3.4499457935912100 | 0.0005606991271589710 | 0.027612364537921700 | 1.5588964016982300 | up |
| **LP_90D1.tig12390.1** | 1 | 0 | 1 | 53 | 7 | 5 | 29.55881837031920 | 6.835771970024580 | 1.8639390521513500 | 3.667379554140870 | 0.0002450489162967730 | 0.01301410911796060 | 1.8855855562060100 | up |
| **C0_90D3.tig04512.1** | 22 | 28 | 27 | 1 | 6 | 8 | 12.086472078243600 | -1.8674956182987600 | 0.5645766799454820 | -3.307780297406360 | 0.0009403855252678790 | 0.04307068903041050 | 1.3658181804846400 | down |
| **C0_90D1.tig00605.3** | 98 | 31 | 201 | 15429 | 4614 | 6995 | 9689.715918282320 | 7.881536491428250 | 1.1801278400560300 | 6.678544666020300 | 2.41326678105004E-11 | 9.30754307884767E-09 | 8.031164945024960 | up |
| **LP_90D3.tig24105.1** | 0 | 4 | 8 | 461 | 258 | 287 | 316.2558179973130 | 7.76020782301056 | 1.2864754842549400 | 6.032145904051100 | 1.61796591552049E-09 | 2.18713619244185E-07 | 6.6601241727079600 | up |
| **LP_90D1.tig01238.1** | 0 | 0 | 1 | 18 | 5 | 22 | 13.290526807522200 | 6.558458893458100 | 1.8362231070739600 | 3.5717113395381900 | 0.00035465618913622300 | 0.01824594067653220 | 1.738833741502070 | up |
| **C0_90D3.tig02935.2** | 21 | 39 | 85 | 1 | 1 | 8 | 19.486722512077500 | -3.291590401025110 | 0.9833451186150180 | -3.3473399508619300 | 0.0008159108666459620 | 0.03794212803125630 | 1.4208783148202000 | down |
| **LP_90D3.tig01717.4** | 1 | 2 | 1 | 9 | 4 | 19 | 8.417740130950520 | 3.8918470983549600 | 1.1706012444729800 | 3.3246565529725900 | 0.0008852751566909140 | 0.04077027890586610 | 1.3896563175676100 | up |
| **C0_90D1.tig00095.1** | 295 | 125 | 1571 | 73201 | 41749 | 27629 | 47782.30316089570 | 7.626105548285790 | 1.243020504095870 | 6.135140589521300 | 8.50839160037985E-10 | 1.26260749842259E-07 | 6.898731635796020 | up |
| **LP_90D1.tig00161.7** | 7 | 0 | 2 | 836 | 138 | 268 | 493.8343850633730 | 8.698950659741460 | 1.6021108205353300 | 5.429680986009920 | 5.64548753442838E-08 | 5.29486469907386E-06 | 5.2761451330440100 | up |
| **LP_90D1.tig00055.12** | 0 | 0 | 3 | 347 | 154 | 160 | 224.19516497704700 | 9.264514409539150 | 1.604035324835860 | 5.775754602216860 | 7.66089603130731E-09 | 8.68424470679787E-07 | 6.061267947387740 | up |
| **LP_90D2.tig01125.4** | 0 | 0 | 6 | 56 | 75 | 182 | 65.45932235369170 | 6.482916208683550 | 1.3093534492130900 | 4.951234681956750 | 7.37441083548697E-07 | 5.59600462241078E-05 | 4.252121935444210 | up |
| **C0_90D1.tig00201.5** | 56 | 30 | 204 | 8913 | 4675 | 4436 | 5923.803859884680 | 7.378263099076960 | 1.1202368432247800 | 6.586342114795510 | 4.5079443901591E-11 | 1.34303800601697E-08 | 7.871911697256900 | up |
| **LP_90D1.tig01972.1** | 1 | 3 | 0 | 72 | 6 | 5 | 39.611841627407200 | 6.256317343704610 | 1.8553453236829600 | 3.372050078141510 | 0.0007461088076207320 | 0.03518619629611390 | 1.4536276787997600 | up |
| **C0_90D2.tig20246.1** | 15 | 13 | 20 | 0 | 1 | 2 | 6.429869089249060 | -3.4829241281360300 | 0.9877811131842250 | -3.5260080210568400 | 0.00042187419149547600 | 0.021342359666427900 | 1.6707575655369000 | down |
| **C0_90D3.tig11299.1** | 19 | 32 | 54 | 0 | 1 | 3 | 13.468863617932300 | -4.2081200690445800 | 0.9880280338040820 | -4.259109989867980 | 2.05242466175976E-05 | 0.0012528045161151100 | 2.902116689736690 | down |
| **C0_90D3.tig07752.1** | 0 | 10 | 67 | 1948 | 1102 | 1033 | 1315.4065375558900 | 7.160858859613280 | 1.6322024652876100 | 4.387236885070780 | 1.14799738923211E-05 | 0.0007314978020815060 | 3.13578697445365 | up |
| **C0_90D1.tig00244.1** | 678 | 5 | 107 | 60370 | 16825 | 28296 | 37833.2578168869 | 8.473002445170920 | 1.6045471423183200 | 5.280619198840590 | 1.28748030764525E-07 | 1.10555236984949E-05 | 4.956420680133700 | up |
| **C0_90D3.tig00765.2** | 29 | 10 | 44 | 6044 | 3767 | 1990 | 3935.538514682170 | 8.561282226763860 | 1.093528315251910 | 7.829044851748180 | 4.9159026167566E-15 | 1.26259286516066E-11 | 10.898736669174800 | up |
| **C0_90D1.tig03455.1** | 0 | 0 | 1 | 84 | 32 | 29 | 52.243165467579800 | 8.576499198539770 | 1.9159118610119000 | 4.476458115359260 | 7.5891548414062E-06 | 0.0004978276836929500 | 3.302920956379920 | up |
| **LP_90D1.tig00763.3** | 4 | 0 | 0 | 578 | 344 | 239 | 380.745352178774 | 9.475200272284510 | 1.6200618135194000 | 5.8486658923838900 | 4.95531280191159E-09 | 5.88800495170911E-07 | 6.230031833436350 | up |
| **LP_90D3.tig01264.1** | 10 | 5 | 6 | 31 | 20 | 33 | 26.152426677176700 | 3.079327706337700 | 0.9018163210596360 | 3.4145841391731400 | 0.0006387946034620950 | 0.030956042111750200 | 1.5092545712096400 | up |
| **LP_90D3.tig13972.1** | 68 | 9 | 131 | 18364 | 5417 | 7512 | 11384.942574101000 | 8.779666953119090 | 1.2915732167518400 | 6.7976533108970400 | 1.06336966427025E-11 | 4.86367804387935E-09 | 8.313035181349300 | up |
| **LP_90D1.tig00056.4** | 0 | 0 | 3 | 776 | 370 | 230 | 486.07455280744000 | 10.385909727910500 | 1.6622235275613400 | 6.24820281731165 | 4.15202310021725E-10 | 6.89710941756983E-08 | 7.161332884193770 | up |
| **C0_90D1.tig00452.1** | 7235 | 69 | 243 | 586907 | 115628 | 172622 | 347088.1858972080 | 8.393077913860810 | 1.7562244465900500 | 4.779046283154250 | 1.76128650684521E-06 | 0.00012646794661732200 | 3.8980195327582600 | up |
| **LP_90D1.tig00144.3** | 0 | 0 | 9 | 177 | 70 | 432 | 163.90125326463700 | 7.248433747662690 | 1.661346104678340 | 4.362988378671450 | 1.28297761261852E-05 | 0.0008097795748151200 | 3.0916331817006700 | up |
| **C0_90D1.tig09408.1** | 870 | 257 | 1747 | 93740 | 29497 | 42574 | 59177.53445365440 | 7.366159592802180 | 1.176370861921820 | 6.261766447332920 | 3.80640743082832E-10 | 6.55114111896529E-08 | 7.183683045274550 | up |
| **LP_90D1.tig00133.9** | 1 | 0 | 0 | 150 | 71 | 38 | 93.04242729854350 | 9.416225393701860 | 1.9297832148320100 | 4.879421336723330 | 1.06397548664602E-06 | 7.87695732231126E-05 | 4.1036415075502300 | up |
| **C0_3D2.tig10285.1** | 57 | 304 | 247 | 4 | 12 | 42 | 84.07006315617720 | -2.927965734447170 | 0.8774684359314360 | -3.336833115072820 | 0.0008473881328907480 | 0.0392964477348461 | 1.4056467065935600 | down |
| **LP_90D1.tig00144.8** | 0 | 0 | 2 | 55 | 28 | 108 | 47.913194580353400 | 7.588776225190430 | 1.4842600808369600 | 5.112834551820040 | 3.17360241224296E-07 | 2.55333520343085E-05 | 4.592892167003740 | up |
| **C0_90D1.tig04312.1** | 355 | 149 | 777 | 63072 | 20335 | 26018 | 39425.205455378800 | 7.952787973201470 | 1.1546772210075100 | 6.887455497097500 | 5.67991330234041E-12 | 3.05881653632007E-09 | 8.514446570629980 | up |
| **C0_90D1.tig17842.2** | 1 | 0 | 18 | 718 | 355 | 287 | 463.83553737612100 | 7.670269055180580 | 1.6300482786141200 | 4.705547164346490 | 2.53185960356756E-06 | 0.00017793739700521000 | 3.749732766889830 | up |
| **LP_90D1.tig02298.2** | 0 | 0 | 3 | 206 | 130 | 134 | 143.74032621477300 | 8.615286517328660 | 1.4919172095640600 | 5.774641154415030 | 7.7117258018763E-09 | 8.69887881077188E-07 | 6.060536719527740 | up |
| **C0_90D2.tig20824.1** | 19 | 23 | 48 | 0 | 2 | 1 | 11.494395697776100 | -4.38603007942029 | 1.0548371906044300 | -4.15801615499264 | 3.21023326342324E-05 | 0.001924353293221520 | 2.7157151925834000 | down |
| **LP_90D1.tig00161.4** | 1 | 0 | 2 | 949 | 183 | 300 | 562.3879735545450 | 10.551615486520300 | 1.6036361061070000 | 6.5798066321514100 | 4.71060487931773E-11 | 1.3558826406512E-08 | 7.867777899497960 | up |
| **C0_90D3.tig09963.1** | 236 | 54 | 298 | 39918 | 11383 | 15573 | 24607.70965069580 | 8.371328719399500 | 1.1951768394667800 | 7.004259489444520 | 2.48295551728407E-12 | 1.50733457757451E-09 | 8.82179033821838 | up |
| **C0_90D3.tig00883.5** | 0 | 0 | 1 | 17 | 13 | 8 | 11.808301022417100 | 6.366873411125950 | 1.7908299061383200 | 3.5552641762920100 | 0.00037759949686140500 | 0.019248350535428200 | 1.715606480911550 | up |
| **C0_90D1.tig02299.3** | 31 | 10 | 59 | 6246 | 2162 | 2715 | 3939.720072625390 | 8.303369157240020 | 1.1606242561893200 | 7.154226799035250 | 8.4145848091904E-13 | 7.20396338959124E-10 | 9.142428502923420 | up |
| **C0_90D1.tig00064.11** | 64 | 26 | 209 | 12430 | 4448 | 5720 | 7915.891222885150 | 7.750868910528160 | 1.1919809834555200 | 6.5025105417861400 | 7.89904838609898E-11 | 1.96821885495119E-08 | 7.705926612097230 | up |
| **LP_90D2.tig00110.6** | 0 | 1 | 59 | 810 | 624 | 472 | 577.5786129909520 | 6.333648381017380 | 1.811068615730120 | 3.4971885250543300 | 0.0004701894250992150 | 0.023536963590161500 | 1.6282495644787300 | up |
| **C0_90D2.tig07120.1** | 12 | 24 | 60 | 1 | 0 | 2 | 12.476335731844100 | -4.285807825224680 | 1.2290151253214100 | -3.4871888367556500 | 0.00048812656828123900 | 0.02425306248265220 | 1.6152334143672400 | down |
| **LP_90D2.tig08444.1** | 1 | 2 | 4 | 222 | 160 | 128 | 155.60825511541900 | 7.477093193036650 | 1.1159731329472300 | 6.70006559502915 | 2.0832600437942E-11 | 8.5874036545981E-09 | 8.066138122422770 | up |
| **LP_90D1.tig02740.2** | 0 | 0 | 1 | 216 | 312 | 119 | 170.00211866245200 | 10.28961953686200 | 1.7144977805016700 | 6.001535641446650 | 1.95460024756037E-09 | 2.55929990846248E-07 | 6.591878818857430 | up |
| **LP_90D1.tig00055.8** | 1 | 0 | 0 | 240 | 101 | 137 | 157.98781957142100 | 10.183455749377900 | 1.8447117885749600 | 5.520350556899010 | 3.38324023043832E-08 | 3.29338215901181E-06 | 5.4823578714987700 | up |
| **LP_90D3.tig02330.1** | 31 | 0 | 2 | 4812 | 944 | 1435 | 2844.0717001045700 | 9.309541797067420 | 1.8281839305898700 | 5.092234780809870 | 3.53867524106282E-07 | 2.79983003847978E-05 | 4.55286833136875 | up |
| **LP_90D3.tig00989.1** | 0 | 0 | 1 | 51 | 14 | 16 | 30.83897792807070 | 7.805873078046790 | 1.9672628157828000 | 3.9678852339516900 | 7.25132370328144E-05 | 0.004138708497929300 | 2.383135161228860 | up |
| **LP_90D1.tig00055.6** | 0 | 0 | 2 | 186 | 67 | 72 | 116.26591104107900 | 8.888824763275740 | 1.704077542778640 | 5.216209086812900 | 1.82622026494961E-07 | 1.53205197051263E-05 | 4.814726502246480 | up |
| **LP_90D1.tig00056.5** | 2 | 1 | 2 | 163 | 57 | 69 | 102.93393797322500 | 7.331750472263700 | 1.2898462419035600 | 5.684205011477550 | 1.31422550581362E-08 | 1.43871066929872E-06 | 5.842026535709680 | up |
| **C0_90D3.tig21701.1** | 0 | 5 | 42 | 780 | 402 | 297 | 506.9990757606080 | 6.491732121470470 | 1.6379877454992100 | 3.9632360738400900 | 7.39405992259275E-05 | 0.004212973835417220 | 2.3754112380412500 | up |
| **LP_90D2.tig14173.1** | 0 | 0 | 1 | 256 | 370 | 219 | 212.56355014835700 | 10.612506012890300 | 1.6477375189758600 | 6.440653253733310 | 1.18960367939176E-10 | 2.57919982150724E-08 | 7.588515009930220 | up |
| **C0_90D1.tig17819.1** | 28 | 33 | 89 | 1 | 4 | 9 | 20.702490358460100 | -2.912483555667250 | 0.8082420399748290 | -3.603479417821360 | 0.00031398562129698100 | 0.01627898433149830 | 1.7883726948390700 | down |
| **C0_90D3.tig01980.2** | 31 | 13 | 202 | 8985 | 4769 | 4311 | 5949.99195594027 | 7.642654932006960 | 1.267637135759810 | 6.029055726129390 | 1.64920419128528E-09 | 2.21145697762347E-07 | 6.65532150511304 | up |
| **C0_90D1.tig01009.2** | 48 | 18 | 118 | 4982 | 2239 | 2670 | 3293.015093237570 | 7.1708476068334700 | 1.1110829763914300 | 6.453926267615870 | 1.08988852573048E-10 | 2.40995284672946E-08 | 7.617991454771830 | up |
| **C0_3D3.tig14745.1** | 1 | 0 | 1 | 11 | 9 | 6 | 8.011913794558070 | 4.826145725062970 | 1.4362063525158400 | 3.360342834167850 | 0.0007784580767402110 | 0.03640821609220720 | 1.4388005998436000 | up |
| **C0_90D2.tig16126.1** | 8 | 161 | 317 | 12106 | 5091 | 6558 | 7970.009968294120 | 7.084204676028270 | 1.3352065088946600 | 5.305699626863640 | 1.12241655344636E-07 | 9.84343357692372E-06 | 5.006853384962530 | up |
| **LP_90D1.tig01533.2** | 0 | 0 | 2 | 51 | 17 | 19 | 31.76837812959550 | 6.995267922350430 | 1.7205947314974000 | 4.065610451022710 | 4.79068988196185E-05 | 0.0027964395886158100 | 2.5533945582825200 | up |
| **C0_90D2.tig13216.2** | 0 | 4 | 34 | 2232 | 1230 | 949 | 1463.710054400190 | 8.339145283058000 | 1.5973401005923900 | 5.2206447956608200 | 1.78301236419283E-07 | 1.49957178408147E-05 | 4.8240327398515800 | up |
| **C0_90D1.tig00064.14** | 187 | 53 | 515 | 35822 | 12781 | 16822 | 22843.261227411200 | 7.9380866089408800 | 1.2053542687940700 | 6.5856875563088500 | 4.52785107073872E-11 | 1.34303800601697E-08 | 7.871911697256900 | up |
| **LP_3D1.tig02057.2** | 4 | 0 | 0 | 18 | 22 | 26 | 16.47557231934030 | 4.887001710421910 | 1.3072042994459200 | 3.7385141041024300 | 0.0001851111094746800 | 0.010001091964806000 | 1.9999525791600800 | up |
| **C0_3D1.tig20266.1** | 5 | 2 | 8 | 529 | 193 | 257 | 339.6072401657220 | 7.4896371516490500 | 1.1589210864035400 | 6.4625945972659000 | 1.02922763209776E-10 | 2.38645009778556E-08 | 7.622247642581190 | up |
| **LP_90D1.tig07481.1** | 50 | 368 | 2169 | 26709 | 15973 | 14889 | 18432.03353707100 | 5.881543504486050 | 1.354968377171650 | 4.340723815830400 | 1.42014131906298E-05 | 0.0008846473601155600 | 3.0532298141340900 | up |
| **C0_90D1.tig02904.1** | 251 | 154 | 1074 | 42488 | 35466 | 17998 | 29497.341488358900 | 7.34945348119788 | 1.1009454516341300 | 6.675583672460010 | 2.46250117808172E-11 | 9.34323316306484E-09 | 8.02950281307369 | up |
| **LP_90D2.tig20852.1** | 0 | 6 | 28 | 394 | 250 | 195 | 269.7504697580760 | 6.034747224849680 | 1.490097691770700 | 4.049900391214300 | 5.1239438530469E-05 | 0.0029701972449545600 | 2.527214709083140 | up |
| **C0_90D3.tig00037.3** | 14 | 0 | 44 | 2709 | 1461 | 1015 | 1754.6322942875100 | 7.943274390664370 | 1.5818815286736700 | 5.021409155288910 | 5.12937627670065E-07 | 3.98290103494786E-05 | 4.399800484579880 | up |
| **LP_90D1.tig00628.2** | 92 | 41 | 46 | 7903 | 2202 | 4347 | 5053.148718470140 | 7.767136674739170 | 1.0917059463343000 | 7.114678362630050 | 1.12174118371132E-12 | 8.51223099612214E-10 | 9.069956599454570 | up |
| **C0_90D1.tig19225.2** | 16 | 23 | 60 | 1 | 0 | 2 | 12.901216465624100 | -4.3535348063016 | 1.1801707740397700 | -3.6889024046912300 | 0.00022522358684809400 | 0.012051266572549700 | 1.918967306901340 | down |
| **C0_90D1.tig06035.3** | 98 | 47 | 304 | 24551 | 13237 | 7763 | 15688.134022904400 | 8.150682413759730 | 1.1599390584080900 | 7.0268195166613000 | 2.11294389013154E-12 | 1.33111478391702E-09 | 8.875784493081000 | up |
| **C0_90D3.tig05779.1** | 32 | 2 | 36 | 3279 | 916 | 1242 | 2016.410020047400 | 7.822567218267060 | 1.3510623371602500 | 5.789938038469090 | 7.04123944431947E-09 | 8.07903586963514E-07 | 6.092640463666080 | up |
| **LP_90D1.tig00055.7** | 2 | 0 | 2 | 154 | 57 | 40 | 93.98872506509900 | 7.519946444685280 | 1.4879664983218800 | 5.053841234440580 | 4.33011601041425E-07 | 3.39385548055684E-05 | 4.469306655045160 | up |
| **LP_90D1.tig03143.1** | 1 | 0 | 1 | 67 | 23 | 21 | 41.203922086872100 | 7.321913851673850 | 1.619928956853430 | 4.519898123122640 | 6.18693967083576E-06 | 0.00041481070013962900 | 3.3821500494101400 | up |
| **LP_90D2.tig00340.5** | 6 | 0 | 9 | 1046 | 264 | 377 | 635.932982843835 | 8.395911382510320 | 1.4966979328411700 | 5.6096231566061200 | 2.02767657595027E-08 | 2.10254947808707E-06 | 5.677253775407650 | up |
| **C0_90D1.tig00101.3** | 25 | 4 | 39 | 4596 | 1067 | 1505 | 2760.985803521270 | 8.3370924050295 | 1.3073648501805500 | 6.377020465157950 | 1.80566340453598E-10 | 3.64488406410864E-08 | 7.438316281095970 | up |
| **C0_90D3.tig01758.1** | 51 | 15 | 298 | 10382 | 5496 | 2531 | 6534.912202082600 | 7.207997977563600 | 1.3602062828307000 | 5.2991947387297600 | 1.16314525153178E-07 | 1.01665593726164E-05 | 4.992825998729580 | up |
| **C0_90D1.tig02652.2** | 34 | 2 | 48 | 3653 | 1077 | 1432 | 2261.2067176311900 | 7.736151268974630 | 1.3625790145431900 | 5.6775799321760400 | 1.36613695080755E-08 | 1.47617950325285E-06 | 5.830860829144270 | up |
| **LP_90D1.tig03543.3** | 0 | 6 | 0 | 3489 | 5655 | 1045 | 2699.388618628310 | 11.821419598463700 | 1.6176502528433500 | 7.307772231781950 | 2.7160766914836E-13 | 3.23882445185521E-10 | 9.489612590630360 | up |
| **LP_90D3.tig01224.2** | 0 | 0 | 3 | 45 | 37 | 191 | 55.7508992186481 | 7.237792928853140 | 1.3662846654246800 | 5.297426745694630 | 1.1744599710299E-07 | 1.02386537787774E-05 | 4.989757142469880 | up |
| **LP_90D1.tig13242.1** | 3 | 0 | 3 | 319 | 178 | 57 | 198.43321455866200 | 8.01765004991067 | 1.4616860856691500 | 5.48520652178218 | 4.12986651952318E-08 | 3.98532119133987E-06 | 5.399536671506180 | up |
| **LP_90D1.tig02740.1** | 0 | 0 | 1 | 69 | 60 | 41 | 49.70990246569290 | 8.501336492139910 | 1.7481351238662300 | 4.863088886022780 | 1.15567917515317E-06 | 8.49933303506369E-05 | 4.070615153174690 | up |
| **LP_90D1.tig00618.2** | 1 | 1 | 7 | 563 | 240 | 121 | 343.3110905520700 | 8.286766618415810 | 1.3858174286478800 | 5.979695771686950 | 2.23554741808236E-09 | 2.84895773825771E-07 | 6.545313993083310 | up |
| **C0_3D1.tig00390.3** | 114 | 28 | 73 | 1525 | 230 | 392 | 909.783136725063 | 4.993410125422200 | 1.3047008119077800 | 3.8272453575932700 | 0.0001295853296861910 | 0.007139809526224810 | 2.146313374051970 | up |
| **C0_90D1.tig04670.2** | 6 | 0 | 0 | 171 | 28 | 94 | 107.12562448844100 | 7.031572048065580 | 1.8407157151329800 | 3.820020652976060 | 0.00013344050012354500 | 0.007316001409893360 | 2.1357262195011400 | up |
| **FM3.tig26716.1** | 4 | 1 | 1 | 69 | 7 | 15 | 39.874679315393000 | 5.628502845034620 | 1.543066816599990 | 3.647607987213760 | 0.0002646930757835270 | 0.01396182797367480 | 1.8550577173211800 | up |
| **C0_90D1.tig06243.2** | 112 | 24 | 204 | 15298 | 3492 | 5052 | 9202.445623235750 | 7.757855429485470 | 1.2820330627889400 | 6.051213228938880 | 1.43759013317442E-09 | 1.97529617105187E-07 | 6.704367778108330 | up |
| **C0_90D3.tig06812.1** | 17 | 42 | 77 | 0 | 0 | 2 | 16.865440023260200 | -5.45279217266678 | 1.326179454219140 | -4.111654840767690 | 3.9283328041876E-05 | 0.002325586950337230 | 2.6334674182054800 | down |
| **LP_90D3.tig17053.1** | 2492 | 4774 | 9727 | 102 | 155 | 828 | 2266.931778209190 | -3.442796359464400 | 0.8404073897125550 | -4.096580303324010 | 4.19298080845788E-05 | 0.0024734882723250900 | 2.606690144437290 | down |
| **C0_90D1.tig23835.1** | 1 | 0 | 0 | 68 | 10 | 15 | 39.08514250137540 | 8.154691125182520 | 2.082067964657960 | 3.9166306112981100 | 8.97951757415765E-05 | 0.005030488461133390 | 2.2983898428416300 | up |
| **C0_90D1.tig18545.1** | 28 | 28 | 66 | 1 | 11 | 5 | 17.711617769174400 | -2.387222295045010 | 0.7136215987910720 | -3.3452214718404600 | 0.0008221689059271410 | 0.03817996884562070 | 1.418164430321100 | down |
| **C0_90D2.tig16190.1** | 0 | 2 | 5 | 55 | 32 | 32 | 38.23373961677160 | 5.440129120006970 | 1.2850622561445300 | 4.23335841823612 | 2.30227143505491E-05 | 0.0013925822634972500 | 2.8561791404790700 | up |
| **LP_90D3.tig00053.13** | 0 | 0 | 23 | 1028 | 577 | 404 | 671.4317509596490 | 7.944840399833580 | 1.9667527760830600 | 4.039572485392070 | 5.35487173990965E-05 | 0.0030933185557758400 | 2.5095753532293600 | up |
| **LP_90D1.tig00022.2** | 0 | 1 | 0 | 582 | 282 | 277 | 379.83542542364100 | 11.452496541806900 | 1.8478441184922000 | 6.197761178660430 | 5.72719527161466E-10 | 9.23793830550443E-08 | 7.034424942464930 | up |
| **LP_90D1.tig00193.6** | 0 | 0 | 1 | 119 | 40 | 33 | 72.128344569992 | 9.046779529323630 | 1.9576684955755500 | 4.621200959084710 | 3.81524979734249E-06 | 0.00026374197822664300 | 3.5788207406606400 | up |
| **LP_90D1.tig01062.1** | 0 | 0 | 15 | 936 | 158 | 529 | 586.2957991570650 | 8.364841652891720 | 1.9681449236003300 | 4.250114690533000 | 2.13661084654113E-05 | 0.001299440793354500 | 2.8862435035467400 | up |
| **LP_90D2.tig01794.1** | 0 | 0 | 11 | 303 | 118 | 151 | 196.23739763395000 | 7.222600862293980 | 1.8075388367456400 | 3.9958205685349500 | 6.44705520047591E-05 | 0.003705003891371600 | 2.431211331544210 | up |
| **C0_90D2.tig03452.2** | 36 | 9 | 51 | 2934 | 946 | 1537 | 1885.0719487949700 | 7.28005025595428 | 1.1496086803992800 | 6.332633338699050 | 2.41011844050365E-10 | 4.58639988804127E-08 | 7.338528081834960 | up |
| **LP_90D1.tig01705.2** | 0 | 0 | 1 | 284 | 119 | 173 | 188.38681093079200 | 10.438429110996100 | 1.8382006771018800 | 5.6786123740599400 | 1.35791810674409E-08 | 1.47206258656099E-06 | 5.832073725039110 | up |
| **C0_3D3.tig03887.1** | 0 | 0 | 3 | 13 | 6 | 169 | 31.919428774886800 | 6.449027993082520 | 1.7296064695210100 | 3.728610008534770 | 0.00019253885483589700 | 0.010368838425993100 | 1.9842698929298500 | up |
| **LP_90D3.tig17533.1** | 0 | 0 | 2 | 325 | 230 | 167 | 223.3484897047930 | 9.827866150884580 | 1.5632490129078400 | 6.2868206342913400 | 3.24033385984305E-10 | 5.72441837282009E-08 | 7.242268633059140 | up |
| **LP_90D1.tig03247.1** | 0 | 0 | 6 | 317 | 146 | 118 | 201.87835651086300 | 8.131321607708460 | 1.6942743733035700 | 4.799294456572380 | 1.59225551638266E-06 | 0.00011507320224350800 | 3.939025801242360 | up |
| **LP_90D2.tig19429.1** | 0 | 0 | 4 | 33 | 14 | 48 | 26.3310584340332 | 5.724662167473740 | 1.4904736485813500 | 3.8408342025520100 | 0.00012261691294793100 | 0.006767034886642100 | 2.1696015845573000 | up |
| **LP_90D3.tig00178.2** | 0 | 0 | 5 | 40 | 33 | 219 | 56.83581011715940 | 6.545769450979340 | 1.4575847322584000 | 4.49083288683823 | 7.09451996320015E-06 | 0.000469067182279782 | 3.3287649508801000 | up |
| **C0_90D2.tig01490.1** | 57 | 64 | 185 | 1 | 5 | 20 | 41.493473654628800 | -3.1345878175516200 | 0.9041610772313910 | -3.466846667576050 | 0.0005266021934100020 | 0.02604847501595050 | 1.5842176969918600 | down |
| **LP_90D1.tig00184.3** | 1 | 0 | 0 | 126 | 27 | 28 | 73.42389964019850 | 9.073035502365280 | 2.036785654355330 | 4.4545853330045200 | 8.40554930681948E-06 | 0.0005460172875591360 | 3.2627936067958400 | up |
| **LP_90D3.tig00646.1** | 0 | 0 | 21 | 109 | 351 | 645 | 196.16364174732800 | 6.287007411437140 | 1.714362595401540 | 3.667256523387100 | 0.0002451668104824100 | 0.01301410911796060 | 1.8855855562060100 | up |
| **LP_90D1.tig00070.7** | 24 | 3 | 78 | 6441 | 2015 | 2981 | 4060.897617471550 | 8.300135584886570 | 1.348731036188150 | 6.154033207647390 | 7.55368569549745E-10 | 1.1412217723392E-07 | 6.942629951423850 | up |
| **C0_90D3.tig04852.1** | 23 | 44 | 140 | 0 | 1 | 6 | 26.039674018063300 | -4.4399354620545400 | 1.1817694645581700 | -3.757023341024050 | 0.00017194646428359900 | 0.009365612554592290 | 2.0284638124820900 | down |
| **LP_90D1.tig04655.2** | 0 | 8 | 89 | 773 | 727 | 460 | 574.1781893844850 | 5.616417997079710 | 1.6623067556838400 | 3.378689268918500 | 0.00072832278535124 | 0.034444715977468200 | 1.4628773919809800 | up |
| **C0_90D3.tig01506.2** | 144 | 41 | 139 | 19195 | 5989 | 8750 | 12083.86259233370 | 8.192604145681780 | 1.126702360011060 | 7.2713117824669800 | 3.5601316876735E-13 | 3.71466365374157E-10 | 9.430080503555350 | up |
| **C0_90D3.tig23438.2** | 7 | 0 | 8 | 511 | 282 | 163 | 328.3220562615390 | 7.42412793824111 | 1.4070997055272000 | 5.276191807217750 | 1.31896037370887E-07 | 1.1291991773786E-05 | 4.947229446908460 | up |
| **LP_90D2.tig05550.1** | 0 | 25 | 2 | 19813 | 35731 | 6285 | 15843.162090934900 | 12.214442120439700 | 1.5768605777049700 | 7.746050787963250 | 9.4794607872916E-15 | 2.06563542629465E-11 | 10.684946326727400 | up |
| **C0_90D1.tig01132.2** | 76 | 11 | 96 | 9793 | 2732 | 3891 | 6044.080559141380 | 8.027707961241340 | 1.2529994732544100 | 6.406792766154180 | 1.48612595925866E-10 | 3.10126622835545E-08 | 7.5084609501306900 | up |
| **C0_90D1.tig11271.1** | 40 | 58 | 162 | 2 | 5 | 15 | 35.52770579846580 | -3.0259461166257600 | 0.8382587593496600 | -3.6097995790385300 | 0.00030643368198676000 | 0.015961800636280700 | 1.796918117854320 | down |
| **C0_90D3.tig16082.1** | 33 | 48 | 159 | 2 | 2 | 9 | 31.73973232008470 | -3.556871915954840 | 0.9605466806738530 | -3.7029662248789300 | 0.00021309326102827000 | 0.011420499024836100 | 1.9423149189450200 | down |
| **C0_90D3.tig07699.2** | 86 | 15 | 224 | 10485 | 6533 | 3649 | 6876.957486002590 | 7.417302346447000 | 1.2315223249426600 | 6.02287282676124 | 1.71348077000572E-09 | 2.28845637718883E-07 | 6.640457361727720 | up |

Note- Genes were identified using differential expression analysis with thresholds of |log₂ fold change| ≥ 1.5 and adjusted P < 0.05. Positive log₂ fold-change values indicate genes upregulated in LP-inoculated silage relative to the control, while negative values indicate downregulation.
