# Supplementary material for: Metatranscriptomic insights into the effect of Lactiplantibacillus plantarum inoculation on fermentation metabolism, bacterial community function and antibiotic resistome of corn silage
Source: Front Microbiol. 2026 Sep 9;17:1876540. doi: 10.3389/fmicb.2026.1876540 (PMC13597671; doi:10.3389/fmicb.2026.1876540)
Supplement: Supplementary file 4 [file Table_4.docx]

Table S4. Differentially expressed genes between LP-treated and control silage at day 3 and day 90.

| **gene** | **Chart** | **CK3D1** | **CK3D2** | **CK3D3** | **CK 3D mean** | **SD value CK 3D** | **CK90D1** | **CK90D2** | **CK90D3** | **CK 90D mean** | **SD value CK 90D** | **FM1** | **FM2** | **FM3** | **FM mean** | **SD value FM** | **LP3D1** | **LP3D2** | **LP3D3** | **LP 3D mean** | **SD value LP 3D** | **LP90D1** | **LP90D2** | **LP90D3** | **LP 90D mean** | **SD value LP 90D** | **F fold 3D** | **F fold 90D** |
| --- | --- | --- | --- | --- | --- | --- | --- | --- | --- | --- | --- | --- | --- | --- | --- | --- | --- | --- | --- | --- | --- | --- | --- | --- | --- | --- | --- | --- |
| **ackA** | UP (3) | 0.68 | 0.96 | 1.33 | 0.99 | 0.33 | 0.86 | 0.19 | 0.09 | 0.38 | 0.42 | 4.10 | 3.23 | 2.28 | 3.20 | 0.91 | 39.30 | 42.46 | 17.70 | 33.15 | 13.48 | 0.78 | 1.17 | 0.33 | 0.76 | 0.42 | 33.53 | 1.99 |
| **eno** | UP (3) | 2.74 | 4.07 | 6.57 | 4.46 | 1.94 | 0.35 | 0.00 | 0.03 | 0.12 | 0.19 | 14.74 | 14.84 | 15.27 | 14.95 | 0.28 | 152.46 | 137.73 | 41.77 | 110.66 | 60.11 | 0.28 | 0.25 | 0.17 | 0.24 | 0.06 | 24.81 | 1.90 |
| **fbp** | UP (3) | 9.85 | 10.10 | 15.21 | 11.72 | 3.02 | 0.41 | 0.00 | 0.28 | 0.23 | 0.21 | 15.40 | 24.25 | 13.15 | 17.60 | 5.87 | 100.39 | 87.60 | 29.87 | 72.62 | 37.57 | 0.00 | 0.00 | 0.00 | 0.00 | 0.00 | 6.20 | 0.01 |
| **mdh** | UP (3) | 5.59 | 11.73 | 15.86 | 11.06 | 5.17 | 2.40 | 0.22 | 0.47 | 1.03 | 1.19 | 39.01 | 38.91 | 36.97 | 38.30 | 1.15 | 99.38 | 103.80 | 32.80 | 78.66 | 39.77 | 0.82 | 1.03 | 1.18 | 1.01 | 0.18 | 7.11 | 0.98 |
| **ppc** | UP (3) | 9.22 | 16.49 | 23.25 | 16.32 | 7.02 | 2.78 | 0.75 | 0.57 | 1.37 | 1.23 | 41.35 | 35.63 | 43.64 | 40.21 | 4.12 | 138.98 | 132.31 | 42.93 | 104.74 | 53.63 | 1.33 | 2.11 | 0.64 | 1.36 | 0.74 | 6.42 | 1.00 |
| **rnfC** | UP (3) | 2.93 | 6.62 | 8.37 | 5.97 | 2.78 | 0.19 | 0.00 | 0.16 | 0.12 | 0.10 | 13.45 | 12.97 | 13.97 | 13.46 | 0.50 | 69.98 | 64.39 | 19.45 | 51.28 | 27.70 | 0.00 | 0.00 | 0.67 | 0.22 | 0.39 | 8.58 | 1.94 |
| **rnfD** | UP (3) | 0.25 | 0.84 | 0.78 | 0.62 | 0.32 | 0.45 | 0.00 | 0.00 | 0.15 | 0.26 | 0.56 | 0.38 | 0.78 | 0.57 | 0.20 | 26.93 | 23.23 | 6.97 | 19.04 | 10.62 | 0.02 | 0.01 | 0.31 | 0.12 | 0.17 | 30.53 | 0.77 |
| **fdoH** | UP (3) | 0.02 | 0.04 | 0.04 | 0.03 | 0.01 | 0.03 | 0.00 | 0.00 | 0.01 | 0.02 | 0.03 | 0.00 | 0.00 | 0.01 | 0.02 | 9.29 | 10.90 | 2.53 | 7.57 | 4.44 | 0.08 | 0.00 | 0.00 | 0.03 | 0.05 | 217.45 | 2.89 |
| **serA** | UP (3) | 0.01 | 0.04 | 0.02 | 0.02 | 0.02 | 0.00 | 0.00 | 0.00 | 0.00 | 0.00 | 0.12 | 0.07 | 0.06 | 0.08 | 0.03 | 0.31 | 0.48 | 0.14 | 0.31 | 0.17 | 0.01 | 0.00 | 0.00 | 0.00 | 0.01 | 13.80 | #DIV/0! |
| **serB** | UP (3) | 0.03 | 0.02 | 0.06 | 0.04 | 0.02 | 0.00 | 0.00 | 0.00 | 0.00 | 0.00 | 0.06 | 0.03 | 0.09 | 0.06 | 0.03 | 2.71 | 2.45 | 0.78 | 1.98 | 1.05 | 0.02 | 0.04 | 0.02 | 0.03 | 0.01 | 56.00 | #DIV/0! |
| **glyA** | UP (3) | 4.88 | 8.58 | 11.33 | 8.27 | 3.24 | 0.70 | 0.33 | 0.05 | 0.36 | 0.32 | 26.04 | 19.13 | 22.35 | 22.51 | 3.46 | 67.61 | 61.14 | 19.83 | 49.53 | 25.92 | 0.47 | 0.51 | 0.15 | 0.38 | 0.20 | 5.99 | 1.04 |
| **eno** | DOWN (3) | 5.26 | 15.71 | 7.89 | 9.62 | 5.44 | 7960.19 | 5062.15 | 6092.25 | 6371.53 | 1469.07 | 4.20 | 1.47 | 0.55 | 2.07 | 1.90 | 5.76 | 4.13 | 2.22 | 4.04 | 1.77 | 2718.78 | 671.96 | 1909.49 | 1766.74 | 1030.85 | 0.42 | 0.28 |
| **ppc** | DOWN (3) | 0.24 | 0.44 | 0.20 | 0.29 | 0.13 | 51.68 | 39.26 | 58.12 | 49.69 | 9.59 | 0.58 | 0.10 | 0.23 | 0.31 | 0.25 | 0.12 | 0.26 | 0.02 | 0.13 | 0.12 | 27.75 | 9.06 | 18.70 | 18.50 | 9.35 | 0.46 | 0.37 |
| **glyA** | DOWN (3) | 0.48 | 0.92 | 0.36 | 0.59 | 0.29 | 42.96 | 29.95 | 47.66 | 40.19 | 9.17 | 5.11 | 3.97 | 4.01 | 4.36 | 0.65 | 0.25 | 0.25 | 0.25 | 0.25 | 0.00 | 20.62 | 6.91 | 14.53 | 14.02 | 6.87 | 0.42 | 0.35 |
| **mdh** | DOWN (3) | 0.11 | 0.25 | 0.09 | 0.15 | 0.09 | 8.11 | 7.26 | 7.47 | 7.62 | 0.45 | 0.03 | 0.01 | 0.03 | 0.03 | 0.01 | 0.05 | 0.11 | 0.02 | 0.06 | 0.05 | 3.04 | 1.31 | 2.58 | 2.31 | 0.90 | 0.41 | 0.30 |
| **eno** | UP(90) | 0.00 | 0.00 | 0.00 | 0.00 | 0.00 | 41.51 | 19.32 | 168.24 | 76.36 | 80.35 | 0.00 | 0.00 | 0.00 | 0.00 | 0.00 | 0.00 | 0.00 | 0.00 | 0.00 | 0.00 | 7382.87 | 2936.86 | 3700.99 | 4673.58 | 2377.22 | #DIV/0! | 61.21 |
| **mdh** | UP(90) | 0.00 | 0.00 | 0.00 | 0.00 | 0.00 | 29.24 | 23.69 | 338.30 | 130.41 | 180.06 | 0.00 | 0.00 | 0.00 | 0.00 | 0.00 | 0.00 | 0.00 | 0.00 | 0.00 | 0.00 | 9538.15 | 4812.10 | 4531.20 | 6293.82 | 2813.18 | #DIV/0! | 48.26 |
| **apgM** | UP(90) | 0.00 | 0.00 | 0.00 | 0.00 | 0.00 | 42.36 | 31.79 | 232.05 | 102.07 | 112.69 | 0.00 | 0.00 | 0.00 | 0.00 | 0.00 | 0.00 | 0.00 | 0.00 | 0.00 | 0.00 | 6527.37 | 5685.90 | 3963.70 | 5392.32 | 1306.81 | #DIV/0! | 52.83 |
| **ppc** | UP(90) | 0.14 | 0.29 | 0.21 | 0.21 | 0.08 | 35.83 | 8.99 | 93.37 | 46.06 | 43.11 | 0.12 | 0.00 | 0.00 | 0.04 | 0.07 | 0.45 | 0.77 | 0.15 | 0.46 | 0.31 | 4353.95 | 2196.05 | 1678.72 | 2742.91 | 1418.98 | 2.14 | 59.55 |
| **fbp** | UP(90) | 15.41 | 14.01 | 17.65 | 15.69 | 1.83 | 15.60 | 6.13 | 58.24 | 26.66 | 27.76 | 0.71 | 0.64 | 0.70 | 0.68 | 0.04 | 9.23 | 19.13 | 8.19 | 12.18 | 6.04 | 3326.47 | 1196.83 | 1874.07 | 2132.46 | 1088.07 | 0.78 | 80.00 |
| **pfkA** | UP(90) | 0.00 | 0.00 | 0.00 | 0.00 | 0.00 | 17.10 | 5.66 | 55.10 | 25.95 | 25.88 | 0.00 | 0.00 | 0.00 | 0.00 | 0.00 | 0.00 | 0.00 | 0.00 | 0.00 | 0.00 | 3231.18 | 1166.35 | 1101.93 | 1833.15 | 1211.15 | #DIV/0! | 70.63 |
| **glyA** | UP(90) | 0.00 | 0.00 | 0.00 | 0.00 | 0.00 | 57.99 | 22.97 | 219.88 | 100.28 | 105.05 | 0.00 | 0.00 | 0.10 | 0.03 | 0.06 | 0.00 | 0.00 | 0.00 | 0.00 | 0.00 | 9235.31 | 4970.36 | 5755.93 | 6653.86 | 2269.84 | #DIV/0! | 66.35 |
| **serA** | UP(90) | 0.00 | 0.00 | 0.00 | 0.00 | 0.00 | 12.25 | 3.90 | 29.03 | 15.06 | 12.80 | 0.00 | 0.00 | 0.00 | 0.00 | 0.00 | 0.00 | 0.00 | 0.00 | 0.00 | 0.00 | 1153.35 | 384.69 | 720.64 | 752.89 | 385.35 | #DIV/0! | 49.99 |
| **rnfA** | UP(90) | 0.00 | 0.00 | 0.00 | 0.00 | 0.00 | 14.42 | 5.68 | 51.46 | 23.85 | 24.31 | 0.00 | 0.00 | 0.00 | 0.00 | 0.00 | 0.00 | 0.00 | 0.00 | 0.00 | 0.00 | 2477.09 | 925.90 | 1603.68 | 1668.89 | 777.65 | #DIV/0! | 69.96 |
| **ackA** | UP(90) | 22.01 | 23.09 | 24.81 | 23.30 | 1.41 | 1.52 | 1.16 | 7.98 | 3.55 | 3.84 | 2.87 | 2.05 | 1.04 | 1.98 | 0.92 | 29.69 | 51.03 | 23.25 | 34.66 | 14.54 | 442.34 | 190.41 | 237.62 | 290.12 | 133.92 | 1.49 | 81.65 |
| **gpml** | DOWN(90) | 0.88 | 0.47 | 0.00 | 0.45 | 0.44 | 29.44 | 54.42 | 164.18 | 82.68 | 71.68 | 1.08 | 0.29 | 0.81 | 0.73 | 0.40 | 0.53 | 0.31 | 1.55 | 0.80 | 0.66 | 3.30 | 3.51 | 15.77 | 7.53 | 7.14 | 1.77 | 0.09 |
| **mdh** | DOWN(90) | 0.02 | 0.00 | 0.00 | 0.01 | 0.01 | 0.32 | 0.72 | 1.79 | 0.94 | 0.76 | 0.03 | 0.00 | 0.03 | 0.02 | 0.02 | 0.00 | 0.00 | 0.01 | 0.00 | 0.01 | 0.02 | 0.00 | 0.07 | 0.03 | 0.03 | 0.46 | 0.03 |
| **nirK** | DOWN(90) | 0.06 | 0.00 | 0.03 | 0.03 | 0.03 | 0.61 | 0.75 | 0.89 | 0.75 | 0.14 | 0.00 | 0.02 | 0.00 | 0.01 | 0.01 | 0.05 | 0.00 | 0.02 | 0.02 | 0.03 | 0.00 | 0.14 | 0.20 | 0.11 | 0.10 | 0.84 | 0.15 |

Note- List of genes significantly differentially expressed in LP-treated silage relative to control at day 3 and day 90. Differential expression analysis was performed using TPM-normalized transcript abundance. Columns include gene identifier, functional annotation, log₂ fold change (LP vs control), adjusted P value (FDR), and direction of regulation (upregulated or downregulated in LP).

Genes were considered significantly differentially expressed at an adjusted P value (FDR) < 0.05. Positive log₂ fold change values indicate higher expression in LP-treated silage, whereas negative values indicate higher expression in control.
