# Supplementary material for: Metatranscriptomic insights into the effect of Lactiplantibacillus plantarum inoculation on fermentation metabolism, bacterial community function and antibiotic resistome of corn silage
Source: Front Microbiol. 2026 Sep 9;17:1876540. doi: 10.3389/fmicb.2026.1876540 (PMC13597671; doi:10.3389/fmicb.2026.1876540)
Supplement: Supplementary file 5 [file Table_5.docx]

| Comparison | Total families tested | Significant (FDR<0.05) | Percent |
| --- | --- | --- | --- |
| C0_3D vs C0_90D | 6 | 75 | 56.4% |
| LP_3D vs LP_90D | 6 | 1 | 0.8% |
| LP_3D vs C0_3D | 6 | 0 | 0% |
| LP_90D vs C0_90D | 6 | 0 | 0% |

Table S5. Differential analysis of CAZy families across treatments and ensiling stages.

Note-Differential analysis of carbohydrate-active enzyme (CAZy) families was performed using pyDESeq2 (v0.5.1) to compare control (C) and Lactiplantibacillus plantarum MTD1-treated (LP) silages at day 3 and day 90.

“Samples” indicates the total number of CAZy families included in each comparison. “Significant (FDR < 0.05)” represents the number of CAZy families exhibiting statistically significant differential abundance after Benjamini–Hochberg correction. “Percent” denotes the proportion of significant families relative to the total tested.
