# Supplementary material for: Metatranscriptomic insights into the effect of Lactiplantibacillus plantarum inoculation on fermentation metabolism, bacterial community function and antibiotic resistome of corn silage
Source: Front Microbiol. 2026 Sep 9;17:1876540. doi: 10.3389/fmicb.2026.1876540 (PMC13597671; doi:10.3389/fmicb.2026.1876540)
Supplement: Supplementary file 6 [file Table_6.docx]

Table S6. Differential expression of nitrogen-associated genes between LP-treated and control silage.

| **gene** | **Chart** | **CK3D1** | **CK3D2** | **CK3D3** | **CK3D mean** | **SD value CK3D** | **CK90D1** | **CK90D2** | **CK90D3** | **CK90D mean** | **SD value CK90D** | **FM1** | **FM2** | **FM3** | **FM mean** | **SD value FM** | **LP3D1** | **LP3D2** | **LP3D3** | **LP3D mean** | **SD value LP3D** | **LP90D1** | **LP90D2** | **LP90D3** | **LP90D mean** | **SD value LP90D** | **F fold 3D** | **F fold 90D** |
| --- | --- | --- | --- | --- | --- | --- | --- | --- | --- | --- | --- | --- | --- | --- | --- | --- | --- | --- | --- | --- | --- | --- | --- | --- | --- | --- | --- | --- |
| **nirK** | UP (3) | 0.06 | 0.09 | 0.10 | 0.08 | 0.02 | 0.02 | 0.01 | 0.00 | 0.01 | 0.01 | 0.36 | 0.15 | 0.18 | 0.23 | 0.12 | 4.83 | 4.55 | 1.40 | 3.59 | 1.90 | 0.08 | 0.08 | 0.08 | 0.08 | 0.00 | 42.98 | 6.66 |
| **nirS** | UP (3) | 0.15 | 0.53 | 0.51 | 0.40 | 0.21 | 0.03 | 0.01 | 0.00 | 0.01 | 0.02 | 0.86 | 0.80 | 0.76 | 0.81 | 0.05 | 16.83 | 15.17 | 4.81 | 12.27 | 6.51 | 0.12 | 0.25 | 0.08 | 0.15 | 0.09 | 30.95 | 11.84 |
| **nosZ** | UP (3) | 17.54 | 25.26 | 22.15 | 21.65 | 3.89 | 0.70 | 0.16 | 0.28 | 0.38 | 0.28 | 1.76 | 1.44 | 1.59 | 1.60 | 0.16 | 163.80 | 124.01 | 77.52 | 121.78 | 43.19 | 0.75 | 1.26 | 0.73 | 0.91 | 0.30 | 5.63 | 2.40 |
| **nifW** | UP (3) | 23.55 | 36.64 | 29.29 | 29.83 | 6.56 | 0.18 | 0.10 | 0.10 | 0.13 | 0.04 | 0.18 | 0.23 | 0.03 | 0.15 | 0.10 | 205.46 | 108.22 | 83.97 | 132.55 | 64.29 | 0.04 | 0.00 | 0.04 | 0.03 | 0.02 | 4.44 | 0.21 |
| **nifH** | UP (3) | 0.95 | 0.92 | 0.92 | 0.93 | 0.02 | 0.01 | 0.00 | 0.04 | 0.02 | 0.02 | 0.86 | 0.66 | 0.70 | 0.74 | 0.11 | 5.64 | 7.38 | 3.51 | 5.51 | 1.94 | 0.05 | 0.04 | 0.10 | 0.06 | 0.03 | 5.93 | 3.78 |
| **nirD** | UP (3) | 0.06 | 0.15 | 0.13 | 0.11 | 0.05 | 0.03 | 0.00 | 0.00 | 0.01 | 0.02 | 0.49 | 0.13 | 0.18 | 0.27 | 0.20 | 6.13 | 5.97 | 2.07 | 4.72 | 2.30 | 0.10 | 0.13 | 0.22 | 0.15 | 0.06 | 42.26 | 14.28 |
| **nirB** | UP (3) | 0.08 | 0.17 | 0.16 | 0.14 | 0.05 | 0.03 | 0.00 | 0.01 | 0.02 | 0.02 | 0.24 | 0.08 | 0.16 | 0.16 | 0.08 | 4.45 | 4.10 | 1.18 | 3.25 | 1.80 | 0.04 | 0.06 | 0.01 | 0.04 | 0.03 | 23.55 | 2.57 |
| **narJ** | UP (3) | 0.02 | 0.05 | 0.06 | 0.04 | 0.02 | 0.00 | 0.00 | 0.00 | 0.00 | 0.00 | 0.75 | 0.56 | 0.70 | 0.67 | 0.10 | 2.45 | 1.59 | 0.38 | 1.47 | 1.04 | 0.01 | 0.01 | 0.01 | 0.01 | 0.00 | 32.88 | #DIV/0! |
| **napC** | UP (3) | 0.00 | 0.05 | 0.02 | 0.02 | 0.02 | 0.00 | 0.00 | 0.00 | 0.00 | 0.00 | 0.00 | 0.08 | 0.06 | 0.05 | 0.04 | 0.55 | 0.74 | 0.21 | 0.50 | 0.27 | 0.00 | 0.00 | 0.04 | 0.01 | 0.02 | 21.04 | #DIV/0! |
| **glnA** | UP (3) | 0.09 | 0.14 | 0.16 | 0.13 | 0.04 | 0.00 | 0.00 | 0.00 | 0.00 | 0.00 | 0.10 | 0.19 | 0.12 | 0.14 | 0.05 | 2.71 | 2.27 | 0.61 | 1.86 | 1.11 | 0.00 | 0.00 | 0.01 | 0.00 | 0.01 | 14.38 | #DIV/0! |
| **nasB** | UP (3) | 0.35 | 0.53 | 0.75 | 0.55 | 0.20 | 0.17 | 0.04 | 0.00 | 0.07 | 0.09 | 0.37 | 0.24 | 0.25 | 0.29 | 0.07 | 6.49 | 7.82 | 2.76 | 5.69 | 2.62 | 0.18 | 0.18 | 0.08 | 0.15 | 0.06 | 10.43 | 2.15 |
| **gdh_K00261** | UP (3) | 0.70 | 1.26 | 1.55 | 1.17 | 0.44 | 0.16 | 0.06 | 0.10 | 0.11 | 0.05 | 0.36 | 0.35 | 0.38 | 0.37 | 0.02 | 11.80 | 11.97 | 3.62 | 9.13 | 4.78 | 0.07 | 0.19 | 0.18 | 0.15 | 0.06 | 7.79 | 1.38 |
| **nmo** | UP (3) | 0.10 | 0.25 | 0.38 | 0.24 | 0.14 | 0.05 | 0.00 | 0.02 | 0.02 | 0.02 | 0.82 | 0.48 | 0.57 | 0.63 | 0.18 | 8.57 | 10.00 | 3.13 | 7.23 | 3.62 | 0.24 | 0.25 | 0.18 | 0.22 | 0.04 | 29.78 | 10.56 |
| **gs_K00266** | UP (3) | 0.27 | 0.22 | 0.35 | 0.28 | 0.07 | 0.02 | 0.00 | 0.00 | 0.01 | 0.01 | 0.77 | 0.30 | 0.35 | 0.47 | 0.26 | 8.95 | 8.44 | 2.95 | 6.78 | 3.33 | 0.00 | 0.01 | 0.02 | 0.01 | 0.01 | 24.24 | 2.05 |
| **ureB** | UP (3) | 0.18 | 0.46 | 0.50 | 0.38 | 0.18 | 0.06 | 0.01 | 0.00 | 0.02 | 0.03 | 0.28 | 0.24 | 0.26 | 0.26 | 0.02 | 3.11 | 2.24 | 0.62 | 1.99 | 1.26 | 0.00 | 0.01 | 0.03 | 0.01 | 0.01 | 5.26 | 0.51 |
| **nirA** | UP (90) | 0.00 | 0.00 | 0.00 | 0.00 | 0.00 | 9.17 | 3.89 | 30.18 | 14.41 | 13.91 | 0.00 | 0.00 | 0.00 | 0.00 | 0.00 | 0.02 | 0.00 | 0.00 | 0.01 | 0.01 | 1355.33 | 514.98 | 896.64 | 922.32 | 420.76 | #DIV/0! | 64.00 |
| **nosZ** | UP (90) | 0.00 | 0.00 | 0.00 | 0.00 | 0.00 | 4.16 | 1.91 | 23.01 | 9.69 | 11.58 | 0.00 | 0.00 | 0.00 | 0.00 | 0.00 | 0.00 | 0.00 | 0.00 | 0.00 | 0.00 | 751.55 | 449.56 | 489.27 | 563.46 | 164.09 | #DIV/0! | 58.14 |
| **nifH** | UP (90) | 0.00 | 0.00 | 0.00 | 0.00 | 0.00 | 1.64 | 0.67 | 5.63 | 2.64 | 2.63 | 0.00 | 0.00 | 0.00 | 0.00 | 0.00 | 0.00 | 0.00 | 0.00 | 0.00 | 0.00 | 259.60 | 89.05 | 151.32 | 166.65 | 86.30 | #DIV/0! | 63.03 |
| **glnA** | UP (90) | 0.00 | 0.00 | 0.00 | 0.00 | 0.00 | 1.31 | 0.43 | 1.50 | 1.08 | 0.57 | 0.00 | 0.00 | 0.00 | 0.00 | 0.00 | 0.00 | 0.00 | 0.00 | 0.00 | 0.00 | 144.09 | 47.10 | 91.04 | 94.08 | 48.56 | #DIV/0! | 87.28 |
| **glsA** | UP (90) | 0.00 | 0.00 | 0.00 | 0.00 | 0.00 | 0.23 | 0.07 | 0.95 | 0.42 | 0.47 | 0.00 | 0.00 | 0.00 | 0.00 | 0.00 | 0.00 | 0.00 | 0.00 | 0.00 | 0.00 | 101.66 | 48.50 | 45.21 | 65.12 | 31.69 | #DIV/0! | 155.76 |
| **nirS** | UP (90) | 0.00 | 0.00 | 0.00 | 0.00 | 0.00 | 1.17 | 0.69 | 3.38 | 1.75 | 1.44 | 0.00 | 0.01 | 0.00 | 0.00 | 0.00 | 0.00 | 0.00 | 0.00 | 0.00 | 0.00 | 117.30 | 43.46 | 84.13 | 81.63 | 36.98 | 0.00 | 46.72 |

Note- Nitrogen-associated genes significantly differentially expressed between *Lactiplantibacillus plantarum* MTD1–treated (LP) and control (C) silages at day 3 and day 90. Differential expression analysis was performed using pyDESeq2 (v0.5.1)2. Columns include gene identifier, functional annotation, log₂ fold change (LP vs control), raw P value, and adjusted P value (FDR). Positive log₂ fold change values indicate higher expression in LP-treated silage, whereas negative values indicate higher expression in control.

Genes with FDR < 0.05 were considered statistically significant after Benjamini–Hochberg correction.
